# Ethnobotanical study of medicinal plants used by the people of Mosop, Nandi County in Kenya

**DOI:** 10.3389/fphar.2023.1328903

**Published:** 2024-01-19

**Authors:** Z. C. Maiyo, S. N. Njeru, F. J. Toroitich, S. A. Indieka, M. A. Obonyo

**Affiliations:** ^1^ Faculty of Science, Department of Biochemistry and Molecular Biology, Njoro, Kenya; ^2^ Centre for Traditional Medicine and Drug Research (CTMDR), Kenya Medical Research Institute, Nairobi, Kenya; ^3^ Faculty of Science, Department of Biological Sciences, Egerton University, Njoro, Kenya

**Keywords:** ethnomedicinal plants, traditional medicine, Kenyan medicinal plants, herbal medicine, quantitative indices

## Abstract

**Background:** Throughout the history, nature has provided mankind with most of their basic needs, which include food, shelter, medicine, clothes, flavours, scents as well as raw materials. Given that they are an integral part of cultural heritage, medicinal plants have played a significant role in human healthcare systems around the world. Investigating various biological resources for use as medicines requires ethnomedicinal studies.

**Methods:** Data on utilization of ethnomedicinal plants from local healers in Kenya’s Mosop Sub-County in Nandi County was documented through open-ended, semi-structured questionnaires. A number of quantitative indices, such as the Use Citation (UC), Informant Consensus Factor (ICF), Use Value (UV), Frequency of Citation (FoC) and Relative Frequency of Citation (RFC) were used to convey the potential medical benefits, vitality and variety of the ethnomedicine.

**Results:** 102 informants provided information on 253 ethnomedicinal plant species, classified into 74 families. There were 249 native plant species identified, along with few exotic species: *Senegalia senegal* (L.) Britton, *Persea americana* Mill, *Carica papaya* L. and *Solanum betaceum* Cav. Of all recorded species, 32% and 27% were herbs and trees, respectively. Among plant parts, leaves were most frequently utilized (27%) and roots (26%), while decoctions (21%) were the most widely used formulations. The dominant family was Asteraceae, with 28 species, followed by Lamiaceae, with 19 species. The highest ICF value was 0.778 for a number of parasitic and infectious illnesses, including ringworms, athlete’s foot rot, tetanus, typhoid, intestinal parasites, abscesses, malaria, and amoebiasis. The study’s data validates the region’s widespread use of traditional medicinal plant remedies.

**Conclusion:** The current study will lay a foundation of knowledge for future research investigations. The abundance of knowledge regarding ethnomedicinal species and their medicinal applications will stimulate further phytochemical and pharmacological research, which could lead to the discovery of potentially significant pharmaceuticals.

## 1 Introduction

Plants have been employed for generations in traditional medicinal therapies by different communities around the world (Vitalini et al., 2013; Nguyen et al., 2019). Herbal medicine is strongly connected to African traditional medicine and is occasionally used in connection with various African societies. It is the most established and extensively utilized primary health self-care system in rural areas, with utilization across all countries and cultures (Idu et al., 2010; Mutie et al., 2020). According to estimates, almost 70% of Kenyans have at some point in their lives used medicinal plants for medical purposes despite the availability of conventional pharmaceuticals (Awiti, 2014; Nankaya et al., 2020). The utilization of plant based medicinal products for the treatment and management of various diseases continues to flourish globally. This is due to a myriad of challenges including decreased efficacy or ineffectiveness of available drugs in treatment of diseases such as malaria, HIV/AIDS, asthma, cancer and diabetes, drug and multidrug resistance as well as the slow pace of discovery of pharmaceuticals with novel modes of action (Mahomoodally, 2013; Mutie et al., 2020). Furthermore, the use of traditional medicinal preparations is always cheap, readily available and easy to prepare and use compared to the contemporary medication which has an associated expense (Antwi-Baffour et al., 2014; Kimutai et al., 2019; Wanjohi et al., 2020a).

Kenya is home to 6,293 native species of vascular plants, of which over 1,200 are valuable medicinally. This diversity makes Kenya a hotspot for ethnomedicinal biodiversity (Melly et al., 2020; Mutie et al., 2020; Mutungi, 2023). Various ethnobotanical researches involving different ethnic groups in Kenya have been done and local communities have accumulated vital traditional knowledge regarding their usage (Jeruto et al., 2008a; Omwenga et al., 2015; Kimutai et al., 2019; Wanjohi et al., 2020b; Mutie et al., 2020; Nankaya et al., 2020). Community members who identify themselves as belonging to a certain culture share experiential knowledge, which serves as the primary representation of ethnobotanical knowledge within a given microsystem. The core body of knowledge is usually associated with the resources that are most easily accessible to the local population; unfortunately this is altered by the entrance of foreign ideas and practices (Ashebo, 2019; Turner et al., 2022). Communities’ cultural identities are defined in part by the indigenous knowledge of how to use and prepare herbs used in traditional medicine. This information also serves as proof of the communities’ historical linkages. In spite of this, the majority of ethnobotanical knowledge about herbal remedies and therapeutic approaches is still not documented (Leonti and Casu, 2013; Zubaidah et al., 2020; Seile et al., 2022). This is exacerbated by the fact that ethnomedicinal knowledge is primarily transmitted orally in Kenyan communities, to limited family members who might not even be eager to acquire the skill (Kipkore et al., 2014; Omwenga et al., 2015).

Ethnomedicine as a science enables the conversion of traditional knowledge from African traditional medicine into knowledge-based research field. This includes understanding the traditional healthcare system and identifying plant-derived substances for therapeutic purposes (Jadid et al., 2020; Tefera and Yihune, 2019; Turner, 2014). Thus, ethnobotanical research that records the uses of plants for medicinal purposes serves as the basis for future phytochemical and pharmacological studies that may form the process of development of innovative treatments and products as well as for the sustainable management of plants (Heinrich, 2000; Jadid et al., 2020). Furthermore, there is a shift in lifestyle globally where there is growing demand for natural organic products. The demand for natural therapeutic products on both domestic and globally has in the recent times increased and sales of products made from medicinal plants such as herbal nutritional supplements, herbal-based cosmetics and herbal healthcare formulations has resulted in considerable economic gains (Ved and Goraya, 2007; Dzoyem et al., 2013; Biagi et al., 2016; Samet and Cikili, 2016). Secondary metabolites from plants can also be valorized for use in the agriculture sector as green and healthy alternatives to chemical pesticides and insecticides because they are generally less toxic, biodegradable, harmless to unintended creatures, and do not affect hosts. Hence the reason plants become the better option as opposed to the synthetics (Naboulsi et al., 2018; Fortunati et al., 2019; Khursheed et al., 2022; Ngegba et al., 2022; Zhao et al., 2022).

While a number of ethnobotanical investigations have been conducted in various locations around Nandi County, including the Aldai sub county (Jeruto et al., 2008b), Tindiret sub county (Kigen et al., 2016), none has been conducted in Mosop Sub-county to gather information about the ethnomedical plants and their applications. Thus, the current study set out to document ethnobotanical knowledge amongst the residents of Mosop Sub County traditionally use. The collected data was qualitatively and quantitatively analyzed using several statistical indices to ascertain the medicinal plant diversity, ethnomedicinal richness as well validate the importance of the cited medicinal species. To get the most out of herbal remedies, researchers should investigate appropriate preparation and dosage formulation techniques as well as traditional medicinal practitioner must work with scientific institutions to aid in the discovery of pharmaceutically active products based on indigenous knowledge.

## 2 Materials and methods

### 2.1 Study area

**FIGURE 1:**
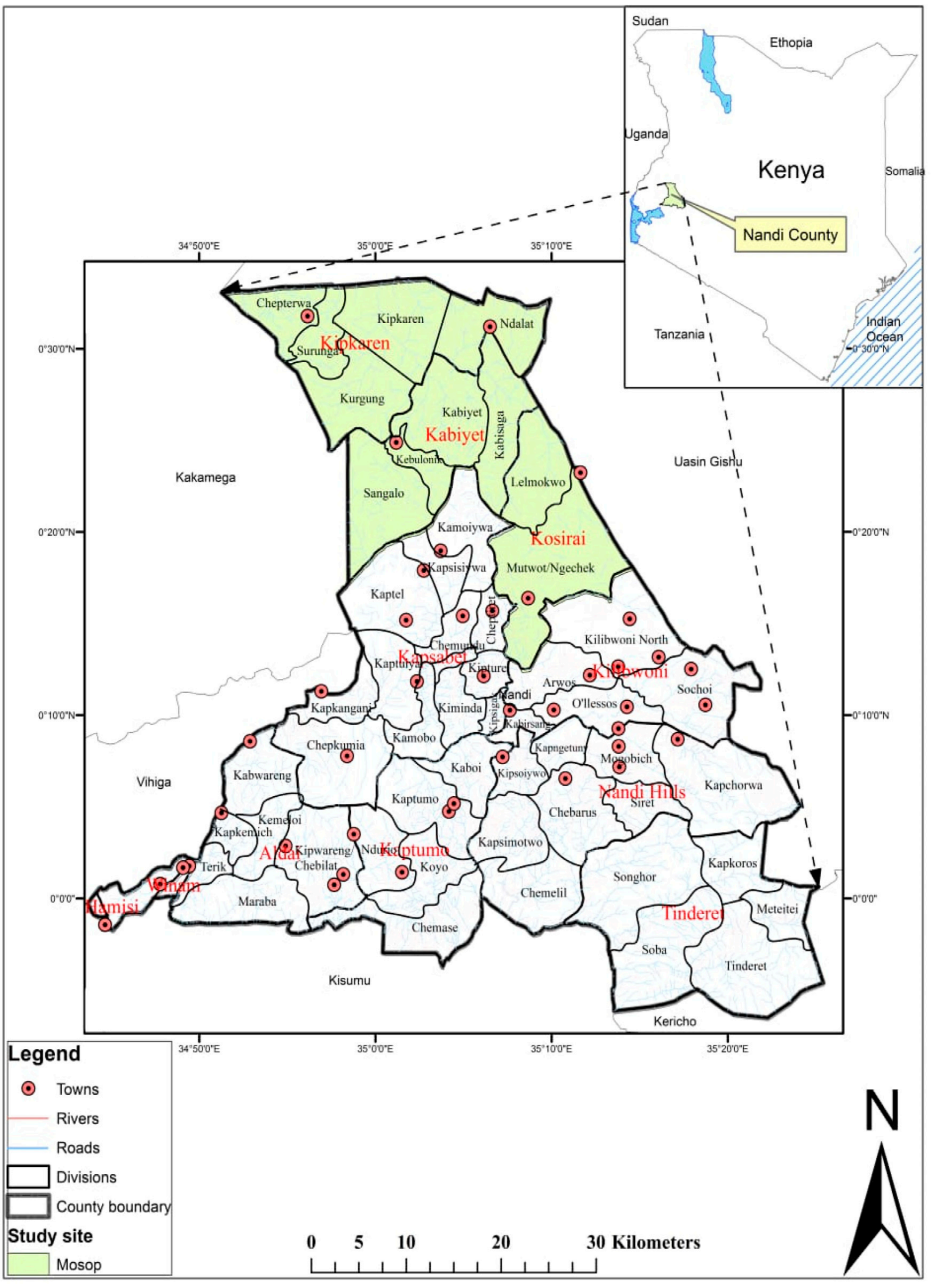
A map showing the study site of Mosop in Nandi county-Kenya.

Mosop Sub County, which covers 602 km^2^, is situated in Kenya’s North Rift Valley to the north of Nandi County. The county is bordered by Uasin Gishu County to the North and East, Kericho County to the South East, Kisumu County to the South, Vihiga County to the South West and Kakamega County to the West (Figure 1). The Equator defines Nandi County from the south, and it stretches north to latitude 0034′N. Both the eastern and western boundaries extend to longitudes of 35025′E and 34045′E, respectively. Rich volcanic soils, chilly rainy environment with temperatures between 15°C and 26°C, and an annual rainfall of 1,200 to 2,000 mm characterize it (http://nandi.co.ke/). This results in a variety of ecological zones, including woods, shrubs, and savannah grassland with swamps along the escarpment, and large areas of the Nandi forest at the top. This region offers a very large topography with a rich biodiversity of various plant species, as well as, in some cases, largely intact native forests along the Teressia, Kaptaroi, and Nandi North forests, which are close to the rich biodiversity of Kakamega forests. The region’s plant biodiversity is exceptionally rich and diverse, and it is frequently said to be a transitional zone between the afro-montane forests of Kenya’s highlands and the lowland forests that traverse Africa from the Zaire basin to western Kenya (Blackett et al., 1994; Girma et al., 2015). Trees, small trees, bushes, shrubs, pteridophytes, creepers, lianas, climbers and succulents are just a few of the many plant species that make up the natural ecosystem of the study site. The traditional medicinal practitioners treat and manage a range of illnesses and diseases affecting the locals by using the many medicinal plants species present in the beautiful distinct habitats in the study site. The study site was chosen because it was the first of its kind in the area and the ease of being able to interact with the respondents in the local dialect.

### 2.2 Research permit and informed consent

The National Commission for Science and Innovation (NACOSTI), Kenya, provided the research permit (NACOSTI/P/21/12175). Before the interview, all respondents provided oral informed consent. The respondents were informed that their information would only be used for scientific research purposes and would not be used for commercial gain. They were also assured that their identities would remain anonymous.

### 2.3 Ethnomedicinal data collection

#### 2.3.1 Inventory-based approach

Guided field trips and an exploratory semi-structured questionnaire approach were used in the study. One or more informants observed the particular plants in their natural habitat during field trips, gathering samples for subsequent botanical identification. Using this technique for gathering data, we collected medicinal plants for identification and preservation, gathered botanical knowledge, and obtained information on their therapeutic applications.

#### 2.3.2 Informant selection

The kind help of local administration personnel allowed for the identification of herbal practitioners prior to the field survey. The traditional medicinal practitioners were chosen based on their willingness to participate in consultative meetings with researchers. This was important since the practitioner needed to know exactly why we were asking for information before they could participate. The interviewees were either active practitioners at the time of the study or former practitioners who had to cut back from the practice owing to aging, commitments or any other personal reasons. Semi-structured open-ended questionnaires were used to record the interviews conducted both at the practitioners’ homesteads and during field sampling with practitioners. All interviews were conducted in the native Nandi dialect and subsequently translated into English. Data gathered included the informant’s name, age, sex, occupation, and educational attainment as well as botanical details including the plant’s local name, source, parts used, and therapeutic uses, as well as preparation and administration techniques. A voucher specimen for the herbarium was collected for every plant species mentioned using standard botanical procedures for further identification and confirmation using the relevant taxonomic keys at Egerton University. Images of all of the mentioned medicinal plants were also taken in order to support the identification processes. The field work was performed between August 2019 and June 2021 where all the traditional practitioners’ responses to structured questionnaires and interviews were used to gather pertinent information.

### 2.4 Data analysis

The data analysis process comprised both qualitative and quantitative approaches. For the purpose of investigating the sociocultural influence on ethnomedicinal data, gender and age of the practitioner were analyzed. Between the quantity of reported medicinal plants species used and gender of the practitioner, a one-way ANOVA analysis was run on Version 25.00 of SPSS. The data was expressed as the mean and standard error of the mean on the quantity of plants species reported by each gender. To further examine any potential relationships between ethnobotanical data and demographics of the practitioners, regression analysis was done to clearly indicate the underlying relationship between the age of the practitioners’ and the quantity of medicinal plant species reported. Moreover, the study tested the validity of the data quantitatively using UC, UV, RFC, FL and ICF.

### 2.5 Quantitative analysis

The formula for calculating the informant consensus factor (ICF) is ICF = Nur-Nt/Nur-1, where Nur denotes the number of use reports from informants for a particular plant-use category and N_t_ denotes the total number of species that are used for that plant use category for all informants (Canales et al., 2005). ICF values fall between 0.00 and 1.00. UV was used to determine the relative importance on uses of the plant species and was calculated from the sum of the informant species use citation for a particular medicinal plant species divided by the total number of informants (N_i_)who reported that species. The UV was calculated according to Hoffman and Gallaher as follows: UV = [∑UV_is_/(N_i_)] (Hoffman and Gallaher, 2007). RFC is an index determined by dividing the number of informants citing the use of a medicinal plant species also known as frequency of citation (FC) by total number of informants in the survey (N) was also calculated using the formula RFC = FC/N (0 < RFC < 1). Where FC = Number of times a particular species was mentioned/total number of times that all species were mentioned × 100 (Tardío and Pardo-de-Santayana, 2008). Fidelity level determines the specific uses of each plant species and preference over other species. It expresses the specificity of disease treated by a reported medicinal plant species. It is calculated by using a formula adopted by Khan et al. (2015). FL= (Ip/Iu) × 100. Where “Ip” is the number of informants who share their knowledge about a given species for the treatment of a specific disease and “Iu” is the total number of all informants who reported all uses about a given plant species (Al-Qura’n, 2009).

## 3 Results

### 3.1 Diversity of respondents’ knowledge and their demographic profiles

**FIGURE 2:**
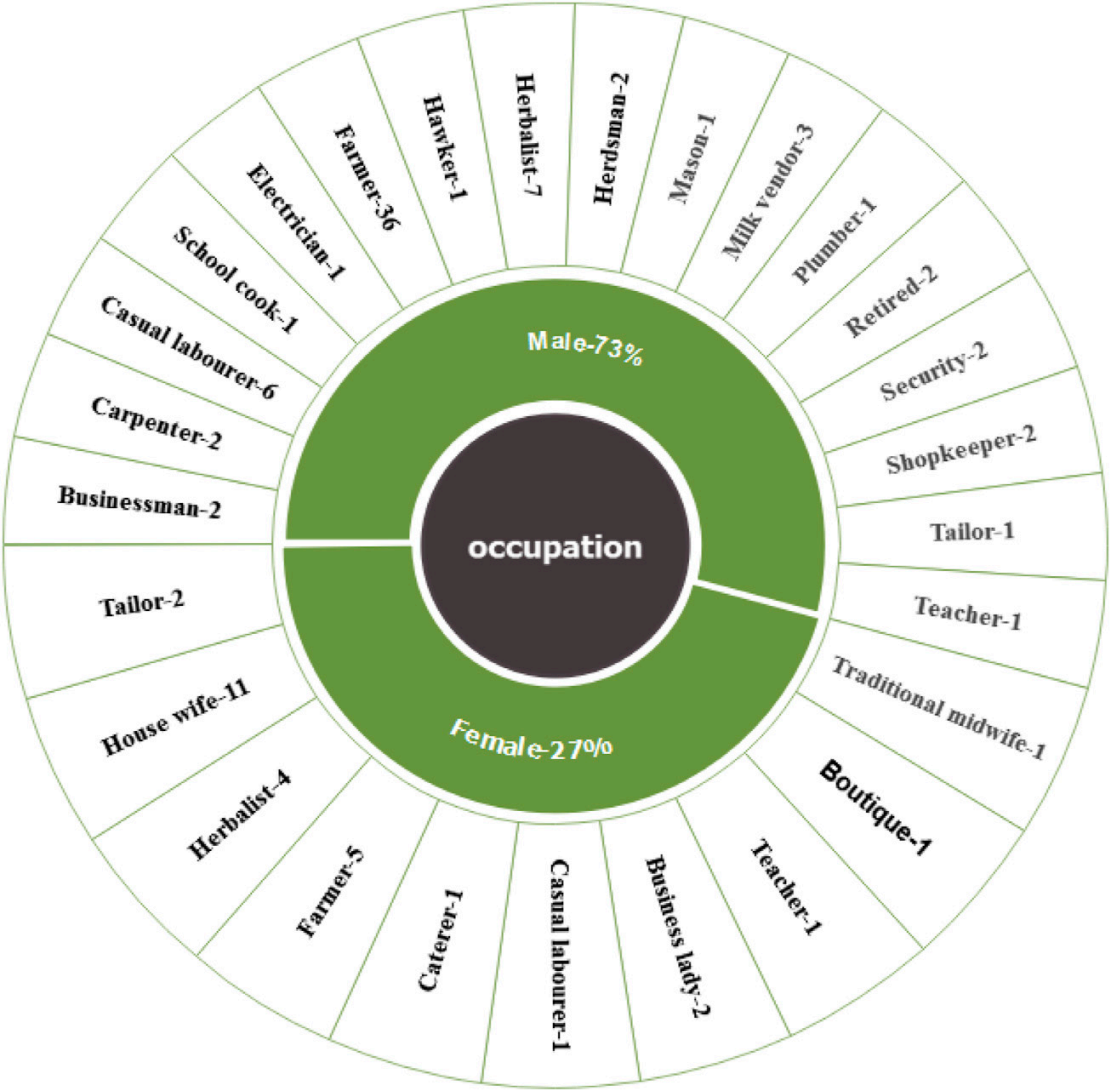
Occupation of the respondents from the study area presented per gender. The inner part of the chart in green shows the gender and the outer part giving the specific occupation per gender.

The study included 102 practitioners as participants. They were engaged in various occupations to support themselves, such as: Carpentry (2), Casual labours (7), Catering (1), Cook in local school (1), Electrical job (1), Farming (41), Hawking (1), Herbalist (11), Herdsman (2), House wives/Home makers (11), Masonry (1), Milk vending (3), Plumbing (1), Retired civil servants (2), Security (2), Shopkeepers (2), Tailoring (3), Teaching (2), Trading (2), traditional midwifery (1), boutique (1) and businesses (4) as presented in Figure 2. The age of the practitioners’ ranged from 35 to 97 years with highest number of practitioners’ being between 45 and 49 years of age. Only seventeen practitioners’ were over 70 years of age. Forty five practitioners’ were between 35 and 49 years old, while forty practitioners’ were between 40 and 69 years old. Among the age groups male practitioners’ were more than the female (Table 1). There were 74 male and 28 female practitioners’ that gave 72.5% and 27.5%, respectively. Most of the practitioners’ had an impressive experience of practicing traditional medicinal therapy with the highest numbers having 10–20 years of experience (37.3%) The numbers declined progressively with the age of the practitioners’ with only 3% of the practitioners’ having the longevity of experience of above 60 years (Table 1). The study observed that older practitioners’ offered rich information that was more profound and detailed since they possessed a greater amount of significant oral tradition knowledge acquired through experience obtained through a lengthy accumulation of generational old traditional medical procedures and therapies as demonstrated by regression analysis done on informant’s age versus the number of medicinal plants species reported by them.

**TABLE 1:** Demographic profile of the respondents.

Variable		No. of informants	%
Gender	Female	29	28.4
	Male	73	71.6
Level of Education	Illiterate	25	24.5
	Non-formal	16	15.7
	Lower Primary	19	18.6
	Upper Primary	19	18.6
	Secondary	19	18.6
	Tertiary	4	3.9
Years of Experience	4–8	6	5.88
	9–13	9	8.82
	14–18	28	27.45
	19–23	11	10.78
	24–28	14	13.73
	29–33	7	6.86
	34–38	4	3.92
	39–43	4	3.92
	44–48	4	3.92
	49–53	8	7.84
	54–58	4	3.92
	64–68	1	0.98
	69–73	2	1.96
	30–40	7	5.88
Age Group	35–39	7	6.86
	40–44	14	13.73
	45–49	24	23.53
	50–54	8	7.84
	55–59	19	18.63
	60–64	7	6.86
	65–69	6	5.88
	70–74	7	6.86
	75–79	7	6.86
	80–84	1	0.98
	95–99	2	1.96
Collaboration with modern medicine	No	30	29.4
	Yes	72	70.6
Any Referrals	No	31	30.4
	Yes	71	69.6
Training for Knowledge Transfer	No	47	46.1
	Yes	55	53.9
	Yes	55	53.9%

**FIGURE 3:**
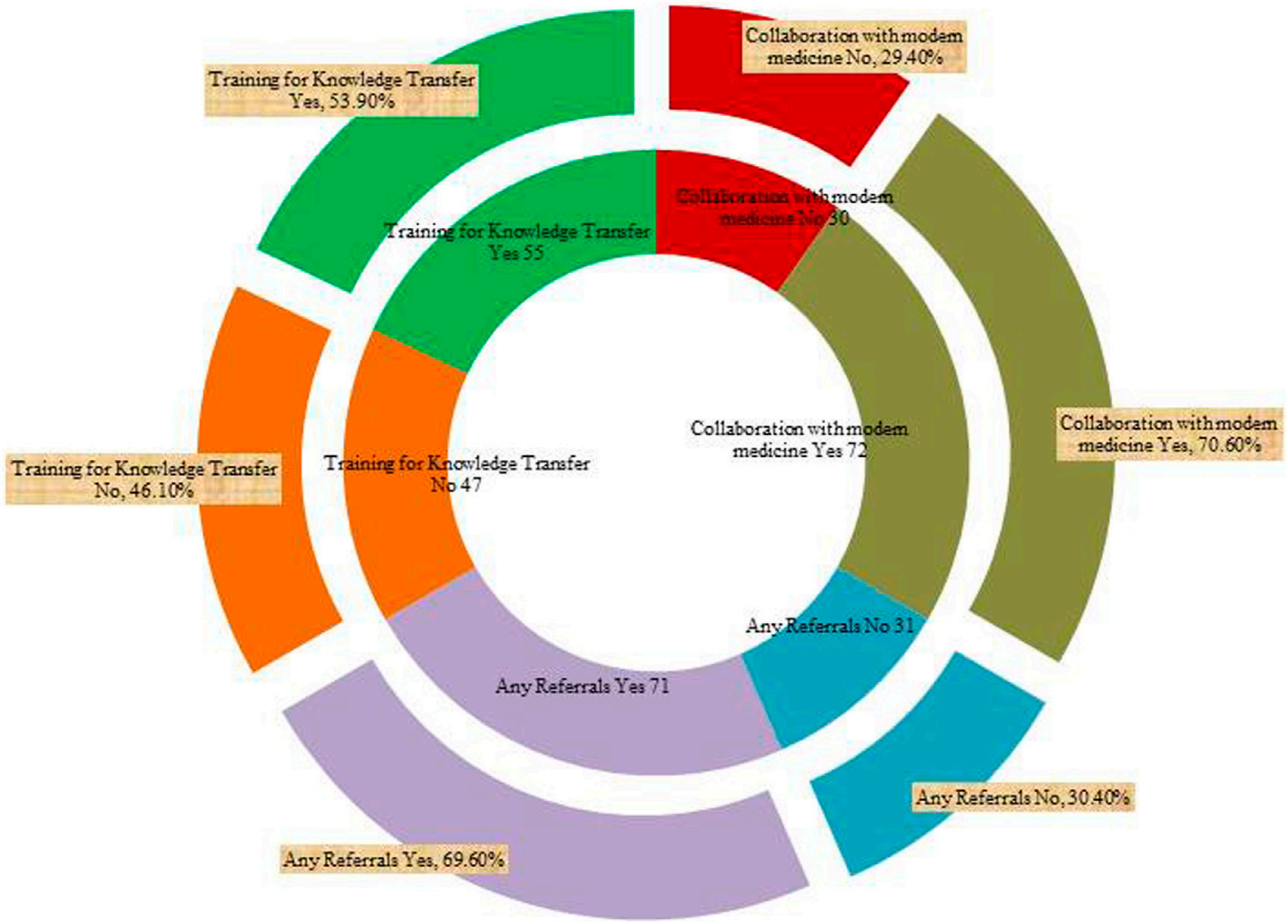
Count of respondents on referrals, training and collaboration with modern medicine. The inner part of the chart gives the numbers on referrals, training and collaboration whereas the outer part of the chart gives their corresponding percentages.

Knowledge transfer was mainly done through apprenticeship and with a family member (65%), apprenticeship and a practitioner (16%), whereas knowledge acquisition via apprenticeship and community elders’ as well as community elders’ was at 8% each. The least mode of acquisition was at 3% through a practitioner. It is apparent from Table 1 that the fundamental sources of knowledge held by the elderly can be attributed to the experiences gathered every day of their lifetime on the traditional usage of medicinal plants and its related skill set. In terms of education, we observed that the majority of the practitioners’ had only basic schooling (24.5% were illiterate), 18.6% had completed both primary and secondary school (sixteen percent each), and only 3.9% had completed their tertiary education (Table 1). The respondents were actively engaged in training for knowledge transfer through a combination of channels at 53.9% as depicted (Figure 3). This comparatively fair rate of knowledge transfer may be due to the fact that most conventional practitioners frequently “ring-fence” their specific area of expertise or pass away without passing on their knowledge and skills to younger generations. Indeed, many practitioners preserved their therapeutic healing secrets; when they pass away without passing on their expertise, that information also disappears with them. It is important to highlight the fact that the younger generation has considerably less knowledge about forests, medicinal plants and their traditional application owing to the fact that they have been cut off from their communities by the current contemporary educational system; and graduates and the learning processes distances them from their villages and their traditions. These result findings further indicate that 70% of the respondents treated various ailments in collaboration with contemporary medicine as well as 71% of the practitioners were making referrals to seek further modern medical attention in cases where the prescribed traditional therapy was not well effective (Figure 3). However, all the practitioners’ had a strong perception that their therapies and remedies were very effective.

**FIGURE 4:**
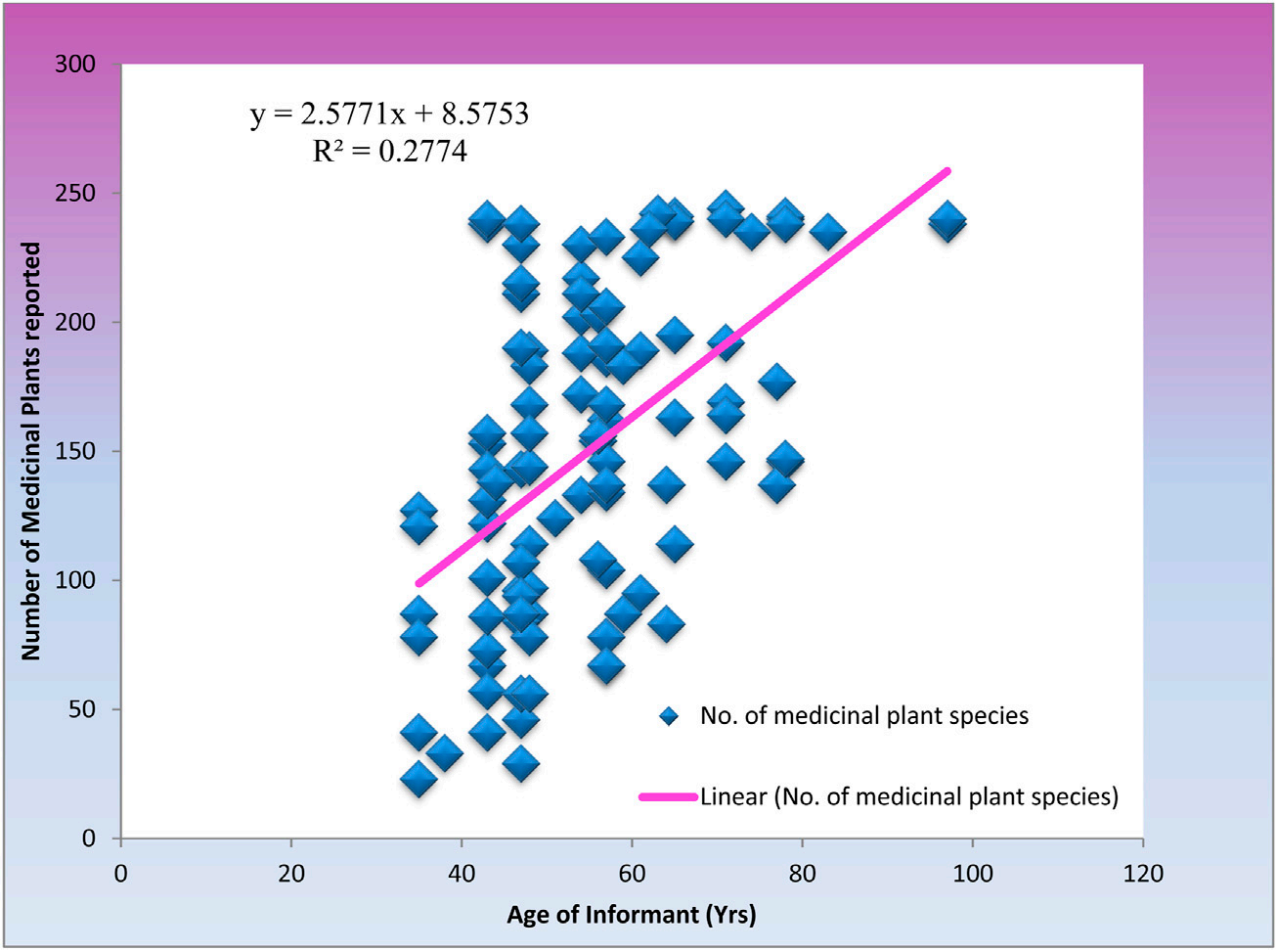
Relationship between number of medicinal plants and age of the respondents.

To find out if there were differences in diversity of knowledge in medicinal plant species used to treat and manage different ailments and diseases in Mosop amongst both genders of the practitioners’, one-way ANOVA demonstrated that the number of medicinal plants species cited by women and men practitioners’ were not significantly different statistically (*p* = 0.844). Despite this, male practitioners reported, on average, a higher medicinal plant species count (152.0401 ± 7.253) than their female counter parts (149.250 ± 12.700) (Mean ± S.E.). This corresponded with the study’s greater participation rate of male practitioners. However, on considering the age of the respondents and their knowledge on medicinal plants, there was a positive correlation (*R* ^2^ = 0. 277, *p* < 0.05) as in Figure 4. As a result, older individuals typically had greater knowledge of medicinal plants than younger practitioners’.

### 3.2 Diversity of medicinal plant species used

**FIGURE 5:**
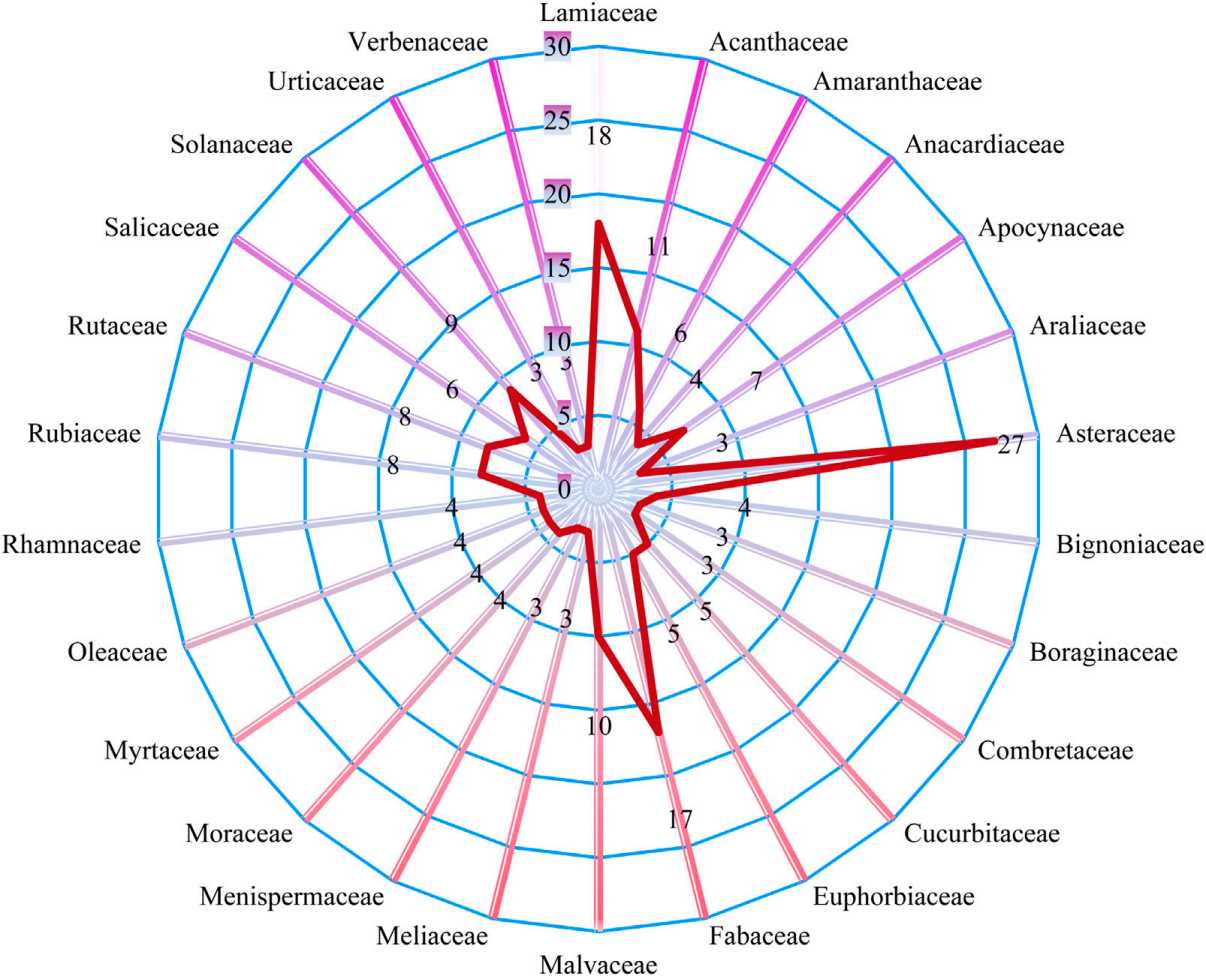
Diversity of medicinal plant families in the study area with ≥3 species.

**FIGURE 6:**
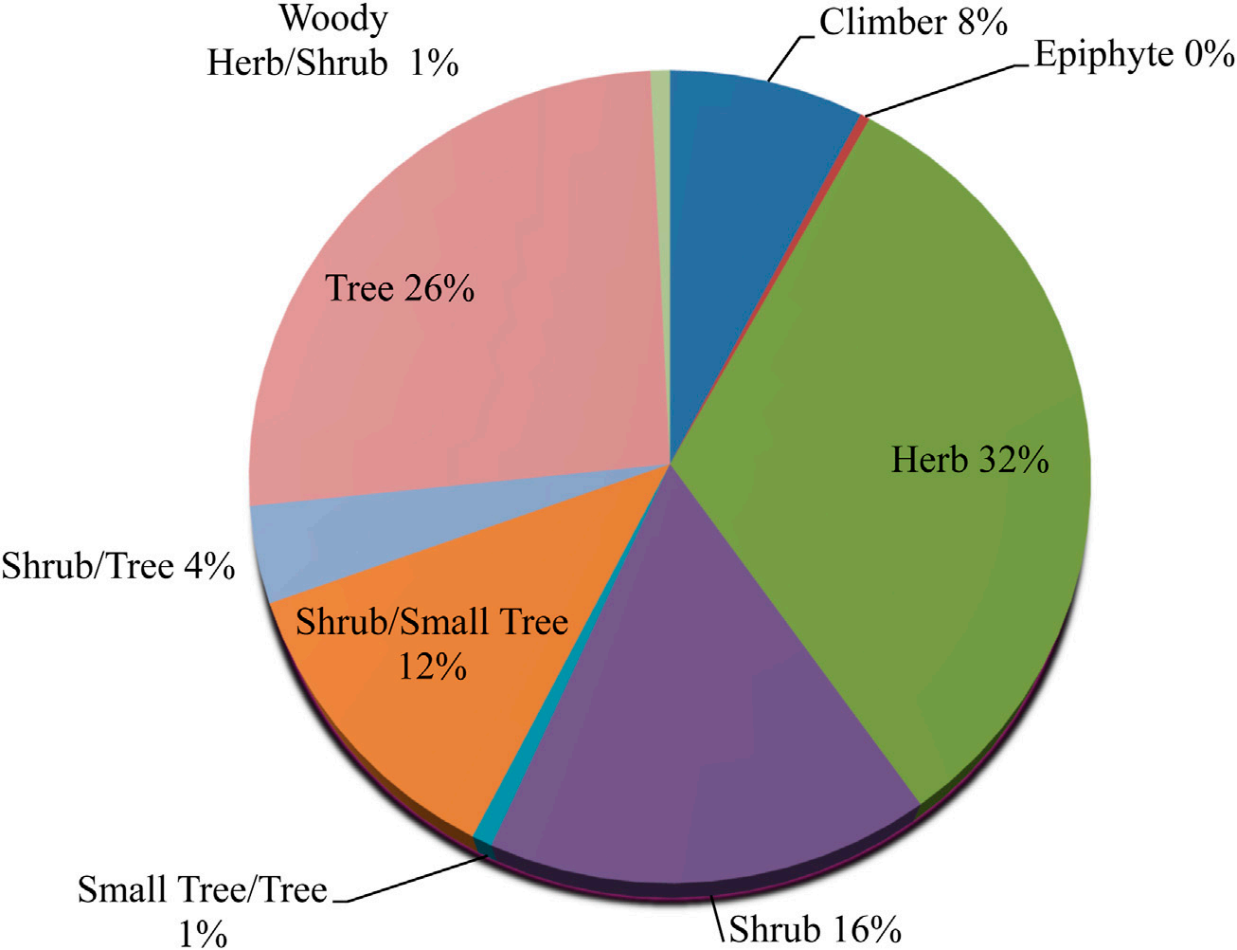
Habit of medicinal plants reported in the current study.

The most dominant families in terms of the highest reported medicinal plant species were Asteraceae (28 species), followed by Lamiaceae (19 species), Fabaceae (19 species) and Acanthaceae (12 species) as in Figure 5. 34 families were represented by only a single medicinal plant species. We identified 253 species divided into 74 families of medicinal plants used in the treatment and management of different ailments and diseases in Mosop Sub-County (Supplementary Table S1). The degree of ethnobotanical richness in the study area is directly accredited to its diverse flora. In this study most of the medicinal plant species were herbs (32%), followed by trees (26%), shrub/small trees (12%), shrub/tree at 12%, climbers (8%) and others (2%) as in Figure 6. The only epiphyte was *Phragmanthera usuiensis* (Oliver) M.G.Gilbert. Medicinal plants status was assessed using the criteria from the International Union for Conservation of Nature (IUCN) (https://www.iucnredlist.org). The study showed that *Warburgia ugandensis* Sprague as critically endangered (CE), *Tiliacora kenyensis* Troupin as endangered (EN); *Polyscias kikuyuensis* Summerh. as near threatened (NT); *Solanum betaceum* Cav. and *Carica papaya* L. as data deficient (DD); *Zanthoxylum chevalieri* Engl., *Justicia flava* Vahl and *Prunus africana* (Hook.f.) Kalkman were vulnerable (VU). The remaining medicinal plant species assessed as of least concern (LC) and not evaluated (NE) were 101 and 144, respectively. *Juniperus procera* Hochst. ex Endl., *Justicia flava* Vahl, *Microglossa pyrifolia* (Lam.) Kuntze, *Carica papaya* L., *Ehretia cymosa* Thonn., *Olea welwitschii* Gilg & G.Schellenb and *Protea gaguedi* J.F.Gmel. were assessed as LC with an alert that their numbers were decreasing.

### 3.3 Plant parts used and methods of preparation

**FIGURE 7:**
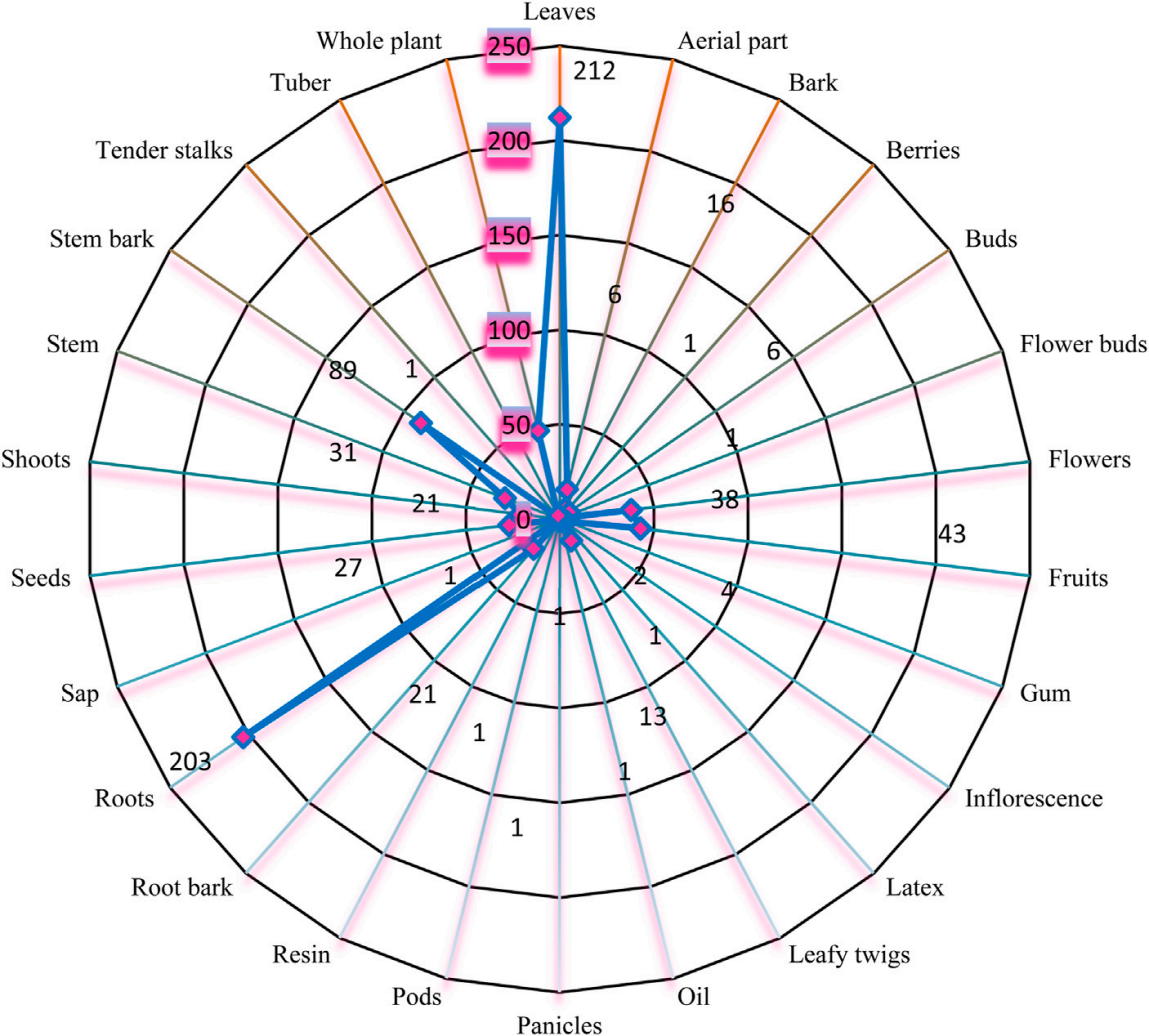
Plant parts used for the traditional medicines in the current study.

**FIGURE 8:**
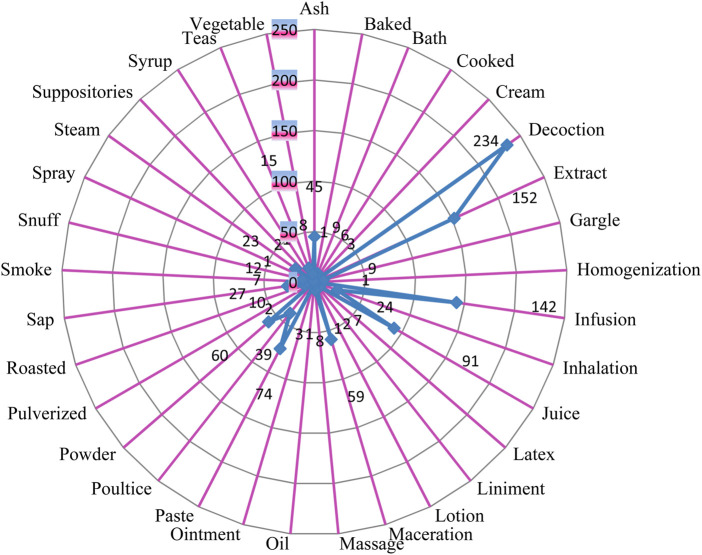
Traditional administration forms of medicinal plants in the study area.

The most commonly used plant parts were leaves (27%), followed by the roots (26%), stem bark (11%), whole plant (6%), stems, shoots, seeds and root bark 3% each; aerial part and leafy twigs (2%) and 1% were the gum, tuber inflorescence, tender stalks, sap, resin, pods, panicles, oil, latex, flower buds and berries as in Figure 7. The leaves were the portion that was most frequently used. The roots and the stem bark were also the most preferred parts of the medicinal plant parts to be used in preparing the herbal remedies. Tender stalks, flowers and the fruits with 48, 38 and 43 entries were also amongst the most popular parts of the medicinal plant sort by the respondents. The most used preparation method was the decoction (21%), followed by extract (14%), Infusion (13%) and the least medicinal preparation were in form of suppositories, lotions, sprays and syrups (Figure 8). Due to its ease of use and preparation, the decoction method was more widely used. While using the decoctions, some additives, such as honey, goat soup, ghee or milk were added to change their flavour. Extracts preparations were obtained adding little water to macerated/pounded/pulverized medicinal plant material with or without filtration. Macerated/pounded/pulverized/powdered medicinal plant material were soaked in hot or cold water and filtered to prepare infusions. The choice of infusion method depended on the desired end product and the properties of the plant material being infused and more so the ailment it is supposed to treat or manage. Closely related to the extract and the infusions were the juice preparations where by the liquid was extracted from medicinal plant material without the addition of any solvent or solution to aid the process. Juices were made exclusively from the raw natural phytochemical constituents of the specific medicinal plant species; no additional ingredients were added.

### 3.4 Quantitative analyses

The present work was the first ever study to record quantitative data of the medicinal plants of Mosop Sub County in Nandi County, including relative frequency of citation, use value, use citation, informant consensus factor and relative frequency of citation. The UV of each medicinal plant was determined to assess the commonness in use of each plant in the study area of Mosop. The UV of the encountered medicinal plant species ranged from 0.53 to 36.54 (Supplementary Table S2). The important medicinal plant species with high use value were *Vachellia nilotica* (L.) *P. J. H. Hurter & Mabb., Vangueria infausta* Burch.*, Zanthoxylum asiaticum* (L.) Appelhans, Groppo & J.Wen*, Clutia abysinicca Jaub*.*& Spach*; and *Croton macrostachyus* Del. While a low value may indicate that a plant is infrequently used, it does not always indicate inefficiency. The lower UV was caused by respondents’ lack of knowledge about the plant species. *Cyathula cylindrica* Moq (1.89), *Scepocarpus hypselodendron* (Hochst. ex A.Rich.) T.Wells & A.K.Monro (1.57) and *Tiliacora triandra* Troupin (0.53) had the low use values.

The range of RFC was from 0.53 to 1.00. The highest value of RFC (1.00) was found in *Lantana trifolia* L*, Ziziphus mucronata* Wild*, Tragia brevipes* Pax*, Zanthoxylum chalybeum* Engl., *Erythrococca Bongensis* Pax, *Grewia similis* K. Schum*, Entada abyssinica* Steudel ex A.Rich. and *Warburgia ugandensis* Sprague (Supplementary Table S2). These result findings shows how frequently these particular plant species are used in the study area to prepare remedies for different ailments. The International Classification of Diseases (ICD-10) version of 2016 aided the clustering of various diseases and disorders into several categories. The entire spectrum of illnesses, ailments, traumas, and other connected medical problems is represented by ICD-10. Based on the documented use of medicinal plant species in the study, 18 categories were identified. ICF values range from 0.00 to 1.00. Higher ICF values are indicative of the fact that only a handful of medicinal plant species are recognized to be given by a higher number of practitioners, whereas lower values show that practitioners are at odds over which species to use in treating or managing a specific ailment or disease. The 18 diseases categories demonstrate the wide range of applications of medicinal plant species from the study area. The ICF values for each ailment category ranged from 0.13 to 0.78 (Table 2).

**TABLE 2:** Informant consensus factor (ICF) by category of ailment within the present study.

Disease category	Specific use report	Use report	Plants	ICF
Certain infectious and parasitic diseases	TB, tetanus, typhoid, intestinal parasites, abscesses, malaria, amoebiasis, amoebic dysentery, athlete’s foot rot, ringworms, Bell’s palsy, diarrhea, body infections, boils, brain infections, brucellosis, candidiasis, chicken pox, childhood diseases, cholera, antiseptic, dysentery, ectoparasites, ENT infections, fungal diseases, hepatitis, herpes, leprosy, mastitis, measles, meningitis, mumps, nasopharyngeal infections, pertussis, pneumonia, polio symptoms, whooping coughs, scabies, sepsis, sleeping sickness, sores and small pox	1,010	225	0.778
Conditions related to sexual health	Infertility, (STIs), amenorrhea, dysmenorrhoea, menstrual issues, gonorrhea, gynecological disorders, syphilis, venereal diseases, sterility, vaginal discharge, erectile dysfunction, impotence, premature ejaculation, orgasm, fertility issues, leucorrhoea, spermatorrhoea, frequent night discharge, breast issues, womb/uterine issues, pain during intercourse, sexual stamina, fibroids, vaginitis and endometriosis	409	141	0.657
Diseases of the blood and blood-forming organs and certain disorders involving the immune mechanism	Fever, inflammations, anemia, strengthen the spleen, splenomegaly, boost immunity, pain ever, blood disorders, bone marrow diseases, sickle cell diseases, allergies, spleen disorders, fever and blood clot formation	373	168	0.551
Diseases of the circulatory system	Stop gum-bleeding, high blood pressure, heart problems; stop bleeding, hemorrhoids, heart condition, blood purifier, abnormal uterine bleeding, detoxification, hemorrhage, abdominal bleeding, blood/circulatory diseases, disturbances in blood circulation, varicose veins, haemolysis, nose-bleeding, heart diseases, cardiovascular complications, pulmonary conditions, piles, rapid heartbeat, stopping heavy bleeding, perspiring feet, promote blood circulation, blood coagulation on fresh cuts, stroke, stop blood flow, blood vessel constriction, fainting, blood clotting and pulmonary ailments	263	132	0.500
Diseases of the digestive system	Relieve indigestion, stomach troubles, constipation, peptic ulcers, toothache, evacuate the stomach, dental care, gastrointestinal disorder, feeding disorder, ulcers, loss of appetite, irritable bowel complaints, cholesterol, passing gas, oral care, strengthen gum, strengthen teeth, sores, hernia, anal infections, poor digestion of lipids, nausea, laxative, nausea, enema, throat infection, mouth wash, itchy anus, heartburn, hyperacidity, stitches, soften stool, increase taste, gastritis, opening up bowels afterbirth, bloody stool, stomachic, carminative, red sores, excessive saliva production, irritable bowel, dental caries, gargle, gastroenteritis, bad odours/breath, gall bladder, reduce alcohol absorption, rectum prolapsed, tooth decay, digestive system disorders, laxative, mouth, bowel disorders, gall bladder stones, gastritis and hiccups	942	217	0.770
Diseases of the ear and mastoid process	Earaches, ear infections, ear-inflammation/pus and ear-drops	32	28	0.129
Diseases of the eye and adnexa	Eye infections, inflamed eye/lids, sore eyes, eye diseases/conjunctivitis, itchy eyes, eye lotion, eyewash, hurting eyes, cataracts, eyelids swelling/trachoma and eye complaints	80	57	0.291
Diseases of the genitourinary system	UTIs, kidney, UTIs, kidney problems, urinary problems, erectile dysfunction, kidney pains, boost urination, swollen prostate gland, kidney diseases, bladder infections, urogenital disorders, kidney pain, kidney problems, urinary system, swollen male genitalia, genital ulceration, renal/kidney stones, urethra infections/disorders, urethral leaks and urination issues	146	117	0.200
Diseases of the musculoskeletal system and connective tissue	Rheumatism, swellings, oedema, bone setting, arthritis, backache, cramps, body massage, paralysis, toning tissues of the buttocks, aching joints/muscles, convulsions, gout, joint pains, gout, general body aches, swollen feet/elephantiasis, muscle spasms, cramps, muscle pain, paralysis, convulsions, stiffness in the joints, muscle wastage, bone defects, burning sensation, irritation, relax muscles during giving birth, sharp aching radiating/burning/stabbing, bone strength, lower back aliments, bone alignment, stitches, cysts, promote walking in infants, internal body ailments, spinal complaints and muscle relaxation	434	164	0.624
Diseases of the nervous system	Epilepsy, migraines, aphrodisiac, headaches, rabies, stimulant, antidepressant, benumbing/sedative agent, anxiety, anesthesia, convulsion, CNS disorders, nervous system, bedwetting, relieve anxiety, thirst, epileptic seizures, libido, virility, calming down nervous activity, snake bite, convalescence, promote sweating, numbness, neck stiffness, seizures, aching teeth, sleep disorders, bites/rabies, boost memory, improve mood, Bell’s palsy “Kiptumarit”, nervous debility, coolant and insomnia	494	165	0.667
Diseases of the respiratory system	Common colds, chest pain, sore throat, tonsillitis, colds, coughs, chest diseases, respiratory problems, flu, asthma, chest complaints, respiratory tract disorders/diseases, sinusitis, bronchitis, bronchial infections, expectorant, breathing difficulty, chest congestion, throat infections, influenza, lung diseases, running nose, soreness, nose infections, hiccups, frequent sneezing, bronchial affections, wheezing, phlegm accumulation, epiglottis issues, hoarseness, voice box infection, lung fluid retention and blocked nostrils	844	189	0.777
Diseases of the skin and subcutaneous tissue	Skin infections, itching, eczema, skin rashes, sunburn, loss of skin colour in patches, warts, sores, acne, wash hairs, abscesses, skin eruptions, depigmentation due to total loss of melanin, dressing of ulcers, sooth and moisturize the skin, skin diseases, burning sensation, septic swellings in the skin, improve skin tone, skin irritation, antiseptic, body massage, ulcerations, skin lesions, inflamed surfaces, pimples, dermatitis, chickenpox irritation, promote pus release, skin afflictions, skin blisters, abrasions, liniment, furuncles, dandruff, odours, body cracks, baldness, scalp infections, freckles, skin discolorations, opportunistic infections, cysts and parasitic skin diseases	339	128	0.624
Endocrine, nutritional and metabolic diseases	Jaundice, gland disorders, hepatic issues, diabetes, obesity, liver diseases, hardening tissues of the liver and spleen, relief off hunger pangs, high blood pressure, chronic liver complications due to alcoholism, strengthen pancreases, liver functions, bile flow, cholesterol/fat complications, kwashiorkor, liver disorders, low blood sugar, weight-loss, cholesterol, health improvement, enlarged liver, hormonal disorders and scurvy	185	105	0.435
Injury, poisoning and certain other consequences of external causes	Bowel poisoning, induce vomiting, wounds, burns, bruises, bite, minor cuts, insect bites, caterpillar bites, antidote, wound dressing, nettle stings, body injuries, sprains, scorpion bite, injury, food poisoning, acute soft tissue injury, fractures, injury massage, scratches, animal poison, gangrene, caterpillar attack and dislocation	386	175	0.548
Mental and behavioural disorders	Mental disorder, dizziness, restore general body strength, boost general health, colic, stress, fatigue, chronic weakness, hysteria, mental disturbances, mental illness, psychiatric problems, tonic, eating disorders, discomfort/illness, release tension, hangover, longevity of life, improve mood, psychoses, insanity, renew memory, unwellness and shock	130	86	0.341
Neoplasms	Cancers, enlarged glands, tumours, cancerous wounds “seriat,” leukemia, cyst formation, cancer “Chepserkechet” and malignant ulcers	140	94	0.331
Pregnancy, childbirth and the puerperium	Prevent abortion, lactation, labour, womb cleansing, uterine issues, expulsion of the placenta, abortion, pregnancy weakness, toxemia, miscarriages, uterine contractions, clearing the conceptus, umbilical cord disinfectant, abdominal pain in pregnancy, prevent pregnancy, oedema in pregnancy, bellybutton/navel, delayed pregnancy, after birth pains, postpartum uterine care, uterine infections, “women’s” complaints, induce abortion, vaginal lacerations after birth, facilitating delivery, labour pain alleviation, reduce morning sickness, induces abortion, pre-natal care, contraception, quieten a foetus, maintain pregnancy to term, pregnancy pain, tone the vagina afterbirth and child birth	164	84	0.491
Others	Obstructions, synergistic for many ailments	2	0	0

A relative informant consensus regarding the usage of medicinal plants species to treat or manage an illness or diseases is indicated by an ICF score greater than 0.5. On the other hand, ICF values less than 0.5 suggested that practitioners’ exchange little information about medicinal plant species therapies. The highest ICF (0.778) was reported for the category of certain infectious and parasitic diseases with 225 medicinal plant species and 1,010 UCs, followed secondly by the diseases of the respiratory system category (0.777) with 189 plant species and 884 UCs. In third place was digestive system diseases category (0.770), with 217 medicinal plant species and 942 UCs. Our ICFs values shows that the practitioners’ have a strong consistent know-how when it comes to choosing and applying medicinal plants species to treat and manage illnesses and diseases that gave high ICF values. The highest ICF (0.778) reported for the category of certain infectious and parasitic diseases was dominantly due to a high citation of respiratory associated conditions. This could be attributed to the climate in the research area, as well as the fact that relative clinical indications are more prevalent for locals seeking the services of the practitioners’ to recognize easily.

Our results showed that FL values ranged from 10% to 93% (Table 3). Senegalia senegal (L.) Britton demonstrated the highest FL for throat infection (93%), followed by *Gynandropsis gynandra* (L.) Briq. (92.86%) and *Baccharoides lasiopus* (O.Hoffm.) H. Rob. (91.67%) for relieving body pain. Low percentage of FL belonged to *Hibiscus diversifolius* Jacq used in nerve diseases (10.10%) and heart diseases and fluid retention at 11.11%. All low FLs in this study were conditions managed using *Hibiscus diversifolius* Jacq and *Triumfetta macrophylla* K. Schum (10.10%–21.21%) shared the same local name “Meswot” belonging to the family Malvaceae. The low fidelity levels observed could be the due to the limited number of species from this family used to treat and manage different ailments and diseases. Additionally, it may suggest that Mosop residents in Nandi County are not well-informed about the utilization of this medicinal shrub. Medicinal plant species with high FL values can be utilized as a basis of further phytochemical and pharmacological evaluation to identify significant bioactive chemicals compounds. This emphasizes how vital it is to use an ethnomedicinal focused method of bio prospecting in order to identify and find new phytocompounds or plant-based products that may be used in a variety of disciplines.

**TABLE 3:** Fidelity levels of medicinal plant species (≥70%).

Medicinal plant	Specific disease	Ip	Iu	FL	References
*Phytolacca dodecandra* L’Hér.	Syphilis	78	99	78.79	Anthoney et al. (2015), Matebie et al. (2019), Feyisa et al. (2022)
Terminalia schimperiana Hochst. ex Engl. & Diels	Cough	79	100	79.00	Burkill (1995), Adebayo and Ishola (2009), Fyhrquist et al. (2014)
*Eucalyptus globulus* Labill	Asthma	81	101	80.20	Silva et al. (2003), Vigo et al. (2004), Mahishi et al. (2005), Manikandan (2005), Mota et al. (2015), Ray et al. (2015), Akhtar et al. (2016), Tomen et al. (2017), Nile and Keum (2018), Göger et al. (2020), Kubera Sampath Kumar et al. (2021), Arooj et al. (2023), Masé and Herbs (2023)
	Inflammations	83	101	82.18	
	Wounds	83	101	82.18	
Nicoteba betonica (L.) Lindau	Vomiting	80	98	81.63	Rao et al. (2006), Jeruto et al. (2008), Bbosa et al. (2013), Mukungu et al. (2016), Pacifica et al. (2018), Prathibha and Jayaramu (2019)
	Constipation	78	98	79.59	
	Pain	77	98	78.57	
	Malaria	84	98	85.71	
	Headache	77	98	78.57	
*Vachellia nilotica* subsp. *tomentosa* (Benth.) Kyal. & Boatwr.	Bronchitis	85	100	85.00	Agunu et al. (2005), Misar et al. (2007), Sanni et al. (2010), Singh et al. (2010), Ali et al. (2012), Farzana and Al Tharique (2014), Hussain et al. (2016), Manzo et al. (2017), Jaiswal et al. (2023)
	Diarrhoea	80	100	80.00	
	Dysentery	80	100	80.00	Kuria et al. (2001), Kariuki (2015), Yacob et al. (2016), Nardos and Makonnen (2017), Kigen (2019)
Ajuga integrifolia Buch.-Ham. ex D. Don	Malaria	85	100	85.00	
*Bridelia micrantha* (Hochst.) Baill.	General health	80	98	81.63	Omeh et al. (2014), Kathare et al. (2021a), Asumang et al. (2021)
*Tragia brevipes* Pax	Headache	85	102	83.33	Chepng’etich et al. (2018), Migabo et al. (2023)
*Urena lobata* L.	Asthma	80	100	80.00	Islam et al. (2015), Gudu et al. (2023)
*Barleria grandicalyx* Lindau	Wounds	81	98	82.65	Rattan (2023)
	Cough	77	98	78.57	
Senegalia senegal (L.) Britton	Throat	93	100	93.00	Mans et al. (2022)
	Discomfort	79	100	79.00	
*Solanecio mannii* (Hoof.f.) C.Jeffrey	Rheumatism	81	96	84.38	Muganga et al. (2010)
*Tarenna graveolens* (S.Moore) Bremek.	Rheumatism	78	97	80.41	Oloro et al. (2016)
*Micromeria* Benth.	Headache	75	91	82.42	Matasyoh et al. (2007), Okach et al. (2013)
*Micromeria biflora* (Buch. - Ham. ex D.Don) Benth.	Headache	76	91	83.52	Bouriah et al. (2021), Rauf et al. (2021), Aljohani et al. (2022)
*Aspilia pluriseta* Schweinf. ex Engl.	Wounds	85	101	84.16	Kuria (2014), Kuri et al. (2015), Njeru and Muema (2020), Njeru and Muema (2021)
*Tylosema fassoglensis* (Kotschy ex Schweinf.) Torre & Hillc	Pneumonia	80	99	80.81	Adongo et al. (2012)
*Thunbergia alata* Bojer ex Sims	Diarrhea	80	98	81.63	Cho et al. (2016), Mugaba (2023)
	Fever	77	98	78.57	
*Gymnosporia undata* (Thunb.) Szyszyl.	Syphilis	77	98	78.57	Mokoka et al. (2013)
*Hoslundia oppositae* Vahl	Wounds	79	99	79.80	An et al. (2008), Onwuka et al. (2016), Namuga et al. (2022)
*Grewia similis* K. Schum	Cold	78	100	78.00	Muithya (2010)
	Cough	83	100	83.00	
*Lippia javanica* Spreng.	Indigestion	85	102	79.21	Nkomo (2010), Nkomo et al. (2011)
*Leonotis nepetifolia* (L.) R.Br.	Cough	80	101	83.67	Parra-Delgado et al. (2004), Pushpan et al. (2017), Giang et al. (2021)
	Burns	82	98	79.59	
	Back pain	78	98	79.59	
	Joint pain	78	98	86.73	
*Olea europaea* subsp. *cuspidata* (Wall. & G. Don) Cif.	Sore throat	85	98	78.57	Masoko and Makgapeetja (2015)
*Zanthoxylum chalybeum Engl.*	Fever	77	98	81.37	Müller-Jakic et al. (1993), Musila et al. (2013), Bbosa (2014), Ngugi (2014), Njenga et al. (2016)
	Malaria	83	102	81.37	
*Coleus barbatus* (Andrews) Benth. ex G. Don	Pain	83	102	85.15	Falé et al. (2011), Ezeonwumelu et al. (2019)
	Coughs	86	101	79.21	
*Cleome gynandra* L.	Bites	80	101	78.57	del Carmen Juárez-Vázquez and Jiménez-Arellanes (2019), Narendhirakannan et al. (2007), Sivakumar et al. (2010)
	Chest pain	77	98	79.59	
	Pain	78	98	92.86	
*Schrebera alata* (Hochst.) Welw.	Bleeding	91	98	88.00	—
*Dovyalis abyssinica* (A.Rich.) Warb.	Stomachache	88	100	80.81	Kipngeno (2019), Legesse et al. (2019), Emam et al. (2021)
	Cancer	80	99	79.80	
*Entada africana* Guill. & Perr.	Stomachache	79	99	78.22	Akindele et al. (2016), Hassan et al. (2018), Yusuf and Abdullahi (2019), Adewole et al. (2022)
	Dysentery	79	101	80.20	
*Entada abyssinica* Steudel ex A.Rich.	Coughs	81	101	78.43	Olajide et al. (2005), Mariita et al. (2010), Teke et al. (2011), Roger et al. (2015)
	Fever	77	97	78.43	
*Combretum pisoniiflorum* (Klotzsch) Engl.	Snake Bite	80	102	82.65	Molander et al. (2014), Miaffo et al. (2015), Hamza et al. (2021)
*Combretum collinum* Fresen	Indigestion	80	102	78.13	Njume et al. (2011), Marquardt et al. (2020)
*Lactuca macrophylla* (Willd.) A.Gray	Sores	81	98	84.85	Waiganjo et al. (2016)
*Vangueria infausta* Burch.	Pneumonia	75	96	79.21	de Boer et al. (2005), Gwatidzo et al. (2018), Addo-Mensah and Holland (2022)
	Fever	84	99	78.22	
*Gymnosporia heterophylla* (Eckl. & Zeyh.) Loes	Wounds	79	101	78.57	Da Silva et al. (2011), Umar et al. (2019), Omara (2020), Tyavambiza et al. (2021)
	Pain	60	74	90.82	
*Croton dichogamus* Pax.	Fever	96	99	80.00	Aldhaher et al. (2016), Matara et al. (2021)
	Stomachache	77	98	81.00	
	Malaria	89	98	80.00	
*Syzygium guineense* (Willd.) DC	Pain	81	100	84.85	Tanko et al. (2008), IOR et al. (2012), Mollika et al. (2014)
*Carissa spinarum* L.	Headache	80	100	78.79	Woode et al. (2007), Hassan et al. (2010), Maina (2015), Fanta Yadang et al. (2019), Dawa et al. (2021), Jepkorir et al. (2023)
	Complaints	79	98	83.84	
	Rheumatism	84	99	84.85	
	Pain	78	99	80.81	
*Vachellia sieberana* var*.* Woodii (Burtt Davy) Kyal. & Boatwr.	Oedema	83	99	86.00	Wuerger et al. (2009), Würger (2010), Jurbe et al. (2015), Ngaffo et al. (2022)
	Stomachache	84	99	84.00	
	Diarrhoea	80	99	82.00	
	Pain	86	100	91.00	
	Inflammation	84	100	91.00	
*Tarchonanthus camphoratus* L.	Bronchitis	82	100	81.19	Ali et al. (2013), Wetungu Martin et al. (2014)
*Flacourtia indica* (Burm.f.) Merr.	Colic	91	100	78.22	Tyagi et al. (2011), Chun and Kundu (2013), Islam et al. (2023)
	Fever	91	100	87.13	
	Cough	82	101	80.20	
*Psidium guajava* L.	Diarrhoea	79	101	78.22	Lozoya et al. (2002), Dutta and Das (2010), Lobo and Ballal (2011), El-Ahmady et al. (2013), Siani et al. (2013), Jang et al. (2014), Sekhar et al. (2014), Raja and Sundar (2016), Ala et al. (2019), Koriem et al. (2019), Hirudkar et al. (2020), Lu et al. (2020)
	Inflammation	88	101	85.15	
	Pain	81	101	81.19	
*Croton megalocarpus Hutch.*	Malaria	82	101	85.00	Gichui (2016), Danyaal (2020)
	Fever	79	97	81.00	
*Physalis peruviana* L.	Worms	75	96	81.19	Maobe et al. (2013), Kamau et al. (2020), Kathare et al. (2021b)
	Malaria	85	100	83.17	
*Markhamia lutea* (Benth.) K. Schum	Diarrhea	81	100	79.80	Abdullah et al. (2011), Lacroix et al. (2011), Ngoufack Azanze et al. (2023)
	Pain	82	101	86.87	
*Ficus sur* Forssk.	Gonorrhoea	84	101	81.19	Omodamiro et al. (2021), Wakeel et al. (2021), Owolabi et al. (2022)
	Pain	79	99	84.16	
*Protea gaguedi* J.F.Gmel.	Diarrhoea	86	99	81.72	—
*Ehretia cymosa* Thonn.	Wounds	82	101	78.00	Nicholas (2017), Adeleye et al. (2021)
	Epilepsy	85	101	82.00	
*Azadirachta indica* A.Juss.	Ulcers	76	93	79.00	Bandyopadhyay et al. (2004), Dorababu et al. (2004), Raji et al. (2004), Ofusori et al. (2010), Kumar et al. (2011), Mohapatra et al. (2012), Bhajoni et al. (2016), Odo (2016), Narapusetty et al. (2017), Abioye et al. (2019), Farzana et al. (2019)
*Basella alba* L.	Pain	78	100	78.35	Narapusetty et al. (2017), Kalusalingam et al. (2018), Abioye et al. (2019)
*Garcinia buchananii* Bak.	Abdominal pain	82	100	87.21	Kalusalingam et al. (2018), Patterson et al. (2023), S et al. (2015)
*Musa acuminata* Colla	Dysentery	79	100	86.14	Mangussad et al. (2021), AMADI et al. (2022)
*Rauvolfia caffra* Sond.	Pneumonia	76	97	78.35	Erasto et al. (2011), Milugo et al. (2013), Njau et al. (2014)
*Gouania longispicata* Engl.	Wounds	87	101	81.19	Demgne et al. (2022), Mehboob et al. (2022)
*Spathodea campanulata* Buch.-Ham. ex DC.	Inflammation	76	97	78.13	Ilodigwe and Akah (2009), Vijayasanthi et al. (2015)
*Solanum aculeastrum* Dunal	Cancer	84	97	78.35	Koduru et al. (2006), Aboyade et al. (2009), Vijayasanthi et al. (2015), Hikaambo et al. (2023)
	Gonorrhea	82	101	83.51	
*Lagenaria siceraria* (Molina) Standl.	Promote urination	75	96	82.65	Aboyade et al. (2009), Mehboob et al. (2022)
	Vomiting	76	97	80.61	
*Rubia cordifolia* L.	Pains	81	97	88.42	Kasture et al. (2001), Patel et al. (2010), Diwane et al. (2015), Shen et al. (2018)
*Warburgia ugandensis* Sprague	Malaria	81	98	83.33	Maobe et al. (2013), Were et al. (2020), Okello et al. (2023), Wekesa et al. (2023)
*Baccharoides lasiopus* (O.Hoffm.) H.Rob.	Stomachache	84	95	87.50	Kareru et al. (2008), Muriithi et al. (2015), Guchu et al. (2020)
	Pain	85	102	91.67	
	Abdominal pain	74	92	78.13	
*Scutia myrtina Kurz*	Fever	84	96	82.80	Hou et al. (2009), Dhone et al. (2022)
	Malaria	88	96	79.57	
*Juniperus procera Hochst. ex Endl.*	Wounds	75	96	78.79	Tumen et al. (2013)
*Croton macrostachyu*s Del.	Epilepsy	77	93	79.21	Bum et al. (2012), Degu et al. (2016), Tegegne et al. (2023)
	Diarrhoea	74	93	80.20	
	Skin diseases	78	99	80.20	
*Ekebergia capensis* Sparrm.	Headache	80	101	79.59	Mulaudzi et al. (2013), Oyedeji-Amusa et al. (2020)
*Prunus africana* (Hook.f) Scweinf.	Cancer	81	101	87.63	Asuzu et al. (2021), Komakech et al. (2022), Nambooze et al. (2022)
*Calpurnia aurea (Aiton) Benth. subsp.aurea*	Eye Diseases	81	101	78.00	Adedapo et al. (2008), Dula and Zelalem (2018), Me et al. (2019), Derso (2020)
*Vachellia hockii* (De Wild.) Seigler & Ebinger	Abdominal pain	78	98	78.35	Kamau (2016), Kamau and Pm (2016), Guchu et al. (2020)
*Clutia abyssinica Jaub.& Spach*	Headache	78	100	85.00	Jeruto et al. (2015), Guchu et al. (2020), Meharie et al. (2020), Zayede et al. (2020)
	Malaria	76	97	78.00	
	Influenza	78	99	80.00	

FL= (Ip/Iu) × 100 Where “Ip” is the number of informants who share their knowledge about a given species for the treatment of a specific disease and “Iu” is the total number of all informants who reported all uses about a given plant species.

#### 3.4.1 Relationship between relative frequency of citation and use value

**FIGURE 9:**
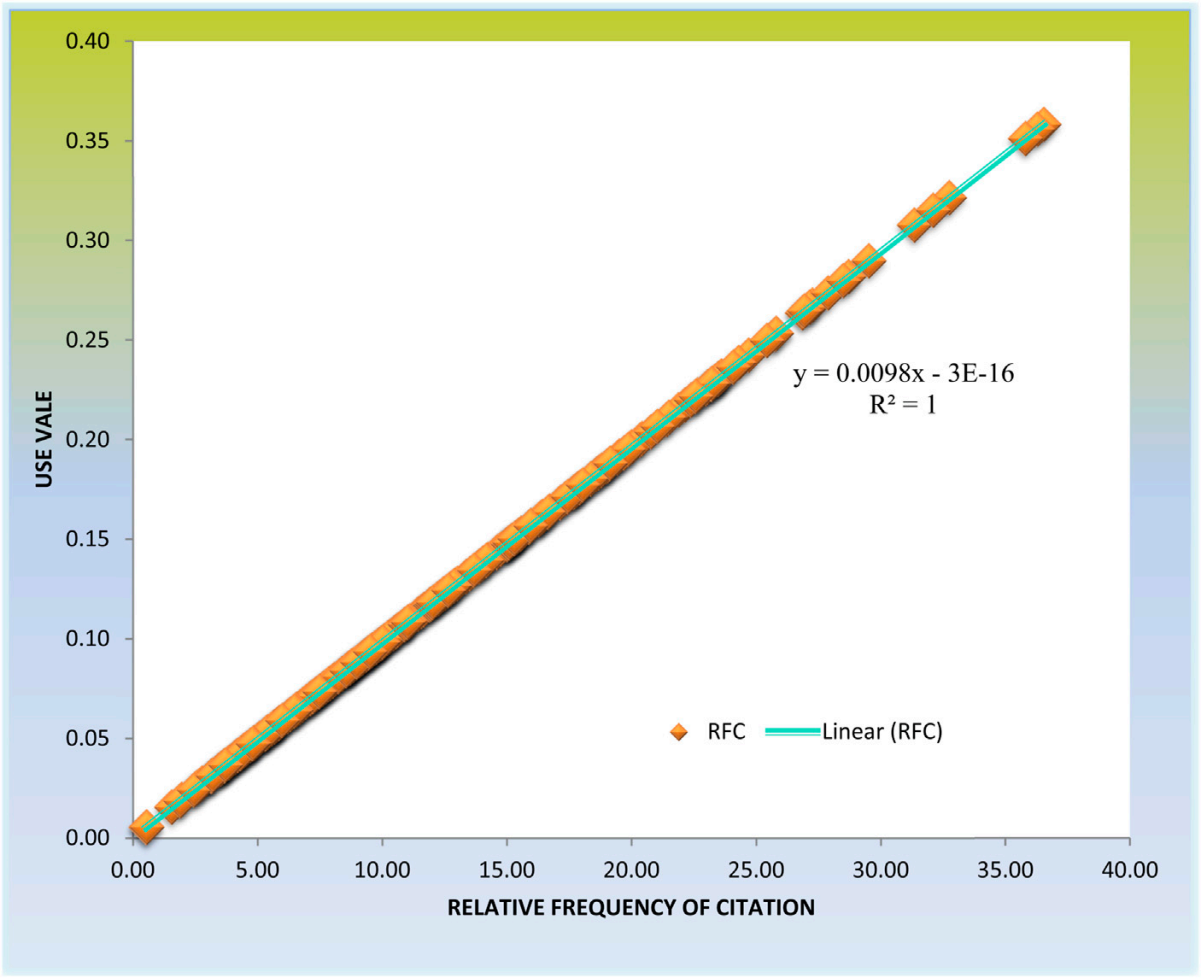
Correlation between relative frequency citation and use value.

The study found a precise correlation between the relative relevance of using medicinal plants and the local importance of each species (Figure 9). The Pearson correlation coefficient between RFCs and UVs was 1.00 (*p*-value <0.05). Despite this finding, some medicinal plant species exhibited high RFC and UV values, whereas others had low UV values but were still highly significant to the community. For instance, *Tiliacora triandra* (Colebr.) Diels with the least UV of 0.53 had an RFC of 0.53; *Tabernaemontana stapfiana* Britten had a UV of 0.55 and RFC value of 0.55; *Sida cordifolia* L had a UV of 1.57 and an RFC of 0.73 and *Leucas calostachys* Oliv. Had a UV of 1.89 and RFC of 0.8 (Supplementary Table S2). Their RFC values are above the half mark (0.5) suggesting that these specific species are commonly used in the study area to prepare remedies for various illnesses and diseases. It simply indicates that the subjective worth these botanical indexes hold for the local population is not always reflected in them. Therefore future research could look into such medicinal plant species with the highest RFCs would be the choice of medicinal plant species for future research on bioactive phytochemicals, drug discovery and development. These medicinal plant species were very important from a community’s medicinal perspective.

## 4 Discussion

The importance of medicinal plants for healthcare is one reason why research on them continues to receive a lot of attention both nationally and globally. It is imperative to have a thorough knowledge both scientifically and culturally through acquired traditional knowledge to promote the use traditional medicinal therapies as an important complement to conventional medicine. Therefore, more research must be done to ascertain the validity and efficacy of medicinal plants before making them available to humans as an alternative medicine. The current study found that the residents hold a very rich cultural tradition of medicinal plant use in treatment and management of different ailments and diseases. In this study, male practitioners’ were more than their female counter parts. However, the range of plant species used and their mode of preparation were quite comparable in both male and female healers. Age of the respondents on the other hand showed a positive correlation to the knowledge on the diversity of medicinal plant species used by the community. The older responders provided more information since they have access to a greater amount of the oral tradition knowledge passed down from generations before them (Miguéis et al., 2019; Dapar et al., 2020; Weldearegay and Awas, 2021). This outcome is based on a great deal of experience, indicating that knowledge on medicinal plants develops over a long period of time (Dapar et al., 2020; Khakurel et al., 2022).

Torres-Avilez et al. (2016) also did not find a significant difference between men and women in terms of their knowledge on traditional herbal medicine because these differences are not unidirectional and can only be detected on small scales of studies. A greater knowledge gap between both genders is not likely or improbable (Torres-Avilez et al., 2016; Khakurel et al., 2022). There have also been various reports of studies on ethnomedicinal knowledge and applications among Kenya’s various communities consistent with the current study reporting a higher proportion of male participants than the females (Kimondo et al., 2015; Ochwang’i et al., 2014; Wanjohi et al., 2020b) and indeed other regional and international studies (Amjad et al., 2017; Faruque et al., 2018; Okori et al., 2022). However, these findings does not support the notion that women are considered repositories traditional medicinal knowledge owing to their critical role as care givers in every community setting (Imperato, 1981; Rasmussen, 1998; Tugume and Nyakoojo, 2019).

The most noticeable families in terms of the greatest number of species of medicinal plants documented were Asteraceae, Lamiaceae, Fabaceae and Acanthaceae. This study findings supports evidence from previous observations where Asteraceae was among the most reported families with species used dominantly in traditional therapeutic preparations due to the large number of its bioactive phytocompounds from Asteraceae (Jeruto et al., 2008b; Gakuya et al., 2020; Mokua et al., 2021). The fact that the same plant species are used to cure the same illness in several locations demonstrates their vast spread and the fact that these plant species are successful in treating the precise illnesses (Bekalo et al., 2009; Amjad et al., 2017; Faruque et al., 2018; Tugume and Nyakoojo, 2019). Fabaceae is the largest plant family in Kenya with 576 species, followed by Asteraceae (403), Malvaceae (219), Lamiaceae (206) and Euphorbiaceae (219) (Zhou et al., 2017; Nankaya et al., 2020). Asteraceae and Lamiaceae species are herbaceous weeds that grow in disturbed regions, making them easily accessible (Nankaya et al., 2020).

The widespread use of herbaceous medicinal plants may be due to their abundance in the research area. Every part of the plant was employed by the local herbalists, but this study indicated that the leaves were the most often used component. This is probably because they are the easiest to gather and are likely to contain the most bioactive compounds. Our results corroborate those of (Tugume and Nyakoojo, 2019; Abdullah et al., 2021; Usman et al., 2021) who found that leaves were the most often used component of the plants. We realized that the most preferred preparation method was decoction. This is consistent with other research that found decoction to be the most popular preparation technique due to its simplicity in preparation (Salinitro et al., 2017; Nankaya et al., 2019; Junsongduang et al., 2020).

Medicinal plant species with high use may have a great potential for healing illnesses, however medicinal plant species with low RFC or UV likewise ought not to be disregarded as there is a possibility that a gradual loss of knowledge could result for not providing information about such medicinal plant species to the next-generation (Zenderland et al., 2019; Cordero et al., 2022; Okori et al., 2022). Identification of the most significant medicinal plant species depends on profound quantitative analysis of data and subjective interpretation of ethnobotanical data acquired in the field to ascertain their authenticity for development of marketable products. As their preferred usage may put their natural populations at risk from overharvesting, these species should also be given priority for conservation (Amjad et al., 2017; Wali et al., 2022; Ullah et al., 2023).

High ICF values are obtained when a considerable number of informants report to have used one or a small number of medicinal plant species to treat or manage a certain illness or diseases. Low ICF values on the other hand suggest that practitioners’ have divergent opinions on the best medicinal plant species to utilize. Additionally, a low ICF score indicates that less traditional treatments are being used due to the accessibility of conventional medications that offer contemporary substitutes for traditional medicinal therapies (Friedman et al., 1986; Faruque et al., 2018). The ICF values may vary from culture to culture due to the variations in medicinal plants species that are identified and used in various regions as well as the illnesses and diseases that these medicinal plants species are used to treat and manage. High ICF values can be utilized to pinpoint very significant medicinal plant species while looking for biologically active compounds.

## 5 Conclusion

The study’s findings showed that there is a significant diversity of medicinal plants in Mosop of Nandi County of Kenya. This being the first ethnobotanical undertaking in the study site it forms a basis of knowledge for future research. The wealth of information on ethnomedicinal plant species and their therapeutic uses could inspire further phytochemical and pharmacological investigations that may result in the development of potentially significant pharmaceuticals. To use traditional medicinal preparations as valuable complement to conventional medicine, more research must be done to ascertain the validity and efficacy of the plants before extending their use to other communities. Elderly practitioners were the main sources of indigenous knowledge and are frequently knowledgeable about the local flora and fauna. Respect for their knowledge must be shown and it must be further investigated, documented, validated, and applied to the advantage of both the community and the general public. Medicinal plant species with the highest use reports were used to treat certain infectious and parasitic category of diseases. The present study’s findings can be used to create or enhance initiatives such as *in situ* establishment of medicinal plant gardens at particular sites in collaboration with the local population.

## Scope statement

The study documented the diversity of medicinal plants used by the community of Mosop in Nandi-Kenya and gathered data from traditional medicine practitioners about the different parts used and the most effective way to administer them. Utilizing statistical methods, this study shows the relationship between the identified disease categories and the medicinal plant species used by determining the quantitative relevance of the data collected using a variety of indicators. The results show the diversity and richness of 253 species of medicinal plants spanning 74 families. The medicinal plant species that have been used to treat specific infectious and parasitic disease categories have the most use reports. The wealth of information from the study will encourage further phytochemical and pharmacological investigations that may result in the development of potentially significant pharmaceuticals. Global warming is having an increasing impact on biological richness, thus the world will have to deal with its effects as they become more obvious. The results, in our opinion, will be of interest to environmental conservationists who are trying to support biodiversity through initiatives such as the *in situ* establishment of medicinal plant gardens at particular sites in collaboration with the local population.

## Data Availability

The original contributions presented in the study are included in the article/Supplementary Material, further inquiries can be directed to the corresponding authors.
