# Supplementary material for: Ethnobotanical study of medicinal plants used by the people of Mosop, Nandi County in Kenya
Source: Front Pharmacol. 2024 Jan 19;14:1328903. doi: 10.3389/fphar.2023.1328903 (PMC10834697; doi:10.3389/fphar.2023.1328903)
Supplement: Supplementary file 1 [file Table1.DOCX]

| **Medicinal plant** | **Specific Disease** | **Ip** | **Iu** | **FL** | **References** |
| --- | --- | --- | --- | --- | --- |
| *Phytolacca dodecandra*L'Hér. | Syphilis | 78 | 99 | 78.79 | [1-3] |
| *Terminalia schimperiana*Hochst. ex Engl. & Diels | Cough | 79 | 100 | 79.00 | [4-6] |
| *Eucalyptus globulus* Labill. | Asthma | 81 | 101 | 80.20 | [7-19] |
|  | Inflammations | 83 | 101 | 82.18 |  |
|  | Wounds | 83 | 101 | 82.18 |  |
| *Nicoteba betonica*(L.) Lindau | Vomiting | 80 | 98 | 81.63 | [20-25] |
|  | Constipation | 78 | 98 | 79.59 |  |
|  | Pain | 77 | 98 | 78.57 |  |
|  | Malaria | 84 | 98 | 85.71 |  |
|  | Headache | 77 | 98 | 78.57 |  |
| *Vachellia nilotica* subsp. *tomentosa*(Benth.) Kyal. & Boatwr. | Bronchitis | 85 | 100 | 85.00 | [26-34] |
|  | Diarrhoea | 80 | 100 | 80.00 |  |
|  | Dysentery | 80 | 100 | 80.00 | [35-39] |
| *Ajuga integrifolia*Buch.-Ham. ex D.Don | Malaria | 85 | 100 | 85.00 |  |
| *Bridelia micrantha*(Hochst.) Baill. | General health | 80 | 98 | 81.63 | [40-42] |
| *Tragia brevipes*Pax | Headache | 85 | 102 | 83.33 | [43, 44] |
| *Urena lobata* L. | Asthma | 80 | 100 | 80.00 | [45, 46] |
| *Barleria grandicalyx* Lindau | Wounds | 81 | 98 | 82.65 | [47] |
|  | Cough | 77 | 98 | 78.57 |  |
| Senegalia senegal (L.) Britton | Throat | 93 | 100 | 93.00 | [48] |
|  | Discomfort | 79 | 100 | 79.00 |  |
| *Solanecio mannii* (Hoof.f.) C.Jeffrey | Rheumatism | 81 | 96 | 84.38 | [49] |
| *Tarenna graveolens* (S.Moore) Bremek. | Rheumatism | 78 | 97 | 80.41 | [50] |
| *Micromeria* Benth. | Headache | 75 | 91 | 82.42 | [51, 52] |
| *Micromeria biflora* (Buch. - Ham. ex D.Don) Benth. | Headache | 76 | 91 | 83.52 | [53-55] |
| *Aspilia pluriseta* Schweinf. ex Engl. | Wounds | 85 | 101 | 84.16 | [56-59] |
| *Tylosema fassoglensis* (Kotschy ex Schweinf.) Torre & Hillc. | Pneumonia | 80 | 99 | 80.81 | [60] |
| *Thunbergia alata* Bojer ex Sims | Diarrhea | 80 | 98 | 81.63 | [61, 62] |
|  | Fever | 77 | 98 | 78.57 |  |
| *Gymnosporia undata*(Thunb.) Szyszyl. | Syphilis | 77 | 98 | 78.57 | [63] |
| *Hoslundia oppositae* Vahl | Wounds | 79 | 99 | 79.80 | [64-66] |
| *Grewia similis* K. Schum | Cold | 78 | 100 | 78.00 | [67] |
|  | Cough | 83 | 100 | 83.00 |  |
| *Lippia javanica* Spreng*.* | Indigestion | 85 | 102 | 79.21 | [68-70] |
| *Leonotis nepetifolia* (L.) R.Br. | Cough | 80 | 101 | 83.67 | [71-73] |
|  | Burns | 82 | 98 | 79.59 |  |
|  | Back pain | 78 | 98 | 79.59 |  |
|  | Joint pain | 78 | 98 | 86.73 |  |
| *Olea europaea*subsp. *cuspidata*(Wall. & G.Don) Cif. | Sore throat | 85 | 98 | 78.57 | [74] |
| *Zanthoxylum chalybeum Engl.* | Fever | 77 | 98 | 81.37 | [75-79] |
|  | Malaria | 83 | 102 | 81.37 |  |
| *Coleus barbatus*(Andrews) Benth. ex G.Don | Pain | 83 | 102 | 85.15 | [80, 81] |
|  | Coughs | 86 | 101 | 79.21 |  |
| *Cleome gynandra*L. | Bites | 80 | 101 | 78.57 | [82-84] |
|  | Chest pain | 77 | 98 | 79.59 |  |
|  | Pain | 78 | 98 | 92.86 |  |
| *Schrebera alata*(Hochst.) Welw. | Bleeding | 91 | 98 | 88.00 | - |
| *Dovyalis abyssinica* (A.Rich.) Warb. | Stomachache | 88 | 100 | 80.81 | [85-87] |
|  | Cancer | 80 | 99 | 79.80 |  |
| *Entada africana* Guill. & Perr. | Stomachache | 79 | 99 | 78.22 | [88-91] |
|  | Dysentery | 79 | 101 | 80.20 |  |
| *Entada abyssinica* Steudel ex A.Rich. | Coughs | 81 | 101 | 78.43 | [92-95] |
|  | Fever | 77 | 97 | 78.43 |  |
| *Combretum pisoniiflorum*(Klotzsch) Engl. | Snake Bite | 80 | 102 | 82.65 | [96-98] |
| *Combretum collinum* Fresen | Indigestion | 80 | 102 | 78.13 | [99, 100] |
| *Lactuca macrophylla*(Willd.) A.Gray | Sores | 81 | 98 | 84.85 | [101] |
| *Vangueria infausta* Burch. | Pneumonia | 75 | 96 | 79.21 | [102-104] |
|  | Fever | 84 | 99 | 78.22 |  |
| *Gymnosporia heterophylla*(Eckl. & Zeyh.) Loes | Wounds | 79 | 101 | 78.57 | [105-108] |
|  | Pain | 60 | 74 | 90.82 |  |
| *Croton dichogamus* Pax. | Fever | 96 | 99 | 80.00 | [109, 110] |
|  | Stomachache | 77 | 98 | 81.00 |  |
|  | Malaria | 89 | 98 | 80.00 |  |
| *Syzygium guineense* (Willd.) DC | Pain | 81 | 100 | 84.85 | [111-113] |
| *Carissa spinarum*L. | Headache | 80 | 100 | 78.79 | [114-119] |
|  | Complaints | 79 | 98 | 83.84 |  |
|  | Rheumatism | 84 | 99 | 84.85 |  |
|  | Pain | 78 | 99 | 80.81 |  |
| *Vachellia sieberana* var*.* Woodii (Burtt Davy) Kyal. & Boatwr. | Oedema | 83 | 99 | 86.00 | [120-123] |
|  | Stomachache | 84 | 99 | 84.00 |  |
|  | Diarrhoea | 80 | 99 | 82.00 |  |
|  | Pain | 86 | 100 | 91.00 |  |
|  | Inflammation | 84 | 100 | 91.00 |  |
| *Tarchonanthus camphoratus* L. | Bronchitis | 82 | 100 | 81.19 | [124, 125] |
| *Flacourtia indica* (Burm.f.) Merr. | Colic | 91 | 100 | 78.22 | [126-128] |
|  | Fever | 91 | 100 | 87.13 |  |
|  | Cough | 82 | 101 | 80.20 |  |
| *Psidium guajava* L. | Diarrhoea | 79 | 101 | 78.22 | [129-140] |
|  | Inflammation | 88 | 101 | 85.15 |  |
|  | Pain | 81 | 101 | 81.19 |  |
| *Croton megalocarpus Hutch.* | Malaria | 82 | 101 | 85.00 | [141, 142] |
|  | Fever | 79 | 97 | 81.00 |  |
| *Physalis peruviana* L*.* | Worms | 75 | 96 | 81.19 | [143-145] |
|  | Malaria | 85 | 100 | 83.17 |  |
| *Markhamia lutea* (Benth.) K. Schum | Diarrhea | 81 | 100 | 79.80 | [146-148] |
|  | Pain | 82 | 101 | 86.87 |  |
| *Ficus sur*Forssk. | Gonorrhoea | 84 | 101 | 81.19 | [149-151] |
|  | Pain | 79 | 99 | 84.16 |  |
| *Protea gaguedi* J.F.Gmel. | Diarrhoea | 86 | 99 | 81.72 | - |
| *Ehretia cymosa* Thonn. | Wounds | 82 | 101 | 78.00 | [152, 153] |
|  | Epilepsy | 85 | 101 | 82.00 |  |
| *Azadirachta indica* A.Juss. | Ulcers | 76 | 93 | 79.00 | [154-164] |
| *Basella alba* L. | Pain | 78 | 100 | 78.35 | [162, 164, 165] |
| *Garcinia buchananii* Bak. | Abdominal pain | 82 | 100 | 87.21 | [165-167] |
| *Musa acuminata*Colla | Dysentery | 79 | 100 | 86.14 | [168, 169] |
| *Rauvolfia caffra* Sond. | Pneumonia | 76 | 97 | 78.35 | [170-172] |
| *Gouania longispicata* Engl. | Wounds | 87 | 101 | 81.19 | [173, 174] |
| *Spathodea campanulata* Buch.-Ham. ex DC. | Inflammation | 76 | 97 | 78.13 | [175, 176] |
| *Solanum aculeastrum* Dunal | Cancer | 84 | 97 | 78.35 | [176-179] |
|  | Gonorrhea | 82 | 101 | 83.51 |  |
| *Lagenaria siceraria* (Molina) Standl. | Promote urination | 75 | 96 | 82.65 | [174, 179] |
|  | Vomiting | 76 | 97 | 80.61 |  |
| *Rubia cordifolia* L. | Pains | 81 | 97 | 88.42 | [180-183] |
| *Warburgia ugandensis* Sprague | Malaria | 81 | 98 | 83.33 | [144, 184-186] |
| *Baccharoides lasiopus* (O.Hoffm.) H.Rob. | Stomachache | 84 | 95 | 87.50 | [187-189] |
|  | Pain | 85 | 102 | 91.67 |  |
|  | Abdominal pain | 74 | 92 | 78.13 |  |
| *Scutia myrtina  Kurz* | Fever | 84 | 96 | 82.80 | [190, 191] |
|  | Malaria | 88 | 96 | 79.57 |  |
| *Juniperus procera Hochst. ex Endl.* | Wounds | 75 | 96 | 78.79 | [192] |
| *Croton macrostachyu*s Del. | Epilepsy | 77 | 93 | 79.21 | [193-195] |
|  | Diarrhoea | 74 | 93 | 80.20 |  |
|  | Skin diseases | 78 | 99 | 80.20 |  |
| *Ekebergia capensis* Sparrm. | Headache | 80 | 101 | 79.59 | [196, 197] |
| *Prunus africana* (Hook.f) Scweinf. | Cancer | 81 | 101 | 87.63 | [198-200] |
| *Calpurnia aurea (Aiton) Benth. subsp.aurea* | Eye Diseases | 81 | 101 | 78.00 | [201-204] |
| *Vachellia hockii*(De Wild.) Seigler & Ebinger | Abdominal pain | 78 | 98 | 78.35 | [187, 205-207] |
| *Clutia abyssinica Jaub.& Spach* | Headache | 78 | 100 | 85.00 | [187, 208-210] |
|  | Malaria | 76 | 97 | 78.00 |  |
|  | Influenza | 78 | 99 | 80.00 |  |

1. Feyisa, K., et al., *Traditional Medicinal Plants Used for the Treatment of Urological and Urogenital Diseases in Ethiopia: A Review.* Pharmacognosy Journal, 2022. **14**(3).

2. Anthoney, S.T., et al., *in vitro antibacterial activity of the aqua extract of phytolacca dodecandra roots against laboratory strains of selected human pathogenic organisms.* 2015.

3. Matebie, W.A., W. Zhang, and G. Xie, *Chemical composition and antimicrobial activity of essential oil from Phytolacca dodecandra collected in Ethiopia.* Molecules, 2019. **24**(2): p. 342.

4. Burkill, H.M., *The useful plants of west tropical Africa, Vols. 1-3.* The useful plants of west tropical Africa, Vols. 1-3., 1995(2. ed.).

5. Adebayo, Ishola, and R. O, *Phytochemical and antimicrobial screening of crude extracts from the root, stem bark, and leaves of Terminalia glaucescens.* African journal of pharmacy and pharmacology, 2009. **3**(5): p. 217-221.

6. Fyhrquist, P., et al., *Antimycobacterial activity of ellagitannin and ellagic acid derivate rich crude extracts and fractions of five selected species of Terminalia used for treatment of infectious diseases in African traditional medicine.* South African journal of botany, 2014. **90**: p. 1-16.

7. Akhtar, M.A., et al., *Medicinal plants of the Australian aboriginal Dharawal people exhibiting anti-inflammatory activity.* Evidence-Based Complementary and Alternative Medicine, 2016. **2016**.

8. Ray, J., P. Goyal, and B.K. Aggarwal, *Approach of Eucalyptus globulus plant parts for human health safety and toxicological aspects.* Brit Open J Plant Sci, 2015. **1**(1): p. 1-10.

9. Masé, G. and G. Herbs, *allergies AND asthma.*

10. Mahishi, P., B. Srinivasa, and M. Shivanna, *Medicinal plant wealth of local communities in some villages in Shimoga District of Karnataka, India.* Journal of Ethnopharmacology, 2005. **98**(3): p. 307-312.

11. Manikandan, P.A., *Folk herbal medicine: A survey on the paniya tribes of Mundakunnu village of the Nilgiri hills, South India.* Ancient Science of life, 2005. **25**(1): p. 21.

12. Vigo, E., et al., *In‐vitro anti‐inflammatory effect of Eucalyptus globulus and Thymus vulgaris: nitric oxide inhibition in J774A. 1 murine macrophages.* Journal of Pharmacy and Pharmacology, 2004. **56**(2): p. 257-263.

13. Nile, S.H. and Y.S. Keum, *Chemical composition, antioxidant, anti-inflammatory and antitumor activities of Eucalyptus globulus Labill.* 2018.

14. Göger, G., et al., *In vitro antimicrobial, antioxidant and anti-inflammatory evaluation of Eucalyptus globulus essential oil.* Natural Volatiles and Essential Oils, 2020. **7**(3): p. 1-11.

15. Arooj, B., et al., *Anti-inflammatory mechanisms of eucalyptol rich Eucalyptus globulus essential oil alone and in combination with flurbiprofen.* Inflammopharmacology, 2023: p. 1-14.

16. Silva, J., et al., *Analgesic and anti-inflammatory effects of essential oils of Eucalyptus.* Journal of ethnopharmacology, 2003. **89**(2-3): p. 277-283.

17. Tomen, I., et al., *Characterization and wound repair potential of essential oil Eucalyptus globulus Labill.* Fresenius Environmental Bulletin, 2017. **26**(11).

18. Mota, V.d.S., R.N.T. Turrini, and V.d.B. Poveda, *Antimicrobial activity of Eucalyptus globulus oil, xylitol and papain: a pilot study.* Revista da Escola de Enfermagem da USP, 2015. **49**: p. 0216-0220.

19. Kubera Sampath Kumar, S., et al., *Study of wound dressing material coated with natural extracts of Calotropis Gigantean, Eucalyptus Globulus and buds of Syzygium Aromaticum solution enhanced with rhEGF (REGEN-DTM 60).* Journal of Natural Fibers, 2021. **18**(12): p. 2270-2283.

20. Rao, D.M., U. Rao, and G. Sudharshanam, *Ethno-medico-botanical studies from Rayalaseema region of southern Eastern Ghats, Andhra Pradesh, India.* Ethnobotanical Leaflets, 2006. **2006**(1): p. 21.

21. Jeruto, P., et al., *An ethnobotanical study of medicinal plants used by the Nandi people in Kenya.* Journal of ethnopharmacology, 2008. **116**(2): p. 370-376.

22. Pacifica, B., B. Nyanchongi, and R. Masai, *Ethnobotanical survey of medicinal plants used for treatment of malaria by Kipsigis people in Kericho County, Kenya.* IOSR Journal of Pharmacy and Biological Sciences, 2018. **13**: p. 24-30.

23. Mukungu, N., et al., *Medicinal plants used for management of malaria among the Luhya community of Kakamega East sub-County, Kenya.* Journal of Ethnopharmacology, 2016. **194**: p. 98-107.

24. Bbosa, G.S., et al., *Anti-Plasmodium falciparum activity of Aloe dawei and Justicia betonica.* 2013.

25. Prathibha, M. and S. Jayaramu, *Phytochemical Screening and in vitro Antihelmintic Properties of Justicia betonica L.*

26. Jaiswal, A., et al., *Babool (Acacia nilotica) and Oral Health.* Pharmacological Studies in Natural Oral Care, 2023: p. 597-606.

27. Ali, A., et al., *Acacia nilotica: a plant of multipurpose medicinal uses.* Journal of medicinal plants research, 2012. **6**(9): p. 1492-1496.

28. Farzana, M. and I. Al Tharique, *A review of ethnomedicine, phytochemical and pharmacological activities of Acacia nilotica (Linn) willd.* Journal of Pharmacognosy and Phytochemistry, 2014. **3**(1): p. 84-90.

29. Singh, R., et al., *Umbelliferone–An antioxidant isolated from Acacia nilotica (L.) Willd. ex. Del.* Food Chemistry, 2010. **120**(3): p. 825-830.

30. Sanni, S., et al., *The effect of Acacia nilotica pob ethyl acetate fraction on induced diarrhea in albino rats.* NY Sci. J, 2010. **3**: p. 16-20.

31. Hussain, F., et al., *Investigation of CNS depressant, anti-diarrheal and cytotoxic activities of crude methanolic extracts of Acacia nilotica and Justicia adhatoda root.* Indo Am J Pharm Res, 2016. **6**(1): p. 3954-3961.

32. Agunu, A., et al., *Evaluation of five medicinal plants used in diarrhoea treatment in Nigeria.* Journal of Ethnopharmacology, 2005. **101**(1-3): p. 27-30.

33. Manzo, L.M., I. Moussa, and K. Ikhiri, *Phytochemical screening of selected medicinal plants used against diarrhea in Niger, West Africa.* International Journal of Herbal Medicine, 2017. **5**(4): p. 32-38.

34. Misar, A., R. Bhagat, and A. Mujumdar, *Antidiarrhoeal activity of Acacia nilotica Willd. bark methanol extract.* Hindustan antibiotics bulletin, 2007. **49**(1-4): p. 14-20.

35. Kigen, C.K., *In silico prediction of anti-malarial activity and pharmacokinetic properties of herbal derivatives of Ajuga remota and Azadirachta indica.* Bachelor of Science degree in Medical Biochemistry in the Jomo Kenyatta University of Agriculture and Technology, 2019.

36. Kuria, K., et al., *Antimalarial activity of Ajuga remota Benth (Labiatae) and Caesalpinia volkensii Harms (Caesalpiniaceae): in vitro confirmation of ethnopharmacological use.* Journal of Ethnopharmacology, 2001. **74**(2): p. 141-148.

37. Nardos, A. and E. Makonnen, *In vivo antiplasmodial activity and toxicological assessment of hydroethanolic crude extract of Ajuga remota.* Malaria Journal, 2017. **16**: p. 1-8.

38. Kariuki, S., *STUDY OF CRUDE EXTRACTS OF Ajuga remota BENTH (LABIATAE) AS POTENTIAL ANTI-MALARIAL DRUG.* Journal of Environmental Sustainability Advancement Research, 2015. **1**.

39. Yacob, T., W. Shibeshi, and T. Nedi, *Antidiarrheal activity of 80% methanol extract of the aerial part of Ajuga remota Benth (Lamiaceae) in mice.* BMC Complementary and Alternative Medicine, 2016. **16**(1): p. 1-8.

40. Kathare, J.M., et al., *Antimicrobial Efficacy, Cytotoxicity, Acute Oral Toxicity, and Phytochemical Investigation of the Aqueous and Methanolic Stem Bark Extracts of Bridellia micrantha (Hochst.) Baill.* Pharmacognosy Journal, 2021. **13**(5).

41. Asumang, P., et al., *Antimicrobial, antioxidant and wound healing activities of methanol leaf extract of Bridelia micrantha (Hochst.) Baill.* Scientific African, 2021. **14**: p. e00980.

42. Omeh, Y.N., et al., *Subacute antidiabetic and in vivo antioxidant effects of methanolic extract of Bridelia micrantha (Hochst Baill) leaf on alloxan-induced hyperglycaemic rats.* Journal of Complementary and Integrative Medicine, 2014. **11**(2): p. 99-105.

43. Chepng’etich, J., et al., *Total phenolic content and in vitro antiproliferative activity of Tragia brevipes (Pax) and Tetradenia riparia (Hochst) leaves extract.* 2018.

44. Migabo, H., et al., *Evaluation of phytochemical profile and antimicrobial activity of Tragia brevipes extracts against pathogenic bacteria.*

45. Gudu, G.J., et al., *Phytochemical Analysis of Some Plants Used for Treatments of Respiratory Tract Disease in Zuru Metropolis.* World, 2023. **8**(3): p. 53-56.

46. Islam, M.H., et al., *Preliminary antihyperglycemic, antinociceptive activity, phytochemical analysis and toxicity studies on leaves of Urena lobata L.* J Chem Pharm Res, 2015. **7**(4): p. 559-63.

47. Rattan, R., *Bioactive Flavonoids from Acanthaceae Species-A.* 2023.

48. Mans, D.R., et al., *Phenolic Compounds and Antioxidant Activities of Eight Species of Fabaceae That Are Commonly Used in Traditional Medical Practices in the Republic of Suriname*, in *Medicinal Plants*. 2022, IntechOpen.

49. Muganga, R., et al., *Antiplasmodial and cytotoxic activities of Rwandan medicinal plants used in the treatment of malaria.* Journal of ethnopharmacology, 2010. **128**(1): p. 52-57.

50. Oloro, J., et al., *African Journal of Pharmacy and Pharmacology Phytochemical and efficacy study on four herbs used in erectile dysfunction: Mondia whiteii, Cola acuminata, Urtica massaica, and Tarenna graveolens.* 2016.

51. Matasyoh, J.C., et al., *Chemical composition and antimicrobial activity of the essential oil of Satureja biflora (Lamiaceae).* Bulletin of the Chemical Society of Ethiopia, 2007. **21**(2): p. 249-254.

52. Okach, D., A. Nyunja, and G. Opande, *Phytochemical screening of some wild plants from Lamiaceae and their role in traditional medicine in Uriri District-Kenya.* International Journal of Herbal Medicine, 2013. **1**(5): p. 135-143.

53. Aljohani, A.S., et al., *In Vivo and In Vitro Biological Evaluation and Molecular Docking Studies of Compounds Isolated from Micromeria biflora (Buch. Ham. ex D. Don) Benth.* Molecules, 2022. **27**(11): p. 3377.

54. Rauf, A., et al., *Antiglycation and enzyme inhibitory potential of salicylalazine isolated from Micromeria biflora (Buch.-Ham. ex D. Don) Benth.* South African Journal of Botany, 2021. **143**: p. 344-349.

55. Bouriah, N., et al., *Composition and profiling of essential oil, volatile and crude extract constituents of Micromeria inodora growing in western Algeria.* Journal of Pharmaceutical and Biomedical Analysis, 2021. **195**: p. 113856.

56. Kuria, J.M., *Efficacy of aspilia pluriseta schweinf in cutaneous wound healing in a mouse model*. 2014, University of Nairobi.

57. Kuria, J.M., et al., *Influence of Aspilia pluriseta Schweinf (Asteraceae) on the healing of dermal excision wounds (mouse model) and skin sensitization activity (Guinea pig model).* 2015.

58. Njeru, S.N. and J.M. Muema, *In vitro cytotoxicity of Aspilia pluriseta Schweinf. extract fractions.* BMC Research Notes, 2021. **14**(1): p. 1-4.

59. Njeru, S.N. and J.M. Muema, *Antimicrobial activity, phytochemical characterization and gas chromatography-mass spectrometry analysis of Aspilia pluriseta Schweinf. extracts.* Heliyon, 2020. **6**(10).

60. Adongo, J.O., et al., *Antimicrobial activity of the root extracts of Tylosema fassoglensis Schweinf. Torre & Hillc (Caesalpiniaceae).* 2012.

61. Cho, Y.-C., et al., *Thunbergia alata inhibits inflammatory responses through the inactivation of ERK and STAT3 in macrophages.* International Journal of Molecular Medicine, 2016. **38**(5): p. 1596-1604.

62. Mugaba, M., *Phytochemical constitunents analysis and formulation of herbal syrup from thunbergia alata extracts for the management of diarrhoea.* 2023.

63. Mokoka, T.A., et al., *Antimicrobial activity and cytotoxicity of triterpenes isolated from leaves of Maytenus undata (Celastraceae).* BMC complementary and alternative medicine, 2013. **13**: p. 1-9.

64. Annan, K. and R. Dickson, *Evaluation of wound healing actions of hoslundia opposita vahl, Anthocleista nobilis G. Don. and Balanites aegyptiaca L.* Journal of Science and Technology (Ghana), 2008. **28**(2): p. 26-35.

65. Namuga, C., et al., *Antibacterial activities of Bidens pilosa L, Hoslundia opposita Vahl, and Ageratum conyzoides L against some common wound pathogens.* African Journal of Pharmacy and Pharmacology, 2022. **16**(5): p. 64-78.

66. Onwuka, N.A., et al., *Evaluation of the immunomodulatory activity of Hoslundia opposita Vahl (Lamiaceae) leaf extract.* Int J Pharmacogn Phytochem Res, 2016. **8**: p. 1-7.

67. Muithya, J.N., *Phytochemical and in Vitro Anti-Microbial Screening of Echinops Hispidus Fresen. and Grewia similis K. Schum*. 2010, School of Pure and Applied Science Kenyatta University, Nairobi, Kenya.

68. Precious, N.L., *In vitro bioactivity of crude extracts of Lippia javanica on clinical isolates of Helicobacter pylori: Preliminary phytochemical screening*. 2010, University of Fort Hare.

69. Nkomo, L.P., *In vitro bioactivity of crude extracts of Lippia javanica on clinical isolates of Helicobacter pylori: preliminary phytochemical screening.* 2010.

70. Nkomo, L., E. Green, and R. Ndip, *Preliminary phytochemical screening and in vitro anti-Helicobacter pylori activity of extracts of the leaves of Lippia javanica.* African Journal of Pharmacy and Pharmacology, 2011. **5**(20): p. 2184-2192.

71. Pushpan, R., et al., *Evaluation of anti-arthritic potential of Leonotis nepetifolia (L.) R. Br. against Freund’s adjuvant induced arthritis.* Journal of Ayurveda and Integrated Medical Sciences, 2017. **2**(05): p. 59-66.

72. Giang, N.T.H., P. Van Ngot, and D.T.N. Thanh, *Morphological, anatomical and antibacterial characteristics of Leonotis nepetifolia plants growing in Binh Thuan Province, Vietnam.* GSC Biological and Pharmaceutical Sciences, 2021. **14**(2): p. 053-063.

73. Parra-Delgado, H., et al., *Anti-inflammatory activity of some extracts and isolates from Leonotis nepetaefolia on TPA-induced edema model.* Revista de la Sociedad Química de México, 2004. **48**(4): p. 293-295.

74. Masoko, P. and D.M. Makgapeetja, *Antibacterial, antifungal and antioxidant activity of Olea africana against pathogenic yeast and nosocomial pathogens.* BMC complementary and alternative medicine, 2015. **15**(1): p. 1-9.

75. Ngugi, D.N., *Study of antiplasmodial activity, cytotoxicity and acute toxicity of Zanthoxylum chalybeum ENGL, and Vernonia lasiopus o. Hoffman*. 2014, University of Nairobi.

76. Bbosa, G.S., et al., *Antiplasmodial Activity of Leaf Extracts of Zanthoxylum chalybeum Engl.* British Journal of Pharmaceutical Research, 2014. **4**(6): p. 705.

77. Müller-Jakic, B., et al., *Anti-inflammatory activity of Zanthoxylum chalybeum extracts and identification of protoberberine and benzophenanthridine alkaloids by GC-MS and HPLC.* Planta Medica, 1993. **59**(S 1): p. A664-A664.

78. Njenga, D., et al., *Antiplasmodial activity, cytotoxicity and acute toxicity of Zanthoxylum chalybeum engl.* World J Pharm Pharm Sci, 2016. **5**: p. 208-17.

79. Musila, M., et al., *In vivo antimalarial activity, toxicity and phytochemical screening of selected antimalarial plants.* Journal of ethnopharmacology, 2013. **146**(2): p. 557-561.

80. Ezeonwumelu, J.O., et al., *Phytochemical screening, toxicity, analgesic and anti-pyretic studies of aqueous leaf extract of Plectranthus barbatus [Andrews. Engl.] in rats.* Pharmacology & Pharmacy, 2019. **10**(04): p. 205-221.

81. Falé, P.L., et al., *Function of Plectranthus barbatus herbal tea as neuronal acetylcholinesterase inhibitor.* Food & function, 2011. **2**(2): p. 130-136.

82. del Carmen Juárez-Vázquez, M. and M.A. Jiménez-Arellanes, *Phytochemical investigation, anti-inflammatory and antinociceptive activities from some species of Cleomaceae family: a systematic review.* Adv. Med. Plans Res, 2019. **7**(4): p. 107-128.

83. Narendhirakannan, R., S. Subramanian, and M. Kandaswamy, *Anti-inflammatory and lysosomal stability actions of Cleome gynandra L. studied in adjuvant induced arthritic rats.* Food and chemical toxicology, 2007. **45**(6): p. 1001-1012.

84. Sivakumar, K., et al., *Antiulcer and Analgesic Activity of the Ethanol Extract of Cleome gynandra Linn Leaves.* Research journal of pharmacy and technology, 2010. **3**(3): p. 766-769.

85. Emam, J.A., et al., *In Vitro Anticancer Activities of Selected Ethiopian Plant Extracts on HeLa and PC3 Cell Lines.* Ethiopian Pharmaceutical Journal, 2021. **37**(1): p. 77-82.

86. Kipngeno, C.D., *Screening and characterization of some anticancer compounds from Salicaceae, Myrtaceae, Euphorbiaceae and Solanaceae families*. 2019, Egerton University.

87. Legesse, B.A., A. Tamir, and B. Bezabeh, *Phytochemical screening and antibacterial activity of leaf extracts of Dovyalis abyssinica.* J Emerg Technol Innov Res, 2019. **6**(6): p. 453-465.

88. Yusuf, A. and M. Abdullahi, *The phytochemical and pharmacological actions of Entada africana Guill. & Perr.* Heliyon, 2019. **5**(9).

89. Hassan, L., et al., *Phytochemical Screening, Isolation and Characterization of Beta-Sitosterol from ethyl acetate Extract of Stem Bark of Entada africana (Fabaceae) Guill. et Perr.* Journal of Chemical Society of Nigeria, 2018. **43**(3).

90. Adewole, E., et al., *Phytochemicals profile and in-vitro antidiabetic potentials of fractionated extracts of Entada Africana and Leptadenia Hastata.* ScienceRise: Pharmaceutical Science, 2022(3 (37)): p. 65-73.

91. Akindele, A.J., et al., *Gastroprotective effects of the aqueous seed extract of Entada gigas (Linn.) Fawc. and Rendle (Fabaceae) in ulcer models in rats.* African Journal of Pharmacology and Therapeutics, 2016. **5**(3).

92. Roger, T., M. Pierre-Marie, and V.K. Igor, *Phytochemical screening and antibacterial activity of medicinal plants used to treat typhoid fever in Bamboutos division, West Cameroon.* Journal of Applied Pharmaceutical Science, 2015. **5**(6): p. 034-049.

93. Teke, G.N., et al., *Antimicrobial and antioxidant properties of methanol extract, fractions and compounds from the stem bark of Entada abyssinica Stend ex A. Satabie.* BMC complementary and alternative medicine, 2011. **11**(1): p. 1-8.

94. Mariita, R.M., et al., *Antifungal, antibacterial and antimycobacterial activity of Entada abysinnica Steudel ex A. Rich (Fabaceae) methanol extract.* Pharmacognosy research, 2010. **2**(3): p. 163.

95. Olajide, O.A., A. Akinola Alada, and O.T. Kolawole, *Anti-inflammatory Properties of Entada abyssinica. Leaves.* Pharmaceutical biology, 2005. **43**(7): p. 583-585.

96. Molander, M., et al., *Hyaluronidase, phospholipase A2 and protease inhibitory activity of plants used in traditional treatment of snakebite-induced tissue necrosis in Mali, DR Congo and South Africa.* Journal of Ethnopharmacology, 2014. **157**: p. 171-180.

97. Hamza, R.Z., S.E. Al-Motaani, and T. Al-Talhi, *Therapeutic and ameliorative effects of active compounds of combretum molle in the treatment and relief from wounds in a diabetes mellitus experimental model.* Coatings, 2021. **11**(3): p. 324.

98. Miaffo, D., et al., *Toxicological evaluation of aqueous and acetone extracts of Combretum molle twigs in Wistar Rats.* eJBio., 2015. **11**: p. 33-45.

99. Njume, C., et al., *In-vitro anti-Helicobacter pylori activity of acetone, ethanol and methanol extracts of the stem bark of Combretum molle (Combretaceae).* Journal of Medicinal Plants Research, 2011. **5**(14): p. 3210-3216.

100. Marquardt, P., et al., *Phytochemical characterization and in vitro anti-inflammatory, antioxidant and antimicrobial activity of Combretum collinum Fresen leaves extracts from Benin.* Molecules, 2020. **25**(2): p. 288.

101. Waiganjo, N., H. Ochanda, and D. Yole, *Phytochemical analysis of the selected five plant extracts.* 2016.

102. de Boer, H.J., et al., *Anti-fungal and anti-bacterial activity of some herbal remedies from Tanzania.* Journal of ethnopharmacology, 2005. **96**(3): p. 461-469.

103. Addo-Mensah, A. and D.P. Holland, *Evaluation of the antimicrobial activity of vangueria volkensii bark, fruit, leaf, and stem extracts.* Journal of Medicinal Plants, 2022. **10**(2): p. 208-214.

104. Gwatidzo, L., et al., *In vitro anti-inflammatory activity of Vangueria infausta: An edible wild fruit from Zimbabwe.* African Journal of Pharmacy and Pharmacology, 2018. **12**(13): p. 168-175.

105. Da Silva, G., et al., *In vivo anti-inflammatory effect and toxicological screening of Maytenus heterophylla and Maytenus senegalensis extracts.* Human & experimental toxicology, 2011. **30**(7): p. 693-700.

106. Umar, S.I., et al., *Antioxidant and antimicrobial activities of naturally occurring flavonoids from M. heterophylla and the safety evaluation in Wistar rats.* Iranian Journal of Toxicology, 2019. **13**(4): p. 39-44.

107. Tyavambiza, C., et al., *Wound healing activities and potential of selected african medicinal plants and their synthesized biogenic nanoparticles.* Plants, 2021. **10**(12): p. 2635.

108. Omara, T., *Antimalarial plants used across Kenyan communities.* Evidence-Based Complementary and Alternative Medicine, 2020. **2020**.

109. Matara, D.N., et al., *Phytochemical analysis and investigation of the antimicrobial and cytotoxic activities of Croton dichogamus pax crude root extracts.* Evidence-Based Complementary and Alternative Medicine, 2021. **2021**.

110. Aldhaher, A., et al., *New terpenoids from Croton dichogamus Pax.* Planta Medica, 2016. **82**(S 01): p. P167.

111. IOR, I., I. Otimenyin, and M. Umar, *Anti-inflammatory and analgesic activities of the ethanolic extract of the leaf of Syzygium guineense in rats and mice.* 2012.

112. Tanko, Y., et al., *Anti-nociceptive and anti-inflammatory activities of ethanol extract of Syzygium aromaticum flower bud in wistar rats and mice.* African Journal of Traditional, Complementary and Alternative Medicines, 2008. **5**(2): p. 209-212.

113. Mollika, S., et al., *Evaluation of analgesic, anti-inflammatory and CNS activities of the methanolic extract of Syzygium samarangense leave.* Global Journal of Pharmacology, 2014. **8**(1): p. 39-46.

114. Maina, G.S., et al., *Antinociceptive properties of dichloromethane: methanolic leaf and root bark extracts of Carissa edulis in rats.* 2015.

115. Hassan, H.S., et al., *Analgesic and anti-inflammatory activities of the saponins extract of Carissa edulis root in rodents.* International Journal of Biological and Chemical Sciences, 2010. **4**(4).

116. Woode, E., et al., *Anti-inflammatory and antioxidant properties of the root extract of Carissa edulis (Forsk.) Vahl (Apocynaceae).* Journal of Science and Technology (Ghana), 2007. **27**(3): p. 5-15.

117. Dawa, I., et al., *Antimicrobial activities of methanol leaf extract of Carissa edulis Vahl (Apocynaceae).* 2021.

118. Jepkorir, M., et al., *In vivo anti-inflammatory activity, safety and gene expression profiles of Carissa edulis, Withania somnifera, Prunus africana and Rhamnus prinoides for potential management of rheumatoid arthritis.* Scientific African, 2023. **22**: p. e01933.

119. Fanta Yadang, S.A., et al., *Quantification of bioactive compounds and evaluation of the antioxidant activity of Carissa edulis Valh (Apocynaceae) leaves.* The Scientific World Journal, 2019. **2019**.

120. Würger, G., *A rational in vitro evaluation of 53 medicinal plants used in the treatment of diarrhoea and the potential use of Deinbollia oblongifolia (Sapindaceae) extracts*. 2010, University of Pretoria.

121. Jurbe, G., et al., *Phytochemical Screening and Antidiarrheal Evaluation of Acetone Extract of Acacia sieberiana var woodii (Fabaceae) stem bark in wistar rats.* 2015.

122. Ngaffo, C.M., et al., *The antiproliferative extract from the leaves of Acacia sieberiana var. woodii (Fabaceae) is harmless as evidenced by the acute and subacute toxicity studies in rats.* South African Journal of Botany, 2022. **150**: p. 217-224.

123. Wuerger, G., et al., *EVALUATION OF THE ACTIVITY OF LEAF EXTRACTS OF TREES THAT HAVE BEEN USED TO TREAT DIARRHOEA IN HUMANS AND ANIMALS.* African Journal of Traditional, Complementary and Alternative Medicines, 2009: p. 475-476.

124. Wetungu Martin, W., J. Matasyoh, and T. Kinyanjui, *Antimicrobial activity of solvent extracts from the leaves of Tarchonanthus camphoratus (Asteraceae).* Journal of Pharmacognosy and Phytochemistry, 2014. **3**(1): p. 123-127.

125. Ali, N.A.A., et al., *Antimicrobial, antioxidant, and cytotoxic activities of the essential oil of Tarchonanthus camphoratus.* Natural Product Communications, 2013. **8**(5): p. 1934578X1300800534.

126. Islam, M.A.F., et al., *Evaluation of analgesic, anti-inflammatory and antipyretic properties of the Flacourtia indica extract in laboratory animal.*

127. Chun, K. and J. Kundu, *Analgesic, Anti-inflammatory and Diuretic Activity of Methanol Extract of Flacourtia indica.* Archives of Basic and Applied Medicine, 2013. **1**(1): p. 39-44.

128. Tyagi, S., et al., *Anti-asthmatic potential of Flacourtia indica Merr.* African Journal of Basic and Applied Sciences, 2011. **3**(5): p. 201-204.

129. El-Ahmady, S.H., M.L. Ashour, and M. Wink, *Chemical composition and anti-inflammatory activity of the essential oils of Psidium guajava fruits and leaves.* Journal of Essential Oil Research, 2013. **25**(6): p. 475-481.

130. Jang, M., et al., *Anti-inflammatory effects of an ethanolic extract of guava (Psidium guajava L.) leaves in vitro and in vivo.* Journal of medicinal food, 2014. **17**(6): p. 678-685.

131. Siani, A.C., et al., *Anti-inflammatory activity of essential oils from Syzygium cumini and Psidium guajava.* Pharmaceutical biology, 2013. **51**(7): p. 881-887.

132. Dutta, S. and S. Das, *A study of the anti-inflammatory effect of the leaves of Psidium guajava Linn. on experimental animal models.* Pharmacognosy research, 2010. **2**(5): p. 313.

133. Lu, J., et al., *Changes of intestinal microflora diversity in diarrhea model of KM mice and effects of Psidium guajava L. as the treatment agent for diarrhea.* Journal of Infection and Public Health, 2020. **13**(1): p. 16-26.

134. Koriem, K.M., M.S. Arbid, and H.N. Saleh, *Antidiarrheal and protein conservative activities of Psidium guajava in diarrheal rats.* Journal of integrative medicine, 2019. **17**(1): p. 57-65.

135. Lobo, R. and M. Ballal, *Screening for antidiarrheal activity of Psidium guajava: A possible alternative in the treatment against diarrhea causing enteric pathogens.* J Chem, 2011. **3**: p. 961-967.

136. Lozoya, X., et al., *Intestinal anti-spasmodic effect of a phytodrug of Psidium guajava folia in the treatment of acute diarrheic disease.* Journal of ethnopharmacology, 2002. **83**(1-2): p. 19-24.

137. Hirudkar, J.R., et al., *The antidiarrhoeal evaluation of Psidium guajava L. against enteropathogenic Escherichia coli induced infectious diarrhoea.* Journal of ethnopharmacology, 2020. **251**: p. 112561.

138. Alabi, A.O., et al., *Anti-nociceptive and anti-inflammatory effects of an aqueous extract of blended leaves of Ocimum gratissimum and Psidium guajava.* Clinical Phytoscience, 2019. **5**(1): p. 1-9.

139. Sekhar, N.C., et al., *Evaluation of antinociceptive activity of aqueous extract of bark of Psidium guajava in albino rats and albino mice.* Journal of clinical and diagnostic research: JCDR, 2014. **8**(9): p. HF01.

140. Raja, N.L. and K. Sundar, *Psidium guajava Linn Confers Analgesic Effects on Mice.* Journal of Pharmaceutical Sciences and Research, 2016. **8**(6): p. 412.

141. Danyaal, M., *The isolation and purification of chemical constituents of'Croton megalocarpus' Hutch husks*. 2020, Kingston University.

142. Gichui, W.G., *Antinociceptive activities of extracts of Croton megalocarpus hutch (Eurphobiaceae) using animal models*. 2016, University of Nairobi.

143. Kamau, P.K., et al., *In vitro antiplasmodial, cytotoxicity assay and partial chemical characterization of Kenyan Physalis peruviana L.(Solanaceae family) extracts.* 2020.

144. Maobe, M.A., et al., *Preliminary phytochemical screening of eight selected medicinal herbs used for the treatment of diabetes, malaria and pneumonia in Kisii region, southwest Kenya.* European journal of applied sciences, 2013. **5**(10): p. 01-06.

145. Kathare, J., et al., *Antimicrobial, cytotoxicity, acute oral toxicity and qualitative phytochemical screening of the aqueous and methanolic extracts of Physalis peruviana L (Solanaceae).* Appl Microbiol Open Access, 2021. **7**: p. 189.

146. Ngoufack Azanze, E., et al., *Markhamia lutea leaves aqueous and ethanolic extract with curative anti-inflammatory activity attenuates paclitaxel toxicity in rat’s intestine.* Journal of Complementary and Integrative Medicine, 2023(0).

147. Lacroix, D., et al., *Antiplasmodial and cytotoxic activities of medicinal plants traditionally used in the village of Kiohima, Uganda.* Journal of Ethnopharmacology, 2011. **133**(2): p. 850-855.

148. Abdullah, W.O., et al., *In vitro antiplasmodial activity and cytotoxicity of ten plants used as traditional medicine in Malaysia.* Jurnal Sains Kesihatan Malaysia, 2011. **9**(2): p. 5-8.

149. Wakeel, O.K., et al., *Anti-Nociceptive and Anti-Inflammatory Effects of Stem Bark Extract of Ficus Capensis Thunb (Moraceae) by Bioactivity Fractionation.* Anti-Inflammatory & Anti-Allergy Agents in Medicinal Chemistry (Formerly Current Medicinal Chemistry-Anti-Inflammatory and Anti-Allergy Agents), 2021. **20**(2): p. 206-218.

150. Omodamiro, O.D., et al., *Evaluation of sub-chronic toxicity, anti-inflammatory and diuretic effect of ethanol leaves extract Ficus capensis in albino rat.* Animal Research International, 2021. **18**(2): p. 4073–4082-4073–4082.

151. Owolabi, A.O., et al., *Antibacterial and Phytochemical Potentials of Ficus capensis Leaf Extracts Against Some Pathogenic Bacteria.* Tropical Journal of Natural Product Research, 2022. **6**(3).

152. Nicholas, K., *IN-VITRO CYTOTOXICITY OF THREE SELECTED MEDICINAL PLANT EXTRACTS FROM KENYA.* 2017.

153. Adeleye, O., et al., *In Vivo Anti-Inflammatory Assessment of a Topical Formulation Containing Ehretia Cymosa Extract Mediated-Silver Nanoparticles.* Nigerian Journal of Pharmaceutical Research, 2021. **17**(2): p. 179-188.

154. Bandyopadhyay, U., et al., *Clinical studies on the effect of Neem (Azadirachta indica) bark extract on gastric secretion and gastroduodenal ulcer.* Life sciences, 2004. **75**(24): p. 2867-2878.

155. Raji, Y., et al., *Effects of Azadirachta indica extract on gastric ulceration and acid secretion in rats.* Journal of ethnopharmacology, 2004. **90**(1): p. 167-170.

156. Dorababu, M., et al., *Effect of Bacopa monniera and Azadirachta indica on gastric ulceration and healing in experimental NIDDM rats.* 2004.

157. Bhajoni, P.S., G.G. Meshram, and M. Lahkar, *Evaluation of the antiulcer activity of the leaves of Azadirachta indica: An experimental study.* Integrative Medicine International, 2016. **3**(1-2): p. 10-16.

158. Farzana, S., S.P. Saha, and N. Sultana, *Gastroprotective Effect of Azadirachta indica Leaves (Neem) Extract on Aspirin Induced Gastric Ulcer in Rats.* Delta Medical College Journal, 2019. **7**(2): p. 61-65.

159. Ofusori, D.A., et al., *Gastroprotective effect of aqueous extract of neem Azadirachta indica on induced gastric lesion in rats.* Int J Biol Med Res, 2010. **1**(4): p. 219-222.

160. Odo, C.E., *Effect of the methanol extract of the leaves of Azadirachta indica on ethanol-induced gastric ulcer in rats.* Journal of Pharmacy Research, 2016. **10**(1): p. 41-45.

161. Mohapatra, B.B., et al., *Anti-ulcer activity of aqueous and ethanolic leaf extract of neem (Azadirachta indica) in albino rats.* Journal of Pharmacy Research, 2012. **5**(3): p. 1571.

162. Abioye, A.V., et al., *Evaluation of the analgesic potential of Basella alba (L.) leaves (Basellaceae): doi. org/10.26538/tjnpr/v3i1. 5.* Tropical Journal of Natural Product Research (TJNPR), 2019. **3**(1): p. 22-25.

163. Kumar, V., et al., *In-vitro anti-inflammatory activity of leaf extracts of Basella alba linn. Var. alba.* Int J Drug Dev Res, 2011. **3**(2): p. 176-179.

164. Narapusetty, N., et al., *Anti-Inflammatory activity of Ethanolic extract of Basella alba in acute and Sub-acute Model.* Asian Journal of Pharmaceutical Research, 2017. **7**(2): p. 88-93.

165. Kalusalingam, A., et al., *Preliminary Phytochemical Screening And evaluation Of Analgesic Activity Of Basella Alba linn.* KPJ Medical, 2018. **7**(1): p. 38.

166. Patterson, S., et al., *Garcinia buchananii stem bark extract and its bioactive constituents manniflavanone, GB-2 and buchananiflavanone attenuate intestinal inhibitory neuromuscular transmission.* Journal of Smooth Muscle Research, 2023. **59**: p. 34-57.

167. Stark, T.D., et al., *Antioxidative compounds from Garcinia buchananii stem bark.* Journal of natural products, 2015. **78**(2): p. 234-240.

168. Mangussad, D., et al., *Isolation and Characterization of the Total Protein in’Lakatan’Banana (Musa acuminata Colla) with Bioactive Peptides Exhibiting Antioxidative and Antihypertensive Activities.* Philippine Agricultural Scientist (Philippines), 2021.

169. AMADI, B., R. BELLO, and R. OHIRI, *AMELIORATIVE POTENTIALS OF DICHLOROMETHANE EXTRACT OF Musa acuminata LATUNDAN BRACT IN INDOMETHACIN ADMINISTERED WISTAR RATS.* Asian Journal of Plant and Soil Sciences, 2022. **7**(1): p. 171-184.

170. Njau, E.-F., et al., *Antimicrobial and antioxidant activity of crude extracts of Rauvolfia caffra var. caffra (Apocynaceae) from Tanzania.* 2014.

171. Milugo, T.K., et al., *Antagonistic effect of alkaloids and saponins on bioactivity in the quinine tree (Rauvolfia caffra sond.): further evidence to support biotechnology in traditional medicinal plants.* BMC complementary and alternative medicine, 2013. **13**: p. 1-6.

172. Erasto, P., et al., *Antimycobacterial, antioxidant activity and toxicity of extracts from the roots of Rauvolfia vomitoria and R. caffra.* 2011.

173. Demgne, O.M.F., et al., *Antibacterial and antibiotic-potentiating activities of nine Cameroonian medicinal plants against multidrug-resistant bacteria expressing active efflux pumps.* Invest Med Chem Pharmacol, 2022. **5**(1): p. 58.

174. Mehboob, M., et al., *Medicinal and nutritional importance of Lagenaria siceraria (Lauki).* Saudi J Biomed Res, 2022. **7**(2): p. 67-73.

175. Ilodigwe, E.E. and P.A. Akah, *Spathodea campanulata: an experimental evaluation of the analgesic and anti-inflammatory properties of a traditional remedy.* Asian Journal of Medical Sciences, 2009. **1**(2): p. 35-38.

176. Vijayasanthi, M., A. Doss, and K. Kannan, *Anti-inflammatory activity of Spathodea campanulata P. Beauv. leaves against carrageenan induced paw edema.* Bio Technology-Elixir International Journal, 2015. **78**: p. 29450-29452.

177. Koduru, S., et al., *In vitro Antitumour Activity of Solanum aculeastrum.* International Journal of Cancer Research, 2006. **2**(4): p. 397-402.

178. Hikaambo, C.N.a., et al., *Antimicrobial Activities of Solanum aculeastrum Fruit Extract against Escherichia coli, Staphylococcus aureus and Candida albicans: Significance of African Traditional Medicine in Combating Infections and Attaining Universal Health Coverage.* Pharmacology & Pharmacy, 2023. **14**(5): p. 176-188.

179. Aboyade, O., et al., *Studies on the toxicological effect of the aqueous extract of the fresh, dried and boiled berries of Solanum aculeastrum Dunal in male Wistar rats.* Human & experimental toxicology, 2009. **28**(12): p. 765-775.

180. Shen, C.-H., et al., *Evaluation of analgesic and anti-inflammatory activities of Rubia cordifolia L. by spectrum-effect relationships.* Journal of Chromatography B, 2018. **1090**: p. 73-80.

181. Patel, A., et al., *Evaluation of anti inflammatory and analgesic activity of roots of Rubia cordifolia in rats.* Journal of Pharmaceutical Sciences and Research, 2010. **2**(12): p. 809.

182. Kasture, S., V. Kasture, and C. Chopde, *Anti-inflammatory activity of Rubia cordifolia roots.* Journal of Natural Remedies, 2001. **1**(2): p. 111-115.

183. Diwane, C., et al., *Protective effect of Rubia cordifolia in paclitaxel-induced neuropathic pain in experimental animals.* Indian J Pain, 2015. **29**: p. 150-154.

184. Okello, D., et al., *Comparative antiplasmodial activity, cytotoxicity, and phytochemical contents of Warburgia ugandensis stem bark against Aspilia africana wild and in vitro regenerated tissues.* Journal of Plant Biotechnology, 2023. **50**(1): p. 97-107.

185. Wekesa, D., et al., *Antiplasmodial and cytotoxic activities of selected medicinal plants in Western Kenya.* 2023.

186. Were, P.S., et al., *Warburgia ugandensis: A potent in vivo phytomedicine against Plasmodium knowlesi.* Journal of Pharmacognosy and Phytochemistry, 2020. **9**(5): p. 01-05.

187. Guchu, B.M., et al., *In vitro antioxidant activities of methanolic extracts of Caesalpinia volkensii Harms., Vernonia lasiopus O. Hoffm., and Acacia hockii De Wild.* Evidence-based Complementary and Alternative Medicine: eCAM, 2020. **2020**.

188. Muriithi, N.J., et al., *Determination of hematological effects of methanolic leaf extract of Vernonia lasiopus in normal mice.* 2015.

189. Kareru, P., et al., *Antimicrobial activity of some medicinal plants used by herbalists in eastern province, Kenya.* African Journal of Traditional, Complementary and Alternative Medicines, 2008. **5**(1): p. 51-55.

190. Hou, Y., et al., *Antiproliferative and antimalarial anthraquinones of Scutia myrtina from the Madagascar forest.* Bioorganic & medicinal chemistry, 2009. **17**(7): p. 2871-2876.

191. Dhone, P.G., et al., *In vivo antipyretic activity of Scutia myrtina acute oral toxicity study.*

192. Tumen, I., et al., *Topical wound-healing effects and phytochemical composition of heartwood essential oils of Juniperus virginiana L., Juniperus occidentalis Hook., and Juniperus ashei J. Buchholz.* Journal of medicinal food, 2013. **16**(1): p. 48-55.

193. Bum, E.N., et al., *Decoctions of Bridelia micrantha and Croton macrostachyus may have anticonvulsant and sedative effects.* Epilepsy & Behavior, 2012. **24**(3): p. 319-323.

194. Degu, A., E. Engidawork, and W. Shibeshi, *Evaluation of the anti-diarrheal activity of the leaf extract of Croton macrostachyus Hocsht. ex Del.(Euphorbiaceae) in mice model.* BMC Complementary and Alternative Medicine, 2016. **16**: p. 1-11.

195. Tegegne, H. and D. Woldegiorgis, *Testing the skin with Histofarcin and examining the in-vitro antifungal properties of Croton Macrostachyus against the mycelial form of Histoplasma capsulatum Variety Farciminosum, isolated from a horse in Adama City, Ethiopia.* 2023.

196. Mulaudzi, R., et al., *Anti-inflammatory and mutagenic evaluation of medicinal plants used by Venda people against venereal and related diseases.* Journal of Ethnopharmacology, 2013. **146**(1): p. 173-179.

197. Oyedeji-Amusa, M. and S. Van Vuuren, *Antimicrobial activity and toxicity of extracts from the bark and leaves of South African indigenous Meliaceae against selected pathogens.* South African Journal of Botany, 2020. **133**: p. 83-90.

198. Komakech, R., et al., *Root extract of a micropropagated Prunus africana medicinal plant induced apoptosis in human prostate cancer cells (PC-3) via caspase-3 activation.* Evidence-Based Complementary and Alternative Medicine, 2022. **2022**.

199. Nambooze, J., O.L. Erukainure, and C.I. Chukwuma, *Phytochemistry of Prunus africana and its therapeutic effect against prostate cancer.* Comparative Clinical Pathology, 2022. **31**(5): p. 875-893.

200. Asuzu, P.C., et al., *In Vitro Assessment of Efficacy and Cytotoxicity of Prunus africana Extracts on Prostate Cancer C4-2 Cells.* bioRxiv, 2021: p. 2021.03. 14.435338.

201. DERSO, S., *PHYTOCHEMICAL INVESTIGATION AND DETERMINATION OF ANTIBACTERIAL ACTIVITY OF CALPURNIA AUREA (DIGITA) SEED AND LEAF EXTRACTS*. 2020, SELAM DERSO.

202. Adedapo, A.A., et al., *Antibacterial and antioxidant properties of the methanol extracts of the leaves and stems of Calpurnia aurea.* BMC Complementary and alternative medicine, 2008. **8**: p. 1-8.

203. Dula, D. and A. Zelalem, *Antioxidant activity assessment of Calpurnia aurea root extract.* Natural Products Chemistry and Research, 2018. **6**(307): p. 2.

204. Melese, A., B. Dobo, and A. Mikru, *Antibacterial activities of Calpurnia aurea and Ocimum lamiifolium extracts against selected gram positive and gram-negative bacteria.* Ethiopian Journal of Science and Technology, 2019. **12**(3): p. 203-220.

205. Kamau, J., et al., *Anti-inflammatory activity of methanolic leaf extract of Kigelia africana (Lam.) Benth and stem bark extract of Acacia hockii De Wild in Mice.* J Dev Drugs, 2016. **5**(2): p. 1-8.

206. Guchu, B.M., et al., *Research Article In Vitro Antioxidant Activities of Methanolic Extracts of Caesalpinia volkensii Harms., Vernonia lasiopus O. Hoffm., and Acacia hockii De Wild.* 2020.

207. Kamau, K., *Antipyretic and Anti-Inflammatory Properties of Methanolic Extracts of Kigelia africana (Lam.) Benth and Acacia hockii de Wild in Animal Models*. 2016, School of Pure and Applied Sciences, Kenyatta University Nairobi, Kenya.

208. Meharie, B.G., G.G. Amare, and Y.M. Belayneh, *Evaluation of hepatoprotective activity of the crude extract and solvent fractions of clutia abyssinica (euphorbiaceae) leaf against CCl4-induced hepatotoxicity in mice.* Journal of Experimental Pharmacology, 2020: p. 137-150.

209. Zayede, D., T. Mulaw, and W. Kahaliw, *Antidiarrheal activity of hydromethanolic root extract and solvent fractions of clutia abyssinica jaub. & spach.(Euphorbiaceae) in mice.* Evidence-Based Complementary and Alternative Medicine, 2020. **2020**.

210. Jeruto, P., R. Nyangacha, and C. Mutai, *In vitro and in vivo antiplasmodial activity of extracts of selected Kenyan medicinal plants.* African Journal of Pharmacy and Pharmacology, 2015. **9**(16): p. 505-505.
