# Supplementary material for: Ethnobotanical study of medicinal plants used by the people of Mosop, Nandi County in Kenya
Source: Front Pharmacol. 2024 Jan 19;14:1328903. doi: 10.3389/fphar.2023.1328903 (PMC10834697; doi:10.3389/fphar.2023.1328903)
Supplement: Supplementary file 2 [file Table3.DOC]

**Supplementary table S1 for medicinal plant diversity**

**Table S1**: List of medicinal plants used to ailments: scientific name; plant family; vernacular name; growth form; plant parts used; ailment treated; condition of use; methods of preparation; routes of administration and additives.

**Key**: **Vernacular name**: Nandi dielect. **Growth form** (GF) - Tree (T); Shrub (S); Small tree (ST); Herb (H); Climber (C), ; Liana (L); Epiphyte (E). **Part used** (PU) -Leaf, L; Root, R; Fruit, Fr; Bark, B; Stem, St; Root bark, Rb; Flower, Fl; Bulb, Bu; Seed, Se; Tuber, Tu; Rhizome, Rh; Latex, Lat; Pods;P; Peel,PL; Tender leaves,TL; Twigs,Tw; Berries, Be; Inflorescence,IF; Shoot,Sh;Gum,G; Whole plant, WP; Aerial part,AP & Stalks,Sk. **Condition of use** (CU) - Dry (D), Fresh (F). **Methods of preparation** (MOP): Extract –Macerated/pounded/pulverized little water added; Juice- Crushed & Squeezed; Infusion- Macerated/pounded/pulverized/powder soaked in hot or cold water and filter; Decoction-boiled in water and filtered; Paste-Crushed. Routes of administration (ROA). **IUCN** **Status:** - Endangered-**EN**; Near Threatened-**NT**; Data Deficient-**DD**; Vulnerable-**VU**; Least Concern -**LC**; Not Evaluated-**NE** & Critically Endangered-**CE**. **Poisonous plant

| **No** | **Scientific name /**  **Voucher No.** | **IUCN**  **Status** | **Vernacular name (Local language)** | **GF** | **PU** | **Ailment treated (Local name)** | **CU** | **MOP** | **ROA** | **Additives** | **Family** |
| --- | --- | --- | --- | --- | --- | --- | --- | --- | --- | --- | --- |
|  | *Clerodendrum myricoides*R.Br. & Vatke  (MNCY- 238) | NE | Abetiot/Kabetiot | S/ST | R | Chest pains, cleaning the bowel, poisoning to induce vomiting | F | Decoction | Oral | Mutton soup | Lamiaceae |
|  |  |  |  |  |  | colds, stop gum-bleeding | F | Infusion | Oral |  |  |
|  |  |  |  |  |  | Gonorrhea, Indigestion, sore throat, tonsillitis, malaria and rheumatism | F | Extract/Juice | Oral |  |  |
|  |  |  |  |  | B | Snakebites, reduce bodily swellings and relief indigestion | D | Powder | Topical |  |  |
|  | *Lantana trifolia* L.  (MNCY- 232) | NE | Baiwaptarit | S | L | Cough, common colds, flu,sore throat, induce labour, gonorrhea, menstrual pains, heart problems, tuberculosis, skin rashes (measles); other skin rashes and dizziness | F | Extract | Oral |  | Verbenaceae |
|  | *Trichocladus ellipticus Eckl. & Zeyh.*  (MNCY- 138) | LC | Barekeiwet | T |  | Bone fractures | F | Paste | Topical (Tied) | Ghee |  |
|  |  |  |  |  |  | Ears and eye infections e.g. trachoma, conjunctivitis and otitis; ringworms | F | Juice | Drops |  |  |
|  |  |  |  |  |  | Fever, anaemia | F | Decoction | Oral |  |  |
|  |  |  |  |  | L & R | Stomachaches | F | Decoction | Oral |  |  |
|  |  |  |  |  | L | Toothache | F | Steam-Squeeze Juice/Paste | Apply on tooth |  |  |
|  |  |  |  |  | L | Hepatic diseases | F | Decoction/Juice |  |  |  |
|  |  |  |  |  | L/FL | Oral care | D | Powder | Brush/Apply on tooth | Salt |  |
|  |  |  |  |  | L | Eye infection, gland disorders | F | Infusion | Oral |  |  |
|  |  |  |  |  | R | Eczema, colic, erectile dysfunction, STI’s and peptic ulcers | F | Decoction | Oral |  |  |
|  |  |  |  |  | R | Rheumatism, general body pains, indigestion | F | Infusion | Oral |  |  |
|  |  |  |  | T | B | Stomach evacuation, inflammations & indigestion | F | Decoction | Oral | Meat/  Soup | Hamamelidaceae |
|  |  |  |  |  | R/B | Oral thrush | D | Powder | Apply |  |  |
|  | *Afrocarpus falcatus*(Thunb.) C.N.Page  (MNCY- 20) | LC | Biribiriet | T | B | Relive pain, diarrhoea, rheumatism and stomachache | F | Decoction/Infusion | Oral |  | Podocarpaceae |
|  |  |  |  |  | Sb | Itching rash | F | Decoction | Apply |  |  |
|  |  |  |  |  | Se | TB, meningitis & sunburn | F | Pulverized/ Decoction | Oral/Apply |  |  |
|  |  |  |  |  | S | Chest complaints | F | Sap | Oral |  |  |
|  |  |  |  |  | B | Fevers, asthma, coughs, cholera, chest complaints, arthritis, rheumatism, hypertension venereal diseases & jaundice | F | Decoction | Oral |  |  |
|  |  |  |  |  | Lt | Rheumatism | F | Decoction | Bath |  |  |
|  |  |  |  |  | Se/Fr | Gonorrhoea | F | Oil | Oral |  |  |
|  |  |  |  |  | R | Cancer | D | Powder-Infusion | Oral |  |  |
|  |  |  |  |  | Rb | pain killer, haemorrhoids | F | Decoction | Oral |  |  |
|  | ***Phytolacca dodecandra*L'Hér. (MNCY- 96) | NE | Botkawet | S/C | R | Roundworms "Magargarek”, induce vomiting; expel tapeworms & bowel evacuation | F | Extract | Oral |  | Phytolaccaceae |
|  |  |  |  |  | L | Cancerous wounds “seriat”. | F | Extract | Apply |  |  |
|  |  |  |  |  | L/R/  Br | Wounds for skin diseases such as ringworms, eczema, scabies, boils and loss of skin colour in patches | F | Sap/Paste | Apply |  |  |
|  |  |  |  |  | Br/R | Rabies, malaria, sore throat, jaundice, epilepsy and respiratory problems. | F | Infusion | Oral |  |  |
|  |  |  |  |  | L | Asthma, oedema, diarrhea, abdominal pain, tuberculosis, eye infections, ear infections, malaria, sore throat, bowel evacuation & tuberculosis | F | Decoction | Oral |  |  |
|  |  |  |  |  | Sh/Tl | Induce abortion | F | Juice | Oral |  |  |
|  |  |  |  |  | Rb/L | Epilepsy | F | Maceration-extract | Oral |  |  |
|  |  |  |  |  | L | Ear infections | F | Sap | Drops |  |  |
|  |  |  |  |  | R | Enlarged glands, syphilis |  | Decoction | Oral |  |  |
|  | *Terminalia schimperiana*Hochst. ex Engl. & Diels  (MNCY- 63) | LC | Bukwet | H | R | Wounds, burns, ulcers & skin diseases | F | Extract | Apply |  | Combretaceae |
|  |  |  |  |  |  | Epilepsy | D | Powder-Infusion | Oral |  |  |
|  |  |  |  |  |  | Malaria, liver complications & coughs; gastrointestinal disorders, kidney pains, gonorrhea, headache, backache; boost urination in men with swollen prostate gland | F | Decoction | Oral |  |  |
|  |  |  |  |  | R | Oral and dental care; clears the bowel, to restore general body strength; aphrodisiac, stimulant or depressant & treating venereal diseases | F | Extract | Oral |  |  |
|  |  |  |  |  | B | Induce vomiting,malaria, pain killer & worm infections | F | Decoction | Oral |  |  |
|  |  |  |  |  | L | Burns, malaria, headaches, stomachache, diarrhoea, dysentery, liver diseases, amenorrhea, cough, asthma, diabetes, obesity &swollen feet. |  | Decoction | Oral |  |  |
|  |  |  |  |  | Fr | Anthelminthic | F | Extract | Oral |  |  |
|  |  |  |  |  | L | Scabies, cough, heart conditions | F | Sap | Oral |  |  |
|  |  |  |  |  | B/R | Aphrodisiac | F | Decoction | Oral |  |  |
|  | *Crassocephalum vitellinum*S.Moore (MNCY- 74) | NE | Busariat | H | WP | Peptic ulcers, liver protection, stomach complications, mouth infections in children, & malaria | F | Decoction | Oral |  | Asteraceae |
|  |  |  |  |  | L | Swollen legs | D | Ash | Apply |  |  |
|  |  |  |  |  |  | Female sterility, strong fever, menstrual cramps, constipation, childhood diseases & expulsion of the placenta; fever, constipation, abortion, gonorrhea & facilitate lactation |  | Decoction | Oral |  |  |
|  |  |  |  |  | L/FL | Whooping cough | F | Extracts | Oral |  |  |
|  |  |  |  |  | L | Feeding disorder, antenatal care | F | Extracts | Oral |  |  |
|  |  |  |  |  | WP | Cough | D | Ash | Oral |  |  |
|  | *Lactuca glandulifera*Hook.f. (MNCY- 216) | NE | Chebara | H | B | Dysentery & malaria |  | Decoction | Oral |  | Asteraceae |
|  |  |  |  |  | L/FL | Stimulate menstrual flow, sinusitis & haemorrhoids | F | Infusion | Oral |  |  |
|  |  |  |  |  | R | Gonorrhoea or syphilis | F | Decoction | Oral |  |  |
|  | *Sonchus asper*(L.) Hill (MNCY- 227) | NE | Chebara katet |  | WP | Wounds, swellings & boils | F | Paste | Poultice |  | Asteraceae |
|  |  |  |  |  | L | Ulcers | F | Extract/Juice | Apply |  |  |
|  |  |  |  |  | Lat | Warts | F | Extract | Apply |  |  |
|  |  |  |  |  | R | Cough, bronchitis, asthma & pertussis | F | Extract | Oral |  |  |
|  |  |  |  |  | R/L | Indigestion, reduce fever, blood purifier, liver issues, malaria, restore body strength, cancer, inflammation, antidepressant & against helminthes. | F | Decoction | Oral |  |  |
|  | *Eucalyptus globulus* Labill.  (MNCY- 248) | LC | Chebarusyot ne leel | T | L | Asthma, fever, flu, whooping cough, loss of appetite, irritable bowel complaints, inflammatory and infectious diseases of kidneys and bladder, diabetes, rheumatic complaints | F | Decoction | Oral |  | Myrtaceae |
|  |  |  |  |  |  | Wounds, acne, poorly healing ulcers, bleeding gums, rheumatism, severe nerve pain, gonorrhoea & sore inflammation inside the mouth | F | Extract | Apply/Oral |  |  |
|  |  |  |  |  |  | Respiratory tract, bronchial asthma; insect repellant, air freshening | F | Steam Bath/massage | Inhalation |  |  |
|  |  |  |  |  | L/R | Headache, stomach ache, bronchial infections, cough, asthma, sore throat, cold, fever; gastrointestinal tract wounds, respiratory and urinary system infectiins; fungal infections, diabetes, rheumatism, headache, backache, inflammations & sepsis | F | Decoction | Oral |  |  |
|  |  |  |  |  | Sh/L | Chicken pox | F | Decoction | Bath |  |  |
|  |  |  |  |  | L | Flu, fever or colds | F | Steam | Inhalation |  |  |
|  | *Nicoteba betonica*(L.) Lindau  (MNCY- 4) | NE | Cheberenet-neo | H | IF | Vomiting, constipation, hair wash | F | Extract | Oral/wash |  | Acanthaceae |
|  |  |  |  |  | L | Abscesses, relieve pain & swelling | F | Poultice | Apply |  |  |
|  |  |  |  |  | L/FL | Cough | D | Ash | Leak |  |  |
|  |  |  |  |  | WP | Vomiting, headache due to malarial symptoms, constipation, diarrhea, malaria, inflammed testacles, pain, snake bite, stomachache, vomiting, respiratory, GIT complaints, inflammatory disorders, malaria, fever, epilepsy, rheumatism, headache, cancer, diabetes, mental disorders & arthritis | F | Decoction | Oral |  |  |
|  |  |  |  |  | R | Lower cholesterol, treat paralysis, earaches, headaches, bruises, diarrhea, vomiting, constipation, pain and inflammation & malaria | F | Decoction | Oral |  |  |
|  |  |  |  |  | R | Snake bite | F | Extract | Apply |  |  |
|  |  |  |  |  | L | Malaria, alleviate weakness in pregnancy | F | Extract | Bath |  |  |
|  |  |  |  |  | R | Hernia, worm infection, toothache & malaria | F | Infusion | Oral |  |  |
|  |  |  |  |  |  | Cough remedy | F | Roast-Extract | Oral |  |  |
|  | *Vachellia nilotica* subsp. *tomentosa*(Benth.) Kyal. & Boatwr.  (MNCY- 205) | LC | Chebitet | T | B | Throat problems | F | Decoction | Gargle |  | Fabaceae |
|  |  |  |  |  |  | Bladder inflammation, inflammation leading to discharge, abnormal uterine bleeding, asthma, skin eruptions in children, toxemia & bronchitis. Cold, bronchitis, diarrhoea, dysentery, passing gas, bad digestion, constipation, stomach pain, bleeding haemorrhoids, depigmentation due to total loss of melanin, sooth and moisturize the skin, anthelmintic, aphrodisiac, promote urination, expectorant, induce vomiting, hemorrhage, wound ulcers, skin diseases, burning sensation, toothache, dysentery and seminal weakness. | F | Decoction | Oral |  |  |
|  |  |  |  |  | R | Cancers and/or tumors (of ear, eye, or testicles), TB, hardening of soft tissues around the abdomen, breasts, buttocks, chest, liver & spleen |  | Decoction | Oral |  |  |
|  |  |  |  |  | B | Eye infections | F | Juice | Drops | Breast milk |  |
|  |  |  |  |  | P | Impotency, involuntary emission of semen & urogenital disorders | D | Powder | Leak |  |  |
|  |  |  |  |  |  | Sexual disorders-spermatorrhoea, frequent night discharges and premature ejaculation | F | Extract | Oral |  |  |
|  |  |  |  |  | B | Gums, strengthens the teeth and manage bleeding gums | F | Paste | Apply |  |  |
|  |  |  |  |  | L | Reduce bleeding from minor cuts & bowels | F | Decoction | Oral |  |  |
|  |  |  |  |  | P/B | Bleeding haemorrhoids | F | Decoction | Oral |  |  |
|  |  |  |  |  | P/L | Diarrhoea | F | Extract | Oral |  |  |
|  |  |  |  |  | FL | Restore body strength after episodes of diarrhoea and dysentery | F | Infusion | Oral |  |  |
|  |  |  |  |  | Fr | Sore throat, cold, bronchitis, pneumonia, eye infections, diarrhoea, dysentery, leprosy and venereal diseases. | F | Decoction | Oral |  |  |
|  |  |  |  |  | L | Diarrhea, aphrodisiac, inflammation | F | Extract | Oral |  |  |
|  |  |  |  |  | L | Dressing ulcers | F | Paste | Apply |  |  |
|  |  |  |  |  | L/B | Colds and pneumonia, reduce fevers and stop excessive bleeding | F | Teas | Oral |  |  |
|  | *Ajuga integrifolia*Buch.-Ham. ex D.Don  (MNCY- 54) | NE | Chelelgatiat | H | WP | Stomachache, malaria, stops diarrhoea, malaria, boils; diarrhea, stomach disorders, evil eye, retained placenta, ascariasis, malaria, swollen legs, hypertension, jaundice and wounds. | F | Decoction | Oral | Soup | Lamiaceae |
|  |  |  |  |  | L | Mouth ulcers, mild toothache, malaria, inflammation, diarrhoea, stomach disorders, retained placenta, ascariasis, swollen legs, hypertension, jaundice and wounds | F | Extract | Chew-Oral |  |  |
|  |  |  |  |  |  | Diabetes, hypertension, fever, stomach pain and malaria | F | Decoction | Oral |  |  |
|  |  |  |  |  |  | Fever, toothache, dysentery, amoeba and high blood pressure | F | Infusion | Oral |  |  |
| 1. 14 | *Bridelia micrantha*(Hochst.) Baill.  Baill. (MNCY-45) | LC | Chemagaldet | T | B | Stomachache & tapeworm deworming | F | Decoction | Oral |  | Phyllanthaceae |
|  |  |  |  |  |  | Stop diarrhoea and regain body strength |  | Decoction | Oral | Soup |  |
|  |  |  |  |  |  | Bloody diarrhoea | F | Infusion | Oral |  |  |
|  |  |  |  |  | R | Headaches | F | Cream | Apply | Ghee |  |
|  |  |  |  |  |  | Aching joints | F | Decoction | Oral |  |  |
|  |  |  |  |  | B | Body strength and general health | F | Decoction | Oral | Soup & milk |  |
|  | ***Emilia discifolia*(Oliv.) C.Jeffrey** (MNCY- 165) | NE | Chemamaiyat | H | WP | Syphilis, vomiting, anaemia, prevention of miscarriage, induction of uterine contractions, expulsion of retained placenta and clearing contents of the conceptus after birth | F | Decoction | Oral |  | Asteraceae |
|  |  |  |  |  |  | Disinfect umbilical cord/wounds | F | Extract | Apply |  |  |
|  |  |  |  |  | WP | Eye infections | F | Extract | Drops |  |  |
|  |  |  |  |  |  | Relieve stomachaches, chronic asthma and ring worms | F | Extract | Oral |  |  |
|  |  |  |  |  | WP | Stimulate lactation | F | Decoction | Oral |  |  |
|  |  |  |  |  | L/R | Stomachaches in pregnant women | F | Decoction | Oral |  |  |
|  |  |  |  |  | L | Sore eyes | F | Roast-Juice | Drops |  |  |
|  |  |  |  |  | L | Intestinal worms | F | Infusion | Oral |  |  |
|  | *Ziziphus mucronata*Willd.  (MNCY- 251) | LC | Chemanambelio | T | R | Gonorrhea, diarrhea, gland swellings, measles, low back pain and dysentery | F | Infusion | Oral |  | Rhamnaceae |
|  |  |  |  |  | R/L | Sores and glandular swellings | F | Decoction | Apply-massage |  |  |
|  |  |  |  |  | B/R | Rheumatism, gastrointestinal complaints and snake bites | F | Decoction | Oral |  |  |
|  |  |  |  |  | R | Relieve all sorts of pains as well as dysentery, to prevent obesity and relief off hunger pangs; snake bites, diabetes, cancer, anxiety, asthma, diarrhea, fatigue, fever, high blood pressure, inflammation and stress. | F | Decoction | Oral |  |  |
|  |  |  |  |  | R/L | Respiratory ailments and other septic swellings of the skin | F | Decoction | Oral |  |  |
|  |  |  |  |  |  | Boils, swollen glands, wounds and sores |  | Paste | Apply |  |  |
|  |  |  |  |  | B | Purify and improve skin appearance | F | Steam | Bath |  |  |
|  |  |  |  |  | L | Treat depression, as an expectorant for cough, respiratory infections and chest problems | F | Decoction | Oral |  |  |
|  |  |  |  |  | B | Relieving body pains & enlarged spleen | F | Infusion | Oral |  |  |
|  |  |  |  |  |  | Boils and other skin infections | D | Baked-Powder | Poultice |  |  |
|  |  |  |  |  |  | Rheumatism and stomach troubles | F | Decoction | Oral |  |  |
|  | *Pavonia kilimandscharica*  Gürke  (MNCY- 160) | NE | Chemanjililiet | S | R | Abdominal problems, malaria, rheumatism, pains in the flesh, pneumonia, rheumatism, ringworms, STDs, stomach disorders, malaria, fever, TB | F | Decoction | Oral |  | Malvaceae |
|  |  |  |  |  | R | Sore throat and expel a retained placenta after birth | F | Extract | Oral |  |  |
|  |  |  |  |  | L | Cuts | F | Extract | Oral |  |  |
|  |  |  |  |  |  | Relief stomachaches in children | F | Infusion | Oral |  |  |
|  |  |  |  |  | R/L | Wounds | D | Ash | Apply-Paste |  |  |
|  |  |  |  |  |  | Stomachache and hypertension | F | Decoction | Oral |  |  |
|  |  |  |  |  | R | Coughs, sore throat, colds and snake bites | D | Infusion | Oral |  |  |
|  | *Tragia brevipes*Pax (MNCY- 52) | NE | Chemelet | H/L | WP | Headache | D | Ash | Apply |  | Euphorbiaceae |
|  |  |  |  |  |  | Relief pain and rheumatism, peptic ulcers, diabetes, stomach problems, relief chain pains, stomachache, cough, oedema, diarrhea, fever, headache, toothache remedies and malaria; male impotence | F | Decoction | Oral |  |  |
|  |  |  |  |  | L | Headaches | F | Scent | Inhalation |  |  |
|  |  |  |  |  | R | Snake bites | D | Powder | Apply |  |  |
|  |  |  |  |  | L | Relieve skin irritation caused by stinging hairs of plants | F | Infusion | Wash |  |  |
|  |  |  |  |  | R | Headaches | F | Pulverized | Rub |  |  |
|  |  |  |  |  |  | Induce vomiting, treat uterine complaints and increase uterine contraction during labour | F | Decoction | Oral |  |  |
|  |  |  |  |  |  | Contraceptive | F | Infusion | Oral |  |  |
|  |  |  |  |  | R/L | Topical local anesthetic agent | F | Decoction | Apply |  |  |
|  | *Allophylus racemosus*Sw. (MNCY- 161) | LC | Chemnoet | ST | R | Stomach and back pains | F | Decoction | Oral |  | Sapindaceae |
|  |  |  |  |  | L | Evil protection | D | Ash | Rub |  |  |
|  |  |  |  |  | L | Head pains & body pains | F | Paste | Dressing |  |  |
|  |  |  |  |  | L | Asthma and bronchitis; kidney pains and hernia | F | Paste | Poultice |  |  |
|  |  |  |  |  | L/Tw | Stomach trouble, abdominal pain, headache, toothache, asthma, bronchitis and inflammation | F | Decoction/Infusion | Oral |  |  |
|  |  |  |  |  | L | Asthma, headache and rheumatism | D/F | Smoke/Fumes | Inhalation |  |  |
|  | *Leucas deflexa*Hook.f. *(*MNCY- 104) | NE | Chemul chuchuniat | H | WP | Headaches | D | Ash | Rub |  | Lamiaceae |
|  |  |  |  |  |  | Pain relief agent,rheumatism, peptic ulcers, diabetes, stomach problems, relief chain pains, stomachache, cough, diarrhea, fever, headache and toothache remedies and malaria | F | Decoction | Oral |  |  |
|  |  |  |  |  | R | Headache, skin rushes and boils | F | Paste | Apply |  |  |
|  |  |  |  |  |  | Inflammations, coughs, colds and flu | F | Decoction | Oral |  |  |
|  | *Leucas martinicensis*(Jacq.) R.Br.  (MNCY- 71) | NE | Chemul chuchuniat | H | WP | Painful menstruation, wounds, fever, stimulate lactation and stomach pain | F | Decoction | Oral |  | Lamiaceae |
|  |  |  |  |  | WP | GIT disturbances, cholera, malaria, syphilis and diarrhea | F | Infusion | Oral |  |  |
|  |  |  |  |  | L | Convulsions, asthma, a sedative; antiseptic & stop vomiting | F | Extract | Oral |  |  |
|  |  |  |  |  |  | Repellant of mosquito and insect bites due to minty odour | F | Extract | Scent/Apply |  |  |
|  | *Urena lobata* L.  (MNCY- 89) | LC | Chemulmeswo | S | L | Asthma, fever, flu, whooping cough, loss of appetite, stomach complaints, kidney problems, urinary tract infections, diabetes, and rheumatic complaints | F | Decoction | Oral |  | Malvaceae |
|  |  |  |  |  | L | Wounds, acne, poorly healing ulcers, bleeding gums, rheumatism, severe nerve pain, gonorrhoea and sore inflammation inside the mouth | F | Extract | Apply/Oral |  |  |
|  |  |  |  |  | L | Disorders of respiratory tract and bronchial asthma, body massage and insect repellant | F | Steam | Bath/Scent/  Inhalation |  |  |
|  |  |  |  |  | L/R | Headache, stomachaches, chest infections, coughs, asthma, oral cavity ulcers, cold, fever; wounds of the gastrointestinal tract, respiratory and urinary system | F | Decoction | Oral |  |  |
|  |  |  |  |  | R | Skin infections, diabetes, backache, rheumatism, headache, sore throat, inflammations and sepsis | F | Decoction | Oral |  |  |
|  |  |  |  |  | Tl/L | Chicken pox | F | Decoction | Bath |  |  |
|  |  |  |  |  | L | Flu, fever or colds | F | Steam | Inhalation |  |  |
|  | *Mesosphaerum pectinatum*(L.) Kuntze  (MNCY- 152) | NE | Chemulngariet | H | WP | Asthma, coughs, chest pains, difficulty in breathing and tuberculosis | F | Decoction | Oral |  | Lamiaceae |
|  |  |  |  |  | AP | Alleviate liver problems and aphrodisiac to build sexual stamina in men | F | Decoction | Oral |  |  |
|  |  |  |  |  | WP | Diarrhoea | D | Powder-Infusion | Oral |  |  |
|  |  |  |  |  | L | Cleansing the blood | F | Infusion | Oral |  |  |
|  |  |  |  |  | L | Oral thrush and persistent sores | F | Decoction | Oral |  |  |
|  |  |  |  |  | L | Soothe the skin and heal chronic wounds | F | Paste | Massage |  |  |
|  |  |  |  |  | L | Insect repellent | F | Smoke | Inhalation |  |  |
|  | *Justicia ladanoides*Lam.  (MNCY- 78) | NE | Chemurguiwet | H | L | Manage internal parasites | F | Decoction | Oral |  | Acanthaceae |
|  |  |  |  |  | L | Cold and chest pain | F | Infusion | Oral |  |  |
|  |  |  |  |  | R | Snake and other insect bites | F | Extract | Oral |  |  |
|  |  |  |  |  | L | Oral cavity-gums | F | Extract | Rub |  |  |
|  |  |  |  |  | WP | Spleen diseases, rheumatism, gout and joint pains | D | Ash | Leak |  |  |
|  |  |  |  |  | L | Cold, coughs and chest pain | F | Infusion | Oral |  |  |
|  | *Dyschoriste hildebrandtii*Lindau ex C.B.Clarke  (MNCY- 59) | NE | Chemurguiwet | H | L | Malaria | F | Infusion | Oral |  | Acanthaceae |
|  |  |  |  |  | WP | Chest congestion, coughs, colds, flu, reduce fever and detoxification | F | Decoction | Oral |  |  |
|  |  |  |  |  |  | Appetizer and stomach complaints | F | Infusion | Oral |  |  |
|  |  |  |  |  | AP | Diarrhea, wound healing and anti-inflammatory management | D | Decoction | Oral |  |  |
|  |  |  |  |  | FL/L | Boils and headaches | F | Paste | Poultice |  |  |
|  |  |  |  |  | FL | Jaundice and to stimulate menstruation | F | Decoction | Oral |  |  |
|  |  |  |  |  | R | Snake bite treatment | F | Decoction | Oral |  |  |
|  |  |  |  |  |  | Antiseptic- wounds | F | Decoction | Wash |  |  |
|  | *Dyschoriste radicans* (Hochst. ex A.Rich.) Nees  (MNCY- 11) | NE | Chemurguiwetab suswek | H | L | Cold, coughs and chest pain | F | Infusion | Oral |  | Acanthaceae |
|  |  |  |  |  | L | Malaria | F | Infusion | Oral |  |  |
|  |  |  |  |  | WP | Chest congestion, coughs, colds, flu, reduce fever and detoxification | F | Decoction | Oral |  |  |
|  |  |  |  |  |  | Appetizer and stomach complaints | F | Infusion | Oral |  |  |
|  |  |  |  |  | AP | Diarrhea, wound healing and anti-inflammatory management | D | Decoction | Oral |  |  |
|  |  |  |  |  | FL/L | Boils and headaches | F | Paste | Poultice |  |  |
|  | *Spermacoce princeae* (K.Schum.) Verdc.  (MNCY- 226) | NE | Chemurguiywet | H | WP | Wounds, sores, ulcerations and general skin diseases | F | Paste | Apply | Ghee | Rubiaceae |
|  |  |  |  |  | L | Liver diseases | F | Decoction | Oral |  |  |
|  |  |  |  |  | L/R | Chronic asthma, cancer, wounds, eye problems, STDs, skin diseases, pneumonia, typhoid, caterpillar bites and diarrhoea | F | Extracts | Oral |  |  |
|  |  |  |  |  | St/L | Female infertility | F | Macerate-Infusion | Oral |  |  |
|  |  |  |  |  | L | Kidney diseases | F | Paste | Oral | Salt & ghee |  |
|  | *Spermacoce senensis*(Klotzsch) Hiern  (MNCY- 32) | NE | Chemurguiywet | H | WP | Haemorrhoids, treating anal infections, UTIs and digestive problems | F | Infusion | Oral |  | Rubiaceae |
|  |  |  |  |  |  | Malaria, digestive problems, fever, hemorrhage, urinary and respiratory infections, skin diseases and headaches | F | Decoction | Oral |  |  |
|  |  |  |  |  | Se | Stomach complaints, indigestion and constipation | F | Paste | Oral |  |  |
|  | *Markhamia zanzibarica*(Bojer ex DC.) K.Schum.  MNCY- 195) | NE | Chepbartanyit | S/ST | B | Syphilis | D | Powder-Decoction | Oral |  | Bignoniaceae |
|  |  |  |  |  | R/B | Abdominal pains/ bleeding, anemia, diarrhea, backache, sore eyes, pain, blood/circulatory diseases, gout, swollen male reproductive organ, TB, infection, convulsion in children and oedema in pregnancy | F | Decoction | Oral |  |  |
|  |  |  |  |  | L | General body aches, leucorrhea and swellings around the anus. | F | Decoction | Oral |  |  |
|  | *Casearia battiscombei*R.E.Fr. (MNCY- 210) | NE | Chepchabaitet | T | R | Abdominal pain in adults |  | Decoction | Oral |  | Salicaceae |
|  |  |  |  |  | R/B | Malaria, stomach troubles, cough, relieve body pain, TB, dysentery and diarrhea | F | Decoction | Oral |  |  |
|  |  |  |  |  | L | Skin lesions, snake bites and small ulcerations | F | Extracts | Oral |  |  |
|  |  |  |  |  |  | Inflammatory pain | F | Decoction | Oral |  |  |
|  | *Barleria grandicalyx* Lindau  (MNCY- 40) | LC | Cheperenet | H | L | Snake bite | F | Chewed/ pulped- Paste | Rub |  | Acanthaceae |
|  |  |  |  |  |  | Cancer wounds | F | Extract | Oral |  |  |
|  |  |  |  |  |  | Infant bellybutton/navel treatment | F | Crush-Extract | Apply |  |  |
|  |  |  |  |  |  | Backache, skin diseases, cough, eye infections and wounds | F | Infusion | Oral |  |  |
|  |  |  |  |  | R | Throat infection, pneumonia, diarrhea, ulcers, flu, dry cough, edema, chest diseases, rheumatism, fever, asthma, pneumonia, tuberculosis, expectorant & to reduce swelling. | F | Decoction | Oral |  |  |
|  |  |  |  |  | R/L | Throat cancer | F | Chew-Extract | Oral |  |  |
|  | *Ocimum kilimandscharicum*  Gürke  (MNCY- 191) | LC | Chepikis | H | L | Measles and rashes | F | Infusion | Oral |  | Lamiaceae |
|  |  |  |  |  |  | Asthma, eye infections, halitosis/bad oral breath, eating poorly/anorexia, headaches, sinus problems and wounds | F | Extract | Oral |  |  |
|  |  |  |  |  |  | Foul body smell, skin diseases, ringworms, scabies, distention of the abdomen, ulcers wounds & stomachaches | F | Decoction | Oral |  |  |
|  |  |  |  |  | R/L | Flatulence with gurgling sound, digestive impairment, rheumatism, tastelessness, diarrhoea, burning sensation, toothache, chronic sinusitis, itching, disease of throat, male impotence, joint pain, helminthiasis/worm infestation, diseases of skin, obesity, asthma, thirst, skin diseases, eczema, disorders due to poison, stomach disorder with piercing pain urinary tract infections | F | Decoction | Oral |  |  |
|  |  |  |  |  | L | Wounds | D | Powder | Apply |  |  |
|  |  |  |  |  |  | Breasts to ease lactational pain/discomfort | F | Juice | Rub |  |  |
|  |  |  |  |  |  | Congested chest, bronchitis, fever and flu | F | Steam | Inhalation |  |  |
|  | *Chrysanthemum americanum*(L.) Vatke ex Weberl. & Lagos  (MNCY- 189) | NE | Chepilibiliot | H | WP | Relieve constipation; disturbances of blood circulation and oedema of the lower limbs, varicose veins and haemorrhoids, chronic disorders of liver associated with heavy drinking/alcoholism, hepatitis; acute episode of malaria with haemolysis (jaundice), poor digestion of lipids (fats) and blood cholesterol. | F | Decoction | Oral |  | Asteraceae |
|  | ***Noronhia mildbraedii*(Gilg & G.Schellenb.) Hong-Wa & Besnard** (MNCY-99) | NE | Chepitet | T | B | Nose-bleeding, complaints of gas, nausea and abdominal discomfort after meals and chronic diarrhea, jaundice and chronic weakness | F | Decoction | Oral |  | Oleaceae |
|  |  |  |  |  | Rb | Strengthen organs specifically the pancreas and spleen | F | Decoction | Oral |  |  |
|  |  |  |  |  |  | Wounds, inflammations, sores and infections | F | Teas/Poultice | Wash |  |  |
|  |  |  |  |  |  | Treat migraine, headache, depression; urinary tract complaints, restore normal health, increases the flow of bile, laxative and a tonic. | F | Chew-Extract | Oral |  |  |
|  |  |  |  |  |  | Treat liver diseases like jaundice and general function, complaints of gas, nausea and abdominal discomfort after meals and other ailments related to poor liver function | F | Infusion | Oral |  |  |
|  | *Celtis africana* Burm.fil.  (MNCY- 22) | LC | Chepkeleliet | S | L/B | Oral thrush | G | Ash | Apply | Ghee | Cannabaceae |
|  |  |  |  |  | R | Threatened abortion, convulsions, influenza, and enlarged spleen and for the management of habitual miscarriage | F | Extract | Oral |  |  |
|  |  |  |  |  |  | Stomachache, cold, cough, bronchitis, nose block and chest pains | F | Infusion | Oral |  |  |
|  |  |  |  |  |  | Genital ulcers associated with STIs | D | Powder | Apply |  |  |
|  |  |  |  |  |  | Influenza and for habitual abortion, dizziness, headache, malaria and stomach-ache | F | Decoction | Oral |  |  |
|  |  |  |  |  |  | Headache and stomachache | F | Decoction | Oral | Soup |  |
|  |  |  |  |  |  | Malaria | F | Steam | Inhalation | Soup |  |
|  |  |  |  |  |  | Influenza, indigestion | F | Extract | Oral |  |  |
|  |  |  |  |  |  | Liver pains |  | Decoction | Oral | Milk |  |
|  |  |  |  |  | R/L | Chest pains, enlarged spleen and shortness of breath | F | Decoction | Oral |  |  |
|  |  |  |  |  | L | Hepatitis, malaria, headaches, stomach problems, inflammation and sores | F | Decoction | Oral |  |  |
|  | *Amaranthus caudatus* L.  (MNCY- 94) | NE | Chepkerta ne birir | H | R | Diabetes, cancer, malaria, heart diseases cholesterol & fat complications, laxative for children, helminthic infestations, wound infections, inflammation, hepatic diseases & cardiovascular complications | F | Decoction | Oral | Honey | Amaranthaceae |
|  |  |  |  |  | L | Urogenital infections, ulcers, diarrhoea, inﬂammation of the mouth and throat | F | Decoction | Oral |  |  |
|  |  |  |  |  | Se | Laxative; relieve pain, blood purifier, tapeworms, jaundice, amoebic dysentery, relieving pulmonary conditions and breasts complaints. | F | Teas | Oral |  |  |
|  |  |  |  |  | L | Applied to piles and sore, treating eye diseases, abscesses, boils and eczema. | F | Extracts | Apply |  |  |
|  | *Centella Coriacea* Nannf.  (MNCY- 178) | NE | Chepketeryot | H | L | Salve on burns | D | Powder-Paste | Apply | Oil | Apiaceae |
|  |  |  |  |  |  | Pneumonia | F | Extract | Nose drops |  |  |
|  |  |  |  |  |  | Rubbed on the gums to treat toothache and on the skin to treat fungal problems | F | Extract | Rub |  |  |
|  |  |  |  |  | Tw | Chest pain, side pain and shortness of breath | F | Sap | Oral |  |  |
|  |  |  |  |  | L/Tw | Skin problems, diarrhea and tachycardia | F | Infusion | Oral |  |  |
|  |  |  |  |  | WP | Menstrual pains | D | Smoked | Inhalation |  |  |
|  | *Ozoroa insignis* Delile (MNCY- 77) | LC | Chepkitong'yot | S/ST | Sb | Peptic ulcers and malaria | F | Decoction | Oral |  | Anacardiaceae |
|  |  |  |  |  | R/Sb | Kidney and liver complaints; ulcers and hernias; throat infections; chest pain; diarrhoea and worm infestations | F | Decoction | Oral |  |  |
|  |  |  |  |  | R | Diarrhea, venereal diseases, tapeworms, kidney trouble, migraine headaches and malaria. | F | Decoction | Oral |  |  |
|  |  |  |  |  |  | Promote lactation. | F | Infusion | Oral |  |  |
|  | *Senegalia senegal* (L.) Britton  (MNCY- 162) | NE | Chepkomon | S/ST | B | Diarrhoea and stomach disorders. | F | Decoction | Oral |  | Fabaceae |
|  |  |  |  |  | R | Relief constipation, stomachache, indigestion, treatment of STI’s specifically gonorrhoea, clear mucus at the back of the nose, the throat or the sinuses, colds, coughs, diarrhea, dysentery, expectorant, hemorrhage, sore throat, typhoid, urinary tract. | F | Decoction | Oral |  |  |
|  |  |  |  |  | L | Wound dressing and eye infections. | F | Paste | Poultice-Apply |  |  |
|  |  |  |  |  | G | Treat minor wounds; cover inflamed surfaces, burns, sore nipples and chronic wounds. | F | Gum | Apply |  |  |
|  |  |  |  |  |  | Relieve irritation especially of mucus in the throat and inflammation, stomach or throat discomfort and controlling coughs; stopping heavy bleeding | F | Infusion | Oral |  |  |
|  |  |  |  |  |  | Antiseptic | D | Infusion | Wash |  |  |
|  | *Solanum betaceum* Cav.  (MNCY- 56) | DD | Chepkoroti | S | Se | Fever, cough, wounds, pain, liver troubles, tooth decay, reproductive problems, arterial hypertension and also poisoning. | D | Infusion | Oral |  | Solanaceae |
|  |  |  |  |  | R | Liver pain, sore throat, stomachache, headache, painful menstruation, pneumonia and rheumatism. | F | Decoction | Oral |  |  |
|  |  |  |  |  | L | Wrapped around the neck as a remedy for sore throat and tonsillitis. | F | Paste | Wrapped |  |  |
|  |  |  |  |  | Fr | Inflamed tonsils | F | Cooked-Pulp | Poultice |  |  |
|  |  |  |  |  | L | Coughs, colds and chest pain | D | Ash | Leak |  |  |
|  |  |  |  |  |  | Leave decoction is used for treatment of hypertension, diarrhea, respiratory tract infections, and various central nervous system (CNS) disorders such as epilepsy and depression. |  | Decoction | Oral |  |  |
|  |  |  |  |  | Fr | Anaemia. | F | Juice | Oral |  |  |
|  | *Solanecio mannii* (Hoof.f.) C.Jeffrey  (MNCY- 65) | LC | Chepkurbet | S/T | L | Enhance lactation in nursing mother (galactagogue) and wound dressing. | F | Pulp | Apply |  | Asteraceae |
|  |  |  |  |  |  | Cough tonic, allergies, cancer, pneumonia, cough, epilepsy, typhoid, localized oedema, heart troubles and relieve rheumatic pain | F | Decoction | Oral |  |  |
|  |  |  |  |  |  | Snake bite and piles from severe diarrhoea and constipation. | F | Extract | Apply |  |  |
|  |  |  |  |  | R | Hypertension, low sperm count and dysentery. | F | Decoction | Oral |  |  |
|  |  |  |  |  | L | Stop bleeding from cuts or injury & sore eyes. | F | Extract | Drops |  |  |
|  |  |  |  |  | L | Pulmonary defects, heart problem, cough and rheumatism. | F | Decoction | Oral |  |  |
|  | ***Coptosperma graveolens*****(S.Moore) Degreef** (MNCY- 163) | NE | Chepkurwet | S/T | B | Remedy for fever, malaria, cholera, diarrhoea, rheumatism, eye infections and tonsillitis. | F | Decoction | Oral |  | Rubiaceae |
|  |  |  |  |  | R | Rheumatism of the joints and sexual dysfunction | F | Decoction | Oral |  |  |
|  |  |  |  |  |  | Reproductive complaints like lack of orgasm and lubrication, pain during penetration, premature ejaculation, lack of sexual arousal and short lasting erections. | F | Infusion | Oral |  |  |
|  |  |  |  |  | L | Lung diseases and rheumatism root. | F | Decoction | Oral |  |  |
|  |  |  |  |  |  | Indigestion and influenza | D | Ash | Leak |  |  |
|  | *Seriphium kilimandscharicum*(O.Hoffm.) Koek.  (MNCY- 193) | NE | Chepkwesit | S | R | Coughs, malaria, for soreness of throat, swelling of the throat and jaws, nose bleeding; clear the bowels and as an anthelmintic, running nose, dysentery and sore throat; bleeding gums | F | Decoction | Oral |  | Asteraceae |
|  |  |  |  |  | Sb | Colds, coughs and chest pain. | F | Decoction/Infusion |  |  |  |
|  |  |  |  |  | FL/Sh | A hot poultice of the flowers and young leaves is used to relieve local pain and a good remedy bruises. |  | Poultice | Apply |  |  |
|  |  |  |  |  | St/L | Coughs. | D | Ash | Leak |  |  |
|  |  |  |  |  | L | Stomach problems. | F | Decoction | Oral |  |  |
|  | *Culcasia falcifolia* Engl.  (MNCY- 47) | LC | Chepnamobon/  Kipnamobon | H | L | Ulcers | F | Paste-dressing | Apply |  | Araceae |
|  |  |  |  |  |  | Ear-inflammation | F | Sap | Drops |  |  |
|  |  |  |  |  |  | Stomachaches | F | Decoction | Oral |  |  |
|  |  |  |  |  |  | Headaches and general body pain. | F | Pulp-Extract | Topical-Apply |  |  |
|  |  |  |  |  |  | Headache | D |  | Leak |  |  |
|  | *Eschenbachia subscaposa*(O.Hoffm.) G.L.Nesom  (MNCY- 127) | NE | Chepng’ombet | H | R | Roots are also used for influenza | F |  |  |  | Asteraceae |
|  |  |  |  |  | R | Throat swellings of tonsils | F | Powder | Topical |  |  |
|  |  |  |  |  | L | Snuffed to stimulate sneezing during the course of a cold | D | Powder | Snuff |  |  |
|  |  |  |  |  | R | Menstrual irregularities, general body health, haemorrhoids and painful menstruation. | F | Teas | Oral |  |  |
|  |  |  |  |  | WP | Coughs and colds | F | Steam | Inhalation |  |  |
|  |  |  |  |  | R | Malaria, wounds, diarrhoea, haemorrhoids, a tonic, headaches & dysentery. | F | Decoction | Oral |  |  |
|  |  |  |  |  | L | Malaria, induce vomiting, indigestion gastrointestinal problems-diarrhea and dysentery; promoting urination, internal hemorrhages, gonorrhea and bleeding haemorrhoids. | F | Infusion | Oral |  |  |
|  |  |  |  |  | WP | Diarrhoea, internal haemorrhages or applied externally to treat gonorrhoea and bleeding haemorrhoids | F | Infusion | Oral/Apply |  |  |
|  |  |  |  |  | L | Relief painful digestive disorders, colic, relieve pain and inflammation. | F | Decoctions | Oral |  |  |
|  |  |  |  |  |  | Scare off insects. | D | Smoke | Scent |  |  |
|  | *Pittosporum viridiflorum* Sims  (MNCY- 125) | LC | Chepng’ororyet | T | B | Chest complaints, malaria and other fevers; relieve pain, gastrointestinal complaints, STDs, kidney, circulatory and inflammatory disorders, cancer, TB, malaria and stomach ailments. | F | Decoction | Oral | Mutton-Soup | Pittosporaceae |
|  |  |  |  |  |  | Chest infection, abdominal pain, fever, chronic bronchitis rheumatism & induce lactation | F | Decoction | Oral |  |  |
|  |  |  |  |  | L | Enema. | F | Paste | Supposotory |  |  |
|  |  |  |  |  | B | Cold and coughs. | F | Roast-Powder | Leak |  |  |
|  |  |  |  |  | R/B | Aphrodisiacs | F | Infusion | Oral |  |  |
|  |  |  |  |  | B | Dysentery | D | Ash | Leak |  |  |
|  |  |  |  |  |  | Abdominal pains, induce vomiting & for respiratory diseases. | F | Infusion | Oral |  |  |
|  |  |  |  |  | R | Chest pains and dizziness | F | Infusion | Oral |  |  |
|  | *Unknown* (MNCY- 139) | NE | Chepng'orit | H | L | Wounds and swollen glands, treat septic wounds, snake bite sores, bruises, and backache and rheumatic joints | F | Poultice | Apply |  | Asteraceae |
|  |  |  |  |  | R | Throat infection | F | Extract | Oral |  |  |
|  |  |  |  |  | R | Cancer, coughs, colds, asthma and other respiratory problems. | F | Infusions | Oral |  |  |
|  |  |  |  |  | R | Headaches, fevers, indigestion, flatulence, hysteria, painful menstruation, delayed pregnancy & UTIs | F | Decoction | Oral |  |  |
|  | *Trimeria grandifolia* (Hochst.) Warb  (MNCY- 140) | LC | Chepnoet | S/T | G | Old wounds |  | Powder | Apply |  | Salicaceae |
|  |  |  |  |  | R | Swollen scrotum/testicles, measles, coughs, cancer and dysmenorrhea. |  | Decoction | Oral |  |  |
|  |  |  |  |  |  | Gonorrhea; joint pain caused by gonorrhea | F | Infusion | Oral |  |  |
|  | ** *Adenia cissampeloides*(Planch. ex Hook.) Harms  (MNCY- 202) | NE | Chepnyalildet | S/C |  | Anaemia, headache, peptic ulcers, anaemia, diarrhoea, infertility, respiratory infections like chest pains, colds, cough and tuberculosis; STIs (gonorrhoea and venereal diseases). | F | Decoction | Oral |  | Passifloraceae |
|  |  |  |  |  |  | Snake bites. |  | Decoction | Topica-Apply |  |  |
|  |  |  |  |  |  | Snake bites |  | Extract | Oral |  |  |
|  |  |  |  |  | R/S | Menstrual problems |  | Infusion | Oral |  |  |
|  | *Dichondra micrantha* Urb.  (MNCY- 75) | LC | Chepnyalilietab iitit | H | WP | Dysentery, jaundice, eye infections, oedema, malaria, abdominal distension, diarrhoea, urinary tract infections, swollen gums, bleeding, swelling and pain of eye. | F | Decoction | Oral |  | Convolvulaceae |
|  |  |  |  |  | WP | Sore throat and coughs | F | Extract | Oral | Honey |  |
|  |  |  |  |  | WP | Ear infections and earaches | F | Extract | Drops |  |  |
|  | *Vachellia elatior*(Brenan) Kyal. & Boatwr.  (MNCY- 72) | LC | Chepnyalilyet | T | B | Diarrhoea, mouth wash, treat a local collection of pus in any part of the body, infections of the brain and swellings; epilepsy, malaria, pneumonia, sterility, stomach trouble, syphilis, treat skin abscess and tonsillitis. | F | Decoction | Oral |  | Fabaceae |
|  |  |  |  |  | L | Constipation, influenza, coughing, promotes semen production and dysentery | F | Decoction | Oral |  |  |
|  |  |  |  |  | B/L | Cure gas and mouthwash to keep oral hygiene | D | Powder | Leak/Gargle | Honey |  |
|  |  |  |  |  | R | Stomachache, treat a child with fever; scabies, impotence, erectile dysfunction, fever, bowels, heart complaints, stomach diseases, skin eruptions in children; clear toxaemia of pregnancy | F | Decoction | Oral |  |  |
|  |  |  |  |  | L | Wounds and other skin problems, haemorrhoids, perspiring feet and eye problems. | F | Paste | Apply |  |  |
|  | *Tagetes minuta* L.  (MNCY- 85) | NE | Chepnyosoret | H | AP | Haemorrhoids, fungal infections, and athlete’s foot, skin infections, asthma, ulcers, stomachache, toothache, stop blood flow and to increase fertility | F | Decoction | Oral |  | Asteraceae |
|  |  |  |  |  | L | Killing maggots in wounds and ulcerations. | F | Extract | Apply |  |  |
|  |  |  |  |  | WP | Anthelmintic, control muscle spasms of the gut, heavy sweating; promote urination, induce vomiting, boost appetite, indigestion, belly pain, bloating and nausea; gastritis, indigestion and internal worms. | F | Decoction | Oral |  |  |
|  | *Micromeria biflora* (Buch. - Ham. ex D.Don) Benth. (MNCY- 172) | NE | Chepsagitiet | H | R | Colds, fever, gonorrhoea, stomach problems and rapid coughs followed by a high-pitched sound (likely to be whooping cough). | F | Decoction/Infusion | Oral |  | Lamiaceae |
|  |  |  |  |  | L | Headache, toothache, nausea, diarrhea, cramps and muscle pains | F | Infusion | Wash |  |  |
|  |  |  |  |  | L | Eye infections and diseases | F | Extract | Drops |  |  |
|  |  |  |  |  | R/L | Headache, vomiting and coughs | F | Decoction | Oral |  |  |
|  | *Micromeria* Benth. (MNCY- 183) | NE | Chepsagitiet | H | R | Asthma, diabetes, urinary diseases, heart disorders, headaches, wounds, skin infections and fever. | F | Decoction | Oral |  | Lamiaceae |
|  |  |  |  |  | WP | Nose bleeds |  | Scent | Inhalation |  |  |
|  |  |  |  |  | L | Flu, throat irritation |  | Tea | Oral |  |  |
|  |  |  |  |  | WP | Sinusitis, nervous system disorders, illnesses of respiratory system especially cough, headaches and fever | F | Juice | Oral/Inhalation |  |  |
|  |  |  |  |  | St/L | Asthma, colds and coughs. | D | Ash |  |  |  |
|  |  |  |  |  | WP | Pneumonia, stomach disorders, headache and wounds. | F | Extract | Oral |  |  |
|  |  |  |  |  | R | A paste of the root is placed between jaws to treat toothaches. | F | Paste | Placed on tooth |  |  |
|  |  |  |  |  | WP | A paste of the plant is used as a poultice to treat wounds. | F | Paste | Poultice |  |  |
|  | *Aspilia pluriseta* Schweinf. ex Engl.  (MNCY- 120) | NE | Chepsitait | H/S | L | Intestinal worms including hookworms, paralysis, oedema, jaundice, amenorrhea, urinary tract obstructions, skin disorders, menstrual disorders especially excessive or painful bleeding, renal stone, urinary disorders, blood detoxification, menstrual flow, promote blood circulation, promote urination, stop coughing blood or vomiting blood, nose bleeding | F | Decoction | Oral |  | Asteraceae |
|  |  |  |  |  | L/FL | To treat nettle stings and other skin inflammations. | F | Extract | Rub |  |  |
|  |  |  |  |  | F | Wounds; it helps to coagulate the blood of fresh cuts and wounds. | F | Juice | Apply |  |  |
|  |  |  |  |  | L | Skin diseases or put on cut wounds | F | Extract | Apply |  |  |
|  |  |  |  |  | WP | Cancer, treat wounds, cough, stomach illness, burn wounds, pimples, ears infections, eye infections, nose infections, kwashiorkor, fever, worms’ disorders, skin diseases, wounds, gonorrhea, abdominal pains, backache, respiratory problems and malaria. | F | Decoction | Oral |  |  |
|  |  |  |  |  | R | Breast feeding mothers to increase milk production. | F | Decoction | Oral |  |  |
|  | *Galinsoga parviflora*  Cav.  (MNCY- 58) | NE | Chepsitaki | H | L/FL | Treat nettle stings and other skin inflammations | F | Paste-Extract | Rub |  | Asteraceae |
|  |  |  |  |  | R | Eczema, stop bleeding, sores, common cold and flu; fever, diarrhoea and vomiting. | F | Decoction | Oral |  |  |
|  |  |  |  |  | WP | Wounds; it helps to coagulate the blood of fresh cuts, heal wounds, body injuries, | F | Juice | Apply |  |  |
|  |  |  |  |  | FL | Relieve pain of toothache, for wounds and eye diseases. | F | Extract | Apply |  |  |
|  |  |  |  |  | Sh | General wellness of wound healing, cold, flu and dermatological effects of a glowing skin |  | Cooked | Vegetable |  |  |
|  | *Hoslundia opposita* Vahl  (MNCY- 179) | NE | Cheptamtam | S | L/R | Fevers and mental illness. | F | Steam | Inhalation |  | Lamiaceae |
|  |  |  |  |  | L | Fever. The pounded leaves are used as a poultice for wounds. | F | Infusion | Bath |  |  |
|  |  |  |  |  | R | Aphrodisiac; as a remedy for colds and coughs and to relieve after-birth pains | F | Decoction | Oral |  |  |
|  |  |  |  |  | L | Skin diseases and as an antidote against snake-bites. | F | Extract | Apply |  |  |
|  | *Tylosema fassoglense*(Kotschy ex Schweinf.) Torre & Hillc.  (MNCY- 168) | NE | Cheptebesiet | S/T | Tu | Diarrhoea, anaemia, gastrointestinal problems, fever, venereal diseases, jaundice, impotence, hypertension, pneumonia, management of postpartum uterine healing and asthmatic attacks. | F | Decoction | Oral |  | Fabaceae |
|  |  |  |  |  | Tu | Impotence, constipation, diarrhea, flu, pneumonia, asthma, gastrointestinal infections, anemia, pneumonia, urinary tract infections and boost the immunity. | D | Powder-Infusion | Oral |  |  |
|  |  |  |  |  | L | Boils | F | Poultice | Apply |  |  |
|  |  |  |  |  | Se | Gastrointestinal problems, fever, anemia, pneumonia and fever | F | Infusion | Oral |  |  |
|  | *Momordica foetida* Schumach.  (MNCY- 48) | NE | Cheptenderet | H/L | L | Earache | F | Paste | Stuck on the ear |  | Cucurbitaceae |
|  |  |  |  |  | L | Ringworm | F | Juice | Rub |  |  |
|  |  |  |  |  | L | Oral thrush. | F | Ash | Apply |  |  |
|  |  |  |  |  | L/R | Abortion | F | Infusion | Oral |  |  |
|  |  |  |  |  | R | Intestinal worm infection. | F | Infusion | Oral |  |  |
|  |  |  |  |  | L | Itchy anus. | F | Extract | Oral |  |  |
|  |  |  |  |  | R | Induce vomiting | F | Decoction | oral |  |  |
|  | *Thunbergia alata* Bojer ex Sims  (MNCY- 215) | NE | Cheptereret | H/L | L | Skin cancer, colds, flu, asthma, TB, bronchitis, rheumatism, arthritis, liver problems, hemorrhoids, bladder, uterus and “women's” complaints; diarrhea and dysentery, stomach ailments, heartburn, peptic ulcers, backache, diabetes, varicose veins, inflammation, inflammatory diseases such as fever, cough and diarrhea | F | Extract | Oral |  | Acanthaceae |
|  |  |  |  |  |  | Headache | F | Extract | Apply |  |  |
|  |  |  |  |  |  | A paste of the leaves is topically on sores | F | Paste | Topical |  |  |
|  |  |  |  |  | L/S | Fever and malaria, diarrhea cough, flu, and backache | F | Infusion | Oral |  |  |
|  |  |  |  |  | L | Internal haemorrhages or for early rectal cancer | F | Sap | Oral |  |  |
|  |  |  |  |  |  | Boils | F | Poultice | Apply |  |  |
|  |  |  |  |  | L | Eyes inflammation (probably conjunctivitis) | F | Sap | Drops |  |  |
|  | *Maytenus undata* (Thunb.) Blakelock  (MNCY- 196) | LC | Cheptuiinik | S/ST | R | Syphilis, diseases affecting the urethra, stomach ailments, malaria, fevers, ulcers, stomach upsets, leukemia, diarrhoea, haemorrhoids, coughs, cold, venereal diseases, measles, dysmenorrhea, promote general body strength, syphilis, hernia, pneumonia, constipation, arthritis, nausea, belly pain, ulcers and inflammation | F | Decoction | Oral |  | Celastraceae |
|  |  |  |  |  | B | As a tonic, malaria, tumours, dysentery, syphilis, urethra infections, epilepsy, general body pain, sore throat, earache, infertility and gastrointestinal disorders. | F | Decoction | Oral |  |  |
|  | *Diospyros abyssinica* (Hiern) F.White  (MNCY- 6) | LC | Cheptuiyet | T | R | Relieve a dry tongue during high fever | F | Extract-Juice | Oral |  | Ebenaceae |
|  |  |  |  |  | R | Chest complaints. | D | Powder-Smoked | Inhalation |  |  |
|  |  |  |  |  | R | Reproductive problems, diarrhoea, gonorrhoea & internal hemorrhage | F | Decoction | Oral |  |  |
|  |  |  |  |  | L | Bedwetting in children, woman’s infertility issues, tuberculosis, malaria, diarrhea, cancer, meningitis, inflammatory diseases, urinary tract infections, wounds, diabetes, skin & soft tissue injuries. | F | Decoction | Oral |  |  |
|  |  |  |  |  | L | Infant complications | D | Ash | Leak |  |  |
|  | *Dolichopentas longiflora*(Oliv.) Kårehed & B.Bremer  (MNCY- 211) | NE | Cheroriet | T | R | Cough, initiate menstruation, headache, fever and rheumatic pain. | F | Infusion | Oral |  | Rubiaceae |
|  |  |  |  |  | R | Tapeworm infestation, cholera, gonorrhoea, fever, syphilis and dysentery. | F | Decoction | Oral |  |  |
|  |  |  |  |  | R | Malaria and induce vomiting | F | Decoction | Oral | Milk |  |
|  |  |  |  |  | R | Itchy rashes and pimples | F | Extract | Apply |  |  |
|  |  |  |  |  | L | Ringworm and skin rashes | F | Extract | Apply |  |  |
|  |  |  |  |  | R | Pimples | F | Paste | Apply | Ghee |  |
|  |  |  |  |  | L | Scabies | F | Infusion | Bath |  |  |
|  |  |  |  |  | L | Snake bite | F | Chew-Poultice | Mounted |  |  |
|  |  |  |  |  | R | Snake bite | F | Infusion | Oral |  |  |
|  | *Typha latifolia*L.  (MNCY- 73) | NE | Cherungut | SS/S | R | Cough remedy, wounds, gonorrhea, stomach pains, sores, bladder inflammation, male aphrodisiac, contract the uterus- induce abortion and/or reduce post-partum bleeding, used post-partum to cleanse the uterus of any blood clots and heal vaginal lacerations after childbirth | F | Decoction | Oral |  | Typhaceae |
|  |  |  |  |  | L | Fever in children. | F | Infusion | Wash |  |  |
|  |  |  |  |  | L | Induce vomiting treat malaria, coughs, chest pain and internal worm infection. | F | Decoction | Oral |  |  |
|  |  |  |  |  | R/L | Fever and colds, coughs, fever, convulsions, mental disturbances, abdominal pains, snake bites, swellings, ulcers, chronic and deep wounds, dermatitis, sore throats, venereal infections | F | Infusion | Oral |  |  |
|  |  |  |  |  | R/L | Insect repellents | F | Infusion | Scent |  |  |
|  |  |  |  |  | L | Fresh wound to accelerate healing | F | Paste | Spray |  |  |
|  | *Grewia similis* K. Schum  (MNCY- 156) | NE | Cheseber | S | R | Chronic skin lesions | F | Poultice | Apply |  | Malvaceae |
|  |  |  |  |  | R | Anemia, chest complaints, cold, diarrhea, snakebites, mental illness, hernia and female infertility. | F | Infusion | Oral |  |  |
|  |  |  |  |  | B | Skin itches and boils. | F | Paste | Apply |  |  |
|  |  |  |  |  | R | Delayed afterbirth; relieve anxiety, dental hygiene, malaria, general body strength, wound care, cough & cold | F | Decoction | Oral |  |  |
|  |  |  |  |  | R | Controlling bleeding and bronchitis, skin disease headache, eye complaints, sores and cholera | F | Extract | Oral |  |  |
|  |  |  |  |  | B | Given to pregnant women for smooth delivery, constipation, expectorant during cough and cold, chest pain, tuberculosis, coughing with blood & rheumatism | F | Extract | Oral |  |  |
|  |  |  |  |  | Fr | Heart complaints, treat dysentery & sore throat | F | Extract | Oral |  |  |
|  |  |  |  |  | Se | Infertility | D | Powder-Infusion | Oral |  |  |
|  |  |  |  |  | B | Anthelminthic, laxative, to treat boils, sores, intestinal inflammation & syphilis | F | Decoction | Oral |  |  |
|  |  |  |  |  | St | Anthelmintic and as a tooth brush. | F | Extract | Oral |  |  |
|  | *Asparagus racemosus* Willd.  (MNCY- 105) | NE | Chesibaiyat | C | R | Stomach complaints and upsets, constipation, aphrodisiac, nervous system disorders, promote appetite, antiseptic, restore general health, diarrhoea, dysentery, tumours, inflammations, cough, bronchitis, hyperacidity, stomach ulcers, oedema, pain, anxiety, cancer, diarrhea, bronchitis, tuberculosis & diabetes. | F | Decoction | Oral |  | Asparagaceae |
|  |  |  |  |  | R | Women issues like premenstrual syndrome (PMS), uterine bleeding, to increase sexual desire and to stimulate breast milk production. | F | Infusion | Oral |  |  |
|  |  |  |  |  | L | Stiffness in the joints and arthritis | F | Paste | Apply |  |  |
|  | *Erythrococca bongensis* Pax  (MNCY- 49) | LC | Chesichet | S/ST | L | Diarrhoea, eye infection (cataracts), cough, belly pain, flatulence, colic, nasal swellings & stomach pain. | F | Decoction | Oral |  | Euphorbiaceae |
|  |  |  |  |  | R | Circulatory issues, chest pain, rheumatism, sexual weakness or impotence. | F | Decoction | Oral |  |  |
|  |  |  |  |  | L | Eye infection. | F | Infusion | Drops |  |  |
|  |  |  |  |  | L | Wounds, measles, burns & malaria | D | Ash | Leak |  |  |
|  | *Achyranthes aspera* L. (MNCY- 82) | NE | Chesirimiot/  Chesirimto | H | R | Bowel complaints and venereal diseases. | F | Infusion | Oral |  | Amaranthaceae |
|  |  |  |  |  | WP | Toothaches | F | Decoction | Gargle |  |  |
|  |  |  |  |  | WP | Pneumonia | F | Decoction | Oral |  |  |
|  |  |  |  |  | R | Constipation in children, cancer, indigestion, cure stitch, softens stool and lowers the risk of constipation, fever, wound healing, tooth ache, arthritis, gynecological disorders, urinary disorders, stomach pain & rheumatic ailments | F | Decoction | Oral |  |  |
|  |  |  |  |  | L | Boils | D | Ash | Apply |  |  |
|  |  |  |  |  | L | Ankle sprains. | D | Powder | Apply |  |  |
|  |  |  |  |  | L/FL | Insect, dog and snake bites. | F | Extract | Apply | Honey |  |
|  |  |  |  |  | R | Stop bleeding on cuts | F | Extract | Apply |  |  |
|  |  |  |  |  | WP | Boils, asthma, in facilitating delivery, bleeding, bronchitis, general body weakness, earache, headache, renal complications, pneumonia, oedema, cold, colic, cough, dog bite, snake bite, scorpion bite, prolonged menstrual flow, abnormal menses, and dysmenorrhea, dysentery & skin diseases; colic relief in infants, treatment of pain, inflammatory diseases, burning sensation in chest, body weakness, fever, sores, boils, smallpox, muscle wastage, lactation complaints, increase taste and appetite. | F | Decoction | Oral |  |  |
|  |  |  |  |  | R | Paralysis, epilepsy, convulsions, diarrhoea, spasm hiccups, cancer, blood purification & detoxification | F | Decoction | Oral |  |  |
|  |  |  |  |  | B | Fever, tumours tapeworms & dysentery | F | Decoction | Oral |  |  |
|  | *Hymenodictyon floribundum*(Hochst. & Steud.) B.L.Rob.  (MNCY- 57) | LC | Chilgatwet | S/ST | R | Paralysis, epilepsy, convulsions, diarrhoea, spasm hiccups, cancer, blood purification, detoxification; colic relief in infants, pain, inflammatory diseases, burning sensation in chest, body weakness, fever, sores, boils, smallpox, muscle wastage, lactation complaints, increase taste & appetite | F | Decoction | Oral |  | Rubiaceae |
|  |  |  |  |  | B | Fever, tumours, tapeworms & dysentery | F | Decoction | Oral |  |  |
|  | *Ficus exasperata*Vahl  (MNCY- 133) | NE | Chomisiat | S/ST | R/Fr | Bone defects, tissue, wound healing, iron deficiency anaemia, heart, toothache, blood and liver disorders, poor feeding, indigestion, thirst, toxemia, stomach complains, hiccups, cough, asthma, promoting sperm production, fevers, diarrhea, throat infections, TB & sexual weaknesses | F | Decoction | Oral |  | Moraceae |
|  |  |  |  |  | B | Stomachs upset, sores, intestinal infections, cough, fever, diarrhoea, dysentery, jaundice & rheumatism. | F | Decoction | Oral |  |  |
|  |  |  |  |  | Rb | Rheumatism and urinary tract problems | F | Decoction | Oral |  |  |
|  |  |  |  |  | FL | Earache | F | Infusion | Drops |  |  |
|  |  |  |  |  | L | Wounds, cuts, relieve irritation & painful rashes | F | Extract | Apply |  |  |
|  |  |  |  |  | FL | Chest and abdominal complaints (productive cough, diarrhea and rheumatic problems) | F | Infusion | Oral |  |  |
|  | *Nuxia congesta*R.Br.  (MNCY- 190) | LC | Chorwet | S/ST | L | Fever, influenza, convulsions, rheumatism, scabies, tonsillitis, coughs, colds, back aches, indigestion stomachaches, joint pains, malaria & anthelmintic | F | Decoction | Oral |  | Stilbaceae |
|  |  |  |  |  | B/L | Indigestion & toothache | F | Extract/Decoction | Oral |  |  |
|  |  |  |  |  | B | Relief for painful dysmenorrhea, reduce bleeding, indigestion, menstrual problems, malaria, headache, enlarged spleen, cough, tonsillitis, dermatological problems, management of rheumatism, joint swellings and as an expectorant. | F | Decoction | Oral |  |  |
|  |  |  |  |  | R | Common colds, flu and abdominal pain. | F | Decoction | Oral |  |  |
|  |  |  |  |  | Tw | Ash of leafy twigs in water is used to treat diarrhea | D | Ash | Leak |  |  |
|  | *Lippia javanica* Spreng*.*  (MNCY- 177) | NE | Chorwet | S/ST | L | Respiratory disorders, cholera, diarrhoea, dysentery & digestive complaints | F | Infusion | Oral |  | Verbenaceae |
|  |  |  |  |  | L/St | Asthma and chronic coughs | D | Smoke | Inhalation |  |  |
|  |  |  |  |  | R | Fever, malaria, influenza, measles, cholera, diarrhoea and dysentery. | F | Decoction | Oral |  |  |
|  |  |  |  |  | L | Lung infections, coughs, aching muscles, malaria, colds, fever, wounds, diarrhoea, chest pains, bronchitis, asthma, other respiratory problems, gastrointestinal diseases, injuries, pain & skin infections | F | Decoction | Oral |  |  |
|  |  |  |  |  | L | Leaves are used as mosquito and other insect repellant | F | Extract | Scent |  |  |
|  | *Leonotis ocymifolia*var. *raineriana*(Vis.) Iwarsson  (MNCY- 243) | NE | Chuchuniat | S | WP | Alleviate pain during labour, kidney issues, stomach pains, hepatic disorders, gastrointestinal problems & abdominal distention | F | Decoction | Oral |  | Lamiaceae |
|  |  |  |  |  | R | Wounds, sores, headaches, flu, dysentery, chest infections, epilepsy, malaria, constipation, indigestion, intestinal worms, insect bites, snake bites, menstrual delays, hypertension & intestinal worms | F | Decoction | Oral |  |  |
|  |  |  |  |  | Sh/Bd | Eye infections. | F | Extract | Drops |  |  |
|  |  |  |  |  | R | Dysentery and intestinal disorders | F | Infusion | Oral |  |  |
|  |  |  |  |  | L | Cramp in the stomach, cold, cough, fever, headache & asthma | F | Extract | Oral |  |  |
|  | *Leonotis nepetifolia* (L.) R.Br.  (MNCY- 198) | NE | Chuchuniat | S | L | Influenza, fever, coughs, uterine complaints after birth, malaria bronchial asthma, fever, diarrhea, cough, relieve pain, burns, breast swelling, ringworm, malaria, epilepsy and rheumatic pain | D | Infusion | Oral |  | Lamiaceae |
|  |  |  |  |  | L | Stomach troubles | F | Extract | Oral |  |  |
|  |  |  |  |  | R | Stomach disorders | F | Infusion | Oral |  |  |
|  |  |  |  |  | L | Chronic wounds including cancer related wounds "Seriat" | D | Powder | Apply |  |  |
|  |  |  |  |  | L/FL | Burns, eczema, insect bites, skin ailments, joint pains & back pain | F | Paste | Apply/  Massage |  |  |
|  |  |  |  |  | WP | Breast pain after birth, stroke, general body pain, body swellings, joint pain, liver problems, stomach infections & cough | D | Ash | Leak | Ghee |  |
|  |  |  |  |  | R | Root decoction is used on cancers | F | Decoction | Oral |  |  |
|  |  |  |  |  | WP | Stomachache, jaundice, regulating the menstrual cycle, diarrhea, asthma, back pain, relieving joint pain & dysentery | F | Decoction | Oral |  |  |
|  |  |  |  |  | R | Stomachache | F | Decoction | Oral |  |  |
|  | *Olea europaea*subsp. *cuspidata*(Wall. & G.Don) Cif.  (MNCY- 214) | NE | Emdit | T | B | Tapeworm, sore throat, kidney problems and backache | F | Infusion | Oral |  | Oleaceae |
|  |  |  |  |  | B | Anthelmintic-tapeworm infestation or used in steam bath and some drunk for treatment of itchy rash, retained placenta, asthma, rheumatism and pain in the muscles. | F | Decoction | Oral/Bath |  |  |
|  |  |  |  |  | L | Treat eye infections | F | Infusions | Drops |  |  |
|  |  |  |  |  |  | Sore throat | F | Infusions | Gargle |  |  |
|  |  |  |  |  |  | Colic, UTIs & blood pressure. | F | Infusions | Oral |  |  |
|  |  |  |  |  |  | Stop bleeding from cuts | D | Powder | Apply |  |  |
|  |  |  |  |  |  | Headache & nose bleeding | D | Powder | Snuff |  |  |
|  |  |  |  |  |  | Hepatic diseases, eye infections, sore throat, kidney problems, UTIs, mouthwash, backaches & headaches | F | Decoction | Oral |  |  |
|  |  |  |  |  |  | Fever, malaria, diabetes, inflammatory & hypertension | F | Decoction | Oral |  |  |
|  | *Zanthoxylum chalybaeum*Engl.  (MNCY- 129) | LC | Igomiet | T | R | Managing bone marrow diseases (anaemia; sickle cell), fever, malaria, worms, yellow fever, body pains, excessively swollen feet, toothache, diabetes, sexual impotence, gonorrhea, malaria, dysmenorrhea and abdominal pain | F | Decoction | Oral |  | Rutaceae |
|  |  |  |  |  | R | Asthma & abdominal pain | D | Powder | Oral | Porridge/  Tea |  |
|  |  |  |  |  | Sb | The stem bark is also useful when chewed to treat toothache. | F | Extract | Apply |  |  |
|  |  |  |  |  | L | Convulsions, oedema, swollen legs, body pains & psychiatric problems | F | Infusion | Oral |  |  |
|  |  |  |  |  |  | Snakebites | F | Extract | Apply |  |  |
|  |  |  |  |  | Sb | Malaria, fevers and headache, sickle cell disease, respiratory tract ailments including colds and TB, skin diseases including ulcers & itchy rash | F | Decoction | Oral |  |  |
|  |  |  |  |  | Fr | Breath fresheners, fever, sore throat, severe colds, chest pain & pneumonia | F | Extract | Oral |  |  |
|  |  |  |  |  | Fr/L | Tonic to children | F | Infusion | Oral |  |  |
|  |  |  |  |  | Se | Sore throat and tonsillitis, tumours, measles, intestinal problems including abdominal pain, diarrhea, intestinal worms, amoebas, colic, general body pain & vomiting | D | Infusion | Oral | Tea/Milk |  |
|  | *Ricinus communis* L.  (MNCY- 97) | NE | Imaniat | S/ST | Sh | Wounds healing | F | Paste | Tied |  | Euphorbiaceae |
|  |  |  |  |  | R | Stimulate appetite, abdominal trouble, arthritis or for the treatment of venereal diseases. | F | Decoction | Oral |  |  |
|  |  |  |  |  | Se | Opening the bowels, as a cure for afterbirth bleeding, & abdominal trouble |  | Extract |  |  |  |
|  |  |  |  |  | Se | Aching ear | D | Oil | Drops |  |  |
|  |  |  |  |  | St/L | Ulcers, stomachache & diarrhoea | F | Juice | Oral |  |  |
|  | *Coleus barbatus*(Andrews) Benth. ex G.Don  (MNCY- 225) | NE | Irokwet | H/S | L | Measles, scabies and skin diseases. | F | Extract | Bath |  | Lamiaceae |
|  |  |  |  |  | R | Gastrointestinal infections, ulcers, intestinal pain, sore throat, oral infections, tonsillitis, mouth ulcers, boils, muscle sprains, blood pressure, heart diseases, colic, burning sensation, nervous system complaints, asthma, respiratory complaints, bowel cleansing, pain during urination, tooth and gum disorders and cancer especially colon cancer. | F | Decoction | Oral |  |  |
|  |  |  |  |  | L | Ringworms, eczema & cleaning wounds as an antiseptic | F | Extract | Apply/Wash |  |  |
|  |  |  |  |  |  | Earache | F | Extract | Drops |  |  |
|  |  |  |  |  |  | Detoxification & headache | F | Steam | Inhalation |  |  |
|  |  |  |  |  |  | Stomachache, clean the bowel, intestinal disturbance, liver fatigue, certain nervous system disorders like pain, heart disease, convulsions, respiratory disorders, pain, painful urination, coughs & colds | F | Juice | Oral |  |  |
|  |  |  |  |  |  | Typhoid, expectorant, stuffy noses, asthma, chronic coughs, rheumatism, colic, dysentery, flatulence, malaria, relieves pain, liver disturbances, epilepsy & bronchitis | F | Infusion | Oral |  |  |
|  | *Cleome gynandra*L (MNCY- 209) | NE | Isakiat | H/SS | R | Chest pain, the leaves to treat diarrhoea migraine headache, epilepsy, stomach ache, ear pain, sepsis, vomiting, promoting contractions of labour during pregnancy, help the process childbirth in pregnant women, stomachache, indigestion, constipation, eye infection, diarrhoea, worm infection, relieve chest pains, arthritis, inflammation, tissue pain & promote lactation | F | Infusion | Oral |  | Cleomaceae |
|  |  |  |  |  | L/FL | Scurvy, insect bites, snake bite & rheumatism. | F | Extract | Topical-Apply |  |  |
|  |  |  |  |  | R | Chest pain, dysentery, gonorrhoea, malaria & arthritis. | F | Infusion | Oral |  |  |
|  |  |  |  |  |  | Fever and cancers | F | Decoction | Oral |  |  |
|  |  |  |  |  | L | Boils, wounds, sepsis and localized pain | F | Poultice | Apply |  |  |
|  |  |  |  |  | L/St | Reduce dizzy episodes in pregnant women. | F | Decoction | Oral |  |  |
|  |  |  |  |  | L | Earache; Drops in to nostrils and eyes to treat epileptic seizures, pain, headaches. |  | Sap | Drops |  |  |
|  | Unknown  (MNCY- 53) | NE | Isiriat | H | R | Chest pain, dewormer and measles. | F | Decoction | Oral |  | Asteraceae |
|  |  |  |  |  | St/L | Pneumonia | F | Decoction | Oral |  |  |
|  | *Olinia rochetiana* A.Juss.  (MNCY- 143) | NE | Kabekeryet | ST/T | B | Tapeworms, rheumatism, bronchitis & indigestion | F | Decoction | Oral |  | Penaeaceae |
|  |  |  |  |  | Sh | Stimulant |  | Chew-Extract | Oral |  |  |
|  |  |  |  |  | L | Throat infection and throat cancer | F | Extract | Oral |  |  |
|  |  |  |  |  | R | Coughs or | F | Decoction | Oral | Milk |  |
|  |  |  |  |  |  | Fever by inducing vomiting of bile | F | Decoction | Oral |  |  |
|  |  |  |  |  | B | Coughs | F | Extract | Oral |  |  |
|  | ***Schrebera alata*(Hochst.) Welw.  (MNCY- 44) | LC | Kakaawet | S/ST | B | Induce vomiting, relax muscles during delivery of pregnant women, STDs, eye infections, skin-related problems, wounds, gastrointestinal diseases, measles, snake bites, skin diseases, respiratory problems, laxative, cancer, inflammation, tonsils, postpartum bleeding, toothache & to restore health | F | Decoction | Oral |  | Oleaceae |
|  |  |  |  |  | L | Tonsillitis, inflammations of the throat, sore throat, toothache, sore throat & headache | F | Extract | Oral |  |  |
|  |  |  |  |  |  | Decoction from the leaves is taken treatment of headache, colds, cough, and fever; cleanse the stomach, tonsils, bleeding, wounds & postpartum bleeding | F | Decoction | Oral |  |  |
|  |  |  |  |  | B | Powerful pain killer; removing sensation agent/anesthesia | F | Infusion | Oral |  |  |
|  |  |  |  |  |  | Toothache | F | Chew-Extract | Apply-On tooth |  |  |
|  | *Erythrina abyssinica*Lam. ex DC.  (MNCY- 68) | LC | Kakaruet | T | B | Eyes inflammation of the eyelids (possibly trachoma). | F | Extract | Drops |  | Fabaceae |
|  |  |  |  |  | B | Cancer | F | Decoction | Oral |  |  |
|  |  |  |  |  | B | Gonorrhea and STDs. | F | Decoction | Oral | Mutton/  Soup |  |
|  |  |  |  |  | B | Ash from the bark is also used on oral thrush | D | Ash | Apply |  |  |
|  |  |  |  |  | B | Burns and general body swellings | D | Roasted-Powder | Apply |  |  |
|  |  |  |  |  | St | Eyes inflammation of the lids, anthelmintic & relieve a special abdominal pain | F | Extract | Oral |  |  |
|  |  |  |  |  | R | Malaria, syphilis, urinary problems & snake bites |  | Decoction | Oral |  |  |
|  | *Dovyalis abyssinica* (A.Rich.) Warb.  (MNCY- 131) | LC | Kapchobiniot | ST | L | Indigestion. | F | Infusion | Oral |  | Salicaceae |
|  |  |  |  |  | R | Gonorrhea, brucellosis, teeth problems, typhoid, diarrhea, brucellosis, tapeworm, sore throat, headache, stomachache, amoebiasis, fever, colic pain in infants, malaria, cancer, circulatory issues, detoxification/ blood purifier | F | Decoction | Oral |  |  |
|  |  |  |  |  |  | Headache | F | Steam | Inhalation |  |  |
|  |  |  |  |  | Fr | Abdominal pain | F | Juice | Oral |  |  |
|  |  |  |  |  | L | Abdominal pain | F | Infusion | Oral |  |  |
|  |  |  |  |  | SB | Oral hygiene | F | Decoction | Gargle |  |  |
|  |  |  |  |  |  | Wound healing, cancer, pneumonia, arthritis, tonsils, infertility, malaria, oedema, typhoid, gonorrhea, stomachache, fever and cancer | F | Decoction | Oral |  |  |
|  |  |  |  |  | R/St | Colds and coughs, gonorrhoea, stomachache & fever | F | Decoction | Oral | Soup |  |
|  | *Entada africana* Guill. & Perr.  (MNCY- 147) | LC | Kapkutuet | T | SB/R | Chronic wounds especially cancer associated, female infertility, respiratory problems, diabetes, hypertension, diarrhoea, bronchitis, colds, coughs, gonorrhoea, syphilis, dysentery, eye infections, stomachache, fever, liver complaints, inflammation & TB | F | Decoction | Oral |  | Fabaceae |
|  |  |  |  |  | R | Rheumatic pains, cancer, female infertility, malaria, rheumatism, arthritis, circulatory troubles, stomachache, induce vomiting, diarrhea, dysentery, worm infections, STDs, stroke, epilepsy, convulsions & muscles spasms | F | Decoction | Oral |  |  |
|  |  |  |  |  | L | Sores, skin infections, induce vomiting, stomachaches, dysentery, fever, malaria & as a tonic | F | Decoction | Oral |  |  |
|  |  |  |  |  | L/St | Restore general health & stomachache | F | Infusion | Oral |  |  |
|  |  |  |  |  | L | Wounds dressing and boils. |  | Paste-Dressing | Apply |  |  |
|  | *Tiliacora triandra* (Colebr.) Diels (MNCY- 146) | NE | Kaporoon | L | R | Dewormer, STDs | F | Decoction | Oral |  | Menispermaceae |
|  | *Cordia africana* Lam. (MNCY- 116) | LC | Kaptilalwet | S/ST | R | Circulatory issues, wounds, paralysis, rheumatism, coughs, fevers, liver ailments, flatulence, jaundice, fevers & convulsions | F | Decoction | Oral |  | Boraginaceae |
|  |  |  |  |  | L | Stomach problems in children, appetizer, fever; promote digestion, tonic, dysentery, colic & diarrhea | F | Decoction | Oral |  |  |
|  |  |  |  |  |  | Sores, boils to bring a head, expel the pus | F | Paste | Poultice |  |  |
|  |  |  |  |  |  | Burns & wounds | D | Ash | Butter |  |  |
|  |  |  |  |  |  | General body ailment, diarrhoea, & tonsillitis | F | Juice | Oral |  |  |
|  |  |  |  |  | B | Broken bone | F | Pieces | Tie |  |  |
|  |  |  |  |  |  | Venereal diseases | F | Decoction | Oral |  |  |
|  |  |  |  |  | R | TB, cough, amoebiasis & burns and wounds asthma | F | Decoction | Oral |  |  |
|  | *Vachellia abyssinica* (Hochst. ex Benth.) Kyal. & Boatwr.  (MNCY- 128) | NE | Katabeleliat | T | L/Bd | Abscess | F | Paste | Poultice-Tie | Ghee | Fabaceae |
|  |  |  |  |  | L | Fever | F | Decoction | Oral |  |  |
|  |  |  |  |  |  | Colds, chest pain & coughs | D | Ash | Leak |  |  |
|  |  |  |  |  | R | Impotence, gonorrhea treatment, UTIs & abdominal pains | F | Decoction | Oral |  |  |
|  |  |  |  |  | L | Chest pains or pneumonia | F | Decoction | Oral |  |  |
|  |  |  |  |  | R | Indigestion or stomach trouble, gonorrhea & chest diseases | F | Decoction | Oral |  |  |
|  |  |  |  |  | B/R | Aphrodisiac, on treatment of colds and pneumonia | F | Decoction | Oral |  |  |
|  | *Carduus schimperi*Sch.Bip.  (MNCY- 62) | NE | Katet/Tekweyot | SS | WP | Liver diseases, stomach complains, eye infections, malaria, menstrual pain, diarrhoea, expectorant, influenza, relieves fever, increases urine output, boils, wounds, aids digestion & stomach upsets | F | Decoction | Oral |  | Asteraceae |
|  |  |  |  |  |  | Promote appetite; enhance bile secretion, jaundice, decrease flatulence & aid digestion | F | Powder-Infusion | Oral |  |  |
|  |  |  |  |  |  | Colds, coughs, liver problems, expectorant, digestive complaints and respiratory diseases | F | Ash | Leak |  |  |
|  | *Entada abyssinica* Steudel ex A.Rich.  (MNCY- 186) | LC | Katutwet |  | B | Induce abortion. | F | Infusion | Oral |  | Fabaceae |
|  |  |  |  |  | R | Gonorrhoea and other STDs, sleeping sickness, coughs, rheumatic fever, abdominal pain & diarrhea | F | Infusion | Oral |  |  |
|  |  |  |  |  | Rb | Relieve frequent sneezing | D | Powder | Snuff |  |  |
|  |  |  |  |  | SB | Coughs, colds, syphilis, chronic bronchial engorgement, rheumatic pains, abdominal pain, peptic ulcers, mouth wounds and malaria; peptic ulcers, treat coughs, rheumatism, bronchitis, abdominal pains, diarrhoea, fever, prevent miscarriages, bronchitis, coughs, diarrhoea, fever & to alleviate arthritic pains | F | Decoction | Oral |  |  |
|  |  |  |  |  | B | Induces abortion therefore used for uterine issues; colds, stomach pains, bronchial problems, antidote, fibroids, blood purifier, libido, venereal diseases & amoeba | F | Decoction | Oral |  |  |
|  |  |  |  |  | Rb | Swollen body parts | D | Powder | Massage | Ghee |  |
|  |  |  |  |  |  | Cataracts and diseases of the eye | D | Powder-Infusion | Drops |  |  |
|  |  |  |  |  | L | Relieve fever, relieve back pain, reduce morning sickness in pregnant women, remove skin rashes, malaria, promote good health & wound healing | F | Decoction | Oral |  |  |
|  |  |  |  |  | R | Syphilis, malaria, wounds, hepatitis, tonsillitis, scabies, liver problems especially jaundice. However caution should be taken because the recommended dose portions must be in very small quantities | F | Infusion | Oral |  |  |
|  |  |  |  |  |  | Insect repellent | F | Decoction | Scent |  |  |
|  | ** *Acokanthera schimperi*(A.DC.) Schweinf.  (MNCY- 124) | LC | Keliot | S/ST |  | Syphilis, malaria, wounds, hepatitis, tonsillitis, scabies, insect repellent & liver problems-jaundice | F | Infusion | Oral |  | Apocynaceae |
|  | *Combretum pisoniiflorum*(Klotzsch) Engl.  (MNCY- 169) | LC | Kemelyet | S/ST | R | Worm infestations, stomach pains, snake bite, chronic wounds, fever, dysentery, general body swellings and is associated with causing abortion | F | Decoction | Oral |  | Combretaceae |
|  |  |  |  |  | R/L | Snake bite | F | Decoction | Oral |  |  |
|  |  |  |  |  | L | Chest complaints & anthelmintic | F | Extract/Juice | Oral |  |  |
|  |  |  |  |  |  | Wounds | F | Paste | Apply |  |  |
|  |  |  |  |  |  | Chest complaints | F | Steam | Bath/  Inhalation |  |  |
|  | *Sida schimperiana*Hochst. ex A.Rich.  (MNCY- 81) | NE | Kerundut | S | WP | Relieve skin itching, irritation from chickenpox & measles spots | F | Decoction | Bath/Wash |  | Malvaceae |
|  |  |  |  |  | L | Nervous and urinary diseases, disorders of the blood and bile, asthma & heart health | F | Infusion | Oral |  |  |
|  |  |  |  |  | Rb | Frequent urination & vaginal discharge | F | Powder | Oral | Milk/  Sugar |  |
|  |  |  |  |  | L | Boils in order to promote pus release, | F | Poultice | Apply |  |  |
|  |  |  |  |  |  | Involuntary discharge of semen, rheumatism and gonorrhea. | L | Juice | Oral |  |  |
|  |  |  |  |  | R | Fevers | F | Decoction | Oral |  |  |
|  |  |  |  |  | L | Relief pain, inflammation, irritation, and diuretic, to sooth the skin, fever, therapeutic on nerves, stimulant & tonic | F | Decoction | Oral |  |  |
|  |  |  |  |  | L | Bleeding haemorrhoids |  | Cooked | Vegetables |  |  |
|  | *Vangueria apiculata* K.Schum.  (MNCY- 194) | LC | Kesekesiet | S/ST | R | Gastrointestinal problems, diabetes, malaria, pain, parasitic worms, hiccups, toothache, antidote for poisoning, eye infection, induce labour, infertility & measles. | F | Decoction | Oral |  | Rubiaceae |
|  |  |  |  |  | L | Stomachache, malaria, pneumonia & cough | F | Juice | Oral |  |  |
|  |  |  |  |  | B | Blood stain in stool, aphrodisiac, cold, headache, menstrual problems, parasitic worms, chest complaints, infertility, fever, candidiasis & abdominal pains. | F | Decoction | Oral |  |  |
|  |  |  |  |  | R | Abdominal pains, indigestion & gastrointestinal complaints | F | Infusions | Oral |  |  |
|  | *Piper umbellatum* L.  (MNCY- 118) | NE | Ketekaa | H | L | Abscesses, wounds and as a poultice on swellings, boils & burns | F | Paste | Topical |  | Piperaceae |
|  |  |  |  |  |  | Stimulate menstrual flow, prevent abortion, reduce itching, internal parasitic worms (tape worms) & promote lactation | F | Infusions | Oral |  |  |
|  |  |  |  |  | Sh | Severe colic, regulation of menses in women &prevent abortion | D | Powder-Infusions | Oral |  |  |
|  |  |  |  |  | R | Cancer, kidney, women reproductive issues, diarrhea, skin infections, burns, rheumatism, malaria, intestinal parasites, inflammation, fever, aid labour during delivery, urinary tract respiratory, kidney problems, stomachache, inflammation, wounds & swellings | F | Decoction | Oral |  |  |
|  |  |  |  |  | L/R | Jaundice, malaria, urinary problems, syphilis, gonorrhea, constipation; stomach pains, tumours, infertility for womb cleansing, migraines headaches, rheumatism, fever, oedema, blood pressure, cough, uterine issues & stomachaches | F | Decoction | Oral |  |  |
|  |  |  |  |  | L | Eye infections and earaches | F | Juice | Drops |  |  |
|  | *Zanthoxylum usambarense* (Engl.) Kokwaro  (MNCY- 126) | NE | Ketemwet/  Kipkombotiet | C | R | Hypertension, toothache, jaundice, malaria, urinary; kidney problems, syphilis, gonorrhea, vaginal discharge, menstrual problems & stomachache | F | Decoction | Oral |  | Rutaceae |
|  |  |  |  |  | B | Expel tapeworms | F | Decoction | Oral |  |  |
|  |  |  |  |  | L | Relieving migraine headache and other forms of headaches; rheumatic pain | F | Paste | Massage |  |  |
|  |  |  |  |  |  | Enema to treat rectal prolapse, haemorrhoids & hernia | F | Paste | Suppositories |  |  |
|  | *Erigeron canadensis* L.  (MNCY- 164) | NE | Ketungutwet | H | Sh | Wounds, burns, boils & abscesses |  | Paste-Dressing | Apply | Ghee | Asteraceae |
|  |  |  |  |  | L | Fever, coughs, chest pain, bronchial infections, colds & flu | F | Decoction | Oral |  |  |
|  |  |  |  |  | R | Abdominal pains, colds, diarrhoea, bleeding, liver ailments, headache & stomachache | F | Decoction | Oral |  |  |
|  |  |  |  |  | B | Dysentery, wounds, abdominal pains, heat rash, stomachache, diarrhoea, joint pains, typhoid, gout & arthritis | F | Decoction | Oral |  |  |
|  |  |  |  |  | R/B | Headache, stomach ache, bronchial affections, cough, asthma, throat inflammations, sore throat, cold, fever; wounds of the gastrointestinal tract; respiratory and urinary system | F | Decoction | Oral |  |  |
|  | *Combretum collinum* Fresen  (MNCY- 245) | LC | Ketyeng'wet | ST | R | Indigestion and as a treatment for indigestion, constipation, jaundice, malaria, urinary and kidney problems; syphilis and gonorrhoea; foul vaginal discharge, menstrual problems & stomachache | F | Decoction | Oral |  | Combretaceae |
|  |  |  |  |  |  | Wounds and inflamed tumours | F | Decoction | Apply |  |  |
|  |  |  |  |  |  | Rheumatism, arthritis & joint pains | F | Extract | Massage |  |  |
|  |  |  |  |  | Tw/Se | Intestinal worms. | F | Extract | Oral |  |  |
|  |  |  |  |  | FL | Coughs, colds, sore throat and fever | F | Infusion | Teas |  |  |
|  | *Maesa lanceolata* Forssk.  (MNCY- 25) | LC | Kibabustanyit | S/ST | Fr | Induce vomiting, sore throat or eaten to cure tapeworm. | F | Extract | Oral |  | Primulaceae |
|  |  |  |  |  | R | Lower pain during pregnancy, stomach problems in children & convulsions in children. | F | Decoction | Oral |  |  |
|  |  |  |  |  | R | Treat “Kiptumarit” (Bell’s palsy) | F | Decoction | Oral |  |  |
|  | *Girardinia diversifolia* (Link) Friis  (MNCY- 60) | NE | Kibiserit | H | R/L | Liver diseases. | F | Extract | Oral |  | Urticaceae |
|  |  |  |  |  | WP | Fever | F | Decoction | Oral |  |  |
|  |  |  |  |  |  | Ringworm and eczema. | D | Ash | Apply |  |  |
|  |  |  |  |  |  | Antidote against snakebites | F | Extract | Apply |  |  |
|  |  |  |  |  | R | Constipation | F | Juice | Oral |  |  |
|  |  |  |  |  | L | Headache, fever & swollen joint | F | Decoction | Oral |  |  |
|  |  |  |  |  | R | Gain strength. | F | Decoction | Oral | Goat’s bone soup |  |
|  |  |  |  |  |  | Heartburn relief | F | Decoction | Oral |  |  |
|  | *Embelia schimperi* Vatke  (MNCY- 181) | LC | Kibong'ong'inik | S/ST/C | L | Anthelmintic, especially for tapeworm. | F | Decoction | Oral |  | Primulaceae |
|  |  |  |  |  | Fr | Induce vomiting & an anthelminthic | F | Extract | Oral |  |  |
|  |  |  |  |  | Fr/R | Intestinal worms | F | Decoction/Infusion | Oral |  |  |
|  | *Brillantaisia vogeliana*Benth.  (MNCY- 106) | NE | Kibong'yot | H | Tu | Reproductive issues of fertility | F | Decoction/Infusion | Oral |  | Acanthaceae |
|  | *Bersama abyssinica* Fresen.  (MNCY- 34) | LC | Kibuimetiet | T | L | Leaves are crushed and used as snuff for colds or | F | Extract | Sniff |  | Francoaceae |
|  |  |  |  |  | B | Anthelmintic for small children. | D | Powder/Decoction | Leak/Oral |  |  |
|  |  |  |  |  | R | Epilepsy, haemorrhoids, reproductive issues in women; washing the wounds | F | Decoction | Oral |  |  |
|  |  |  |  |  | Tw | Dysentery, stomachache & roundworm | F | Extract | Oral |  |  |
|  |  |  |  |  | L | Aphrodisiac. | F | Juice | Oral |  |  |
|  | *Lactuca macrophylla*(Willd.) A.Gray  (MNCY- 24) | NE | Kimogit | H | L | Stomach upsets, alleviating stomach pains, sores on the throat, mouth and body & to relieve fever | F | Extract | Oral |  | Asteraceae |
|  |  |  |  |  | Tu | Reproductive issues of infertility in women, measles, malaria, hernia, stomach pain, blood pressure or body temperature problems, boils, other skin afflictions, chicken pox. | F | Decoction | Oral |  |  |
|  |  |  |  |  | L | Diabetes | F | Decoction | Oral |  |  |
|  | *Vangueria infausta* Burch.  (MNCY- 70) | LC | Kimolwet | S/ST | R/L | Malaria, chest ailments like pneumonia, to bowel evacuation, abdominal pains, to slow down palpitation & ringworms | F | Infusion | Oral |  | Rubiaceae |
|  |  |  |  |  | B | Bloody diarrhoea, syphilis, gastro-intestinal disorders, malaria, pneumonia, cough, menstrual problems, aphrodisiac, parasitic worms, chest complaints, snake bites, infertility, fever, candidiasis & abdominal pains | F | Decoction | Oral |  |  |
|  |  |  |  |  | Fr/L/R | Clear parasitic worms | F | Decoction | Oral |  |  |
|  |  |  |  |  | R/Se/  Fr | Menstrual problems | F | Decoction | Oral |  |  |
|  |  |  |  |  | L | Abscesses and swellings, lactational mastitis and toothache. | F | Decoction | Oral |  |  |
|  |  |  |  |  | B/L/R | Asthma, vaginal candidiasis or oral candidiasis, chest complaints, cough diarrhoea and stomach problems, induce labour; skin blisters & blood pressure. | F | Infusion | Oral |  |  |
|  |  |  |  |  | L/R | Aphrodisiac, to induce vomiting, manage diabetes, hernia, cold, headache & alleviate fever | F | Decoction | Oral |  |  |
|  |  |  |  |  | R | Painful menstruation | F | Decoction | Oral |  |  |
|  |  |  |  |  | L | Toothache | F | Infusion | Oral |  |  |
|  |  |  |  |  | L/Tw | treat swellings and a massage on the affected parts of the body | F | Decoction | Oral |  |  |
|  |  |  |  |  | R | Heart ailments | F | Decoction | Oral |  |  |
|  |  |  |  |  | L/R/  Se | Pneumonia | F | Decoction | Oral |  |  |
|  |  |  |  |  | R | Infertility in women, epilepsy, measles; nervous system disorders, inflammation of the umbilical cord and vaginal discharge, male virility and stomach ulcers; pre-natal care in infants in colic & gastrointestinal upsets | F | Decoction | Oral |  |  |
|  |  |  |  |  |  | Snake repellent or remedy for snake bites | F | Decoction | Scent/Apply |  |  |
|  | *Bidens pilosa* L.  (MNCY- 249) | NE | Kipkoleitet | H | WP | Headache, antidote, diarrhea, arthritis, diabetes, stomach and bowel cancers; heart diseases, liver diseases, high blood pressure and malaria | F | Decoction | Oral |  | Asteraceae |
|  |  |  |  |  | L | Malaria, stomach ulcers, mouth ulceration, diarrhea & hangover | F | Extract | Oral |  |  |
|  |  |  |  |  | L | Sore eyes, abdominal distress, swollen glands, burns, conjunctivitis, eye irritation, ulcers or rubbed between palms & ringworms | F | Macerated- Juice | Oral |  |  |
|  |  |  |  |  | Sh/Bd/FL | Its shoots, buds and flowers extract or infusion of the dry shoots and flowers is used to manage stomach ailments, haemorrhoids and diabetes. | F | Extract | Oral |  |  |
|  |  |  |  |  | Sh | Stomach ailments, haemorrhoids & diabetes | D | Infusion | Oral |  |  |
|  |  |  |  |  | L | Headaches, ear infections, kidney problems & flatulence | F | Decoction | Oral |  |  |
|  |  |  |  |  | AP | Inflammations, high blood pressure, ulcers & diabetes | F | Extract/Infusion | Oral |  |  |
|  |  |  |  |  | L | Anaemia, body weakness & heart diseases | F | Cooked- Vegetable | Oral |  |  |
|  |  |  |  |  |  | Protect the liver & hypertension | F | Teas-Infusion | Oral |  |  |
|  |  |  |  |  | Se | Sedative and pain killer | D | Infusion | Oral |  |  |
|  |  |  |  |  | L | Ear and eye infections. | F | Sap | Drops |  |  |
|  | *Zanthoxylum asiaticum*(L.) Appelhans, Groppo & J.Wen  (MNCY- 31) | NE | Kipkoskosit | S/C | L/Tw | The leaves and branches are boiled and the steam used as vapour both for nasal, lung diseases nasal and bronchial pains. | F | Decoction-Steam | Inhalation |  | Rutaceae |
|  |  |  |  |  | L | Rheumatism, treat, stomachache, snake bites, stomach problems, coughs, fever, diabetes mellitus poisonous snakebites, nausea, bronchitis, wounds, contaminated ulcers, epilepsy, gonorrhea, general body weakness after illness cancer & in rituals | F | Decoction | Oral |  |  |
|  |  |  |  |  | Fr | Coughs, malaria, chest pain & colds | F | Extract/Decoction | Oral |  |  |
|  |  |  |  |  | R | Coughs and for stomachache | F | Chew-Juice | Oral |  |  |
|  |  |  |  |  |  | Indigestion, stomach upsets & stomachaches | F | Decoction | Oral | Soup |  |
|  |  |  |  |  |  | Paralysis caused by snake-bite, evacuate the stomach & nervous disorders | F | Juice | Rub-topical/  Oral |  |  |
|  |  |  |  |  | R | Malaria & amoeba infection | F | Decoction | Oral | Milk |  |
|  |  |  |  |  |  | Relieve pain, inflammation, malaria,indigestion, fever, chest pains, food poisoning, sore throat, indigestion, influenza, rheumatism, fever, cholera, diarrhea, skin and lung diseases, arthritis, sprains, abrasions; sharp, aching, radiating, burning, or stabbing pain around the chest area; cough, dysentery, an ache localized in the stomach or abdominal region and calming down nervous activity during convalescence from fevers. |  |  |  |  |  |
|  |  |  |  |  | Fr | Stimulant, liniment for arthritis & alleviate pain | F | Juice | Oral |  |  |
|  |  |  |  |  | Rb | Bitter stomachic, tonic & febrifuge | F | Infusion | Oral |  |  |
|  |  |  |  |  | L | Stomach disorders. | F | Extract | Oral |  |  |
|  |  |  |  |  | Rb | Stimulating body strength & carminative | F | Infusion/Extract | Oral |  |  |
|  |  |  |  |  | L | Furuncles /over afflicted areas | F | Poultice | Apply |  |  |
|  | *Crotalaria laburnifolia* L.  (MNCY- 26) | NE | Kipkurkuriet | S | R | Throat infection and inflammation. | F | Chew-Juice | Oral |  | Fabaceae |
|  |  |  |  |  |  | Aphrodisiac, gonorrhea, fertility, painful urination & urogenital ailments | F | Extract | Oral |  |  |
|  |  |  |  |  | L | Snake-bite | F | Paste | Apply |  |  |
|  |  |  |  |  | WP | Sore throats and mouth inflammations | F | Infusion | Gargle-Oral |  |  |
|  |  |  |  |  | Se | Blood-purifying effect, to treat sore throats, skin ailments & promote menstrual discharge | D | Extract | Oral |  |  |
|  |  |  |  |  | R | Red sores on the skin & scabies | F | Decoction | Apply |  |  |
|  |  |  |  |  |  | Production of excess saliva, jaundice, cough, bad digestion, constipation, irritable bowel, fever, cardiac abnormalities; oral diseases, skin allergies, wound healing, liver protection, diabetes, antipyretic & on depression | F | Decoction | Oral |  |  |
|  |  |  |  |  | WP | Warts, in particular those on the sole of the toes | F | Paste | Topical |  |  |
|  | ***Lannea schimperi* (A.Rich.)Engl.  (MNCY- 188) | NE | Kipng’etingwet | T | R | Oral cavity | F | Decoction | Gargle-Oral |  | Anacardiaceae |
|  |  |  |  |  |  | Constipation, sore throat, toothache, yellow fever, induces labour, syphilis, intestinal parasites & relieves toothaches | F | Decoction | Oral |  |  |
|  |  |  |  |  | R/B | Colds, chest troubles, coughs, throat inflammation and colds. | F | Decoction | Oral |  |  |
|  |  |  |  |  | L | Bloody diarrhea | F | Decoction | Oral |  |  |
|  |  |  |  |  | L | Stop severe diarrhoea normally accompanied with blood | F | Extract | Oral |  |  |
|  |  |  |  |  | B | Bark decoction of is used for tuberculosis, backache, chronic diarrhea, diabetes, epilepsy, general body weakness and malaria | F | Decoction | Oral |  |  |
|  |  |  |  |  | B/R | Abdominal chest pains | F | Decoction | Oral |  |  |
|  |  |  |  |  | R/B/ L Se | Cough | F | Decoction | Oral |  |  |
|  |  |  |  |  | L/B | Skin infections and rashes | F | Infusion | Oral |  |  |
|  |  |  |  |  | L | Chronic coughs | F | Infusion | Oral |  |  |
|  |  |  |  |  | B/R/L | Anemia, mental disorders, snake bites & tumors | F | Infusion | Oral |  |  |
|  |  |  |  |  | R/SB | Diarrhea & dysentery | F | Decoction | Oral |  |  |
|  |  |  |  |  | B/R/L | Stomach problems | F | Infusion | Oral |  |  |
|  | *Scepocarpus hypselodendron*(Hochst. ex A.Rich.) T.Wells & A.K.Monro  (MNCY- 46) | NE | Kipsotiet | L/C | R | Infertility & associated reproductive ailments in women | F | Decoction | Oral |  | Urticaceae |
|  |  |  |  |  | St | Given to pregnant women who feel pain in the lower part of the belly | F | Decoction | Oral | Milk |  |
|  | *Sida cordifolia* L  (MNCY- 98) | NE | Kiptutung'it | H/SS | R | The roots are pounded, mixed with fat and rubbed in as a cure for low back pain, skin for numbness, nerve pain, muscle cramps, skin disorders, tumours, joint diseases, wounds, ulcers, insect stings, snakebite and for body massage | F | Paste | Apply/  Massage | Fat | Malvaceae |
|  |  |  |  |  | B | Stimulata menstruation | F | Extract | Oral |  |  |
|  |  |  |  |  | L | Diarrhoea | F | Infusion | Oral |  |  |
|  |  |  |  |  | R | Asthma, allergies, fatigue, erectile dysfunction, common cold, bronchial asthma, tuberculosis, colds, flu, promote sweating, headaches, nasal congestion, cough and wheezing, urinary infections, sores in the mouth & fluid retention | F | Decoction | Oral |  |  |
|  |  |  |  |  | WP | Heart disease, stroke, paralysis, tissue pain and swelling, nerve pain, achy joints, stimulant, painkiller; restore general body health; increase urine production and raise sexual arousal | F | Decoction | Oral |  |  |
|  |  |  |  |  | R | Relieve on and off fever, promote strong bones, treat vaginal discharges | F | Decoction | Oral |  |  |
|  | *Aeschynomene schimperi* Hochst. ex A.Rich.  (MNCY- 187) | LC | Koibeiyot | S/ST | R | Stop palpitations | F | Juice | Oral |  | Fabaceae |
|  |  |  |  |  |  | Relieving arthritis pain, cleaning toxins, resolving swellings; removing water accumulation; relieving pain due to acute soft tissue injury, vitalizing blood, controlling pain; controlling bleeding, resolving phlegm accumulation, cough & lung disorders | F | Decoction | Oral |  |  |
|  |  |  |  |  | St | Pregnant women who feel pain in the lower part of the belly | F | Decoction | Oral | Milk |  |
|  |  |  |  |  | L | Sick chicken | F | Infusion | Oral |  |  |
|  |  |  |  |  | B/L/FL/Fr | Multifactorial diseases like leprosy, gout, acute rheumatic fever, sores, boils, bloody diarrhea and vaginitis | F | Decoction | Oral |  |  |
|  | *Zanthoxylum chevalieri P.G.Waterman*  (MNCY- 14) | VU | Kokchat | S/ST | L | Migraine as a pain-killer |  | Powder | Snuff |  | Rutaceae |
|  |  |  |  |  | SB/R | Cancer | F | Decoction | Oral |  |  |
|  |  |  |  |  | Fr | Rheumatism | F | Pulp | Oral |  |  |
|  |  |  |  |  | SB | Contraceptive to prevent pregnancy in women | F | Decoction | Oral | Porridge |  |
|  |  |  |  |  | R | The strong-smelling roots are sniffed to promote menstrual flow. | F | Maceration | Snuff |  |  |
|  |  |  |  |  | B | Scabies | F | Extract | Apply |  |  |
|  |  |  |  |  | R | Head-ache, rheumatic, lumbar & sharp strong pain on the chest | F | Ointment | Apply-Massage |  |  |
|  |  |  |  |  | Rb | Enema to treat gonorrhoea. | F | Paste | Apply |  |  |
|  |  |  |  |  | B | Aphrodisiac, treat intestinal worms, dysentery & treat venereal diseases | F | Decoction | Oral |  |  |
|  |  |  |  |  | L | Sores | F | Pulp | Apply |  |  |
|  |  |  |  |  |  | Mouth-wash to soothe toothache r | F | Decoction | Gargle |  |  |
|  |  |  |  |  |  | Relieve tooth pain | F | Extract | Placed on tooth |  |  |
|  |  |  |  |  | R | Chest pain, body pain & heart palpitations | F | Decoction | Oral |  |  |
|  |  |  |  |  | RB | Swollen legs, toothache, sexual impotence, gonorrhea, malaria, painful menstrual flow, abdominal pain & snake bites | F | Extract | Oral |  |  |
|  |  |  |  |  | R | Mouthwash & against a sore throat. | F | Decoction | Oral/Gargle |  |  |
|  |  |  |  |  | B | Eye infections- notably conjunctivitis with pus |  | Pulp-Extract | Drops |  |  |
|  |  |  |  |  | R/SB | Sore gums, toothache & dental caries | F | Chew-Extract | Oral |  |  |
|  |  |  |  |  | B | Severe cough, including asthma | F | Chew-Extract | Oral |  |  |
|  |  |  |  |  | B | Hypertension | F | Decoction | Oral |  |  |
|  | *Billieturnera helleri*(Rose ex A.Heller) Fryxell (MNCY- 112) | NE | Korkoriet/ Chepkurkuriet | H/SS | L | Skin rashes due to measles; other skin rashes | D | Powder | Topical | Jelly | Malvaceae |
|  |  |  |  |  | R | Sore throat, throat inflammation & oral cavity hygiene | F | Chew-Juice | Oral |  |  |
|  |  |  |  |  |  | Pregnant women to quieten a foetus which moves too much in the womb; pain during pregnancy & maintaining the pregnancy to term. | F | Decoction | Oral | Milk |  |
|  |  |  |  |  | L | Coughs, common colds, epilepsy and infant cerebral malaria | F | Extract | Snuff |  |  |
|  |  |  |  |  |  | Induce labour during child birth, ease menstrual pains, dizziness; headaches, disinfect umbilical cord wounds & sore throat | F | Decoction | Oral |  |  |
|  |  |  |  |  |  | Enhances manhood | F | Chew-Extract | Oral |  |  |
|  |  |  |  |  |  | Align the bones in treatment of fractures and sprains (bone setting). |  | Pound-Paste | Topical-Tie | Ghee |  |
|  |  |  |  |  |  | Prevention of allergies due to meat consumption | D | Teas-infusion | Oral |  |  |
|  |  |  |  |  | S | Tooth brush for dental care | F | Extract | Oral |  |  |
|  | *Rhamnus prinioides* L’Hér.  (MNCY-35) | LC | Kosisitiet | S/ST | L | Skin infections like eczema ringworm & dandruff | D | Powder-Ointment | Topical | Butter | Rhamnaceae |
|  |  |  |  |  |  | Blood purification & detoxification | F | Infusion | Oral |  |  |
|  |  |  |  |  | R | Gonorrhea, syphilis, amoeba infection, colic, dysentery & stomachaches. | F | Decoction | Oral |  |  |
|  |  |  |  |  |  | Rheumatism in the legs, general joint pain & arthritis | F | Decoction | Oral | Bone soup |  |
|  |  |  |  |  |  | Skin complaints digestive disorders; fevers & blood purifier; colds, flu/cold, brucellosis, rheumatism, pneumonia, stomach-ache, back pain, gonorrhea and malnutrition | F | Decoction | Oral |  |  |
|  |  |  |  |  |  | Blood purifier | F | Decoction | Oral | Milk |  |
|  |  |  |  |  | L | Alleviate joint sprains |  | Liniment | Apply |  |  |
|  |  |  |  |  | L/St | Therapy of tonsils, sore throat, arthritis, back pain, brucellosis, flu, common cold, indigestion, loss of appetite; pneumonia, fatigue, STDs, stomach ache and ear; nose and throat infections | F | Decoction | Oral |  |  |
|  |  |  |  |  | L | Chest pain, stomach complications, fever, common cold, diarrhea, malaria & ringworm infections | F | Decoction | Oral |  |  |
|  | *Gymnosporia heterophylla*(Eckl. & Zeyh.) Loes.  (MNCY- 110) | LC | Kukerwet | S/ST | R | STI's, severe and frequent menstrual cramps, pain during menstrual period & preventing abortion | F | Decoction | Oral |  | Celastraceae |
|  |  |  |  |  | St/R | Dysentery, snake bites, wounds & respiratory diseases | F | Decoction | Oral |  |  |
|  |  |  |  |  | R | Digestive system inflammation and pain, chest pain, rheumatism, arthritis, hemorrhoids, skin rashes, gastritis, ulcers, indigestion, passing gas, body pain, wounds, back pain, stomach pain, sore throat, rheumatism, gastrointestinal diseases, chest pain, malaria, fever, wounds, snake bites, sore throat & stomach diseases | F | Decoction | Oral |  |  |
|  | *Croton dichogamus* Pax.  (MNCY- 92) | LC | Kulelwet | T | L | Fever and headache. | D | Smoke | Inhalation |  | Euphorbiaceae |
|  |  |  |  |  |  | Excellent remedy for chest ailments, asthma & respiratory ailments | D | Cigarette | Smoked |  |  |
|  |  |  |  |  | R | Restoration of general body health. | F | Decoction | Oral | Soup-Goat’s meat |  |
|  |  |  |  |  | R/B | Stomachache, stomach pain, indigestion & as a remedy for stomach diseases | F | Decoction | Oral |  |  |
|  |  |  |  |  | R | Cardiovascular diseases caused by high levels of cholesterols, stomachaches, arthritis, asthma & other respiratory illnesses | F | Decoction | Oral | Milk and Meat |  |
|  |  |  |  |  |  | Tuberculosis | D | Powder | Oral | Porridge and Honey |  |
|  |  |  |  |  | L | restore body strength, treat malaria, urinary tract infections & toothaches | F | Decoction | Oral |  |  |
|  |  |  |  |  | R | Tuberculosis, impotence, infertility, chest pains, respiratory tract infections, stomachaches, fever, STDs such as syphilis gonorrhea, impotence, arthritis, toothache & malaria | F | Decoction | Oral |  |  |
|  |  |  |  |  | St/L | Back-pains, malaria, stomachache, chest problems, fever, oedema & cough | F | Infusion | Oral |  |  |
|  |  |  |  |  | R/FL/L | Relieve abdominal pain, oral thrush & wheezing | F | Decoction | Oral |  |  |
|  |  |  |  |  | R | Vaginal application to enhance female reproductivity | F | Paste | Apply |  |  |
|  | ** *Euphorbia candelabrum*Welw.  (MNCY- 132) | LC | Kuresyet | T | SB | Clear out the afterbirth | F | Extract | Oral |  | Euphorbiaceae |
|  |  |  |  |  | R | Skin rash, itchy skin & eczema | F | Decoction | Oral |  |  |
|  |  |  |  |  | S | Malaria, breathing disorders including asthma, bronchitis and chest congestion; treat mucus in the nose and throat, throat spasms, fever, induce vomiting and tumors | F | Extract | Oral |  |  |
|  |  |  |  |  | St | Purgative to cure syphilis, treating worms, severe diarrhea, gonorrhea, and digestive problems |  | Sap | Oral | Honey |  |
|  | *Vepris nobilis*(Delile) Mziray  (MNCY- 201) | LC | Kuriot | T | L/R | Chest infections, pneumonia, reduce fever, malaria, headache, joint pains, common cold, pneumonia, intestinal worms, chest pain, pneumonia & arthritis | F | Decoction | Oral | Honey | Rutaceae |
|  |  |  |  |  | R | Anthelminthic | F | Decoction | Oral |  |  |
|  |  |  |  |  | L/St | Fever and relieve pain | F | Steam | Inhalation |  |  |
|  |  |  |  |  | B | Gonorrhoea stomach ache and cough | F | Decoction | Oral |  |  |
|  |  |  |  |  | R | Cancer and rheumatism | F | Decoction | Oral |  |  |
|  |  |  |  |  | St | Dental care | F | Extract | Oral |  |  |
|  | *Clutia pulchella*L. (MNCY- 200) | NE | Kurmenyat | S | R | Influenza, colds, coughs, chest pain & indigestion | F | Extract | Oral |  | Peraceae |
|  |  |  |  |  |  | Headache, stomachache and malaria. | F | Decoction | Oral | Mutton soup |  |
|  |  |  |  |  |  | Malaria | F | Steam | Inhalation |  |  |
|  |  |  |  |  | L | Anti-inflammatory agent, sores & ulcerations | F | Decoction | Apply |  |  |
|  |  |  |  |  |  | Liver ailments | F | Decoction | Oral |  |  |
|  |  |  |  |  | R | Liver pains | F | Decoction | Oral | Milk |  |
|  |  |  |  |  |  | Threatened abortion, convulsions, influenza, enlarged spleen & management of habitual miscarriage | F | Extract | Oral |  |  |
|  |  |  |  |  |  | Stomachache and chest pains | F | Infusion | Oral |  |  |
|  | *Kalanchoe crenata* (Andrews) Haw.  (MNCY- 15) | NE | Kuserwet | S | L | Skin infections |  | Roasted-Ash | Apply |  | Crassulaceae |
|  |  |  |  |  |  | Liver diseases & respiratory tract infections | F | Macerated-Extract | Oral |  |  |
|  |  |  |  |  |  | Chicken pox, ear infections, cough, asthma, palpitations, headache, convulsions & general body weakness after illness | F | Juice | Oral |  |  |
|  |  |  |  |  |  | Burns, abscesses, ulcers, insect bites, rheumatism & inflammation | F | Macerated -Paste | Apply |  |  |
|  |  |  |  |  | R | Back pain relief diabetes, dissolving kidney stones, facilitates the dropping of the placenta, gastric ulcer & edema of legs | F | Decoction | Oral |  |  |
|  |  |  |  |  | L | Fever relief, expel worms, bronchitis, pneumonia and others forms of respiratory tract infections such as asthma | F | Infusion | Oral |  |  |
|  |  |  |  |  |  | Wounds, boils & insect bites | F | Macerated-Extract | Apply |  |  |
|  |  |  |  |  |  | Injuries | F | Heated Leaves | Massage |  |  |
|  | *Hoffmannanthus abbotianus*(O.Hoffm.) H.Rob., S.C.Keeley & Skvarla  (MNCY- 91) | NE | Kwamberiet | S | L | Fever | F | Pounded-Juice | Oral |  | Asteraceae |
|  |  |  |  |  |  | Clear spots on the body, nausea and liable to vomit | F | Infusion | Bath |  |  |
|  |  |  |  |  |  | Malaria, backaches & chickenpox | F | Infusion | Oral |  |  |
|  |  |  |  |  | R | Stomachache & induce vomiting | F | Chew-Extract | Oral |  |  |
|  |  |  |  |  | L | Eyes infections |  | Juice | Drops |  |  |
|  |  |  |  |  | R | Pneumonia management, chest pain, colds, influenza & throat sores | F | Decoction | Oral |  |  |
|  |  |  |  |  | L | Stomach evacuation | F | Decoction | Oral |  |  |
|  | *Cirsium vulgare* (Savi) Ten.  (MNCY- 153) | NE | Kwermetyot/  Kipkermetyot | H | R/L | Stiffness especially of the neck, seizures & nervous disorders | F | Infusion | Oral |  | Asteraceae |
|  |  |  |  |  | L | Boost urination, relief fever & liver complaints | F | Decoction | Oral |  |  |
|  |  |  |  |  | WP | Rheumatic joint pains & bleeding haemorrhoids | F | Decoction | Oral |  |  |
|  |  |  |  |  | R | Aching jaws | F | Paste | Topical-Poultice |  |  |
|  |  |  |  |  |  | Lower blood sugar and cholesterol levels, blood pressure; chest pains, syphilis, muscular inflammation & relief hiccups | F | Decoction | Oral |  |  |
|  | *Solanum incanum* L. (MNCY- 122) | LC | Labotwet | S | R | Abdominal pains, fever, stomachache & indigestion | F | Decoction | Oral |  | Solanaceae |
|  |  |  |  |  |  | Toothache | F | Extract | Rubbing on tooth |  |  |
|  |  |  |  |  | Sh | Snake bite | F | Chew-Paste | Rub |  |  |
|  |  |  |  |  | L | Earache | F | Infusion | Drops/Appy |  |  |
|  |  |  |  |  | Fr | Fresh cuts or wounds; ringworm, skin diseases | F | Pulp | Apply |  |  |
|  |  |  |  |  |  | Induce vomiting in children Precaution should be taken | F | Juice | Oral |  |  |
|  | *Solanum nigriviolaceum*Bitter  (MNCY- 102) | NE | Labotwet | S |  | Asthma, to clear mucus on the back of the nose, throat and sinuses, oedema, chest pain, chronic fever, colic, dry and irregular cough, difficult urination & worm infestation | F | Infusion | Oral |  | Solanaceae |
|  |  |  |  |  | R | Oedema, sore throat, stomachache, malaria, common cold, blood pressure, diabetes, headache, painful menstruation, liver pain, pneumonia & rheumatism | F | Decoction | Oral |  |  |
|  |  |  |  |  |  | Cough remedy | F | Roast-/Extract | Chew-Oral |  |  |
|  |  |  |  |  | Fr | Skin infections | F | Extracts | Apply |  |  |
|  | *Solanum melongena*L.  (MNCY- 233) | LC | Labotwet ap teta | S | R | Gonorrhea, expectorant, stimulant, bronchitis, itching, general body aches, asthma, mouthwash, wounds, clear mucus on sinuses, heart conditions, cancer, fever, colic, insect stings, difficult urination, worm infestation, erectile failure, boost appetite, abdominal pain, oedema & deworming | F | Decoction | Oral |  |  |
|  |  |  |  |  |  | Relive toothache, jigger wounds | F | Extract | Apply |  |  |
|  |  |  |  |  | Fr | Cancer wounds & ringworm | F | Juice | Apply |  |  |
|  |  |  |  |  | Be | Chest complaints |  | Chew-Pulp | Oral |  |  |
|  | *Solanum mauense* Bitter  (MNCY- 250) | LC | Labotwet ne tenden | S | Fr | Epiglottis issues, gout, arthritis, ring worm and earache. | F | Juice | Oral |  | Solanaceae |
|  |  |  |  |  | Be | Pneumonia, colds, coughs, asthma, indigestion, gas, flatulence, dysentery, expel worms & boost appetite | F | Cooked pulp | Oral |  |  |
|  |  |  |  |  | R | Treat people and animals with anthrax, pneumonia, aching teeth, stomachache, tonsillitis, worms, pain, inflammation & fever | F | Decoction | Oral |  |  |
|  |  |  |  |  | Fr | Induce vomiting, manage TB & chest ailments |  | Infusion | Oral | Honey |  |
|  | *Solanum aculeatissimum* Jacq.  (MNCY- 149) | NE | Labotwetab kimagetiet | S | R | Gonorrhoea. | F | Decoction | Oral | Meat broth | Solanaceae |
|  |  |  |  |  |  | Stomach disorders like indigestion, flatulence, gas, heartburns & treatment of various cancers. | F | Decoction | Oral |  |  |
|  |  |  |  |  | Fr/L | Cancer, indigestion, and stomach disorders | F | Decoction | Oral |  |  |
|  |  |  |  |  | Be | Wounds, gonorrhea, syphilis, swollen joints in fingers, toothaches, bronchitis & ringworm | F/C | Pulp | Oral/Apply |  |  |
|  |  |  |  |  |  | Improve erection & breast cancer |  | Paste | Apply |  |  |
|  |  |  |  |  | R | Dysmenorrhea & bronchitis | F | Decoction | Oral |  |  |
|  | *Mimusops bagshawei* S. Moore  (MNCY- 113) | LC | Lalyat/leliat | T | L/B | Leaf and bark decoction is used to treat malaria. | F | Decoction | Oral |  | Sapotaceae |
|  |  |  |  |  | Fr | Boost appetite and digestion | F | Extract | Oral |  |  |
|  |  |  |  |  | B | Wounds and sores, bleeding gums, sexually transmitted infections (STIs), especially gonorrhea, inflammation & tuberculosis | F | Decoction | Oral |  |  |
|  |  |  |  |  | B/R | Wounds & ulcers | F | Decoction | Wash |  |  |
|  |  |  |  |  | R | Candidiasis, tuberculosis, eating disorders, womb problems & STIs | F | Infusion | Oral |  |  |
|  | *Syzygium guineense* (Willd.) DC  (MNCY- 67) | LC | Lamaiyuetab kewet | T | R | Asthma, cancer, and infertility in women, wound, hypertension, diabetes, pain, inflammation, tuberculosis, respiratory infections, stomach problems & diarrhoea | F | Decoction | Oral |  | Myrtaceae |
|  |  |  |  |  | L | Diarrhoea, abdominal pains, induce vomiting, treatment of burns, wounds, relieve pain in dental care, tooth infections; toothache hemorrhages, syphilis, leprosy, wounds, ulcers; lung diseases, mouth ulcers & irregular menstruation | F | Decoction | Oral |  |  |
|  |  |  |  |  | B | Stomachache; worm infection, induce vomiting | F | Pounded-Extract | Oral |  |  |
|  |  |  |  |  |  | Strength & infertility | F | Decoction | Oral | Goat's bone soup |  |
|  |  |  |  |  |  | Abdominal pains, arthritis, rheumatism, venereal diseases, malaria, nasopharyngeal infections, constipation, diarrhea, dysentery, pain & circulatory disorders | F | Infusion | Oral |  |  |
|  |  |  |  |  | R/B | Stomachaches, colon cancers, indigestion, diarrhoea, breast & venereal diseases. | F | Infusion | Oral |  |  |
|  |  |  |  |  | Fr | Coughs and colds, fever, diarrhea and dysentery | F | Extract | Oral |  |  |
|  |  |  |  |  | R/St | Stomachache; worm infection, induce vomiting | F | Decoction | Oral |  |  |
|  | ***Syzygium cordatum*** Hochst. (MNCY- 115) | LC | Lamaiywet | T | B | Respiratory ailments, stomach complaints, burns, sores, wounds, colds, cough, respiratory complaints, sexually transmitted infections (STIs), tuberculosis, fever & malaria | F | Decoction | Oral |  | Myrtaceae |
|  |  |  |  |  | SB/R | Diarrhea, dysentery & gastro-intestinal complications | F | Infusion | Oral |  |  |
|  |  |  |  |  | L/B | Stomachache | F | Infusion | Oral |  |  |
|  |  |  |  |  | B | Amenorrhea, chest complaints, colds, fever, headache & respiratory ailments | F | Decoction | Oral |  |  |
|  |  |  |  |  | L | Leave decoction is used on malaria, dry cough, topically on skin rash, anaemia & liver issues | F | Decoction | Oral |  |  |
|  |  |  |  |  | L | Snake bites | F | Extract | Apply |  |  |
|  |  |  |  |  | L/B | Wounds ulcers & mouth wash | F | Infusion | Apply/Gargle |  |  |
|  | *Carissa spinarum*L.  (MNCY- 240) | LC | Legetetwet (Tamuryekiat) | S/ST | R | Indigestion, painkiller, venereal diseases, malaria, polio symptoms, gonorrhea, libido, restore virility, to treat gastric ulcers, expectorant, blood pressure, headache, chest complaints, syphilis, rheumatism, gonorrhoea, inflammation, arthritis, epilepsy, cancer disease & relieve of pain | F | Decoction | Oral |  | Apocynaceae |
|  |  |  |  |  | R | Chest pains, stomachache, diarrhea, malaria & coughs remedy | F | Infusion | Oral |  |  |
|  |  |  |  |  | Fr | Dysentery & stomach complaints | F | Pulp-Juice | Oral |  |  |
|  |  |  |  |  | R | Chest complaints, colds, headache, chest complains, rheumatism, oedema, gonorrhoea, syphilis, fever, sickle cell anaemia, cough, ulcer, toothache, fever, cold and cough; ulcer, toothache, rheumatism, diarrhoea, epilepsy, anaemia, cardiovascular diseases & worm infestation | D | Powder-Decoction | Oral |  |  |
|  |  |  |  |  | R | Cataract problems | F | Infusion | Drops |  |  |
|  |  |  |  |  | R | Detox as well as relief for headache | F | Steam | Inhalation |  |  |
|  | ***Vachellia sieberiana*(DC.) Kyal. & Boatwr.** (MNCY- 229) | LC | Leldet | T | SB | Tapeworms, oedema, gout, haemorrhage, stomachache, syphilis, gonorrhea, urethral diseases, rheumatism, oedema, eye infection & diarrhoea | F | Decoction | Oral |  | Fabaceae |
|  |  |  |  |  | Rb | Relief fever in children | F | Infusion | Oral/Wash |  |  |
|  |  |  |  |  | R | Stomachache, general body aches; inflammation of the urinary tract, cold, chest problems, cough, kidney problems, eye inflammation & pain reliever | F | Decoction | Oral |  |  |
|  |  |  |  |  | SB | Back pain, fever & body pain | F | Infusion | Oral |  |  |
|  |  |  |  |  | R | Acne, tapeworms, urethral problems, oedema, stomachache, inflammation of the urinary tract, pain-killer, ear ache, gout, arthritis, boost urination, throat infection & body pain | F | Decoction | Oral |  |  |
|  |  |  |  |  |  | Antiseptic, coughs, epilepsy & dysentery | F | Infusion | Oral |  |  |
|  |  |  |  |  | L | Gonorrhoea, syphilis, earache, diarrhoea, haemorrhage & dewormer, oedema; inflammation, rheumatism, eye infection and diarrhoea | F | Decoction | Oral |  |  |
|  | *Tarchonanthus camphoratus* L.  (MNCY- 2) | LC | Lelechwet | T | L | Blocked sinuses, asthma, bronchitis, sinus-related complaints & headache | F | Smoke | Inhalation |  | Asteraceae |
|  |  |  |  |  |  | Stomach ailments, asthma, chest ailments, heartburn, over-anxiety, tired legs and sore feet & bronchitis | F | Infusion | Oral |  |  |
|  |  |  |  |  |  | Coughing, toothache, abdominal pain, bronchitis headache, toothache, chest ailments, asthma, cough, abdominal cramps, cold and flu, bronchitis & inflammation | F | Decoction | Oral |  |  |
|  |  |  |  |  |  | Alleviate toothache. | F | Extract | Apply |  |  |
|  |  |  |  |  |  | Chest complaints & body stiffness. | F | Poultice | Massage |  |  |
|  | *Flacourtia indica* (Burm.f.) Merr.  (MNCY- 182) | LC | Lichet | S/ST | R | Indigestion, general stomach pain, hoarseness, pneumonia, intestinal worms, colic, arthritis, headaches, fever, coughs, malaria, asthma & bronchitis | F | Decoction | Oral |  | Salicaceae |
|  |  |  |  |  | L/S | Fevers | F | Juice | Oral |  |  |
|  |  |  |  |  | L | Leave decoction is taken to treat asthma, bronchitis, coughs, dysentery, diarrhea, indigestion and colic. | F | Decoction | Oral |  |  |
|  |  |  |  |  | B | Arthritis, cough, pneumonia, throat infection, fever, diarrhoea, malaria, urinary tract infection & inflammations | F | Decoction | Oral |  |  |
|  |  |  |  |  | L/R | Kidney complaints & enlarged spleen | D | Ash | Leak |  |  |
|  |  |  |  |  | B | Infusion of the bark used as a gargle for oral infections, epilepsy, headache, fever, stomachache, diarrhea and sleep disorders. | F | Infusion | Oral |  |  |
|  |  |  |  |  | G | Cholera, stomachaches, coughs | D | Infusion | Oral | Honey |  |
|  |  |  |  |  | L/R | Snakebites | F | Decoction | Oral |  |  |
|  | *Commelina cyanea*R.Br.  (MNCY- 79) | NE | Loblobityet | H | L/St | Inflammation, irritation or infection in the voice box, sore throats, tonsillitis, nose bleeding, fever, malaria, mumps, gonorrhea, common cold, cough, coughing up blood, influenza, bladder infection & oedema | F | Extract/Juice | Oral |  | Commelinaceae |
|  |  |  |  |  | WP | UTIs, swellings, inflammation, diarrhoea, hemorrhoids, and eye infections, uterine complaints in pregnant women, painful menses, sore throat, malaria, tumours and oedema. | F | Decoction | Oral |  |  |
|  |  |  |  |  | L | Bites from insects, snake & bugs | F | Extract | Topical-Apply |  |  |
|  |  |  |  |  |  | Abscess, boils & burns |  | Poultice | Apply-Dressing |  |  |
|  |  |  |  |  | WP | Bleeding, diarrhoea, fever & diabetes | D | Decoction | Oral |  |  |
|  |  |  |  |  | AP | Stomach discomfort, headache & indigestion | F | Cooked-Vegetable | Oral |  |  |
|  |  |  |  |  | L | Jaundice in infants | F | Infusion | Oral |  |  |
|  | *Psidium guajava* L.  (MNCY- 114) | LC | Maberyat | S/ST | Fr/L | Cholera, diarrhoea, digestive problems, dysentery, inflamed mucous membranes, swollen throat and mouth, skin problems, sore throat, ulcers, vaginal discharge, boost appetite, diarrhea, reduce fever, hypertension, diabetes, caries, pain relief & wounds | F | Decoction | Oral |  | Myrtaceae |
|  |  |  |  |  | L | Wounds & ulcers | F | Extract | Apply |  |  |
|  |  |  |  |  |  | Rheumatic pain, diabetes, hypertension, diarrhoea, stomachaches & indigestion | F | Extract | Oral |  |  |
|  |  |  |  |  | Sh | Relieve toothache | F | Extract | Oral-on tooth |  |  |
|  |  |  |  |  | L/B | Expel the placenta after childbirth, gastrointestinal tract complaints, dysentery & reduce bleeding | F | Decoction | Oral |  |  |
|  |  |  |  |  |  | Skin ailments | F | Decoction | Bath |  |  |
|  |  |  |  |  | B | Gastroenteritis, dysentery, stomach pain, indigestion, dysentery, sore throats, vomiting, stomach upsets, regulate menstrual periods, mouth sores, bleeding gums, as a douche for vaginal discharge, to tighten & tone vaginal walls after childbirth | F | Decoction | Oral |  |  |
|  |  |  |  |  |  | Inflammations of the mouth and throat | F | Decoction | Gargle |  |  |
|  |  |  |  |  | FL | Painful eye conditions such as sun strain, conjunctivitis or eye injuries | F | Extract | Drops |  |  |
|  |  |  |  |  | L | Women reproductive issues, convulsions & uterine wash | F | Decoction | Oral |  |  |
|  |  |  |  |  | FL/Bd | Constipation, cholera, inflammation, rheumatism & epilepsy | F | Decoction | Oral |  |  |
|  |  |  |  |  | R | Chest pain; gastrointestinal, respiratory disturbances & inflammation | F | Decoction | Oral |  |  |
|  | *Ageratum conyzoides* L.  (MNCY- 185) | NE | Mabibiuet | H | R | Chest pain | F | Decoction | Oral |  | Asteraceae |
|  | *Tabernaemontana stapfiana* Britten  (MNCY- 230) | LC | Mabondet | T | B/Se/R | Control high blood pressure, pain relief, fever & amoeba | F | Decoction | Oral |  | Apocynaceae |
|  |  |  |  |  | Lx | Wounds, local pain reliever, antidote for snake bites & removal of warts | F | Latex | Apply |  |  |
|  |  |  |  |  | L | Appetizer, boost lactation, malaria, wounds, sores and abscesses & indigestion. Strong doses- induce abortion | F | Decoction | Oral |  |  |
|  |  |  |  |  | R | Pulmonary disease and chest pains | F | Decoction | Oral |  |  |
|  |  |  |  |  | Se/SB | Heart disease & cancer | F | Decoction | Oral |  |  |
|  | *Helichrysum schimperi* (Sch.Bip. ex A. Rich.) Moeser  (MNCY- 83) | NE | Manarariat | S/L | L/R | Cough remedy | F | Extract | Oral | Ghee | Asteraceae |
|  |  |  |  |  | R | Stomachache, diarrhea & gonorrhoea | F | Decoction | Oral |  |  |
|  |  |  |  |  | L | Colic, stomachache discomfort & malaria | F | Decoction | Oral |  |  |
|  |  |  |  |  | St | Wounds | D | Ash | Apply |  |  |
|  |  |  |  |  | Sh | Wounds & sniffed to treat cold | F | Extract | Apply/Snuff |  |  |
|  | *Zehneria scabra* Sond. (MNCY- 12) | NE | Manarariat | S/L | WP | Burns | D | Ash | Apply |  | Asteraceae |
|  |  |  |  |  | L | Skin rashes | F | Pounded-Extract | Wash |  |  |
|  |  |  |  |  | R | Malaria, anaemia, STDs, skin diseases, syphilis, gonorrhea, abdominal pain, expel worms & malaria | F | Decoction | Oral |  |  |
|  |  |  |  |  | L | Diarrhoea | F | Infusion | Oral |  |  |
|  |  |  |  |  |  | Malaria | F | Decoction | Oral | Other plants |  |
|  |  |  |  |  |  | Fever, paralysis & headache | F | Juice | Oral |  |  |
|  |  |  |  |  |  | Wound dressing and healing & swelling | F | Paste | Apply/Tied |  |  |
|  |  |  |  |  | WP | Discomfort, illness, or unease whose exact cause is difficult to identify & fever | F | Steam | Inhalation |  |  |
|  |  |  |  |  | R | Constipation and mixed with other plant extracts for dog bites/rabies | F | Infusion | Oral | Other plants |  |
|  | *Gerrardanthus lobatus*(Cogn.) C.Jeffrey  (MNCY- 220) | NE | Manareriat | S/L | L | Sores on the tongue | F | Extract | Oral |  | Cucurbitaceae |
|  |  |  |  |  | WP | Diabetes, enlarged glands & malaria | F | Juice | Oral |  |  |
|  |  |  |  |  | L/St | Abrupt contractions, excessive discharge or build-up of mucus in the nose or throat, inflammation of the mucous membrane & expectorant | F | Decoction | Oral |  |  |
|  |  |  |  |  | L | Sores and other skin diseases | F | Paste | Apply | Ghee |  |
|  |  |  |  |  | R | Malaria | F | Decoction | Oral |  |  |
|  |  |  |  |  | R | Gonorrhoea and STDs | F | Maceration-Extract | Oral |  |  |
|  | *Phragmanthera usuiensis* (Oliver) M.G.Gilbert (MNCY- 220) | NE | Mandawet | E | WP | Paralysis, stroke, gout, stomaches, migraine, fever & cancer | F | Decoction | Oral |  | Cucurbitaceae |
|  |  |  |  |  | L | Leave infusion is taken to treat joint pain and arthritis. | F | Infusion | Oral |  |  |
|  |  |  |  |  | WP | Indigestion, constipation & stomach discomfort | D | Ash | Leaked |  |  |
|  | *Zehneria minutiflora* (Cogn.) C.Jeffrey  (MNCY- 213) | NE | Manereriat (Kimanererit) | S/L | L | Gonorrhoea | F/D | Decoction | Oral |  | Cucurbitaceae |
|  |  |  |  |  | R | Pneumonia, toothache & eye infections | F | Decoction | Oral |  |  |
|  |  |  |  |  | R/L | Malaria, intestinal worms, inflammation of the ear and with pus in some cases, rheumatism, bowel evacuation, purification or purgation that brings about spiritual renewal or release from tension, diarrhea, dysentery & cholera. | F | Decoction | Oral |  |  |
|  |  |  |  |  | L | Leave infusion is to manage bad odours/breath and as a bath to manage odours. | F | Infusion | Oral |  |  |
|  |  |  |  |  | WP | Uterine fibroids, intestinal pain, venereal diseases (syphilis, gonorrhoea); skin diseases: fungal infections, prevention of miscarriages, threatened abortion, care after childbirth, gonorrhea and constipation in enema. | F | Infusion | Oral |  |  |
|  |  |  |  |  | L | Itching, sprains, wounds, abscess, measles, tiny blisters that eventually burst and leave small wet patches of red skin that may weep fluid & snakebite | F | Extract | Apply/  Massage |  |  |
|  |  |  |  |  |  | Migraine, fevers & headaches | F | Extract | Snuff |  |  |
|  |  |  |  |  |  | Diarrhoea, venereal diseases, parasite infestation, care during and after pregnancy, promote lactation, urogenital infections, tetanus, diarrhoea, dysentery, malaria, mumps, conjunctivitis, oedema, snakebites & eye infections | F | Extract | Oral |  |  |
|  | *Richardia scabra* L. (MNCY- 39) | NE | Manereriat (Kimanererit) | S/L | R | Stimulating the gastric and bronchial systems, lowering fevers, induce vomiting & preventing cyst formation in amoebic dysentery | F | Decoction |  |  | Rubiaceae |
|  |  |  |  |  | L | Ringworms; cancer & boils | F | Extract/Paste | Apply |  |  |
|  |  |  |  |  |  | Skin diseases, wound healing, induce sweating, UTIs, asthma, induce vomiting & stomachaches | F | Extract | Oral |  |  |
|  |  |  |  |  | WP | Restore body strength, asthma & skin infections | F | Decoction | Oral |  |  |
|  |  |  |  |  | Sh | Abdomen pain in urinary tract infection | F | Paste | Apply |  |  |
|  | *Cassipourea malosana* (Baker) Alston  (MNCY- 103) | LC | Martit | T | B | Evacuate a retained placenta after birth & induce abortion | F | Infusion | Oral |  | Rhizophoraceae |
|  |  |  |  |  |  | Skin ailments & pimples | F | Decoction | Oral |  |  |
|  |  |  |  |  | Rb | Skin diseases, dispel bad dreams & protection from evil spirits | F | Infusion | Oral |  |  |
|  |  |  |  |  | B | Burns | F | Poultice | Apply |  |  |
|  | *Croton megalocarpus Hutch.*  (MNCY- 208) | LC | Masineitet | T | B | Fresh wounds or cuts, | F | Decoction | Apply |  | Euphorbiaceae |
|  |  |  |  |  |  | Malaria, intestinal worms, hypertension, diabetes, cancer, digestive problems, gastric inflammation, fever, pain, ulcers, dysentery, weight-loss, haemorrhoids, constipation & heart issue related to cholesterol | F | Decoction | Oral |  |  |
|  |  |  |  |  | SB/Rb | Skin rashes | F | Decoction | Bath |  |  |
|  |  |  |  |  | L | Coughs | D | Ash | Leak |  |  |
|  |  |  |  |  |  | Blood clotting | F | Juice | Oral |  |  |
|  |  |  |  |  | Se | Blood purification | F | Decoction | Oral |  |  |
|  |  |  |  |  |  | Skin infections or muscle aches or pain | F | Paste-Poultice | Apply/  Massage |  |  |
|  |  |  |  |  | B/R | Malaria, induce abortion, expel worms, whooping cough, pneumonia, stomach-aches, fevers such as malaria, & abdominal complaints associated with gall bladder and spleen. | F | Decoction | Oral |  |  |
|  |  |  |  |  | Se/B/R | Induce vomiting & treat gonorrhoea | F | Decoction | Oral |  |  |
|  |  |  |  |  | L/R | Headaches; gonorrhoea, cough, malaria, stomach problems, hypertension, asthma, colic & fever | F | Decoction | Oral |  |  |
|  |  |  |  |  | Tw | Insect and lice repellent | F | Smoke | Scent |  |  |
|  |  |  |  |  | Rb | Female infertility |  | Infusion | Oral |  |  |
|  | *Plantago palmata* Hook.f.  (MNCY- 224) | NE | Masiririet | H/S | L | Wounds, liver issues, expulsion of worm; pregnancy and lactation troubles, improvement of health after disease. coughs, constipation, colic & general pains | F | Decoction | Oral |  | Plantaginaceae |
|  |  |  |  |  |  | Hepatic diseases and the root | F | Extract | Oral |  |  |
|  |  |  |  |  | R | Colic | F | Decoction | Oral |  |  |
|  | *Amaranthus graecizans* L.  (MNCY- 93) | NE | Mbogiat | H | L | Tonsillitis, reduce bleeding of minor cuts & ulcerated mouths and throats | F | Extract | Oral |  | Amaranthaceae |
|  |  |  |  |  |  | Boil to facilitate the creation of the head | F | Paste | Spray |  |  |
|  |  |  |  |  |  | Ulcers, warts and sores | F | Extract | Wash |  |  |
|  |  |  |  |  | R | Controls vomiting | F | Paste | Oral | Honey |  |
|  |  |  |  |  |  | Dysentery | F | Paste | Oral | Sugar and water |  |
|  |  |  |  |  |  | Gonorrhea | F | Decoction | Oral |  |  |
|  |  |  |  |  | L/R | Skin diseases/ disorders such as abscesses, bruises, burn, eczema, inflammation, gonorrhea, heavy menstrual bleeding and wound | F | Smooth paste | Apply-Poultice |  |  |
|  |  |  |  |  | WP | Internal bleeding, diarrhea, excessive menstruation, snake bites, boils, stomach disorders, ulcerated mouths, vaginal discharges, nosebleeds & wounds | F | Decoction | Oral |  |  |
|  |  |  |  |  | R | The juice of the roots is used externally to relieve headaches. | F | Juice | Apply |  |  |
|  |  |  |  |  |  | Menstrual periods with abnormally heavy or prolonged bleeding, gonorrhoea, eczema & colic | F | Paste | Oral |  |  |
|  |  |  |  |  |  | Fevers, urinary troubles, diarrhea & dysentery | F | Juice | Oral |  |  |
|  |  |  |  |  | St | Eye infections & convulsions |  | Sap | Wash |  |  |
|  | *Physalis peruviana* L*.*  (MNCY- 175) | LC | Mboniot | S | L | Treatment of worms and bowel complaints | F | Juice | Oral |  | Solanaceae |
|  |  |  |  |  | FL | Diabetes, kidney and bladder stones, fluid retention, gout and urinary tract disorders; cancer, malaria, asthma, hepatitis, dermatitis & rheumatism | F | Decoction | Oral |  |  |
|  |  |  |  |  | WP | Rash and ringworms | F | Juice | Apply |  |  |
|  |  |  |  |  | FL | Extract from the flower is applied on wounds | F | Extract | Apply |  |  |
|  |  |  |  |  | L | The leaves are heated over the fire and rubbed on swollen body parts, on boils, cuts and scratches. | F | Heated leaves | Rub |  |  |
|  |  |  |  |  |  | Asthma, intestinal worms, enlarged spleen, inducing labour, ease childbirth, abdominal disorder during pregnancy, jaundice, malaria, diabetes, malaria & pneumonia | F | Decoction | Oral |  |  |
|  |  |  |  |  |  | Inflammations | F | Poultice | Apply |  |  |
|  |  |  |  |  | L/St | Swelling of legs and feet for pregnant women | F | Extract | Oral |  |  |
|  |  |  |  |  | Fr | Sore throat, gastro-intestinal disorders & typhoid fever | F | Pulp | Oral |  |  |
|  |  |  |  |  |  | Eye infection & cataract; Ear pain | F | Juice | Drops |  |  |
|  |  |  |  |  |  | Diabetes | F | Juice | Oral |  |  |
|  |  |  |  |  | AP | Cancer and cancer like symptoms, diabetes, colic in children, spleen & malaria | F | Decoction | Oral |  |  |
|  |  |  |  |  | L | Typhoid |  | Infusion | oral |  |  |
|  | *Amaranthus dubuis* Mart.  (MNCY- 218) | NE | Mborochet | H | L | Fever, haemorrhage, anaemia, constipation, kidney complaints asthma, cancer, kidney and liver disorders & malaria | F | Decoction | Oral |  | Amaranthaceae |
|  |  |  |  |  | R | Controls vomiting & dysentery | F | Paste | Oral | Honey |  |
|  |  |  |  |  | L/R | Skin diseases/ disorders such as abscesses, bruises, burn, eczema, inflammation & wound | F | Extract | Apply |  |  |
|  |  |  |  |  | WP | Stomachache, internal bleeding, diarrhea, excessive menstruation, snake bites, boils, stomach disorders, ulcerated mouths, vaginal discharges, nosebleeds & wounds | F | Decoction | Oral |  |  |
|  | *Tiliacora kenyensis Troupin*  (MNCY- 88) | EN | Mborosiat | C | L | Leaf paste applied on boils to promote head formation | F | Paste | Appy |  | Menispermaceae |
|  |  |  |  |  | B | Stomach upset, chest pain, treating malaria, fever, worm evacuation & detoxification | D | Extract | Oral |  |  |
|  |  |  |  |  | L | Juice from macerated leaves is applied to cuts. | F | Juice | Apply |  |  |
|  |  |  |  |  | L/R | Bites | F | Paste | Appy |  |  |
|  |  |  |  |  | R | Snake bites & cancerous diseases | F | Decoction | Oral |  |  |
|  |  |  |  |  | L | Dysentery | F | Juice | Oral | Salt |  |
|  |  |  |  |  |  | Bronchial asthma, diarrhoea & relieve pain | F | Decoction | Oral |  |  |
|  | *Indigofera arrecta* Hochst. ex A.Rich*.*  (MNCY- 130) | NE | Menjeiwet | H/SS | L/R | Induce abortion, treat symptoms of abdominal pain, including spasms and cramps, promote passing of urine in men with enlarged prostate, reduce fever, evacuate the bowel, promote sleep, boost appetite &digestion | F | Infusion/Decoction | Oral |  | Fabaceae |
|  |  |  |  |  | Sh | Aching tooth with cavity & inflammation in the gums | F | Paste | Oral-on tooth |  |  |
|  |  |  |  |  | WP | Gastric disorders, headaches, chest pain, promotes passage of urine in men, liver and spleen enlargement, UTIs, skin infections, urinary stones & relieve cough | F | Decoction | Oral |  |  |
|  |  |  |  |  | FL | Kidney stones, diarrhea & dysentery | F | Extract | Oral |  |  |
|  |  |  |  |  | L | Epilepsy, nervous disorders; sores, ulcers; treat skin diseases, swellings & wounds | F | Extract | Oral |  |  |
|  |  |  |  |  | R | Gum infections, snakebites, gonorrhea, epilepsy & jaundice | F | Decoction | Oral |  |  |
|  |  |  |  |  | Tw | Oral care | F | Extract | Oral |  |  |
|  |  |  |  |  | L/R | Itching | F | Paste | Apply |  |  |
|  |  |  |  |  | Sh | Diabetes management | F | Extract | Oral |  |  |
|  |  |  |  |  | Fr/Se | Eye infections | F | Extract | Drops |  |  |
|  | *Triumfetta brachyceras*K.Schum.  (MNCY- 29) | NE | Meswot | S | R | Internal ulcerations | F | Decoction | Oral |  | Malvaceae |
|  |  |  |  |  | L | Anaemia | D | Powder-Infusion | Oral |  |  |
|  |  |  |  |  | R | Aphrodisiac; restore body strength, appetizer, cancer tumours, dysentery, internal bleeding & gonorrhoea | F | Decoction | Oral |  |  |
|  |  |  |  |  | Rb | Cancerous wounds “Seriat”. | D | Powder | Apply |  |  |
|  |  |  |  |  | L | General body weakness, dysentery, diarrhoea & intestinal ulcers | F | Extract | Oral |  |  |
|  |  |  |  |  |  | Dental caries | F | Heated-leaves | Rub |  |  |
|  |  |  |  |  | FL | Boils | F | Poultice | Apply |  |  |
|  |  |  |  |  | R/L | Ease childbirth, expel the placenta & sterility in women | F | Sap | Oral | Water |  |
|  |  |  |  |  | L | Reduce bleeding in minor cuts; sooth and moisturize the skin | F | Paste | Apply |  |  |
|  | *Hibiscus diversifolius* Jacq.  (MNCY- 237) | NE | Meswot | S | L | Insect stings, pain. | F | Extract | Apply |  | Malvaceae |
|  |  |  |  |  | B | Malaria | F | Decoction | Oral |  |  |
|  |  |  |  |  | L | Control vomiting | F | Extract | Oral |  |  |
|  |  |  |  |  | R/FL | Pneumonia | F | Decoction | Oral |  |  |
|  |  |  |  |  | L | Bronchitis & chronic coughing especially to children | F | Decoction | Oral |  |  |
|  |  |  |  |  | R | Easing labour pains by relaxing the muscles, heart & nerve diseases | F | Decoction | Oral |  |  |
|  |  |  |  |  | FL | Boost urination; stimulate bile production, fevers, blood pressure; hangover remedy to reduce the rate of alcohol absorption & fluid urination | F | Teas-infusion | Oral |  |  |
|  |  |  |  |  | Se | Induce lactation, indigestion, relieve painful urination, general body strength, cough remedy, cracks in the feet, sores & wounds | D | Decoction | Oral |  |  |
|  |  |  |  |  | FL | Stimulate menstruation, colds, hypertension, loss of appetite, heart and nerve diseases; upper respiratory tract pain and swelling; fluid retention, stomach irritation, disorders of circulation; dissolving the thick, sticky stuff that hangs around in the back of the throat when one is sick; gentle laxative & increase urine output | F | Juice | Oral |  |  |
|  |  |  |  |  | FL | Relieve coughs | F | Infusion | Oral |  |  |
|  | *Rumex abyssinicus* Jacq.  (MNCY- 10) | NE | Mindeywet | H | R | Colic, malaria, gonorrhea, antidote for suspected poisoning, liver problems, constipation, pain that runs down one or both legs from the lower back, blood pressure, migraine, rheumatism, breast cancer, stomach discomfort, earache, hemorrhoids, diabetes, oedema, skin disorders, inflammation, relieve pain & wounds. | F | Decoction | Oral |  | Polygonaceae |
|  |  |  |  |  | L | Coughs, colds & chest infections | D | Ash | Leak |  |  |
|  |  |  |  |  |  | Joints and areas of rheumatism | F | Paste | Massage-Apply |  |  |
|  |  |  |  |  | Rh | Wounds | F | Poultice | Apply |  |  |
|  |  |  |  |  |  | Diabetes, laxative, dewormer detoxification & haemorrhage | F | Extract | Oral |  |  |
|  |  |  |  |  | Sh/L | Reduce bleeding on minor cuts | F | Extract | Apply |  |  |
|  |  |  |  |  | St | Jaundice and related liver diseases, stomach-ache, neck ache & low blood pressure | F | Extract | Oral |  |  |
|  |  |  |  |  | L | Gonorrhea and abscesses | F | Decoction | Oral |  |  |
|  |  |  |  |  | Tu | Oral thrush, wounds, sores & parts affected by scabies | D | Powder | Apply |  |  |
|  | *Markhamia lutea* (Benth.) K. Schum  (MNCY- 90) | LC | Mobet | ST/T | L/B | Toothache | F | Extract | Gargle-Oral |  | Bignoniaceae |
|  |  |  |  |  |  | Stomachache, headache & convulsion in children | F | Extract | Oral |  |  |
|  |  |  |  |  | L | Snakebites | F | Extract | Apply |  |  |
|  |  |  |  |  | Sh | Throat complaints, lower back pain cough, malaria & diarrhea | F | Extract | Oral |  |  |
|  |  |  |  |  | R | Convulsions in children | F | Decoction | Oral |  |  |
|  |  |  |  |  |  | Earache | F | Decoction | Drops |  |  |
|  |  |  |  |  | B | Aphrodisiac | F | Decoction | Oral |  |  |
|  |  |  |  |  | R | Reduce symptoms of watery bloodless diarrhea, urination, relief pain, syphilis wounds, relieve rheumatic pain & respiratory tract infections | F | Infusion | Oral |  |  |
|  |  |  |  |  | Rb | Anemia, diarrhea & backache | F | Decoction | Oral |  |  |
|  |  |  |  |  | B/R | Restore body strength, promote urination, oedema of the legs, swollen scrotum, diarrhea, dysentery, pain, inflammation, anaemia, liver disease, boost appetite, stomachache, asthma, cough, gonorrhoea, headache, skin rash, eye infection, throat diseases, snakebites & cancer management | F | Decoction | Oral |  |  |
|  |  |  |  |  | R | Venereal diseases, anemia, diarrhea, backache, relieve pain on the lower back, pulmonary troubles, gout, swollen scrotum, arthritis, external skin diseases and for preventing abortion. | F | Decoction | Oral |  |  |
|  |  |  |  |  | L/B | Skin lotion, skin-affections, sores & itches | F | Lotion  Paste- | Apply/Rub |  |  |
|  |  |  |  |  | R | Sore eyes and earaches | F | Decoction | Drops |  |  |
|  | ** *Ficus sur*Forssk.  (MNCY- 108) | NE | Mogoiwet | T | R | Cough, sore throat, diarrhoea, stomach pain in babies, chest pain, infertility, uterine pain, gonorrhoea & oedema | F | Decoction | Oral |  | Moraceae |
|  |  |  |  |  | L | Peptic ulcer & chest problems | F | Extract | Oral |  |  |
|  |  |  |  |  | B | Skin rashes & mouth sores | D | Powder | Apply |  |  |
|  |  |  |  |  |  | Fever and cough | F | Extract | Oral |  |  |
|  |  |  |  |  |  | Pain, rheumatism, diarrhoea, stomach problems, infertility &swelling in children | F | Decoction/Infusion | Oral |  |  |
|  |  |  |  |  | St | Wounds, toothache, eye problems, general body pain, lung and throat problems, threatened abortion, gonorrhoea & to induce vomiting | F | Latex | Oral |  |  |
|  |  |  |  |  | B | Relieve fever in infants and strengthening the bones | F | Decoction | Bath |  |  |
|  |  |  |  |  | L | Fertility in men, dysentery, treat infertility, tuberculosis, abscesses and sores, boost lactation, clear the bowel, oedema, epilepsy, gonorrhoea, respiratory disorders, gastrointestinal tract & boost general body health | F | Decoction | Oral |  |  |
|  |  |  |  |  | Sh | Aching tooth & relieving the pain | F | Sap | Apply |  |  |
|  |  |  |  |  | B | Stomach upsets | F | Infusion | Oral |  |  |
|  | *Protea gaguedi* J.F.Gmel.  (MNCY- 64) | LC | Mogondwet | S | L/R | Diarrhoea | F | Decoction | Oral |  | Proteaceae |
|  |  |  |  |  | L | Wounds | F | Paste | Apply |  |  |
|  |  |  |  |  | R | Ear infections, broken bones, aphrodisiac, back pains, ear infection, stroke, diarrhoea, wound healing & skin diseases | F | Decoction | Oral |  |  |
|  |  |  |  |  | FL | Cough and other chest ailments | F | Decoction | Oral |  |  |
|  |  |  |  |  | R/B | Internal bleeding, stomach ulcers, diarrhoea & dizziness | F | Infusion | Oral |  |  |
|  |  |  |  |  | B | The bark is chewed to relieve coughs | F | Extract | Oral |  |  |
|  | *Landolphia buchananii Stapf*  (MNCY- 207) | NE | Molongwet | S/C | R | Gonorrhea | F | Decoction | Oral |  | Apocynaceae |
|  |  |  |  |  | L | Gonorrhoea wounds | F | Infusion | Apply |  |  |
|  |  |  |  |  | St/R | Stomach inflammation, gastric ulcers & stomach cramps | F | Decoction | Oral |  |  |
|  |  |  |  |  | R | Pain relief, aphrodisiac, general body strength & haemorrhoids | F | Extract | Oral |  |  |
|  |  |  |  |  | St | Different eye infections | F | Latex | Drops |  |  |
|  |  |  |  |  | SB | Dewormer. | F | Paste | Oral |  |  |
|  | *Rubus steudneri* Scweinf.  (MNCY- 16) | NE | Momoniat | S | R | Indigestion and abdominal pains. | F | Decoction | Oral |  | Rosaceae |
|  |  |  |  |  |  | Coughs & colds in children | F | Infusion | Oral |  |  |
|  |  |  |  |  |  | Indigestion, diabetes, inflammation of the stomach, diarrhoea, rheumatism, stomachache, diarrhea & cough | F | Decoction | Oral |  |  |
|  |  |  |  |  | L | Relieve pain, treat arthritis &swollen feet | F | Paste | Massage |  |  |
|  | *Rubus pinnatu*s Willd.  (MNCY- 231) | NE | Momoniat | S | R | Coughs & colds in children | F | Extract | Oral |  | Rosaceae |
|  |  |  |  |  |  | Indigestion, abdominal pains, dysentery, diabetes, inflammation of the stomach, diarrhoea, rheumatism, stomachache, cough & reduce bleeding on minor cuts | F | Decoction | Oral |  |  |
|  |  |  |  |  | L | Joints to relieve pain, treat arthritis & swollen feet | F | Paste | Massage |  |  |
|  | *Gymnanthemum amygdalinum*(Delile) Sch.Bip. ex Walp.  (MNCY- 95) | NE | Mororwet | S/ST | R/L | Epilepsy, nausea, diabetes, loss of appetite, dysentery and other gastrointestinal tract problems, STDs & diabetes | F | Decoction | Oral |  | Asteraceae |
|  |  |  |  |  | R | Stomach ache, vaginal itching, bowel evacuation and to boost appetite and digestion | F | Decoction | Oral |  |  |
|  |  |  |  |  | L | Diabetes, malaria, fever, constipation, high blood pressure and to evacuate bowels, induction of uterine mobility & control of post-parturn hemorrhage | F | Decoction | Oral |  |  |
|  |  |  |  |  |  | Breast milk enhancement. | D | Powder | Oral |  |  |
|  |  |  |  |  |  | Leaves infusion is used to treat convulsion, stomachache, malaria, menstruation pain, wound dressing, urinary tract inflammation, STDs, worms, wound healing, hypertension, boils & burns | F | Infusion | Oral |  |  |
|  |  |  |  |  |  | Fever | F | Juice | Oral |  |  |
|  |  |  |  |  |  | Coughs & colds | D | Ash | Leak |  |  |
|  |  |  |  |  | R | Intestinal worms | F | Infusion | Oral |  |  |
|  | *Sorghum bicolor* (L.) *Moench*  (MNCY- 23) | LC | Mosong'iot | H | L/FL | Anaemia | F | Decoction | Oral |  | Poaceae |
|  |  |  |  |  | Tw | Jaundice, cancer & alleviate chronic pain | F | Decoction | Oral |  |  |
|  |  |  |  |  | FL | Dry up mucus secretions, treatment of kidney & urinary complaints | F | Extract | Oral |  |  |
|  |  |  |  |  | Se | Relieve irritation on the mouth, soothe membranes, relieve minor pain, inflammation of the membrane & promote urination. | F | Decoction | Oral |  |  |
|  |  |  |  |  | WP | Restore general body health | F | Decoction | Oral |  |  |
|  |  |  |  |  | Se | Liver problems & detoxify | F | Infusion | Oral |  |  |
|  |  |  |  |  | Tw | Jaundice | F | Decoction | Oral |  |  |
|  | *Ocimum tenuiflorum* L.  (MNCY- 42) | NE | Mosoriot | S | L/St | Fever in children, colds, influenza, sinusitis, headaches, rheumatism, arthritis, digestive disorders, indigestion, cramps; low libido & diabetes | F | Decoction | Oral |  | Lamiaceae |
|  |  |  |  |  | AP | Expectorant especially in cases where fever is involved | F | Teas-Infusion | Oral | Honey |  |
|  |  |  |  |  | L | Cold and flu | F | Extract | Oral |  |  |
|  |  |  |  |  | WP | Nausea and vomiting, cough, asthma, diarrhea, fever, dysentery, arthritis, eye diseases, indigestion, gastric ailments retaining skin moisture levels; reduces skin roughness, prevents wrinkles & makes the skin smooth | F | Decoction | Oral |  |  |
|  |  |  |  |  | L/Se | Malaria, stomach ulcers & eye diseases | F | Decoction | Oral |  |  |
|  |  |  |  |  | R | Peptic ulcers, bronchitis, indigestion, malaria, stomach ulcers, eye diseases, insect bites, stress relief, eczema, anxiety, stress, managing diabetes, managing high cholesterol & promotes longevity of life | F | Decoction | Oral |  |  |
|  |  |  |  |  | FL | Bronchitis, chest pain, coughs & colds | F | Extract | Oral |  |  |
|  |  |  |  |  | L | Insect bites, antiseptic to skin infections, spots, ringworm & earaches | F | Juice | Apply/Drops |  |  |
|  | *Lagenaria abyssinica* (Hook.f) C.Jeffrey (MNCY- 221) | NE | Motondorwet | H/C | L | Gonorrhea, TB & scabies | F | Decoction | Oral |  | Cucurbitaceae |
|  |  |  |  |  | Fr | Boost memory, epilepsy & other nervous diseases | F | Juice | Oral | Honey |  |
|  |  |  |  |  | Se | Boils, toothache & gums | D | Poultice | Apply | Salt/Ghee |  |
|  |  |  |  |  | L | Skin irritation, hair loss & tumors | F | Paste/ Poultice | Apply |  |  |
|  |  |  |  |  |  | Leaf juice is widely used for baldness. | F | Juice | Apply |  |  |
|  |  |  |  |  | Fr | Induce vomiting, promote sleep, evacuate the bowel, cooling & promote urination | F | Pulp | Oral |  |  |
|  |  |  |  |  |  | Jaundice, diabetes, ulcer, haemorrhoids & colitis | F | Decoction | Oral |  |  |
|  |  |  |  |  | R | Relief stomachache | F | Decoction | Oral |  |  |
|  | *Hibiscus calyphyllus* Cav.  (MNCY- 203) | NE | Motosyet | S | L | sores and wounds | F | Extract | Apply |  | Malvaceae |
|  |  |  |  |  | FL | Epilepsy, diabetes, fertility & blood pressure | F | Decoction | Oral |  |  |
|  |  |  |  |  | R | Heavy menstrual bleeding | D | Powder-Infusion | Oral |  |  |
|  |  |  |  |  |  | Gonorrhea | F | Juice | Oral |  |  |
|  |  |  |  |  | AP | Constipation, fever & diarrhea | F | Decoction | Oral |  |  |
|  |  |  |  |  | L/FL | Promote hair growth, heal ulcerations & fatigue | F | Paste | Apply/  Massage |  |  |
|  |  |  |  |  | Sh | Wounds | F | Paste | Apply | Saliva |  |
|  |  |  |  |  | WP | Coughs, colds, chest pain, bronchitis & pneumonia | F | Steam | Inhalation |  |  |
|  |  |  |  |  | L | Wounds | F | Paste-Dressing | Apply |  |  |
|  | *Hydrocotyle hirsuta var.* hirsute  (MNCY- 3) | NE | Mungutab beliot ne chabai | H | WP | Relief headache, abdominal pain, fever, bowel complaints, rheumatism, tonsillitis, fluid around the lungs, liver disease, stomach pain, diarrhea, indigestion, stomach ulcers, swelling in the lining of the stomach, epilepsy, asthma, anemia & general body strength. | F | Decoction | Oral |  | Araliaceae |
|  |  |  |  |  | L | Syphilitic ulcers & wound healing | F | Paste | Poultice-Apply |  |  |
|  |  |  |  |  | WP | Absence of menstrual periods, arouse sexual desire, cholera, dysentery, syphilis, common cold, flu, TB, circulation problems including varicose veins, prevent blood clots in the legs; prevent damage of small blood vessels in people with diabetes. | F | Extract | Oral |  |  |
|  | *Centella asiatica* (L.) Urb.  (MNCY- 154) | LC | Mungutab beliot ne sing’ortot | H  creeper | WP | Headache, peptic ulcers, stimulant, heart related conditions, syphilis, blood purifier, blood pressure, depression & diabetes | F | Decoction | Oral |  | Apiaceae |
|  |  |  |  |  | L | Inflammation of the breasts, healing wounds & burns | F | Paste | Apply |  |  |
|  | ***Olea capensis*L.** (MNCY- 43) | LC | Murguiwet | T | B | Reduce blood sugar, gout, diabetes, hypertension, inflammation, diarrhea, urinary tract infections, and respiratory infections like asthma, stomach; intestinal diseases, hemorrhoids, malaria, rheumatism, mouth cleanser & boost blood circulation | F | Decoction | Oral |  | Oleaceae |
|  |  |  |  |  |  | Eye infections | D | Powder-Extract | Drops |  |  |
|  |  |  |  |  |  | Headache, diarrhoea, fever & UTIs | F | Extract | Oral |  |  |
|  | *Albizia coriaria* Welw. ex Oliv.  (MNCY- 18) | LC | Musengertet | T | B | Dysentery, bronchial problems & pain caused by fever | F | Decoction | Oral |  | Fabaceae |
|  |  |  |  |  |  | Liver issues | F | Extract | Oral |  |  |
|  |  |  |  |  |  | Sores, pimples & other skin complaints | F | Decoction | Apply |  |  |
|  |  |  |  |  | L | Induce abortion. | F | Enema | Apply |  |  |
|  |  |  |  |  |  | Headache & kill body parasites | F | Decoction | Wash |  |  |
|  |  |  |  |  |  | Fever, toothache & malaria | F | Steam | Inhalation |  |  |
|  |  |  |  |  | SB/L | Cough, sore throats, syphilis, skin diseases, general body strength & eye diseases | F | Decoction | Oral |  |  |
|  |  |  |  |  | B | Insect bites, skin infections, boils, abscesses, ulcers, tied up on fractures & sprains | F | Paste | Apply/Tied |  |  |
|  |  |  |  |  |  | Anxiety, cancer, depression, sleep problems, sore throat, to improve mood & reduce swelling associated with trauma | F | Infusions |  |  |  |
|  | *Olinia rochetiana* A.Juss.  (MNCY- 151) | NE | Museset | S/ST | R | Fever, intestinal worms (as antihelminthic), rheumatic pain, respiratory; indigestion problems, headaches, backaches, scabies & psychoses | F | Decoction | Oral |  | Penaeaceae |
|  |  |  |  |  |  | Malaria | F | Extract | Oral |  |  |
|  |  |  |  |  | B | Deworm tapeworms, rheumatism, bronchitis & indigestion | F | Decoction | Oral |  |  |
|  |  |  |  |  |  | Babies with coughs | F | Decoction | Oral | Milk |  |
|  |  |  |  |  |  | Coughs | F | Chew-Extract | Oral |  |  |
|  |  |  |  |  | Sh | Stimulant | F | Chew-Extract | Oral |  |  |
|  | *Ehretia cymosa* Thonn.  (MNCY- 7) | LC | Mutereriet | S/ST | B | amenorrhea, mitigation of jaundice, asthma, dysentery, ulcers, diarrhea, ringworm, eczema, diabetes, syphilis, cuts, wounds, inflammation, liver problems, regulate menstrual cycle, venereal & infectious disorders | F | Decoction | Oral |  | Boraginaceae |
|  |  |  |  |  | R | skin infections as a cream | F | Decoction-Oil | Apply |  |  |
|  |  |  |  |  | R/L | Aphrodisiac  Caution should be taken because of can be also toxic | F | Decoction | Oral |  |  |
|  |  |  |  |  | L | Mild laxative for children | F | Sap | Oral |  |  |
|  |  |  |  |  | L | Fractured bones to promote healing | F | Poultice | Tied |  |  |
|  |  |  |  |  | R | Epilepsy | F | Decoction | Oral |  |  |
|  |  |  |  |  | L | Healing wounds | F | Juice | Apply |  |  |
|  |  |  |  |  |  | Epilepsy | D | Ash | Leak |  |  |
|  |  |  |  |  | L | Insect bites | F | Extract | Apply |  |  |
|  |  |  |  |  | R/L | Tetanus, brucellosis & dysentery | F | Decoction | Oral |  |  |
|  |  |  |  |  | R | The root juice is applied to wounds | F | Juice | Apply |  |  |
|  |  |  |  |  | R | Stomach complaints, venereal diseases, epilepsy, dry cough, tonsillitis, typhoid, malaria, asthma, wounds &s an aphrodisiac | F | Extract | Apply |  |  |
|  |  |  |  |  | L | The leaves infusion is used as a wash to treat fevers, | F | Infusion | Wash |  |  |
|  |  |  |  |  |  | Convulsions in children, relieve pain, paralysis, epilepsy, spasm, toothache & as a laxative | F | Infusion | oral |  |  |
|  | *Azadirachta indica* A.Juss.  (MNCY- 150) | LC | Mwarubaini | T | B | Malaria | F | Decoction | Oral |  | Meliaceae |
|  |  |  |  |  | L | Boost appetite, purify the blood & diabetes | F | Decoction |  |  |  |
|  |  |  |  |  |  | Reduce pain, discomfort, arthritis, healing of eczema, ringworm, wounds, ulcers & acne | F | Extract | Apply |  |  |
|  |  |  |  |  |  | Detoxify the body, kidney problems, liver issues, reduce inflammation of the gastrointestinal tract which helps reduce a series of diseases like constipation, stomach ulcer, flatulence, mouthwash & boost the immune system | D | Poeder-Infusion | Oral/Gargle |  |  |
|  |  |  |  |  |  | Acne, rashes & skin diseases | F | Extract | Apply |  |  |
|  |  |  |  |  | Fr | Boils, syphilitic sores, skin diseases, glandular swellings, peptic ulcers, bowel evacuation, intestinal worm infestation, urinary diseases & jaundice | F | Juice |  |  |  |
|  |  |  |  |  | FL | Restore general body strength, treat constipation & indigestion | D | Infusion | Oral |  |  |
|  |  |  |  |  |  | Chronic eczema | D |  | Apply |  |  |
|  |  |  |  |  |  | Fly repellant | D | Smoke | Scent |  |  |
|  |  |  |  |  | B | A weak infusion of the bark is drunk to relieve fever. | F | Infusion |  |  |  |
|  | *Lippia kituiensis* Vatke  (MNCY- 61) | NE | Mwokiot | S | L | Chronic joint pains, arthritis, to relieve flu respiratory disorders, bronchitis, asthma, colds & coughs | F | Decoction | Oral |  | Verbenaceae |
|  |  |  |  |  | R | Abdominal pain & malaria | F | Decoction | Oral |  |  |
|  |  |  |  |  |  | Sedative, relaxing remedy, antifertility, diabetes & remedy for menstrual disorders | F | Infusion | Oral |  |  |
|  |  |  |  |  | L | Soothe aches, applied externally to treat skin diseases, burns, wounds, ulcers, treat syphilis, malaria, diarrhea & dysentery | F | Infusion | Oral |  |  |
|  |  |  |  |  | R/L | Infusion of the leaves and roots is used for shortness of breath, chest pain, taken to stimulate the appetite, gastrointestinal remedy, stomach ache and indigestion and treat hepatic diseases. | F | Infusion | Oral |  |  |
|  |  |  |  |  | R/L | Stomach problems, dysentery, colds and cough, reduce fever, treat digestive disturbances, nausea, cough & bronchitis | F | Decoction | Oral |  |  |
|  | *Cyathula tomentosa*(Roth) Moq.  (MNCY- 84) | NE | Namgwet | H | L | The ash from leaves is used skin condition eczema and ringworms. | D | Ash | Oral |  | Amaranthaceae |
|  |  |  |  |  | Tu | Fever, malaria, induce vomiting & evacuate the bowel | F | Infusion | Oral |  |  |
|  |  |  |  |  |  | stomach troubles | F | Chew-Extract | Oral |  |  |
|  |  |  |  |  | R | Root decoction is used to stop excessive bleeding on menstruation. | F | Decoction | Oral |  |  |
|  |  |  |  |  | L | Affected body part | F | Extract | Apply |  |  |
|  |  |  |  |  |  | Hypertension | F | Powder-Infusion |  |  |  |
|  | *Acanthus pubescens* (Oliv.)Engl  (MNCY- 38) | NE | Ndakariat | H/S | L | Calm an upset stomach, ulcers, liver problems, dysentery, UTIs, urethral pain, endometritis, bladder inflammation, threatened abortion, inflammation, aches & pains | F | Decoction | Oral |  | Acanthaceae |
|  |  |  |  |  |  | Unite broken bones and strengthen joints; burnt skin fetches out the fire | F | Paste-Poultice | Apply |  |  |
|  |  |  |  |  |  | An infusion of the leaves is used in treatment of painful teeth as a gargle. | F | Teas-Infusion | Oral/Gargle |  |  |
|  |  |  |  |  |  | Dry cough and chest infections, pneumonia, chronic asthma, cancer, tonsils & flu. (Mireiwek') | D | Ash |  |  |  |
|  | *Basella alba* L.  (MNCY- 1) | NE | Nderemiat | C | R | Reduce bleeding of minor cuts | F | Extract | Apply |  | Basellaceae |
|  |  |  |  |  |  | Diarrhoea | F | Cooked-vegetable | Oral |  |  |
|  |  |  |  |  | L | Boils and sores | F | Paste | Apply |  |  |
|  |  |  |  |  | R | Swellings & rubefacient | F | Paste | Apply |  |  |
|  |  |  |  |  | L/St | Laxative properties | F | Cooked-vegetable | Oral |  |  |
|  |  |  |  |  | FL | Antidote to poisons | F | Extract | Oral |  |  |
|  |  |  |  |  | Fr | Conjunctivitis | F | Extract | Drops |  |  |
|  |  |  |  |  | L | Demulcent, dysentery, diuretic, febrifuge, febrifuge, laxative & aperient for pregnant women | F | Juice | Oral |  |  |
|  |  |  |  |  |  | Alleviate labour | F | Decoction | Oral |  |  |
|  | *Garcinia buchananii* Bak.  (MNCY- 206) | NE | Nderiot | S/T | R | Abdomen pain during pregnancy or shortly after childbirth | F | Infusion | Oral | Milk | Clusiaceae |
|  |  |  |  |  | SB | Chronic diarrhea, dysentery, abdominal pain, inflammation & diabetes | F | Extract | Oral |  |  |
|  |  |  |  |  | St | Malaria | F | Decoction | Oral |  |  |
|  | ***Musa acuminata*Colla** (MNCY- 27) | LC | Ndisyot | H | Fr | Reduce bleeding because of minor cuts & diarrhoea | F | Sap | Apply/Oral |  | Musaceae |
|  |  |  |  |  | St | Epilepsy, leprosy, dysentery & diarrhea | F | Sap | Oral |  |  |
|  |  |  |  |  |  | Insect stings & bite | F | Sap | Apply |  |  |
|  |  |  |  |  |  | Dysentery | F | Pulp-Infusion | Oral |  |  |
|  |  |  |  |  |  | Swellings of the armpit and groin & haemorrhoids | F | Extract | Oral |  |  |
|  |  |  |  |  | R | Convulsions & STDs | F | Decoction | Oral |  |  |
|  |  |  |  |  |  | Reduce bleeding on minor cuts | F | Extract | Apply |  |  |
|  |  |  |  |  |  | Coughing up of blood | F | Decoction | Oral |  |  |
|  |  |  |  |  | Fl | Bronchitis, dysentery, diabetics & ulcers | F | Cooked | Oral |  |  |
|  |  |  |  |  | L | Burns & other skin ailments | F | Poultice | Oral |  |  |
|  |  |  |  |  |  | Painful urination & dysentery | F | Juice | Oral |  |  |
|  |  |  |  |  | Pl | Abortive; salve to ease insect stings and bites & ringworms | F | Decoction | Oral |  |  |
|  |  |  |  |  | Pl/L | Dysentery, diarrhea & malignant ulcers | D | Ashes | Leak |  |  |
|  | *Fuerstia africana* T.C.E.Fr.  (MNCY- 13) | NE | Ngariab sawe | H | L/Sh | Stomach ulcers, wounds from syphilis & tongue infection including oral thrush. | F | Extract | Apply/Oral |  | Lamiaceae |
|  |  |  |  |  | L | Malaria, induce vomiting & anthelmintic - tapeworm | F | Decoction | Oral |  |  |
|  |  |  |  |  |  | Eyes to relieve pain & eye infection | F | Juice | Drops |  |  |
|  |  |  |  |  | R | Cancers, reproduction to facilitate conception | F | Decoction | Oral |  |  |
|  | *Cyathula cylindrica* Moq  (MNCY- 76) | NE | Ng'atumyat | H | B/R | Malaria, leprosy, induce vomiting & a purgative | F | Decoction | Oral |  | Amaranthaceae |
|  | *Leucas calostachys* Oliv.  (MNCY- 137) | NE | Ng'ejepchiat | S/SS | L | Pneumonia, throat infection & serious stomachache | F | Chew-Juice | Oral |  | Lamiaceae |
|  |  |  |  |  |  | Amoebiasis | F | Extract | Oral |  |  |
|  |  |  |  |  | R/L | Heartburns, ulcers, diarrhoea, coughs, colds, flu, pneumonia, abdominal pains & measles | F | Decoction | Oral |  |  |
|  |  |  |  |  | WP | Peptic ulcers, abdominal distention, heartburns & as a synergistic plant in managing several ailments | F | Decoction | Oral |  |  |
|  | *Balanites aegyptiaca* (L.) Delile  (MNCY- 241) | LC | Ng'oswet | S/T | R | The root decoction is used induce vomiting, induce vomiting and clear helminthes. It’s also used to treat malaria. | F | Decoction | Oral |  | Zygophyllaceae |
|  |  |  |  |  |  | Oedema & stomach pains | F | Decoction | Oral | Soup |  |
|  |  |  |  |  | Rb | Purgative and vermifuge | F | Decoction | Oral |  |  |
|  |  |  |  |  | B | Heartburn | F | Infusion | Oral |  |  |
|  |  |  |  |  | G | Chest pains | D | Infusion | Oral | Maize meal porridge |  |
|  | *Fagaropsis angolensis* (Engl.) H.M.Gardner (MNCY- 180) | NE | Noiwet | T | L/R | Back pain, arthritis, joint-aches, malaria, male fertility and cancer | F | Decoction | Oral |  | Rutaceae |
|  |  |  |  |  | Se | Malaria | D | Extract | Oral |  |  |
|  |  |  |  |  | B | Malaria, gonorrhea, pneumonia, back ache and joins, amoeba infections & diarrhea | F | Decoction | Oral |  |  |
|  | *Loeseneriella africana*(Willd.) R.Wilczek  (MNCY- 21) | NE | Ng'ing'ichet | S/L | R | Fever, malaria, body pains, diabetes & diarrhea | F | Decoction | Oral |  | Celastraceae |
|  |  |  |  |  | L/Sb | Liver disease, amoebiasis, stomachache, gastritis, diarrhea, cough and wounds | F | Decoction | Oral |  |  |
|  |  |  |  |  | Lx | Antidote | F | Latex | Apply |  |  |
|  | *Dovyalis abysinicca* (A. Rich.) Warb  (MNCY- 141) | LC | Nukchat/Nokok | T | L | Indigestion | F | Infusion | Oral |  | Salicaceae |
|  |  |  |  |  | R | Epilepsy, cancer & recurrent miscarriages | F | Decoction | Oral |  |  |
|  |  |  |  |  | R | Headache & detoxification |  | Steam | Inhalatiopn |  |  |
|  | *Lepidagathis scariosa* Nees  (MNCY- 36) | NE | Nyamdutiet | H | L | Diarrhoea, wounds, oedema & pneumonia | F | Infusion | Oral |  | Acanthaceae |
|  |  |  |  |  |  | Cough, skin diseases, wounds, eye infections, anti-diarrhea, edema, pneumonia & backache | F | Decoction | Oral |  |  |
|  | *Oxalis corniculata* L. (MNCY- 166) | NE | Nyonyoek | H | L | Inflamed eye-lids | F | Juice | Oral |  | Oxalidaceae |
|  |  |  |  |  |  | Stomach trouble & coughs | F | Extract | Oral |  |  |
|  |  |  |  |  |  | Diarrhoea | F | Extract | Oral | Other plants |  |
|  |  |  |  |  |  | Ringworm | F | Paste | Apply-Rub | Ghee |  |
|  | *Zanthoxylum chalybaeum* Engl.  (MNCY- 239) | LC | Oloisugit | T | B/R | Synergistic herb | F | Decoction | Oral |  | Rutaceae |
|  |  |  |  |  | B | Breast and cervical cancer, malaria | F | Decoction | Oral |  |  |
|  |  |  |  |  | L/R | Joint complaints | F | Decoction | Oral |  |  |
|  |  |  |  |  | R | Whooping cough, TB, STIs |  |  |  |  |  |
|  |  |  |  |  |  | Heart infections | D | Infusion | Oral | Tea/  Porridge |  |
|  |  |  |  |  | L | Chest pain | F | Chew-Extrcat | Oral |  |  |
|  | *Persea americana* Mill.  (MNCY- 50) | LC | Ovacado | T | L/B | Dysentery, relieving coughs, lowering blood pressure, treating liver obstructions, promoting menstrual flow, clearing high uric acid/salts – gout & diarrhoea | F | Infusion | Oral |  | Lauraceae |
|  |  |  |  |  |  | Reduce bleeding from minor cuts, GIT gases or their expulsion, combatting flatulence, colds, coughs, respiratory infections, stimulate menstrual flow when menstruation is absent for reasons other than pregnancy, such as hormonal disorders & manage blood pressure | F | Decoction | Oral |  |  |
|  |  |  |  |  | Se | Scabies, pus producing wounds, injuries of the scalp & dandruffs | D | Powder-Ointment | Apply |  |  |
|  |  |  |  |  | Fr | Expel worms | F | Rind-Infusion | Oral |  |  |
|  |  |  |  |  |  | wounds producing & scalp to promote hair growth | F | Rind-Infusion | Apply |  |  |
|  |  |  |  |  |  | Unripe fruit - induce abortion | F | Extract | Oral |  |  |
|  |  |  |  |  |  | Aphrodisiac | F | Pulp | Oral |  |  |
|  |  |  |  |  |  | Sooth and moisturizes the skin, relieve flatulence & lower blood cholesterol levels | F | Pulp | Apply |  |  |
|  | *Carica papaya* L. (MNCY- 204) | DD | Paipai | S/Ss | Fr | Ringworm | F | Sap | Apply |  | Caricaceae |
|  |  |  |  |  | Se | Skin infections | F | Extract | Apply |  |  |
|  |  |  |  |  | Fr | Flatulence, indigestion, relieve gas, inflammation, promote urination, & boost appetite | F | Pulp | Oral |  |  |
|  |  |  |  |  | L/Se | Amoebiasis & expel worms | F | Extract | Oral |  |  |
|  |  |  |  |  | L | Malaria | F | Steam | Inhalation |  |  |
|  |  |  |  |  |  | Malaria | F | Decoction | Oral |  |  |
|  |  |  |  |  |  | Hypertension | F | Decoction | Oral | Lemon  grass/  guava leaves |  |
|  | *Acmella*Rich. ex Pers.  (MNCY- 176) | NE | Putputik | H | FL | Oral thrush | F | Extract | Oral |  | Asteraceae |
|  |  |  |  |  | WP/FL | Toothache, wounds in the mouth, sores on the tongue, ulcers, halitosis & sore throat | F | Juice | Oral |  |  |
|  | *Kigelia africana* (Lam.) Benth  (MNCY- 144) | LC | Ratinuet | T | B | A decoction from the bark is drunk to cure syphilis and gonorrhea. | F | Decoction | Oral |  | Bignoniaceae |
|  |  |  |  |  | B/R | Toothache | D | Powder/Infusion | gargle |  |  |
|  |  |  |  |  |  | Ulcers, pneumonia, malaria, headaches, diabetes post-partum & haemorrhage | D | Powder/Infusion | Oral |  |  |
|  |  |  |  |  | R | Root decoction is used on peptic ulcers and breast cancers. | F | Decoction | Oral |  |  |
|  |  |  |  |  | Se | Enlarge sexual organs of men | D | Roast-Decoction | Oral |  |  |
|  |  |  |  |  | Fr | Measles | F | Fermented-Extract | Bath |  |  |
|  |  |  |  |  | L | Malaria & backaches | F | Decoction | Oral |  |  |
|  | *Ocimum gratissimum* L. (MNCY- 134) | NE | Chepchai/Rekeriot | S | FL/L | Teas and infusion. This is where they get their local name “Chepchai”. | F | Teas-Infusion | Oral |  | Lamiaceae |
|  |  |  |  |  | L | Wounds | F | Juice | Apply |  |  |
|  |  |  |  |  |  | Measles | F | Infusion | Bath |  |  |
|  |  |  |  |  |  | Sore eyes | F | Infusion | Drops |  |  |
|  |  |  |  |  |  | Abdominal pains | F | Infusion | Oral |  |  |
|  |  |  |  |  |  | Upper respiratory tract infections, diarrhoea, headache, diseases of the eye, skin diseases, pneumonia, cough & fever | F | Decoction | Oral |  |  |
|  |  |  |  |  |  | Blocked nostrils | F | Extract | Snuff |  |  |
|  |  |  |  |  |  | Abdominal pains, sore eyes, cancer and blood purifier, ear infections, coughs, barrenness, fever, convulsions and tooth gargle, haemorrhoids, regulation of menstruation; prolapse of the rectum, mental illness, management of the baby’s cord; keep the wound surfaces sterile; fungal infections, fevers & cold | F | Decoction | Oral |  |  |
|  |  |  |  |  | R | Sedative, treatment of epilepsy, high fever & diarrhoea | F | Decoction | Oral |  |  |
|  | ***Rauvolfia caffra* Sond.  (MNCY- 145) | NE | Rerendet | T | St | Rreduce bleeding on minor cuts, purgative/ induce vomiting, fever, swellings, abscesses, hepatitis & pneumonia | F | Decoction | Oral |  | Apocynaceae |
|  |  |  |  |  | B | Powdered bark is applied to skin rashes caused by measles, urticaria and other forms of rashes. | D | Powder | Apply |  |  |
|  |  |  |  |  | R | Root juice mixed with honey is applied on fractures | F | Juice | Apply |  |  |
|  |  |  |  |  |  | Coughs, diarrhea, gastrointestinal disturbances, skin infections, hypertension, dysentery, scabies, worm infections & malaria | F | Decoction | Oral |  |  |
|  |  |  |  |  | B | General body swellings, rheumatism, pneumonia, malaria, diabetes; parasitic & microbial infections | F | Decoction | Oral |  |  |
|  |  |  |  |  | SB | Cough, toothaches & venereal diseases | F | Chew-Extract | Oral |  |  |
|  |  |  |  |  |  | Measles, skin lesions or itching rashes | F | Extract | Apply |  |  |
|  | *Gouania longispicata* Engl.  (MNCY- 86) | LC | Riksoet/Kirkipsoo | L | L | Oral thrush | F | Extract | Apply |  | Rhamnaceae |
|  |  |  |  |  | R | Venereal diseases, stomachaches, allergy, urinary retention, syphilis, tooth decay, sore throat, wounds, itchy eyes, inflammations, skin infections, mastitis, worms, headache, asthma, body weakness, itchy body & colic pains | F | Decoction | Oral |  |  |
|  |  |  |  |  | St | Wounds | F | Sap | Apply |  |  |
|  | *Microglossa pyrifolia* (Lam.) O.Kuntze  (MNCY- 171) | NE | Rirmosok/  Nanwaket | S/C | R | Headache & colds | F | Infusion | Oral |  | Asteraceae |
|  |  |  |  |  | L | Fever, malaria, abdominal pain, diarrhoea & rheumatism | F | Extract | Oral |  |  |
|  |  |  |  |  | R | Oral thrush, meningitis & fever symptoms | F | Decoction | Oral |  |  |
|  |  |  |  |  | L | Limb fractures | F | Paste | Apply-Tied |  |  |
|  |  |  |  |  |  | Malaria & induce vomiting | F | Infusion | Oral |  |  |
|  | *Justicia flava* Vahl (MNCY- 242) | VU | Rokorab chepkimis/  Chepyochoit | H | R | Chronic wounds, boils, burns & stomach ulcer | F | Poultice | Apply/Oral |  | Acanthaceae |
|  |  |  |  |  |  | Stomachache, skin infections & diarrhoea | F | Cooked-Extact | Oral |  |  |
|  |  |  |  |  |  | Coughs | F | Extract | Oral |  |  |
|  |  |  |  |  | L | Induce vomiting eye lotion, cough, TB, paralysis, fever, epilepsy, convulsion; spasm, skin infections and disorders | F | Decoction | Oral |  |  |
|  | *Zanthoxylum gilletii* (De Wild.) P.G.Waterman (MNCY- 199) | LC | Sagawatiet | T | B | Stomachache & alleviate toothache | F | Juice | Oral |  | Rutaceae |
|  |  |  |  |  | L | Snake bites & enlarged spleen | F | Sap | Apply |  |  |
|  |  |  |  |  |  | Heart complaints, diarrhoea, gastritis & snake bites | F | Extract | Oral |  |  |
|  |  |  |  |  |  | Cough gonorrhea, schistosomiasis | F | Decoction | Oral |  |  |
|  |  |  |  |  | B | Cough, colds, skin complaints & smallpox | F | Extract | Apply |  |  |
|  |  |  |  |  |  | Gonorrhea, urogenital problems-kidney complaints; & vermifuge | F | Decoction | Oral |  |  |
|  |  |  |  |  |  | Diarrhoea | F | Enema | Apply |  |  |
|  |  |  |  |  | B/St | Analgesic, burns, rheumatism, headache, stomach-ache, toothache colic, fever, aphrodisiac & pain after childbirth | F | Decoction | Oral |  |  |
|  | *Polyscias kikuyuensis* Summerh.  (MNCY- 236) | NT | Saiyet | T | B | Mental illness. | F | Maceration-Extract | Drops |  | Araliaceae |
|  |  |  |  |  |  | Fever, malaria & purgative | F | Infusion/decoction | Oral |  |  |
|  |  |  |  |  |  | Epilepsy | F | Decoction | Oral | Other plants |  |
|  |  |  |  |  |  | Colic | F | Enema | Apply |  |  |
|  |  |  |  |  |  | Painkiller, coughs, haemoptysis & TB | D | Powder | Snuff |  |  |
|  |  |  |  |  | L | Intestinal complaints including parasites | F | Decoction | Oral |  |  |
|  |  |  |  |  |  | Peptic ulcers. | F | Extract | Oral |  |  |
|  |  |  |  |  |  | Fractures | F | Paste | Apply |  |  |
|  | *Lepidotrichilia volkensii* (Gürke) J.-F.Leroy  (MNCY- 184) | LC | Sakamwet | T | R | Joint pain, gout symptoms, female sterility & inflammation | F | Decoction | Oral |  | Meliaceae |
|  |  |  |  |  | Bd | Ulcer management & leucorrhoea | F | Decoction | Oral |  |  |
|  |  |  |  |  | Fr | Fever & cancer | F | Decoction | Oral |  |  |
|  |  |  |  |  | B | Stomachaches & coughs | F | Decoction | Oral |  |  |
|  | *Cordia africana* Lam.  (MNCY- 135) | LC | Samutet | T | B | Broken bone | F | Pieces | Tie |  | Boraginaceae |
|  |  |  |  |  | L | Headache, cardiovascular diseases, diabetes, nose bleeding, dizziness; vomiting during pregnancy, wounds & worms | F | Decoction | Oral |  |  |
|  |  |  |  |  | B | Fatigue | F | Extract | Oral |  |  |
|  |  |  |  |  |  | Malaria and its related symptoms | D | Powder-Infusion | oral |  |  |
|  | *Podocarpus latifolius* (Thunb.) R.Br. ex Mirb.  (MNCY- 121) | LC | Saptet | T | B | Stomach pains, malaria, asthma, bronchitis, toothaches constipation & gonorrhoea | F | Decoction | Oral |  | Podocarpaceae |
|  |  |  |  |  |  | Stomachache, chest complaints & headaches | F | Infusion | Oral |  |  |
|  |  |  |  |  | L | Vermifuge | F | Decoction | Oral |  |  |
|  | *Ensete ventricosum* (Welw.) Chessman (MNCY- 155) | LC | Sasuruet | H | St/L | Liver & miscarriage problems | F | Decoction | Oral |  | Musaceae |
|  |  |  |  |  | L | Stimulate labour or induce abortion | F | Decoction | Oral |  |  |
|  |  |  |  |  | Fr/L | Hepatitis and other liver complaints | Dry | Ash | Leak |  |  |
|  |  |  |  |  |  |  |  | Infusion | Oral |  |  |
|  |  |  |  |  | Se | Treat wounds | D | Powder | Apply |  |  |
|  | *Spathodea campanulata* Buch.-Ham. ex DC.  (MNCY- 223) | LC | Sebetaiyat | T | B | Oedemas, skin diseases like herpes & sores | F | Pulp | Oral |  | Bignoniaceae |
|  |  |  |  |  |  | Dysentery & stomachache | F | Infusion | Oral |  |  |
|  |  |  |  |  | L | Kidney disease, gonorrhoea, urethra inflammation, pain, constipation, dysentery; women‘s pelvic disorders & as an antidote | F | Decoction | Oral |  |  |
|  |  |  |  |  | FL | Diuretic & anti-inflammatory | F | Decoction | Oral |  |  |
|  |  |  |  |  | B/FL | Wound & ulcers | F | Paste | Apply |  |  |
|  |  |  |  |  | L | Urethral inflammation | F | Infusion | Oral |  |  |
|  |  |  |  |  |  | Epilepsy and convulsion; kidney diseases, urethritis & antidote | F | Decoction | Oral |  |  |
|  |  |  |  |  | B | Kidney disorders, eczemas, fungal skin disease, herpes, stomachache & diarrhea | F | Decoction | Oral |  |  |
|  | *Ficus sycomorus* L.  (MNCY- 107) | LC | Sebetuet | T | B | Liver diseases, pain killer, parasitic infection, epilepsy, wounds, diarrhea, dysentery, convulsions, vomiting, snake bite, jaundice, coughs & mental illness | F | Decoction | Oral |  | Moraceae |
|  |  |  |  |  | Fr/L/  SB | Cough, diarrhea, skin infections, stomach disorders, liver disease, epilepsy, TB, lactation disorders, expel worms, infertility, sterility & diabetes | F | Extract | Oral |  |  |
|  |  |  |  |  | L | Anthelmintic & purgative | F | Extract | Oral |  |  |
|  |  |  |  |  | SB | Cough, throat infection & chest pains | F | Decoction | Oral |  |  |
|  |  |  |  |  | St | Chest diseases, colds & dysentery | F | Latex | Oral |  |  |
|  | *Albizia gummifera* sensu Capuron, p.p.  (MNCY- 30) | LC | Seet | T | B | Boils, abscesses, cough, eye, flu, puffy gums and sometimes tender or that bleed easily, lung problems, tonic, abdominal tumors, malaria, pectoral problems & “Kiptumarit | F | Decoction | Oral |  | Fabaceae |
|  |  |  |  |  | R | Anxiety, depression, sleep problems, sore throat, improve mood, treat fractures, reduce swellings associated with trauma, sprains & heal fractures | F | Decoction | Oral |  |  |
|  |  |  |  |  | Pd | Stomach pains | F | Extract | Oral |  |  |
|  |  |  |  |  | R | skin diseases | F | Extract | Bath |  |  |
|  | *Senna didymobotrya*(Fresen.) H.S.Irwin & Barneby  (MNCY- 235) | LC | Senetwet | S | L/St/R | Purgative | F | Decoction | Oral |  | Fabaceae |
|  |  |  |  |  | L | Gonorrhea & backaches in women. | F | Cooked-Decoction | Oral |  |  |
|  |  |  |  |  |  | Measles | F | Decoction | Bath |  |  |
|  |  |  |  |  |  | Stomach troubles. | F | Decoction | Oral |  |  |
|  |  |  |  |  | R/L | Fever, headaches & bile | F | Decoction | Oral |  |  |
|  |  |  |  |  | L | The burnt ash from leaves are applied ringworms and skin rush “Musamusik”. | D | Ash | Leak |  |  |
|  |  |  |  |  | R | Antidote for general poisoning | F | Decoction | Oral |  |  |
|  |  |  |  |  | B/R | Purgative & induce vomiting | F | Infusion | Oral |  |  |
|  | *Laggera crispata*(Vahl) Hepper & J.R.I.Wood  (MNCY-66) | NE | Serkutiet | H | R | Gonorrhea/STI’s & cancer | F | Decoction | Oral |  | Asteraceae |
|  |  |  |  |  |  | Hiccups | F | Juices | Oral |  |  |
|  |  |  |  |  |  | Colds, coughs, side stitch & expectorant | F | Infusion | Oral |  |  |
|  |  |  |  |  | L | Spider bites | F | Extract | Apply |  |  |
|  |  |  |  |  |  |  | D | Ash | Apply |  |  |
|  | *Vachellia nilotica*(L.) *P.J.H.Hurter & Mabb.* (MNCY- 252) | LC | Sertwet | T | SB | Sore throat & coughs | F | Juices | Oral |  | Fabaceae |
|  |  |  |  |  | L/B | Colds & pneumonia | F | Teas | Oral |  |  |
|  |  |  |  |  | L | Eyes at night - conjunctivitis | D | Powder | Apply |  |  |
|  |  |  |  |  | Fr | Ulcers & venereal disease | F | Decoction | Topical |  |  |
|  |  |  |  |  | B/R | Aphrodisiac | F | Decoction | Oral |  |  |
|  |  |  |  |  | R | Indigestion or stomach trouble, gonorrhea & chest diseases | F | Decoction | Oral |  |  |
|  |  |  |  |  | L | Chest pains &r pneumonia | F | Decoction | Oral |  |  |
|  |  |  |  |  | R | Impotence, gonorrhea treatment & UTIs | F | Decoction | Oral |  |  |
|  |  |  |  |  | B | Cancer, diarrhea and accompanying intestinal pains; leucorrhea, fever in children, aid digestion & powerful stimulant | F | Decoction | Oral |  |  |
|  |  |  |  |  |  | Reduce bleeding of minor cuts, tightening and toning tissues, excess mucus along with swollen and inflamed tissues | F | Decoction | Oral |  |  |
|  | *Clematis simensis* Fresen.  (MNCY- 247) | NE | Sesiat | C | L | Warts & swellings | F | Extract | Apply |  | Ranunculaceae |
|  |  |  |  |  |  | Headaches, digestive system disorders, inflammations colds & eczema | F | Decoction | Oral |  |  |
|  |  |  |  |  | St | Headaches & colds | F | Extract | Oral |  |  |
|  |  |  |  |  | L | Cancer | F | Extract | Oral |  |  |
|  |  |  |  |  | L/FL | Epilepsy and Bell’s palsy disease “Kiptumarit”. | F | Extract | Inhalation |  |  |
|  |  |  |  |  | L | Malaria, cough & liver diseases | D | Powder | Snuff |  |  |
|  | *Mimulopsis solmsii* Schweinf.  (MNCY- 192) | NE | Setyot | S | L | Inflammations, malaria, venereal diseases & anthelmintic | F | Decoction | Oral |  | Acanthaceae |
|  |  |  |  |  |  | Skin diseases and snakebites | F | Extract | Bath |  |  |
|  |  |  |  |  | St | Eye infections | F | Extract | Drops |  |  |
|  |  |  |  |  | R | Malaria & purgative | F | Decoction | Oral |  |  |
|  | *Chlorophytum gallabatense*Schweinf. ex Baker  (MNCY- 5) | NE | Sigotiet | H | R | Deworming, rheumatism, cough & cold | F | Decoction | Oral |  | Asparagaceae |
|  |  |  |  |  |  | Aphthae of mouth and throat | D | Fry-Powder | Oral |  |  |
|  | *Solanum aculeastrum* Dunal  (MNCY- 142) | LC | Sigowet | S/ST | Fr/L | Cancer, indigestion & stomach disorders | F | Decoction | Oral |  | Solanaceae |
|  |  |  |  |  | Be | Breast cancer, stomach disorders & various cancers | F | Decoction | Oral |  |  |
|  |  |  |  |  | Rb | Sexually transmitted bacterial diseases e.g. gonorrhea & acne | F | Decoction | Oral |  |  |
|  |  |  |  |  | Fr/L | Jigger infestations, wounds, swollen joints in fingers, gangrene, toothaches, gonorrhea, bronchitis, rheumatism, ringworm & eyewash | F/D | Decoction/ Powder/ Ash | Oral/Apply |  |  |
|  |  |  |  |  | R | Bronchitis, gonorrhea & skin rashes | F | Decoction | Oral | Meat broth |  |
|  |  |  |  |  | Be | Jigger wounds, gonorrhea & acne | F | Decoction | Oral |  |  |
|  | *Lagenaria siceraria* (Molina) Standl.  (MNCY- 80) | NE | Silakwet | H/V | L | Haemorrhoidal swellings | F | Poultice | Apply |  | Cucurbitaceae |
|  |  |  |  |  |  | Relief headache, induce vomiting | F | Juice | Snuff |  |  |
|  |  |  |  |  |  | Baldness &headache | F | Extract | Apply |  |  |
|  |  |  |  |  |  | Jaundice | F | Decoction | Oral | Sugar |  |
|  |  |  |  |  | Be | Jigger wounds, gonorrhea & acne | F | Decoction | Oral |  |  |
|  |  |  |  |  | Fr | Diuretic, coughs & antidote | F | Pulp | Oral |  |  |
|  |  |  |  |  | L | Headaches | F | Poultice | Apply |  |  |
|  |  |  |  |  | Fr | Cure pain, ulcers, fever; pectoral cough, asthma & other bronchial disorders | F | Syrup | Oral |  |  |
|  |  |  |  |  | FL | Antidote to poison | F | Extract | Oral |  |  |
|  |  |  |  |  | Fr | Diuretic & uterus/ womb problems | F | Rind-Extract | Oral |  |  |
|  |  |  |  |  |  | Stomach acidity, indigestion & ulcers | F | Juice | Oral |  |  |
|  |  |  |  |  | L | Alternative purgative | F | Decoction | Oral |  |  |
|  |  |  |  |  | St | Diuretic | F | Decoction | Oral |  |  |
|  |  |  |  |  | Se | Boils | F | Boiled-Poultice | Apply |  |  |
|  |  |  |  |  | R | Induce vomiting & oedema | F | Decoction | Oral |  |  |
|  | *Dombeya torrida* (J.F.Gmel.) Bamps (MNCY- 28) | NE | Silpchet | S/ST | B | Allergies & joint pains | F | Decoction | Oral |  | Malvaceae |
|  |  |  |  |  | FL/B | Digestive problems | F | Decoction | Oral |  |  |
|  |  |  |  |  | R | Colic relief in infants, asthma, chest pain, backache, typhoid fever & colds | F | Decoction | Oral |  |  |
|  |  |  |  |  | B | Headache & stomachache | F | Decoction | Oral |  |  |
|  |  |  |  |  | Rb | Wound | F | Paste-dressing | Apply |  |  |
|  |  |  |  |  | St/B | Diarrhoea and stomachaches | F | Decoction | Oral |  |  |
|  | *Buckollia volubilis*(Schltr.) Venter & R.L.Verh.  (MNCY- 111) | NE | Simatwet | T | R | Gonorrhea/STI’s, stomachaches &cancer | F | Decoction | Oral |  | Apocynaceae |
|  |  |  |  |  | St | Malaria | F | Decoction | Oral |  |  |
|  |  |  |  |  | Tu | Galactagogue | F | Decoction | Oral |  |  |
|  |  |  |  |  | R | Enhance lactation | F | Extract | Oral |  |  |
|  |  |  |  |  | SB | childbirth | F | Decoction | Oral |  |  |
|  | ***Cyphostemma orondo* (Gilg & M.Brandt) Desc. (MNCY- 217) | NE | Simet | C | R | Cancer “Chepserkechet” | F | Infusion | Oral |  | Vitaceae |
|  |  |  |  |  | L | Insect bites specifically caterpillar “Melmeldo” | F/D | Extract/Ash | Apply |  |  |
|  |  |  |  |  |  | Abscesses and boils | F | Extract | Apply |  |  |
|  |  |  |  |  |  | Low back pain | F | Sap | Apply |  |  |
|  | *Ficus thonningii*Blume  (MNCY- 234) | LC | Simotuet | T | St | Influenza, sore throat, colds, arthritis, stimulate lactation, rheumatism & relieve inflammation | F | Infusion | Oral |  | Moraceae |
|  |  |  |  |  | B | Diarrhoea, cysts, skin diseases, ulcers; enhance fertility; induce the menstrual cycle, relief stomach pains, chest pains, diarrhoea, pneumonia, constipation, bowel disorders, malaria, fever, hepatitis & dental pains | F | Decoction | Oral |  |  |
|  |  |  |  |  | St | Fever, tooth decay, ringworm, wounds, bone movement disorders, thrush, scabies & athlete's foot rot | F | Latex | Oral |  |  |
|  | *Periploca linearifolia* Dillon & A.Rich.  (MNCY- 228) | NE | Sinendet | H/L | L/R | Wounds, skin diseases, whitlow, warts & ulcers | F | Latex | Apply |  | Apocynaceae |
|  |  |  |  |  | L/R | Skin diseases | F | Decoction | Oral |  |  |
|  |  |  |  |  | RB | Tapeworm and other intestinal worms & stomachache | F | Infusion | Oral |  |  |
|  |  |  |  |  |  |  | F | Enema | Apply |  |  |
|  |  |  |  |  | R | Fever, malaria, chest complaints, including cough; pneumonia, female infertility; venereal diseases especially gonorrhoea & syphilis | F | Decoction | Oral | Soup |  |
|  |  |  |  |  |  | Haemorrhoids | D | Powder | Apply |  |  |
|  |  |  |  |  |  |  | F | Latex | Apply |  |  |
|  |  |  |  |  |  | Rectal prolapse | F | Juice | Oral | Beer |  |
|  |  |  |  |  | L | Female sterility | F | Pounded-Extract | Oral |  |  |
|  |  |  |  |  |  | Galactagogue. | F | Decoction | Oral | Other plants |  |
|  |  |  |  |  |  | Mastitis | F | Maceration-Extract | Apply |  |  |
|  |  |  |  |  |  | Snakebites | F | Extract | Apply | Other plants |  |
|  |  |  |  |  |  | Diarrhoea, intestinal worms & insanity | F | Decoction | Oral |  |  |
|  |  |  |  |  |  | Toni in pregnant women & stop vomiting | F | Sap/Decoction | Oral |  |  |
|  |  |  |  |  |  | Malnutrition | F | Decoction | Oral |  |  |
|  | *Rubia cordifolia* L.  (MNCY- 159) | NE | Sing'ortet | H/C | R | Stomach disorders, sores in the mouth, blood purification by detoxifying, blood clots; unblocking any obstructions in the circulatory system & antidote | F | Decoction | Oral |  | Rubiaceae |
|  |  |  |  |  | WP | Regulate blood pressure, irregular menstruation blood; vessel constriction, blood clot formation, gout, arthritis; urinary infections, diarrhea, dysentery & chronic fevers | F | Decoction | Oral |  |  |
|  |  |  |  |  | WP | Ring worms, pimples, freckles, other discolorations, promotes the healing of skin tissues damaged by injury or infection, skin diseases like eczema, dermatitis & skin ulcers | D | Ash | Apply | Ghee |  |
|  |  |  |  |  | St/L | Mouth sores | F | Extract | Apply |  |  |
|  |  |  |  |  |  | Diarrhoea & bleeding gums | F | Extract | Oral |  |  |
|  | *Clerodendrum johnstonii* Oliv. (MNCY- 253) | LC | Singoruet | S/ST | R | Facilitate-walking in infants | D | Burned-Powder | Apply |  | Lamiaceae |
|  |  |  |  |  | L | Abdominal pains, abscess, stomach troubles & hernia | F | Juice | Oral |  |  |
|  |  |  |  |  | R | Coughs & chest pain relief | F | Decoction | Oral |  |  |
|  |  |  |  |  | L | Toothache | F | Extract | Apply |  |  |
|  |  |  |  |  | R | Abdominal pains and joint pains. | F | Decoction | Oral |  |  |
|  |  |  |  |  | R/St/L | Stomachache & malaria | F | Decoction | Oral |  |  |
|  | *Searsia pyroides*(Burch.) Moffett  (MNCY- 109) | NE | Siriat (ne isururu) | S/ST | R | Dysentery, hemorrhoids & sterility | F | Decoction | Oral |  | Anacardiaceae |
|  |  |  |  |  | R/St/L | Colds, abdominal pains, pneumonia & gonorrhea | F | Decoction | Oral |  |  |
|  |  |  |  |  | St | Coughs | F | Decoction | Oral |  |  |
|  |  |  |  |  | L | Sterility | F | Decoction | Oral |  |  |
|  |  |  |  |  | R | Syphilis, amoebiasis, fever & stomachaches | F | Infusion | Oral |  |  |
|  |  |  |  |  |  | Antidote | F | Decoction | Oral | Milk |  |
|  |  |  |  |  |  | Measles | F | Infusion | Bath |  |  |
|  | ***Searsia natalensis*(Bernh. ex C.Krauss) F.A.Barkley** (MNCY- 69) | LC | Siriat(monjororiat) | S/ST | R | Influenza, abdominal pains & gonorrhoea | F | Extract | Oral |  | Anacardiaceae |
|  |  |  |  |  |  | Hookworm | F | Decoction | Oral |  |  |
|  |  |  |  |  | L | cough | F | Extract | Oral |  |  |
|  |  |  |  |  |  | Colds | F | Steam | Inhalation |  |  |
|  |  |  |  |  |  | Stomachache & abdominal pains- in young children. | F | Decoction | Oral |  |  |
|  | *Ocimum basilicum* L.  (MNCY- 219) | NE | Sisiyat | S | R/AP | Headaches, insect bites, coughs, diarrhea, constipation, evacuation of worms, kidney cleansing, stomachaches, diabetes, stomach spasms, boost appetite, intestinal gas & fluid retention | F | Decoction | Oral |  | Lamiaceae |
|  |  |  |  |  | AP | Infusion of the aerial part is used before and after childbirth to promote blood circulation and to start the flow of breast milk. | F | infusion | Oral |  |  |
|  |  |  |  |  | R | Cancer, detoxification, gargle for oral and dental care, inflammation, swelling, convulsion, boost immunity, earaches, diarrhea, epilepsy, insanity, sore throat & high pitched cough that is likely to be whooping cough | F | Decoction | Oral |  |  |
|  |  |  |  |  | L | Sinusitis, nose block & colds | F | Extract | Rub-Inhalation |  |  |
|  |  |  |  |  |  | Skin infections | F | Paste | Massage |  |  |
|  | *Urtica massaica* Mildbr.  (MNCY- 9) | NE | Siwot | H | R | Stomachache, constipation, high blood pressure, night sweats, diabetes & indigestion | F | Decoction | Oral |  | Urticaceae |
|  |  |  |  |  | L | Malaria & diabetes | F | Decoction | Oral |  |  |
|  |  |  |  |  | R/L | Hepatic diseases & blood purifier | F | Macerated-Extract | Oral |  |  |
|  |  |  |  |  | WP | Numerous ailments, including bruises, injuries, fractures, venereal diseases, hair loss, rheumatism & urethral leak | F | Decoction | Oral | Other plants |  |
|  |  |  |  |  | R/L | Painful muscle and joints, arthritis, gout, anaemia, high blood pressure, sexual stimulant, injury & typhoid | F | Decoction | Oral |  |  |
|  |  |  |  |  | AP | Skin cancer | F | Powder | Apply |  |  |
|  |  |  |  |  | R | Aphrodisiac | F | Decoction | Oral |  |  |
|  |  |  |  |  |  |  |  |  |  |  |  |
|  |  |  |  |  | St/R | Bruises, fractures, venereal diseases, stomach problems, skin infections, bladder complications, cough & headache | F | Decoction | Oral | Other plants |  |
|  | *Warburgia ugandensis* Sprague  (MNCY- 246) | CE* | Soget | T | B | Stomachache, constipation, coughs, fever, toothache, muscle pains, weak joints, fever or malaria & general body pains | D | Juice | Oral |  | Canellaceae |
|  |  |  |  |  |  | Prevention of diarrhoea | D | Powder | Oral | Soup |  |
|  |  |  |  |  | L | Leaves are chewed to relief coughs | F | Extract | Oral |  |  |
|  |  |  |  |  | B/L | Malaria | F | Decoction | Oral |  |  |
|  |  |  |  |  | B/R | Pneumonia, asthma, malaria, candidiasis, skin infections, opportunistic infections, diarrhea gastro-intestinal disorders like cholera, diarrhea and dysentery; cold, cough and sore throat, respiratory and dental problems & measles | F | Decoction | Oral |  |  |
| 1. 7 | *Polyscias fulva* (Hiern) Harms  (MNCY- 51) | LC | Soiyet | T | B | Fever, malaria, respiratory disorders, venereal infections & purgative | F | Infusion | Oral |  | Araliaceae |
|  |  |  |  |  |  | Colic | F | Paste-Enema | Apply |  |  |
|  |  |  |  |  |  | Epilepsy | F | Decoction | Oral | Other plants |  |
|  |  |  |  |  |  | mental illness |  | Macerated-Extract | Drop |  |  |
|  |  |  |  |  |  | Painkiller, coughs & TB | F | Pulverized-Extract | Snuff |  |  |
|  |  |  |  |  | L | Intestinal complaints including those caused by parasites | F | Decoction | Oral |  |  |
|  |  |  |  |  |  | Peptic ulcers | F | Decoction | Oral |  |  |
|  |  |  |  |  |  | Fractures | F | Paste | Apply |  |  |
|  |  |  |  |  |  | Leaves infusion is drunk for headaches relief | F | Infusion | Oral |  |  |
|  |  |  |  |  |  | Allergies | D | Ash | Leak |  |  |
|  | *Phoenix reclinata* Jacq*.*  (MNCY- 170) | LC | Sosiot | T | R | Reproductive issues, abdominal complaints, fevers, loss of consciousness, constipation, toothache, nervous debility; helminthiasis & heart complaints | F | Decoction | Oral |  | Arecaceae |
|  |  |  |  |  | Fr | Sore throats, fever, colds, intestinal troubles, bronchial catarrh, gonorrhea, edema, cysts; liver & abdominal issues | F | Extract | Oral |  |  |
|  |  |  |  |  | St | Gonorrhea & anaemia | F | Sap | Oral |  |  |
|  | *Baccharoides lasiopus* (O.Hoffm.) H.Rob.  (MNCY- 212) | NE | Sugumeriet | S/ST | Se | Clean stomach parasites/deworming | F | Extract | Oral |  | Asteraceae |
|  |  |  |  |  | B/L/R | Colds, pneumonia, cough & malaria | F | Decoction | Oral |  |  |
|  |  |  |  |  | L | Stomachache, back pain, aphrodisiac, diabetes, liver diseases & jaundice | F | Extract/Decoction | Oral |  |  |
|  |  |  |  |  | R | Stomachache & sex stimulant - men | F | Decoction | Oral |  |  |
|  |  |  |  |  | L/R | Abdominal pains, indigestion & purgative | F | Decoction | Oral |  |  |
|  |  |  |  |  |  | Relieving labour and postpartum pain, colic pain, fever, abdominal pain, constipation, diarrhoea, indigestion, stomachaches, heart burn, inflammation & splenomegaly “kipkarasonit” | F | Decoction | Oral |  |  |
|  |  |  |  |  | R/St/L | Root, stem and leave decoction as anthelminthic | F | Decoction | Oral |  |  |
|  |  |  |  |  | St | Convulsions, epilepsy, fainting, colorectal cancer & sores | F | Decoction | Oral |  |  |
|  | *Scutia myrtina  Kurz* (MNCY- 197) | LC | Sumbeiwet | S/C | L | Hasten parturition of both the baby and the placenta | F | Ointment | Apply |  | Rhamnaceae |
|  |  |  |  |  | Rb | Fever | F | Decoction | Oral |  |  |
|  |  |  |  |  |  | Malaria | F | Infusion | Oral |  |  |
|  |  |  |  |  | L/Rb | Gonorrhea & intestinal worms | F | Decoction | Oral |  |  |
|  |  |  |  |  | R | Blood detoxification | F | Decoction | Oral |  |  |
|  | *Stephania abyssinica* Walp.  (MNCY- 55) | NE | Tabarariet | L | R | Aphrodisiac, anthelminthic, jaundice, cardiovascular disordersascariasis & menorrhagia | F | Decoction | Oral |  | Menispermaceae |
|  |  |  |  |  | WP | Aching | D | Powder | Apply |  |  |
|  |  |  |  |  |  | Common cold | F | Steam | Inhalation |  |  |
|  | *Dodonaea viscosa Jacq* var. *angustifolia* (L.f) Benth*.*  (MNCY- 222) | LC | Tabilikwet | S/ST | St/L | Fever | F | Decoction | Oral |  | Sapindaceae |
|  |  |  |  |  | L | Relieve itching, fevers, swellings, aches & malaria | F | Decoction | Oral |  |  |
|  |  |  |  |  | R | Anthelmintic | F | Pulverized-Extracts | Oral |  |  |
|  |  |  |  |  | R | Stimulate lactation, dysmenorrhea & irregular menstruation | F | Decoction | Oral |  |  |
|  |  |  |  |  | R/B | Stop bleeding during pregnancy | F | Decoction | Oral |  |  |
|  |  |  |  |  | L | Mild purgative, rheumatism, sore throat & haemorrhoids | F | Extract | Oral |  |  |
|  |  |  |  |  |  | Trachoma | F | Juice | Drops |  |  |
|  |  |  |  |  |  | Roundworms | D | Powder-infusion | Oral |  |  |
|  |  |  |  |  | L/R | Digestive system disorders, including indigestion & diarrhea | F | Decoction | Oral |  |  |
|  |  |  |  |  | Se | Sore throat treatment & malaria | F | Extract | Oral |  |  |
|  |  |  |  |  | L/R | Painkiller to soothe toothaches, pneumonia, TB, skin rashes & headaches | F | Extract | Oral |  |  |
|  |  |  |  |  |  | Throat & oral thrush | F | Extract | Gargle |  |  |
|  |  |  |  |  | L | Itchy skin, skin rashes, sprains, bruises, burns & wounds | F | Lotion | Apply |  |  |
|  |  |  |  |  | R | Root infusion is good to treat colds. | F | Infusion | Oral |  |  |
|  |  |  |  |  | St | Rheumatism, colds, influenza, stomach trouble & measles | F | Decoction | Oral |  |  |
|  | *Oncoba routledgei* Sprague  (MNCY- 148) | NE | Takurkurwet | S/ST | R | Dysentery, headache, tonic &urinary problems | F | Decoction | Oral |  | Salicaceae |
|  |  |  |  |  | St | Stomachache & loss of appetite | F | Decoction | Oral |  |  |
|  |  |  |  |  | Tw/R | Sores & venereal diseases | F | Decoction | Wash |  |  |
|  |  |  |  |  | L/R | Urethral discharges | F | Decoction | Oral |  |  |
|  |  |  |  |  | L | Vertigo | F | Sap | Oral |  |  |
|  | *Aloe kedongensis* Reynolds  (MNCY- 136) | NE | Tangaratwet | H/SS | R | Colic, typhoid, wounds, oral thrush, colic & malaria | F | Decoction | Oral |  | Asphodelaceae |
|  |  |  |  |  | L | Colds, fever, diarrhoea & malaria | F | Decoction | Oral |  |  |
|  |  |  |  |  |  | Ear infection | F | Sap | Apply |  |  |
|  |  |  |  |  |  | Intestinal pain, digestive disorders & rheumatic arthritis | F | Decoction | Oral |  |  |
|  |  |  |  |  |  | Purgative, blood cleanser, allergy & typhoid | F | Juice | Oral |  |  |
|  | *Gloriosa superba* L. (MNCY- 19) | LC | Taptet ne biirir | H/L | Tu | Kidney problems | F | Decoction | Oral |  | Colchicaceae |
|  |  |  |  |  |  | Abdominal pain, coughs, fever & malaria | F | Decoction/  Maceration | Oral |  |  |
|  |  |  |  |  |  | Venereal diseases, wounds, head lice & parasitic skin diseases; relieve bruises & inflammation | F | Paste | Apply |  |  |
|  |  |  |  |  | R | Scalp area; kill all the fungus & stimulate the growth of new hair | D | Powder | Apply |  |  |
|  |  |  |  |  | Tu | Gonorrhea | D | Powder-Extract | Oral |  |  |
|  |  |  |  |  | L | Powdered leaves are applied to wounds and sores to kill germs and promote healing | D | Powder | Apply |  |  |
|  |  |  |  |  | WP | Antimalarial | F | Juice | Oral |  |  |
|  |  |  |  |  | L | Coughs, general pain & oedema of the scrotum | F | Decoction/  Liniment | Apply |  |  |
|  |  |  |  |  |  | Fainting | F | Juice | Drops |  |  |
|  |  |  |  |  |  | Asthma & rheumatism | F | Extract | Apply |  |  |
|  |  |  |  |  | WP | Wounds | D | Ash | Apply |  |  |
|  |  |  |  |  | L | Earaches & ear infections | D | Powder | Swab | Lime juice |  |
|  | *Juniperus procera Hochst. ex Endl.*  (MNCY- 173) | LC | Tarakwet | T | Tw | Intestinal worms | F | Powder-Infusion | Oral |  | Cupressaceae |
|  |  |  |  |  | L | Stomach aches, TB, bronchitis, pneumonia, ulcers; heal wounds, liver diseases & intestinal worms | F | Juice | Oral |  |  |
|  |  |  |  |  |  | Wounds | D | Powder | Apply |  |  |
|  |  |  |  |  |  | Fever | F | Extract | Bath |  |  |
|  |  |  |  |  | Re | The resin is used as a stimulant and is also applied to ulcers. | F | Resin | Oral/Apply |  |  |
|  |  |  |  |  | B | Birth-control agents & vaginal wash | F | Maceration-extract | Oral/Wash |  |  |
|  |  |  |  |  | R | Skin diseases like skin rash and eczema; respiratory tract diseases like asthma, common cold, cough, bronchitis, throat inflammation, pneumonia and TB; urinary tract inflammations, renal, gall bladder stones & rheumatism | F | Decoction | Oral |  |  |
|  | *Gymnanthemum auriculiferum*(Hiern) Isawumi  (MNCY- 41) | NE | Tebeng'wet | S | L | Applied to the affected part | F | Roasted-Poultice | Apply |  | Asteraceae |
|  |  |  |  |  |  | Fever | F | Juice | Oral |  |  |
|  |  |  |  |  |  | Spots on the body or for a person feeling hot and liable to vomit | F | Extract | Oral |  |  |
|  |  |  |  |  | R | Malaria, fever & stomachache | F | Infusion | Oral |  |  |
|  |  |  |  |  | L | Expel placenta | F | Infusion | Oral |  |  |
|  |  |  |  |  | L | Eye infections | F | Extract | Oral |  |  |
|  |  |  |  |  |  | Headaches | F | Extract | Snuff |  |  |
|  |  |  |  |  |  | Blood clotting & wound healing | F | Extract | Apply |  |  |
|  | *Croton macrostachyu*s Del.  (MNCY- 136) | LC | Tebeswet | T | R | Purgative | F | Decoction | Oral |  | Euphorbiaceae |
|  |  |  |  |  | B/L | Epilepsy, insomnia & typhoid | F | Decoction | Oral |  |  |
|  |  |  |  |  | B/L/R | Backache, bleeding, cancer, colds, cough, diarrhoea, dysmenorrhea, malaria, measles, obesity, pneumonia, ringworm, skin diseases, typhoid, warts & wounds | F | Decoction | Oral |  |  |
|  |  |  |  |  | B/R | Headache, stomach ache & worms | F | Decoction | Oral |  |  |
|  |  |  |  |  | B/L/R/Tw | Abdominal pain, bleeding, bloat, colic, constipation, epilepsy, fever, ringworm, scabies, skin diseases, warts & wounds | F | Decoction | Oral |  |  |
|  |  |  |  |  | L | Constipation, coughs, dandruff, diarrhoea, dysentery, dysmenorrhea, eczema & epilepsy | F | Decoction | Oral |  |  |
|  |  |  |  |  | R | Anthelmintic for tapeworm, diarrhoea in children, ascariasis, backache, cancer, skin diseases, rectum prolapse stomachache & purgative | F | Decoction | Oral |  |  |
|  |  |  |  |  | L | Coughs & oral thrush | D | Ash | Leak |  |  |
|  |  |  |  |  | L/Sh | Juice from a fresh leaf/young stem is applied on fresh wounds to hasten blood clotting. | F | Extract | Apply |  |  |
|  |  |  |  |  | R | Juice from boiled roots is drunk for malaria or venereal diseases | F | Juice | Oral |  |  |
|  |  |  |  |  | SB | Skin rashes | F | Decoction | Bath |  |  |
|  |  |  |  |  | R | Cancers | F | Decoction | Oral |  |  |
|  |  |  |  |  | B | Dysmenorrhea & cancer | F | Decoction | Oral |  |  |
|  |  |  |  |  | L | Chronic wounds that probably are maggot infested | F | Juice | Apply |  |  |
|  |  |  |  |  | L | Allergies | F | Extract | Rub-Apply |  |  |
|  | *Oldeania alpina*(K.Schum.) Stapleton  (MNCY- 119) | NE | Tegat | S | Sh | Labour, expulsion of the placenta by inducing uterine contractions. & decrease cholesterol | F | Decoction | Oral |  | Poaceae |
|  |  |  |  |  |  | Cleaning wounds & healing infections |  | Poultice | Wash/Apply |  |  |
|  |  |  |  |  | L | Coughing and phlegm, fainting, spasms, controlling vomiting &fever | F | Decoction | Oral |  |  |
|  |  |  |  |  | Sh | Respiratory disorders. | F | Decoction | Oral | Honey |  |
|  | *Acanthus eminens*  C.B.Clarke  (MNCY- 8) | NE | Tegeltet | T | WP | Spleen diseases “Kipkarasonit” & joint pains | D | Ash | Leak |  | Acanthaceae |
|  |  |  |  |  | L | Wounds | F | Infusion | Oral |  |  |
|  |  |  |  |  | L/R | High blood pressure, stomachaches, malaria, constipation, splenomegaly & liver problems | F | Decoction | Oral |  |  |
|  | *Ekebergia capensis* Sparrm.  (MNCY- 117) | LC | Teldet | T | L | Vermifuge | F | Decoction/Infusion | Oral |  | Meliaceae |
|  |  |  |  |  |  | Treatment of headaches, fevers, coughs & skin complaints | F | Maceration | Oral/Apply |  |  |
|  |  |  |  |  |  | Intestinal worms | F | Decoction | Oral |  |  |
|  |  |  |  |  |  | Relieve cold & fever | F | Decoction | Oral | Salt |  |
|  |  |  |  |  | B | Gastritis, dysentery, heartburn, epilepsy & gonorrhoea | F | Decoction/Infusio | Oral |  |  |
|  |  |  |  |  | St | Skin complications including abscesses, ulcers, boils, scabies, acne, pimples & skin itching | F | Paste | Topical |  |  |
|  |  |  |  |  |  | Headache, cold & sinusitis | F | Powder | Snuff |  |  |
|  |  |  |  |  |  | Facilitate childbirth | F | Decoction | Oral |  |  |
|  |  |  |  |  | R | Kidney problems, dysentery, headaches, heartburn, chronic coughs & other respiratory complications | F | Decoction | Oral |  |  |
|  |  |  |  |  |  | The pulverized roots are also sniffed as a means of relieving headache, colds and blocked nose. | F | Pulverized-Extract | Oral |  |  |
|  |  |  |  |  |  | Headaches, heartburn & chronic coughs | F | Decoction | Oral |  |  |
|  |  |  |  |  | B | Waist pain | D | Powder | Apply-topical |  |  |
|  |  |  |  |  |  | Stomach upsets, constipation, blood purifier & cancer | F | Decoction | Oral |  |  |
|  | *Prunus africana* (Hook.f) Scweinf.  (MNCY- 157) | VU | Tendwet | T | B/L | Breast and prostate cancer, typhoid, animal protein allergy, STDs, arthritis, gout, blood purifier/ cleanse, appetizer, stomach problems, constipation, malaria, hyperacidity, ulcers, fibroids, joints & renew memory | F | Decoction | Oral |  | Rosaceae |
|  |  |  |  |  | L | Fever | F | Paste | Inhalation |  |  |
|  |  |  |  |  | B | Stomachache | F | Extract | Oral |  |  |
|  |  |  |  |  | L | Leaf infusion is drunk to improve appetite | F | Infusion | Oral |  |  |
|  |  |  |  |  | R | Root decoction is used in urine problems like retention and cancer | F | Decoction | Oral |  |  |
|  |  |  |  |  | L | Dress wounds | D | Powder | Apply |  |  |
|  |  |  |  |  |  | Relieve fever & in increasing appetite | F | Infusion | Oral |  |  |
|  |  |  |  |  | B | Chest pains | F | Infusion | Oral |  |  |
|  | *Calpurnia aurea (Aiton) Benth. subsp.aurea*  (MNCY- 123) | NE | Tetee | T | R | STI’s, coughs, syphilis & urinary problems | F | Decoction | Oral |  | Fabaceae |
|  |  |  |  |  | L | Destroy maggots in wounds & allergic rashes from caterpillars | F | Sap | Apply |  |  |
|  |  |  |  |  |  | Destroy lice & relieve itches | L | Powder | Apply |  |  |
|  |  |  |  |  | Se | Abscesses and wounds. | D | Powder | Apply |  |  |
|  |  |  |  |  | D/Se | Stomach complaints, headache, eye diseases, amoebic dysentery, tapeworm & scabies | D | Powder-Extract | Oral |  |  |
|  |  |  |  |  | B | Syphilis, malaria, diabetes, hypertension, diabetes, diarrhoea, eye infection, fungal diseases stomachache, vomiting, headaches and eye diseases &swellings | F | Decoction | Oral |  |  |
|  | *Pteridium aquilinum* (L.) Kuhn  (MNCY- 101) | LC | Tilalwet | H | R | Stomach cramps, chest pains, internal bleeding, diarrhoea, colds expel worms & cancer | F | Teas-Infusion | Oral |  | Dennstaedtiaceae |
|  |  |  |  |  | L | Arthritis | F | Steam | Bath |  |  |
|  |  |  |  |  | R | Sores, burns & caked breasts | F | Poultice | Apply |  |  |
|  |  |  |  |  |  | Rheumatism, weak blood, uterine prolapse, suffering after birth, caked breast, weakness and headaches, blood production booster especially after menses or childbirth, diarrhea, nausea and vomiting, infections & stomach cramps | F | Decoction | Oral |  |  |
|  |  |  |  |  | Sh | The young shoots are diuretic, coolant & vermifuge | F | Decoction | Oral |  |  |
|  |  |  |  |  | WP | Tuberculosis | F | Decoction | Oral |  |  |
|  |  |  |  |  | L | Sores & bind broken bones |  | Poultice-Fronds | Apply |  |  |
|  |  |  |  |  | R | Stop vomiting, antiseptic, appetizer & tonic | F | Decoction | Oral |  |  |
|  | *Vachellia hockii*(De Wild.) Seigler & Ebinger  (MNCY- 17) | NE | Tilatiliet | T | R | Abdominal pains, general body pain, stomach troubles, spinal complains, back pain, swellings & urinal problems | F | Decoction | Oral |  | Fabaceae |
|  |  |  |  |  | L | Children skeletal problems | F | Extract/Decoction | Oral |  |  |
|  |  |  |  |  | R | Allergy to eat meat normally | F | Infusion | Oral | Meat |  |
|  |  |  |  |  | L/Bd | Abscess |  |  | Apply/Tied | Ghee |  |
|  |  |  |  |  | B | Inflammation, oedema, gout, malaria, abdominal pain, cancer & bloody diarrhoea | F | Decoction | Oral |  |  |
|  |  |  |  |  | B/R | Digestive problems, bacterial infection, malaria, abscess, gastritis, abdominal pains, stomachache & TB | F | Decoction | Oral |  |  |
|  |  |  |  |  | R | Snake bite. | F | Decoction | Oral | Sweet potato extract |  |
|  | *Rhynchosia hirta* (Andrews) Meikle & Verdc  (MNCY- 244) | NE | Tilyamook | T | L | Induce uterine contractions, aid the birth process, labour & abortion. | F | Decoction | Oral |  | Fabaceae |
|  |  |  |  |  | R | Relieve gastric problems, flatulence, constipation, purgative, headache nausea & vomiting | F | Decoction | Oral |  |  |
|  | *Rhoicissus tridentata* (L.f.) Wild & R.B.Drumm.  (MNCY- 188) | LC | Torotwet | S/C | Tu | Infertility, stomach, kidney and bladder complaints & dysmenorrhea; tone the uterus and also to facilitate delivery & expel a retained placenta | F | Decoction | Oral |  | Vitaceae |
|  |  |  |  |  | R | Ease indigestion | F | Chew-Juice | Oral |  |  |
|  |  |  |  |  |  | Abdominal menstruation pains | F | Decoction | Oral |  |  |
|  |  |  |  |  |  | Swollen glands in the the pelvis | F | Heat-Pieces | Apply |  |  |
|  |  |  |  |  | St | Cuts & sores for healing and anaesthetizing | F | Sap | Apply |  |  |
|  |  |  |  |  | R | Hypertension & blood purification | F | Decoction | Oral |  |  |
|  |  |  |  |  | L | Relief coughs | F | Chew-Extract | Oral |  |  |
|  |  |  |  |  | Tu | Wounds | F | Vortex-Gum | apply |  |  |
|  |  |  |  |  |  | Diarrhoea | F | Juice | Oral |  |  |
|  | *Nicotiana rustica* L. (MNCY- 87) | NE | Tumotet | H | L | Backache, toothache, low back pain, gastritis, ulcers & wounds | F | Extract | Oral |  | Solanaceae |
|  |  |  |  |  |  | Sprains & bruises | F | Decoction | Apply-Rub |  |  |
|  |  |  |  |  |  | Boils, infected wounds, rheumatic swelling & skin disorders |  | Poultice | Apply |  |  |
|  |  |  |  |  |  | Baldness | F | Paste | Apply | Oil |  |
|  |  |  |  |  |  | Indisposition, sedative & snakebites | F | Juice | Oral |  |  |
|  |  |  |  |  |  | Pulmonary ailments | D | Powder | Snuff |  |  |
|  |  |  |  |  | L/St | Cold | L | Juice | Inhalation |  |  |
|  |  |  |  |  | L | Muscle relaxation in dislocation, hernia, inflammation of the testacles, low back pain, rheumatism & gout | F | Decoction | Apply |  |  |
|  |  |  |  |  |  | Cuts, wounds & insect bites | F | Paste | Apply |  |  |
|  |  |  |  |  |  | Rheumatic swelling, skin diseases & scorpion stings | F | Poultice | Wash |  |  |
|  | *Chaetacmi aristata* Planch.  (MNCY- 174) | LC | Tungururwet | S/ST | L | The young fronds are used as a laxative. | F | Extract | Oral |  | Ulmaceae |
|  |  |  |  |  |  | Meningitis & encephalitis | F | Sap | Oral | Other plants |  |
|  |  |  |  |  | R | “Kiptumarit” characteristically Bell's palsy diseases, epilepsy & hiccups | F | Decoction | Oral |  |  |
|  |  |  |  |  | B | False labour contraction before term | F | Decoction | Oral |  |  |
|  | *Clutia abysinicca Jaub.& Spach*  (MNCY- 33) | NE | Turmanyat | H/S | R | Enlarged spleen, kidney problems, difficulty of urination, headache, stomachache & malaria. The root | F | Decoction | Oral | Soup | Peraceae |
|  |  |  |  |  | Rb | Laxative | F | Decoction | Oral |  |  |
|  |  |  |  |  | R | Relieve colic pain in infants, erectile dysfunction, malaria, treat influenza &for habitual abortion | F | Decoction | Oral |  |  |
|  |  |  |  |  | R/L | Venereal, skin diseases, bloody diarrhoea, chest problems, fertility issues & cancer | F | Decoction | Oral |  |  |
|  |  |  |  |  | R | Roundworm infections, clean kidneys & stomachaches | F | Infusion | Oral |  |  |
|  |  |  |  |  | L | Gastritis, herpes zoster, ring worm, spider bite, internal parasite infection, treat dysentery, toothache, eczema, rheumatism, kill ectoparasites & hypertension | F | Extract | Oral |  |  |
|  |  |  |  |  |  | Toothache | F | Paste | Hold on tooth |  |  |
|  |  |  |  |  |  | Dizziness, ascariasis, for habitual miscarriage, convulsions, enlarged spleen & influenza | D | Powder-Infusion | Oral | Other plants |  |
|  |  |  |  |  |  | Oral thrush | D | Ash | Apply | Ghee |  |
|  |  |  |  |  |  | Malaria, headaches & stomach problems | F | Decoction | Oral |  |  |
|  |  |  |  |  |  | Gonorrhea | D | Enema | Apply |  |  |
|  |  |  |  |  | R | Headache | F | Decoction | Rub |  |  |
|  |  |  |  |  |  | Circulatory problems | F | Decoction | Oral |  |  |
|  |  |  |  |  | L | Caterpillar attack | F | Juice | Apply |  |  |
|  |  |  |  |  | R/L | Cancer, influenza, coughs and colds; fevers, malaria; liver problems, stomachaches, indigestion; headaches; intestinal worms & tonic for pregnant women | F | Decoction | Oral |  |  |
|  | *Euclea divinorum* Hiern  (MNCY- 37) | LC | Usuet | S/ST | R | Deworming | F | Chew hot-Extract | Oral |  | Ebenaceae |
|  |  |  |  |  | R | Maceration is drunk to treat abdominal problems & expel intestinal worms | F | Maceration-Extract | Oral |  |  |
|  |  |  |  |  | L | Stimulant for the uterus -induce the process of childbirth or to induce an abortion; sciatic pain, coughs, whooping cough & headaches | D | Powder-Infusion/  Decoction | Oral |  |  |
|  |  |  |  |  |  | Shock | F | Decoction | Oral |  |  |
|  |  |  |  |  |  | Diarrhea in children, whilst a | F | Sap-Enema | Apply |  |  |
|  |  |  |  |  |  | Malaria | F | Steam | Inhalation |  |  |
|  |  |  |  |  | R | Genital and oral disease, diarrhoea, cancer & dermal ailments | F | Decoction | Oral |  |  |
|  |  |  |  |  | R | Induce or augment labour & urine retention | F | Infusion | Oral |  |  |
|  |  |  |  |  | Fr | Fruits are chewed to treat abdominal upsets, skin, kidney and respiratory disorders. | F | Extract | Oral |  |  |
|  |  |  |  |  | L | Malaria, leprosy “Mogondosaik”, gonorrhea, syphilis & tapeworm | F | Decoction | Oral |  |  |
|  |  |  |  |  | Tw | Oral care where as the bark infusion | F | Chew-Extract | Oral |  |  |
|  |  |  |  |  |  | Appetizer | F | Infusion | Oral |  |  |
