# Supplementary material for: Ethnobotanical study of medicinal plants used by the people of Mosop, Nandi County in Kenya
Source: Front Pharmacol. 2024 Jan 19;14:1328903. doi: 10.3389/fphar.2023.1328903 (PMC10834697; doi:10.3389/fphar.2023.1328903)
Supplement: Supplementary file 3 [file Table2.DOCX]

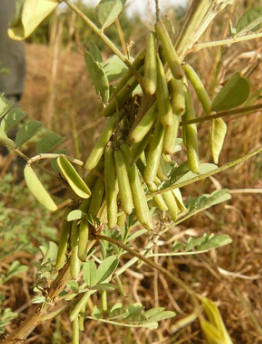
*.*
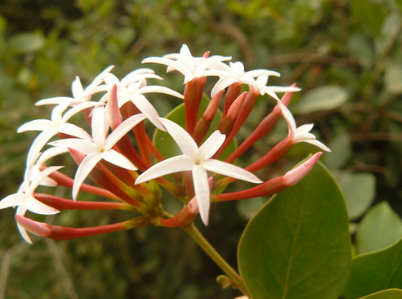

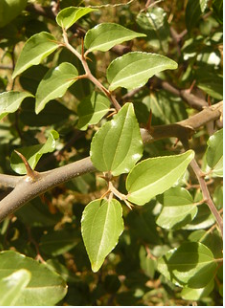


*Indigofera arrecta* Hochst. ex A.Rich *Carissa spinarum*L. *Ziziphus mucronata*Willd.


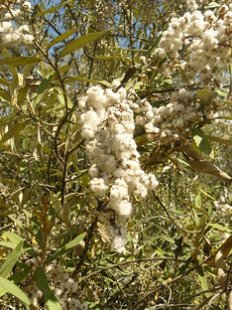

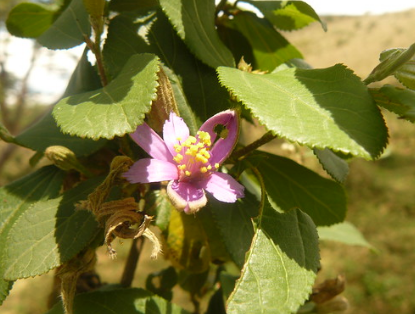

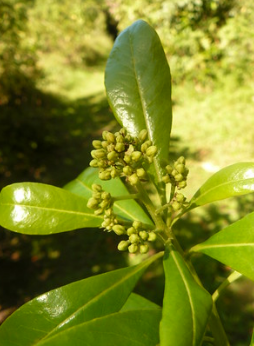


*Tarchonanthus camphoratus* L. *Grewia similis* K.Schum. *Pittosporum viridiflorum* Sims


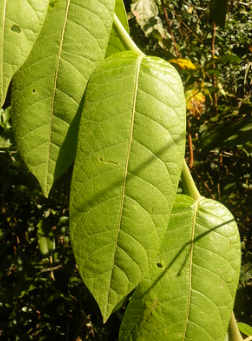

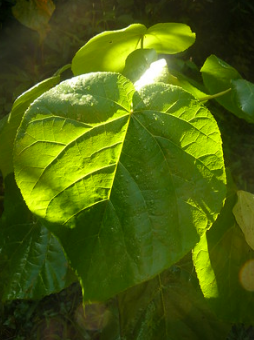

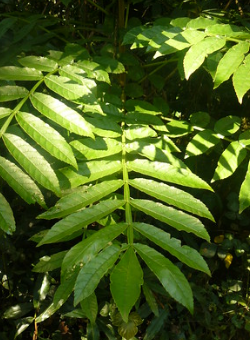


*Polyscias fulva* (Hiern) Harms *Croton macrostachyu*s Del. *Bersama abyssinica* Fresen.


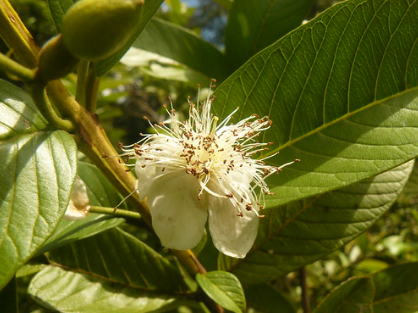

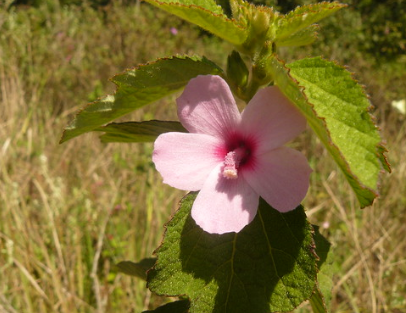

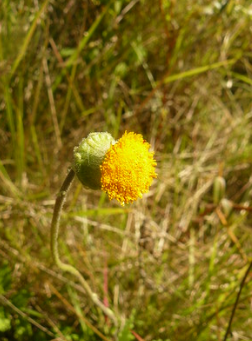


*Psidium guajava* L.  *Urena lobata* L.  *Crassocephalum vitellinum*S.Moore


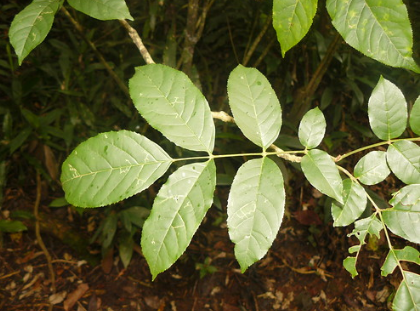

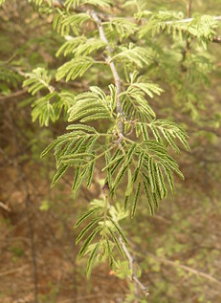

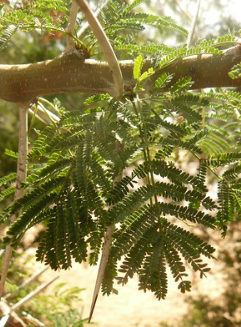


*Markhamia lutea* (Benth.) K. Schum *Senegalia senegal* (L.) Britton *Vachellia elatior*(Brenan) Kyal. & Boatwr.


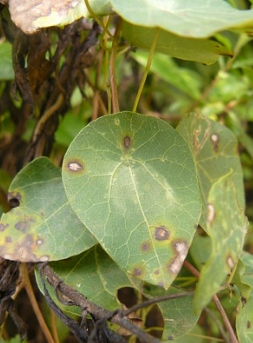

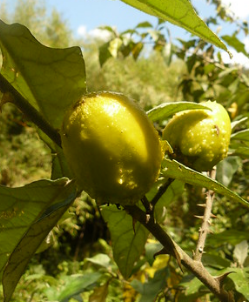

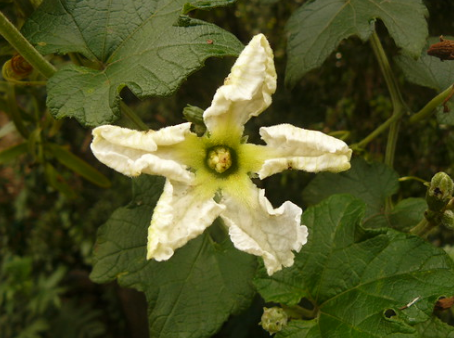


*Stephania abyssinica* Walp. *Solanum aculeastrum* Dunal *Lagenaria abyssinica* (Hook.f) C.Jeffrey


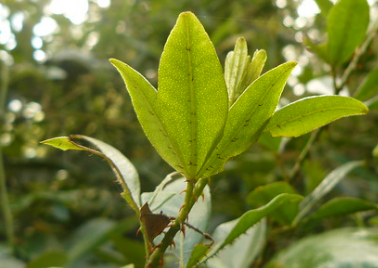

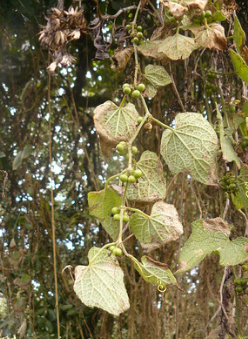

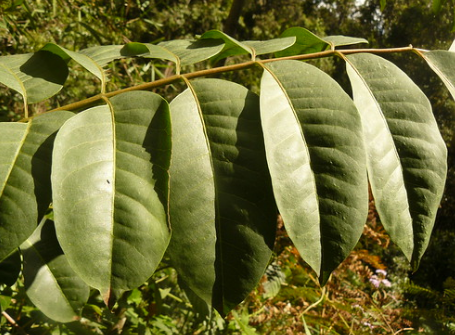


*Zanthoxylum asiaticum*(L.) *Zehneria scabra* Sond. *Ekebergia capensis* Sparrm.

Appelhans, Groppo & J.Wen


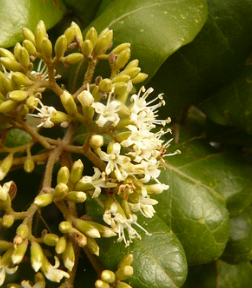

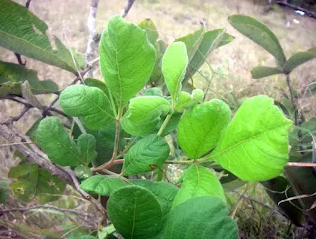

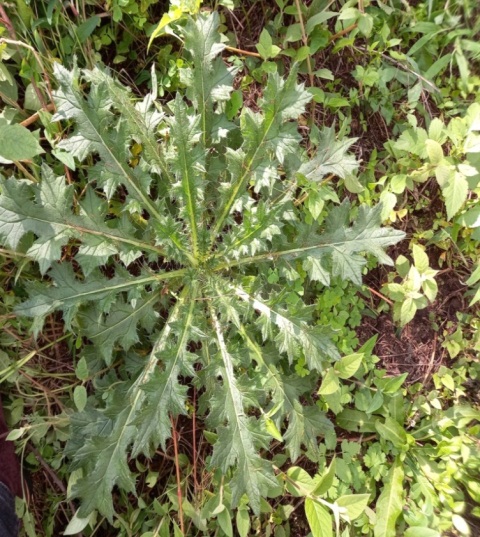


*Nuxia congesta*R.Br. *Searsia pyroides*(Burch.) Moffett *Carduus schimperi*Sch.Bip.


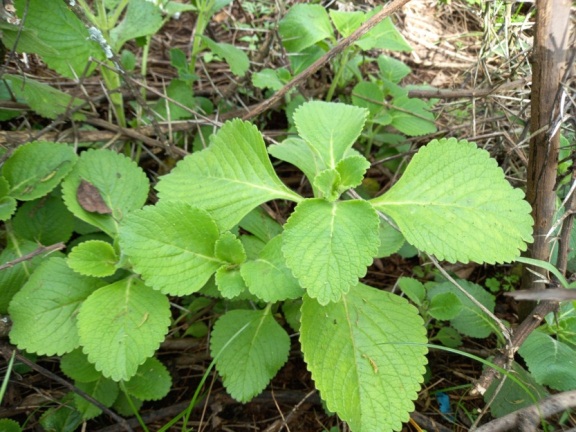

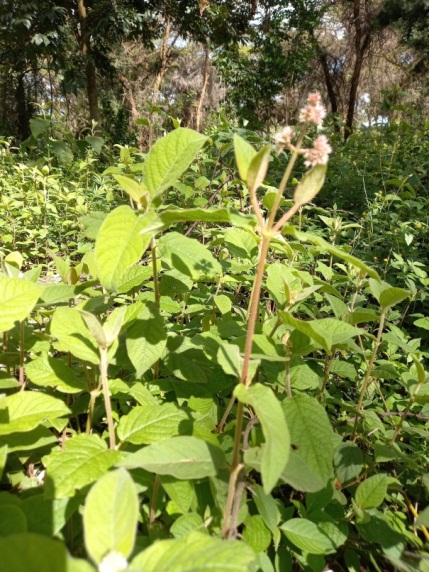

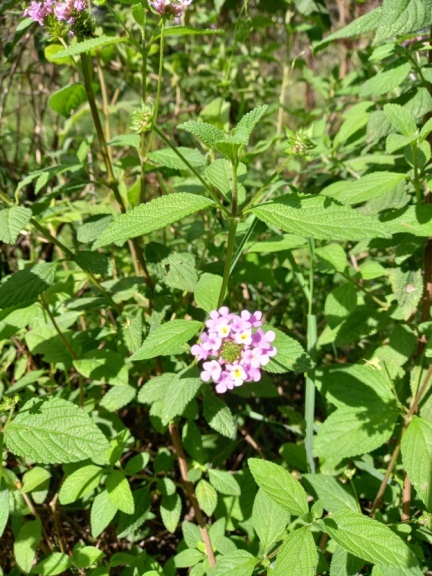


*Coleus barbatus*(Andrews) *Cyathula tomentosa*(Roth) Moq. *Lantana trifolia* L.

Benth. ex G.Don


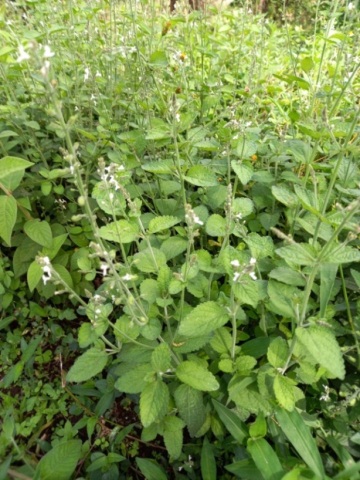

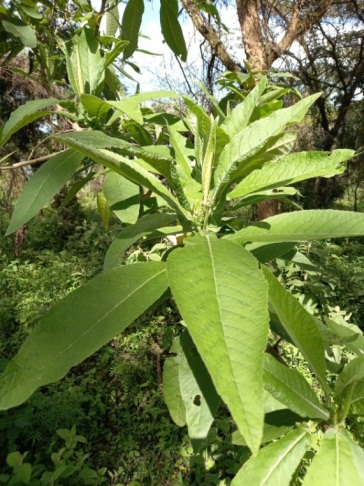

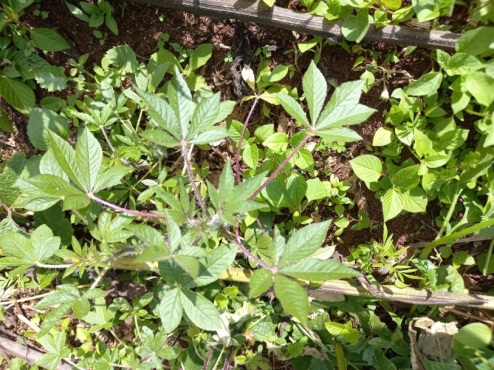


*Fuerstia africana* T.C.E.Fr *Gymnanthemum auriculiferum  Cleome gynandra*(L.) Briq.

(Hiern) Isawumi


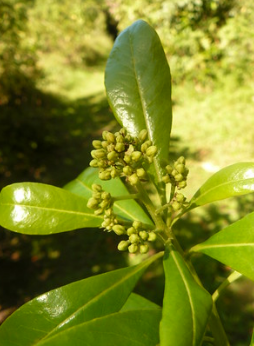

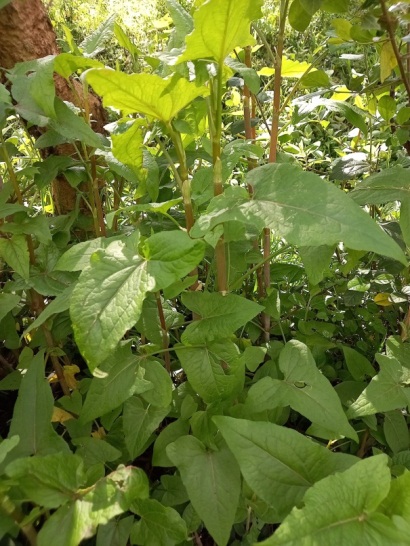

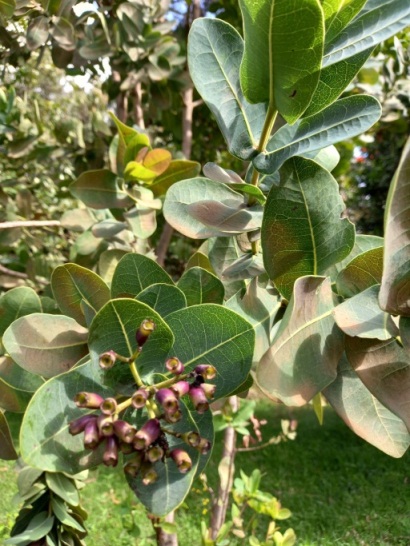


# Pittosporum viridiflorum Rumex abyssinicus Jacq. Syzygium cordatum Hochst.


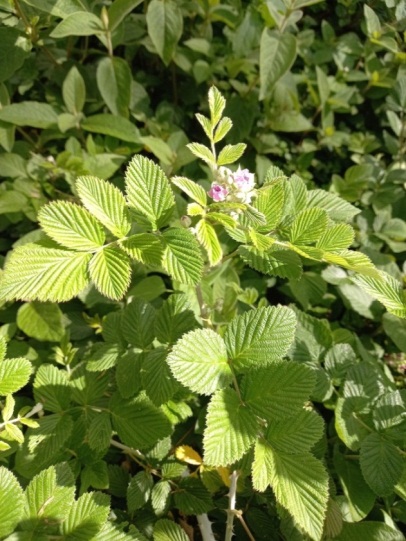

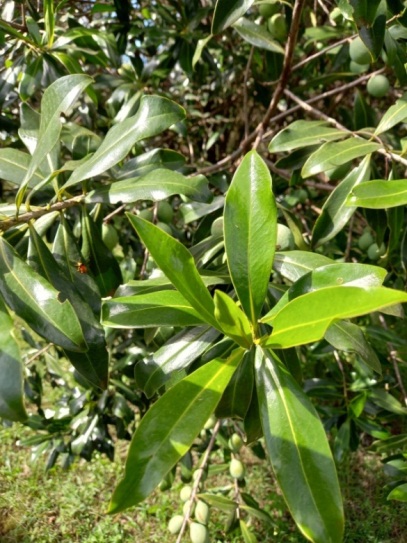

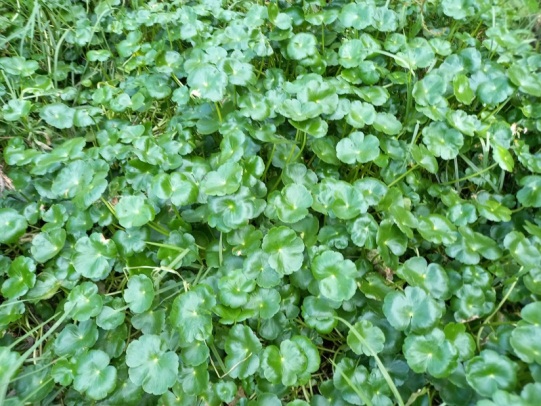


*Rubus pinnatu*s Willd. *Warburgia ugandensis* Sprague  *Dichondra micrantha* Urb.


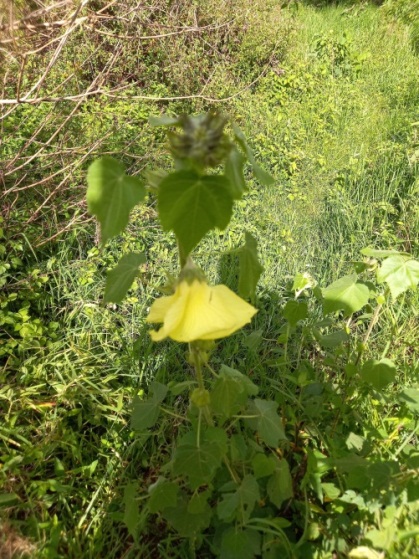

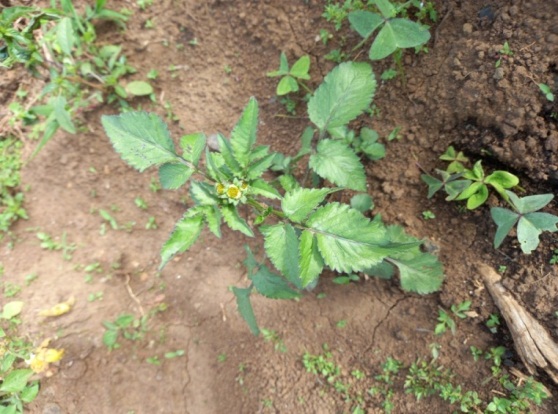

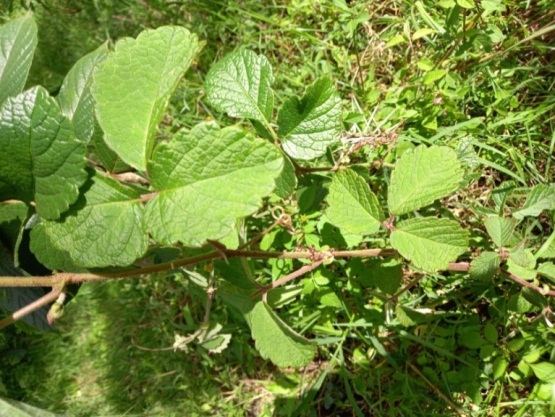


*Triumfetta macrophylla* K.Schum. *Bidens pilosa* L. *Cyphostemma orondo* (Gilg & M.Brandt) Desc.


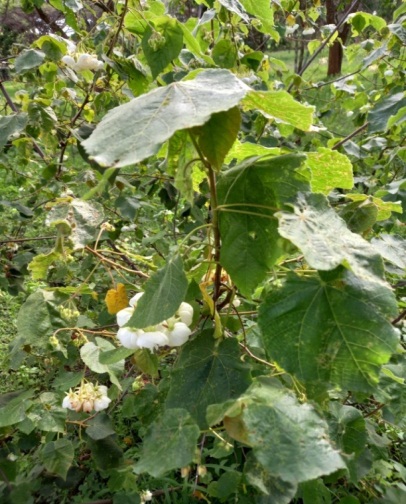

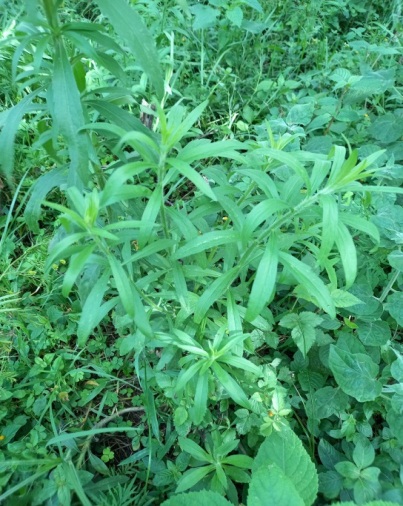

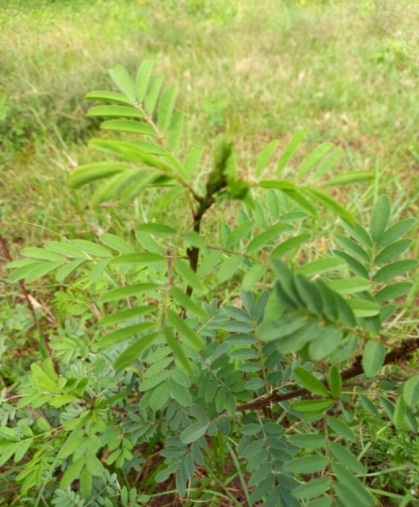


*Dombeya torrida* (J.F.Gmel.) Bamps *Erigeron canadensis* L. *Indigofera arrecta* Hochst. ex A.Rich.


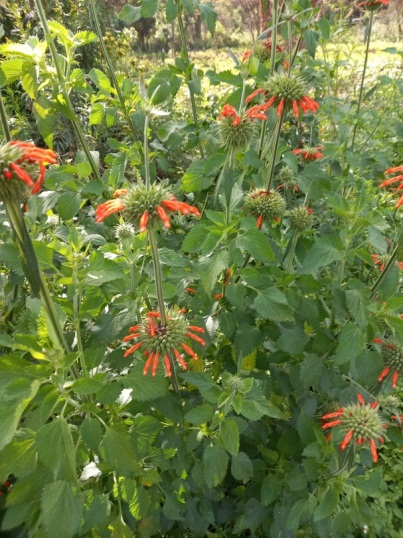

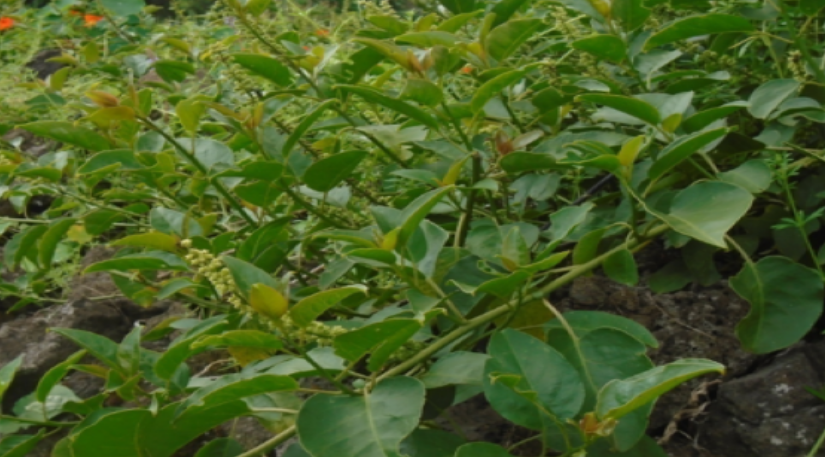

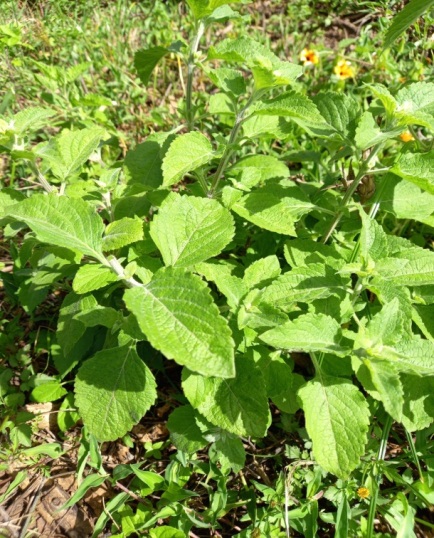


*Leonotis nepetifolia* (L.) R.Br. *Phytolacca dodecandra* L'Hér. *Ocimum gratissimum* L.


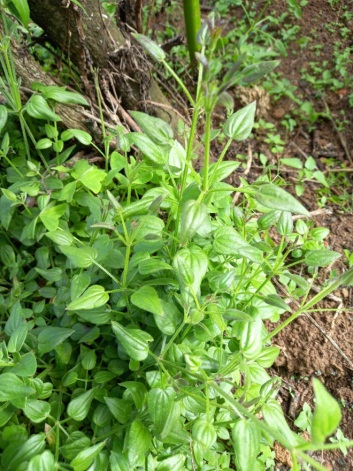

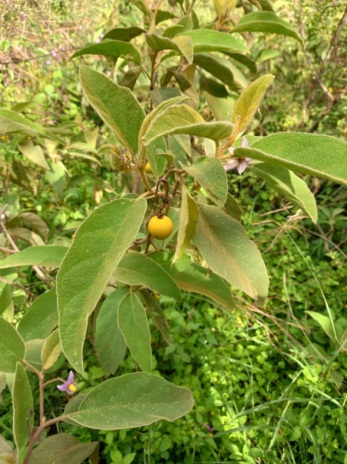

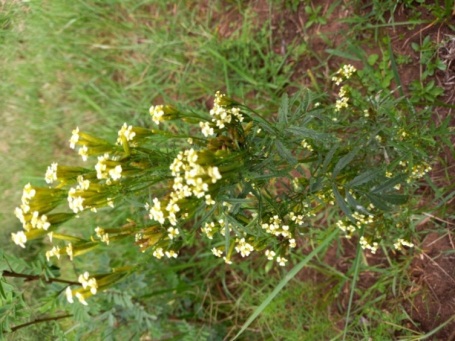


*Rubia cordifolia* L. *Solanum incanum* L *Tagetes minuta* L.


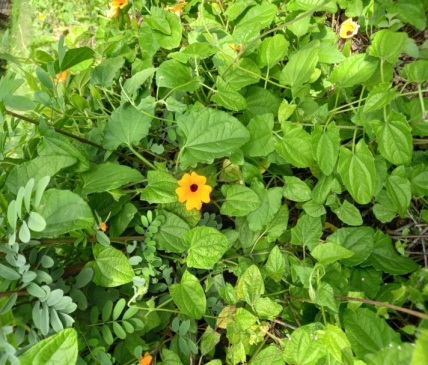

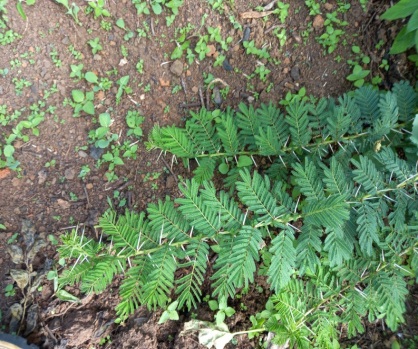

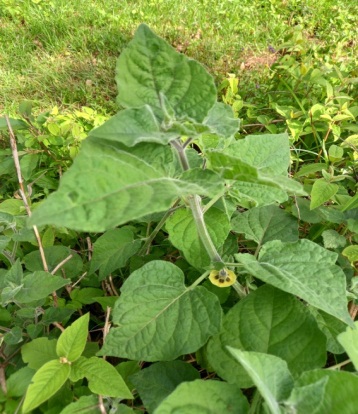


*Thunbergia alata*  *Vachellia hockii* (De Wild.) *Physalis peruviana* L.

Bojer ex Sims Seigler & Ebinger

#
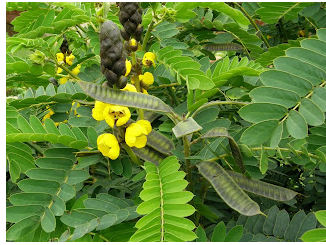

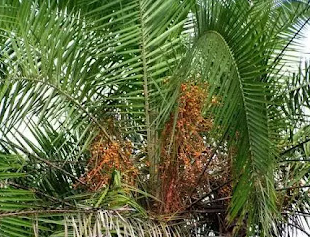

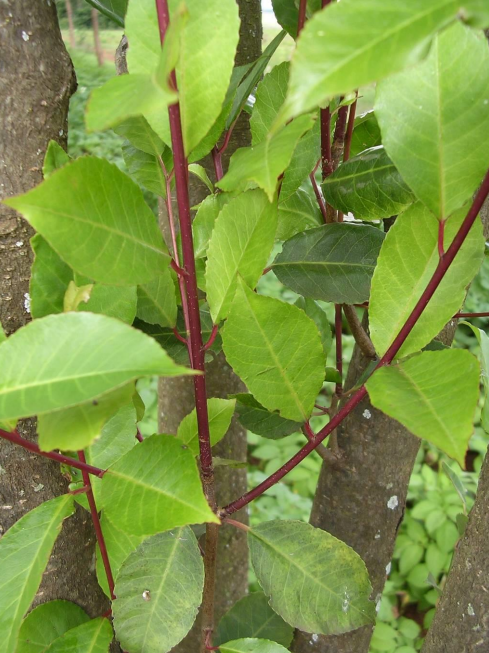


# Senna didymobotrya (Fresen.) Phoenix reclinata Jacq. Prunus africana (Hook.fil.) Kalkman

H.S.Irwin & Barneby
