# Supplementary material for: Ethnobotanical study of medicinal plants used by the people of Mosop, Nandi County in Kenya
Source: Front Pharmacol. 2024 Jan 19;14:1328903. doi: 10.3389/fphar.2023.1328903 (PMC10834697; doi:10.3389/fphar.2023.1328903)
Supplement: Supplementary file 4 [file Table4.DOCX]

**Table S2**: Supplementary table S2 for quantitative indices of medicinal plant diversity

|  | **Scientific name** | **FoC** | **UC** | **UV** | **RFC** |
| --- | --- | --- | --- | --- | --- |
|  | *Clerodendrum myricoides*R.Br. & Vatke | 93.14 | 96 | 5.70 | 0.94 |
|  | *Lantana trifolia* L. | 100 | 102 | 17.90 | 1.00 |
|  | *Trichocladus ellipticus Eckl. & Zeyh.* | 91.18 | 83 | 3.46 | 0.81 |
|  | *Afrocarpus falcatus*(Thunb.) C.N.Page | 96.08 | 100 | 13.37 | 0.98 |
|  | *Phytolacca dodecandra*L'Hér. | 98.04 | 99 | 19.53 | 0.97 |
|  | *Terminalia schimperiana*Hochst. ex Engl. & Diels | 99.02 | 100 | 20.36 | 0.98 |
|  | *Crassocephalum vitellinum*S.Moore | 96.08 | 97 | 10.45 | 0.95 |
|  | *Lactuca glandulifera*Hook.f. | 91.18 | 89 | 4.16 | 0.87 |
|  | *Sonchus asper*(L.) Hill | 95.10 | 91 | 8.71 | 0.89 |
|  | *Eucalyptus globulus* Labill. | 99.02 | 101 | 26.85 | 0.99 |
|  | *Nicoteba betonica*(L.) Lindau | 99.02 | 98 | 23.51 | 0.96 |
|  | *Vachellia nilotica* subsp. *tomentosa*(Benth.) Kyal. & Boatwr. | 99.02 | 100 | 36.54 | 0.98 |
|  | *Ajuga integrifolia*Buch.-Ham. ex D.Don | 97.06 | 100 | 18.60 | 0.98 |
|  | *Bridelia micrantha*(Hochst.) Baill. | 88.24 | 98 | 4.84 | 0.96 |
|  | *Emilia discifolia*(Oliv.) C.Jeffrey | 95.10 | 93 | 9.16 | 0.91 |
|  | *Ziziphus mucronata*Willd. | 97.06 | 102 | 23.25 | 1.00 |
|  | *Pavonia kilimandscharica Gürke* | 92.16 | 97 | 11.05 | 0.95 |
|  | *Tragia brevipes*Pax | 95.10 | 102 | 12.75 | 1.00 |
|  | *Allophylus racemosus*Sw. | 99.02 | 97 | 7.90 | 0.95 |
|  | *Leucas deflexa*Hook.f. | 97.06 | 95 | 10.31 | 0.93 |
|  | *Leucas martinicensis*(Jacq.) R.Br. | 96.08 | 97 | 8.75 | 0.95 |
|  | *Urena lobata* L. | 100 | 100 | 23.55 | 0.98 |
|  | *Mesosphaerum pectinatum*(L.) Kuntze | 96.08 | 93 | 7.75 | 0.91 |
|  | *Justicia ladanoides*Lam. | 91.18 | 96 | 7.34 | 0.94 |
|  | *Dyschoriste hildebrandtii*Lindau ex C.B.Clarke | 91.18 | 96 | 8.84 | 0.94 |
|  | *Dyschoriste radicans* (Hochst. ex A.Rich.) Nees | 94.12 | 100 | 11.47 | 0.98 |
|  | *Spermacoce princeae* (K.Schum.) Verdc. | 90.20 | 98 | 9.21 | 0.96 |
|  | *Spermacoce senensis*(Klotzsch) Hiern | 90.20 | 98 | 8.09 | 0.96 |
|  | *Markhamia zanzibarica*(Bojer ex DC.) K.Schum. | 92.16 | 92 | 7.67 | 0.90 |
|  | *Casearia battiscombei*R.E.Fr. | 92.16 | 89 | 5.08 | 0.87 |
|  | *Barleria grandicalyx* Lindau | 96.08 | 98 | 14.25 | 0.96 |
|  | *Ocimum kilimandscharicum Gürke* | 96.08 | 93 | 7.93 | 0.91 |
|  | *Chrysanthemum americanum*(L.) Vatke ex Weberl. & Lagos | 93.14 | 92 | 5.75 | 0.90 |
|  | *Noronhia mildbraedii*(Gilg & G.Schellenb.) Hong-Wa & Besnard | 98.04 | 99 | 12.29 | 0.97 |
|  | *Clutia abyssinica Jaub. & Spach* | 99.02 | 98 | 17.15 | 0.96 |
|  | *Amaranthus caudatus* L. | 98.04 | 96 | 15.58 | 0.94 |
|  | *Centella Coriacea* Nannf. | 91.18 | 95 | 5.70 | 0.93 |
|  | *Ozoroa insignis* Delile | 95.10 | 98 | 9.61 | 0.96 |
|  | *Senegalia senegal* (L.) Britton | 98.04 | 100 | 18.99 | 0.98 |
|  | *Solanum betaceum* Cav. | 93.14 | 100 | 15.47 | 0.98 |
|  | *Solanecio mannii* (Hoof.f.) C.Jeffrey | 94.12 | 96 | 10.93 | 0.94 |
|  | *Coptosperma graveolens (S.Moore) Degreef* | 96.08 | 97 | 9.45 | 0.95 |
|  | *Seriphium kilimandscharicum*(O.Hoffm.) Koek. | 93.14 | 99 | 9.72 | 0.97 |
|  | *Culcasia falcifolia* Engl. | 77.45 | 82 | 2.56 | 0.80 |
|  | *Eschenbachia subscaposa*(O.Hoffm.) G.L.Nesom | 97.06 | 101 | 19.45 | 0.99 |
|  | *Pittosporum viridiflorum* Sims | 93.14 | 98 | 15.34 | 0.96 |
|  | *Unknown* | 97.06 | 100 | 12.94 | 0.98 |
|  | *Trimeria grandifolia* (Hochst.) Warb | 90.20 | 87 | 3.66 | 0.85 |
|  | *Adenia cissampeloides*(Planch. ex Hook.) Harms | 94.12 | 100 | 8.73 | 0.98 |
|  | *Dichondra micrantha* Urb. | 91.18 | 92 | 7.51 | 0.90 |
|  | *Vachellia elatior*(Brenan) Kyal. & Boatwr. | 99.02 | 97 | 11.67 | 0.95 |
|  | *Tagetes minuta* L. | 99.02 | 100 | 12.54 | 0.98 |
|  | *Micromeria biflora* (Buch. - Ham. ex D.Don) Benth. | 94.12 | 91 | 8.03 | 0.89 |
|  | *Micromeria* Benth. | 94.12 | 91 | 11.91 | 0.89 |
|  | *Aspilia pluriseta* Schweinf. ex Engl. | 100 | 101 | 22.15 | 0.99 |
|  | *Galinsoga parviflora Cav.* | 92.16 | 97 | 9.34 | 0.95 |
|  | *Hoslundia opposita* Vahl | 97.06 | 89 | 4.56 | 0.87 |
|  | *Tylosema fassoglense*(Kotschy ex Schweinf.) Torre & Hillc. | 98.04 | 99 | 13.52 | 0.97 |
|  | *Momordica foetida* Schumach. | 82.35 | 83 | 2.79 | 0.81 |
|  | *Thunbergia alata* Bojer ex Sims | 97.06 | 98 | 19.71 | 0.96 |
|  | *Gymnosporia undata*(Thunb.) Szyszyl. | 99.02 | 98 | 16.73 | 0.96 |
|  | *Diospyros abyssinica* (Hiern) F.White | 87.25 | 93 | 4.42 | 0.91 |
|  | *Dolichopentas longiflora*(Oliv.) Kårehed & B.Bremer | 97.06 | 96 | 10.57 | 0.94 |
|  | *Typha latifolia*L. | 97.06 | 99 | 18.70 | 0.97 |
|  | *Grewia similis* K. Schum | 99.02 | 100 | 23.63 | 0.98 |
|  | *Asparagus racemosus* Willd. | 97.06 | 99 | 15.13 | 0.97 |
|  | *Erythrococca bongensis* Pax | 96.08 | 102 | 9.26 | 1.00 |
|  | *Achyranthes aspera* L. | 96.08 | 99 | 23.33 | 0.97 |
|  | *Hymenodictyon floribundum*(Hochst. & Steud.) B.L.Rob. | 99.02 | 98 | 12.48 | 0.96 |
|  | *Grewia similis* K.Schum. | 99.02 | 102 | 22.63 | 1.00 |
|  | *Nuxia congesta*R.Br. | 96.08 | 101 | 17.68 | 0.99 |
|  | *Lippia javanica* Spreng*.* | 96.08 | 101 | 15.44 | 0.99 |
|  | *Leonotis ocymifolia*var. *raineriana*(Vis.) Iwarsson | 94.12 | 98 | 14.60 | 0.96 |
|  | *Leonotis nepetifolia* (L.) R.Br. | 94.12 | 98 | 22.99 | 0.96 |
|  | *Olea europaea*subsp. *cuspidata*(Wall. & G.Don) Cif. | 95.10 | 98 | 15.04 | 0.96 |
|  | *Zanthoxylum chalybaeum*Engl. | 99.02 | 102 | 27.26 | 1.00 |
|  | *Ricinus communis* L. | 90.20 | 94 | 5.42 | 0.92 |
|  | *Coleus barbatus*(Andrews) Benth. ex G.Don | 100 | 101 | 31.35 | 0.99 |
|  | *Gynandropsis gynandra*(L.) Briq. | 96.08 | 98 | 21.41 | 0.96 |
|  | *Unknown* | 81.37 | 81 | 1.96 | 0.79 |
|  | *Olinia rochetiana* A.Juss. | 84.31 | 92 | 4.14 | 0.90 |
|  | *Schrebera alata*(Hochst.) Welw. | 99.02 | 100 | 18.71 | 0.98 |
|  | *Erythrina abyssinica*Lam. ex DC. | 93.14 | 96 | 9.09 | 0.94 |
|  | *Dovyalis abyssinica* (A.Rich.) Warb. | 97.06 | 99 | 21.90 | 0.97 |
|  | *Entada africana* Guill. & Perr. | 99.02 | 101 | 24.70 | 0.99 |
|  | *Tiliacora triandra* (Colebr.) Diels | 57.84 | 54 | 0.53 | 0.53 |
|  | *Cordia africana* Lam. | 95.10 | 97 | 9.95 | 0.95 |
|  | *Vachellia abyssinica* (Hochst. ex Benth.) Kyal. & Boatwr. | 93.14 | 96 | 8.39 | 0.94 |
|  | *Carduus schimperi*Sch.Bip. | 95.10 | 95 | 13.02 | 0.93 |
|  | *Entada abyssinica* Steudel ex A.Rich. | 98.04 | 102 | 27.15 | 1.00 |
|  | *Acokanthera schimperi*(A.DC.) Schweinf. | 91.18 | 94 | 4.47 | 0.92 |
|  | *Combretum pisoniiflorum*(Klotzsch) Engl. | 89.22 | 98 | 6.57 | 0.96 |
|  | *Sida schimperiana*Hochst. ex A.Rich. | 93.14 | 95 | 11.60 | 0.93 |
|  | *Vangueria apiculata* K.Schum. | 97.06 | 98 | 13.87 | 0.96 |
|  | *Piper umbellatum* L. | 97.06 | 99 | 24.32 | 0.97 |
|  | *Zanthoxylum usambarense* (Engl.) Kokwaro | 94.12 | 95 | 10.59 | 0.93 |
|  | *Erigeron canadensis* L. | 97.06 | 99 | 20.01 | 0.97 |
|  | *Combretum collinum* Fresen | 94.12 | 96 | 11.68 | 0.94 |
|  | *Maesa lanceolata* Forssk. | 89.22 | 82 | 2.67 | 0.80 |
|  | *Girardinia diversifolia* (Link) Friis | 91.18 | 92 | 5.42 | 0.90 |
|  | *Embelia schimperi* Vatke | 90.20 | 90 | 4.74 | 0.88 |
|  | *Brillantaisia vogeliana*Benth. | 86.27 | 91 | 3.59 | 0.89 |
|  | *Bersama abyssinica* Fresen. | 84.31 | 95 | 4.87 | 0.93 |
|  | *Lactuca macrophylla*(Willd.) A.Gray | 98.04 | 99 | 9.89 | 0.97 |
|  | *Vangueria infausta* Burch. | 99.02 | 101 | 36.29 | 0.99 |
|  | *Bidens pilosa* L. | 88.24 | 98 | 4.84 | 0.96 |
|  | *Zanthoxylum asiaticum*(L.) Appelhans, Groppo & J.Wen | 99.02 | 92 | 36.29 | 0.90 |
|  | *Crotalaria laburnifolia* L. | 97.06 | 101 | 32.75 | 0.99 |
|  | *Lannea schimperi* (A.Rich.)Engl. | 95.10 | 97 | 15.00 | 0.95 |
|  | *Scepocarpus hypselodendron*(Hochst. ex A.Rich.) T.Wells & A.K.Monro | 98.04 | 100 | 19.06 | 0.98 |
|  | *Sida cordifolia* L | 77.45 | 74 | 1.57 | 0.73 |
|  | *Aeschynomene schimperi* Hochst. ex A.Rich. | 97.06 | 100 | 23.47 | 0.98 |
|  | *Zanthoxylum chevalieri P.G.Waterman* | 94.12 | 99 | 11.03 | 0.97 |
|  | *Billieturnera helleri*(Rose ex A.Heller) Fryxell | 100 | 100 | 21.52 | 0.98 |
|  | *Rhamnus prinioides* L’Hér. | 96.08 | 97 | 12.77 | 0.95 |
|  | *Gymnosporia heterophylla*(Eckl. & Zeyh.) Loes. | 98.04 | 101 | 23.08 | 0.99 |
|  | *Croton dichogamus* Pax. | 97.06 | 98 | 17.83 | 0.96 |
|  | *Euphorbia candelabrum*Welw. | 99.02 | 100 | 22.67 | 0.98 |
|  | *Vepris nobilis*(Delile) Mziray | 96.08 | 99 | 11.10 | 0.97 |
|  | *Clutia pulchella*L. | 98.04 | 100 | 10.07 | 0.98 |
|  | *Kalanchoe crenata* (Andrews) Haw. | 95.10 | 92 | 7.63 | 0.90 |
|  | *Hoffmannanthus abbotianus*(O.Hoffm.) H.Rob., S.C.Keeley & Skvarla | 97.06 | 99 | 16.40 | 0.97 |
|  | *Cirsium vulgare* (Savi) Ten. | 95.10 | 95 | 5.86 | 0.93 |
|  | *Solanum incanum* L. | 90.20 | 97 | 6.69 | 0.95 |
|  | *Solanum nigriviolaceum*Bitter | 90.20 | 95 | 6.81 | 0.93 |
|  | *Solanum melongena*L. | 90.20 | 95 | 11.92 | 0.93 |
|  | *Solanum mauense* Bitter | 97.06 | 99 | 14.06 | 0.97 |
|  | *Solanum aculeatissimum* Jacq. | 98.04 | 98 | 14.64 | 0.96 |
|  | *Mimusops bagshawei* S. Moore | 97.06 | 96 | 10.07 | 0.94 |
|  | *Syzygium guineense* (Willd.) DC | 96.08 | 97 | 8.59 | 0.95 |
|  | *Syzygium cordatum*Hochst. | 99.02 | 99 | 25.76 | 0.97 |
|  | *Carissa spinarum*L. | 98.04 | 99 | 15.04 | 0.97 |
|  | *Vachellia sieberiana*(DC.) Kyal. & Boatwr. | 99.02 | 99 | 27.88 | 0.97 |
|  | *Tarchonanthus camphoratus* L. | 100 | 100 | 28.72 | 0.98 |
|  | *Flacourtia indica* (Burm.f.) Merr. | 99.02 | 101 | 17.76 | 0.99 |
|  | *Commelina cyanea*R.Br. | 98.04 | 101 | 22.40 | 0.99 |
|  | *Psidium guajava* L. | 99.02 | 98 | 20.43 | 0.96 |
|  | *Ageratum conyzoides* L. | 99.02 | 101 | 29.53 | 0.99 |
|  | *Tabernaemontana stapfiana* Britten | 50.98 | 56 | 0.55 | 0.55 |
|  | *Helichrysum schimperi* (Sch.Bip. ex A. Rich.) Moeser | 97.06 | 95 | 9.61 | 0.93 |
|  | *Zehneria scabra* Sond. | 95.10 | 93 | 5.31 | 0.91 |
|  | *Gerrardanthus lobatus*(Cogn.) C.Jeffrey | 95.10 | 93 | 9.95 | 0.91 |
|  | *Phragmanthera usuiensis* (Oliver) M.G.Gilbert | 90.20 | 97 | 8.38 | 0.95 |
|  | *Zehneria minutiflora* (Cogn.) C.Jeffrey | 93.14 | 96 | 5.88 | 0.94 |
|  | *Richardia scabra* L. | 100 | 96 | 25.81 | 0.94 |
|  | *Cassipourea malosana* (Baker) Alston | 100 | 96 | 10.72 | 0.94 |
|  | *Croton megalocarpus Hutch.* | 81.37 | 88 | 3.15 | 0.86 |
|  | *Plantago palmata* Hook.f. | 97.06 | 100 | 23.21 | 0.98 |
|  | *Amaranthus graecizans* L. | 89.22 | 94 | 5.35 | 0.92 |
|  | *Physalis peruviana* L*.* | 99.02 | 99 | 20.78 | 0.97 |
|  | *Amaranthus dubuis* Mart. | 97.06 | 101 | 24.09 | 0.99 |
|  | *Tiliacora kenyensis Troupin* | 96.08 | 100 | 15.10 | 0.98 |
|  | *Indigofera arrecta* Hochst. ex A.Rich*.* | 96.08 | 97 | 7.17 | 0.95 |
|  | *Triumfetta brachyceras*K.Schum. | 98.04 | 101 | 19.88 | 0.99 |
|  | *Hibiscus diversifolius* Jacq. | 93.14 | 99 | 12.27 | 0.97 |
|  | *Rumex abyssinicus* Jacq. | 93.14 | 99 | 17.36 | 0.97 |
|  | *Markhamia lutea* (Benth.) K. Schum | 97.06 | 98 | 23.25 | 0.96 |
|  | *Ficus sur*Forssk. | 100 | 99 | 28.52 | 0.97 |
|  | *Protea gaguedi* J.F.Gmel. | 97.06 | 101 | 25.45 | 0.99 |
|  | *Landolphia buchananii Stapf* | 91.18 | 93 | 8.86 | 0.91 |
|  | *Rubus steudneri* Scweinf. | 91.18 | 93 | 6.23 | 0.91 |
|  | *Rubus pinnatu*s Willd. | 91.18 | 100 | 8.60 | 0.98 |
|  | *Gymnanthemum amygdalinum*(Delile) Sch.Bip. ex Walp. | 91.18 | 100 | 9.39 | 0.98 |
|  | *Sorghum bicolor* (L.) *Moench* | 97.06 | 99 | 19.13 | 0.97 |
|  | *Ocimum tenuiflorum* L. | 95.10 | 93 | 7.40 | 0.91 |
|  | *Lagenaria abyssinica* (Hook.f) C.Jeffrey | 100 | 99 | 26.96 | 0.97 |
|  | *Hibiscus calyphyllus* Cav. | 96.08 | 96 | 11.98 | 0.94 |
|  | *Hydrocotyle hirsuta var.* hirsute | 95.10 | 99 | 10.65 | 0.97 |
|  | *Centella asiatica* (L.) Urb. | 97.06 | 98 | 15.20 | 0.96 |
|  | *Olea welwitschii* Gilg & G.Schellenb. | 91.18 | 95 | 5.87 | 0.93 |
|  | *Albizia coriaria* Welw. ex Oliv. | 95.10 | 96 | 10.22 | 0.94 |
|  | *Olinia rochetiana* A.Juss. | 96.08 | 98 | 17.16 | 0.96 |
|  | *Ehretia cymosa* Thonn. | 94.12 | 98 | 9.48 | 0.96 |
|  | *Azadirachta indica* A.Juss. | 98.04 | 100 | 22.60 | 0.98 |
|  | *Lippia kituiensis* Vatke | 100 | 100 | 18.36 | 0.98 |
|  | *Cyathula tomentosa*(Roth) Moq. | 100 | 97 | 18.81 | 0.95 |
|  | *Acanthus pubescens* (Oliv.)Engl | 97.06 | 89 | 3.86 | 0.87 |
|  | *Basella alba* L. | 88.24 | 99 | 10.92 | 0.97 |
|  | *Garcinia buchananii* Bak. | 96.08 | 97 | 9.57 | 0.95 |
|  | *Musa acuminata*Colla | 95.10 | 86 | 3.88 | 0.84 |
|  | *Fuerstia africana* T.C.E.Fr. | 85.29 | 101 | 14.77 | 0.99 |
|  | *Cyathula cylindrica* Moq | 99.02 | 98 | 7.85 | 0.96 |
|  | *Leucas calostachys* Oliv. | 92.16 | 82 | 1.89 | 0.80 |
|  | *Balanites aegyptiaca* (L.) Delile | 80.39 | 100 | 8.25 | 0.98 |
|  | *Fagaropsis angolensis* (Engl.) H.M.Gardner | 94.12 | 95 | 4.54 | 0.93 |
|  | *Loeseneriella africana*(Willd.) R.Wilczek | 94.12 | 98 | 8.67 | 0.96 |
|  | *Dovyalis abysinicca* (A. Rich.) Warb | 94.12 | 91 | 3.69 | 0.89 |
|  | *Lepidagathis scariosa* Nees | 87.25 | 94 | 5.43 | 0.92 |
|  | *Oxalis corniculata* L. | 96.08 | 86 | 2.56 | 0.84 |
|  | *Zanthoxylum chalybaeum* Engl. | 79.41 | 89 | 3.86 | 0.87 |
|  | *Persea americana* Mill. | 92.16 | 94 | 9.78 | 0.92 |
|  | *Carica papaya* L. | 97.06 | 96 | 12.91 | 0.94 |
|  | *Acmella*Rich. ex Pers. | 90.20 | 86 | 6.58 | 0.93 |
|  | *Kigelia africana* (Lam.) Benth | 88.24 | 97 | 3.53 | 0.84 |
|  | *Ocimum gratissimum* L. | 93.14 | 97 | 7.16 | 0.95 |
|  | *Rauvolfia caffra* Sond. | 95.10 | 95 | 17.72 | 0.93 |
|  | *Gouania longispicata* Engl. | 97.06 | 97 | 17.93 | 0.95 |
|  | *Microglossa pyrifolia* (Lam.) O.Kuntze | 99.02 | 101 | 11.04 | 0.99 |
|  | *Justicia flava* Vahl | 94.12 | 94 | 6.96 | 0.92 |
|  | *Zanthoxylum gilletii* (De Wild.) P.G.Waterman | 95.10 | 97 | 8.73 | 0.95 |
|  | *Polyscias kikuyuensis* Summerh. | 98.04 | 95 | 16.38 | 0.93 |
|  | *Lepidotrichilia volkensii* (Gürke) J.-F.Leroy | 91.18 | 95 | 7.14 | 0.93 |
|  | *Cordia africana* Lam. | 92.16 | 91 | 4.96 | 0.89 |
|  | *Podocarpus latifolius* (Thunb.) R.Br. ex Mirb. | 89.22 | 92 | 5.59 | 0.90 |
|  | *Ensete ventricosum* (Welw.) Chessman | 92.16 | 96 | 6.33 | 0.94 |
|  | *Spathodea campanulata* Buch.-Ham. ex DC. | 89.22 | 86 | 3.16 | 0.84 |
|  | *Ficus sycomorus* L. | 97.06 | 96 | 15.03 | 0.94 |
|  | *Albizia gummifera* sensu Capuron, p.p. | 97.06 | 96 | 13.90 | 0.94 |
|  | *Senna didymobotrya*(Fresen.) H.S.Irwin & Barneby | 93.14 | 96 | 12.27 | 0.94 |
|  | *Laggera crispata*(Vahl) Hepper & J.R.I.Wood | 94.12 | 97 | 7.30 | 0.95 |
|  | *Vachellia nilotica*(L.) *P.J.H.Hurter & Mabb.* | 84.31 | 96 | 3.86 | 0.94 |
|  | *Clematis simensis* Fresen. | 98.04 | 98 | 14.21 | 0.96 |
|  | *Mimulopsis solmsii* Schweinf. | 95.10 | 96 | 8.78 | 0.94 |
|  | *Chlorophytum gallabatense*Schweinf. ex Baker | 89.22 | 87 | 3.22 | 0.85 |
|  | *Solanum aculeastrum* Dunal | 88.24 | 88 | 3.69 | 0.86 |
|  | *Lagenaria siceraria* (Molina) Standl. | 96.08 | 97 | 12.20 | 0.95 |
|  | *Dombeya torrida* (J.F.Gmel.) Bamps | 97.06 | 98 | 16.34 | 0.96 |
|  | *Buckollia volubilis*(Schltr.) Venter & R.L.Verh. | 94.12 | 94 | 7.25 | 0.92 |
|  | *Cyphostemma orondo* (Gilg & M.Brandt) Desc. | 80.39 | 87 | 2.44 | 0.85 |
|  | *Ficus thonningii*Blume | 86.27 | 92 | 3.19 | 0.90 |
|  | *Periploca linearifolia* Dillon & A.Rich. | 95.10 | 98 | 17.46 | 0.96 |
|  | *Rubia cordifolia* L. | 91.18 | 96 | 12.04 | 0.94 |
|  | *Clerodendrum johnstonii* Oliv. | 87.25 | 98 | 13.70 | 0.96 |
|  | *Searsia pyroides*(Burch.) Moffett | 93.14 | 95 | 6.55 | 0.93 |
|  | *Searsia natalensis*(Bernh. ex C.Krauss) F.A.Barkley | 94.12 | 100 | 7.48 | 0.98 |
|  | *Ocimum basilicum* L. | 99.02 | 94 | 4.23 | 0.92 |
|  | *Urtica massaica* Mildbr. | 98.04 | 100 | 18.38 | 0.98 |
|  | *Warburgia ugandensis* Sprague | 98.04 | 97 | 16.44 | 0.95 |
|  | *Polyscias fulva* (Hiern) Harms | 97.06 | 102 | 15.56 | 1.00 |
|  | *Phoenix reclinata* Jacq*.* | 99.02 | 98 | 8.56 | 0.96 |
|  | *Baccharoides lasiopus* (O.Hoffm.) H.Rob. | 94.12 | 92 | 10.02 | 0.90 |
|  | *Scutia myrtina  Kurz* | 99.02 | 96 | 18.06 | 0.94 |
|  | *Stephania abyssinica* Walp. | 88.24 | 93 | 4.38 | 0.91 |
|  | *Dodonaea viscosa Jacq* var. *angustifolia* (L.f) Benth*.* | 87.25 | 94 | 4.31 | 0.92 |
|  | *Oncoba routledgei* Sprague | 98.04 | 102 | 21.06 | 1.00 |
|  | *Aloe kedongensis* Reynolds | 86.27 | 95 | 5.35 | 0.93 |
|  | *Gloriosa superba* L. | 95.10 | 98 | 8.99 | 0.96 |
|  | *Juniperus procera Hochst. ex Endl.* | 98.04 | 99 | 15.98 | 0.97 |
|  | *Gymnanthemum auriculiferum*(Hiern) Isawumi | 96.08 | 99 | 16.29 | 0.97 |
|  | *Croton macrostachyu*s Del. | 94.12 | 96 | 4.84 | 0.94 |
|  | *Oldeania alpina*(K.Schum.) Stapleton | 97.06 | 101 | 32.11 | 0.99 |
|  | *Acanthus eminens*  C.B.Clarke | 96.08 | 91 | 6.49 | 0.89 |
|  | *Ekebergia capensis* Sparrm. | 88.24 | 101 | 4.81 | 0.99 |
|  | *Prunus africana* (Hook.f) Scweinf. | 97.06 | 98 | 19.17 | 0.96 |
|  | *Calpurnia aurea (Aiton) Benth. subsp.aurea* | 95.10 | 97 | 15.11 | 0.95 |
|  | *Pteridium aquilinum* (L.) Kuhn | 98.04 | 100 | 15.89 | 0.98 |
|  | *Vachellia hockii*(De Wild.) Seigler & Ebinger | 98.04 | 99 | 15.31 | 0.97 |
|  | *Rhynchosia hirta* (Andrews) Meikle & Verdc | 98.04 | 97 | 12.67 | 0.95 |
|  | *Rhoicissus tridentata* (L.f.) Wild & R.B.Drumm. | 90.20 | 94 | 6.00 | 0.92 |
|  | *Nicotiana rustica* L. | 94.12 | 99 | 9.53 | 0.97 |
|  | *Chaetacmi aristata* Planch. | 99.02 | 99 | 16.67 | 0.97 |
|  | *Clutia abysinicca Jaub.& Spach* | 88.24 | 89 | 3.73 | 0.87 |
|  | *Euclea divinorum* Hiern | 92.16 | 100 | 35.81 | 0.98 |
